# Sulfation of Heparan and Chondroitin Sulfate Ligands Enables Cell-Specific Homing of Nanoprobes

**DOI:** 10.1002/chem.202202622

**Published:** 2022-12-14

**Authors:** Sandhya Mardhekar, Balamurugan Subramani, Prasanna Samudra, Priyadharshini Srikanth, Virendrasinh Mahida, Preeti Ravindra Bhoge, Suraj Toraskar, Nixon M. Abraham, Raghavendra Kikkeri

**Affiliations:** [a] Department of Chemistry, Indian Institute of Science Education and Research, Dr. Homi Bhabha Road, Pune-411008 (India); [b] Laboratory of Neural Circuits and Behaviour (LNCB), Department of Biology, Indian Institute of Science Education and Research, Dr. Homi Bhabha Road, Pune-411008, (India)

**Keywords:** CD44 receptor, chondrotin sulfate, gold nanoparticles, heparan sulfate, neural cell lines

## Abstract

Demystifying the sulfation code of glycosaminoglycans (GAGs) to induce precise homing of nanoparticles in tumor cells or neurons influences the development of a potential drug- or gene-delivery system. However, GAGs, particularly heparan sulfate (HS) and chondroitin sulfate (CS), are structurally highly heterogeneous, and synthesizing well-defined HS/CS composed nanoparticles is challenging. Here, we decipher how specific sulfation patterns on HS and CS regulate receptor-mediated homing of nanoprobes in primary and secondary cells. We discovered that aggressive cancer cells such as MDA-MB-231 displayed a strong uptake of GAG-nanoprobes compared to mild or moderately aggressive cancer cells. However, there was no selectivity towards the GAG sequences, thus indicating the presence of more than one form of receptor-mediated uptake. However, U87 cells, olfactory bulb, and hippocampal primary neurons showed selective or preferential uptake of CS-E-coated nanoprobes compared to other GAG-nanoprobes. Furthermore, mechanistic studies revealed that the 4,6-*O*-disulfated-CS nanoprobe used the CD44 and caveolin-dependent endocytosis pathway for uptake. These results could lead to new opportunities to use GAG nanoprobes in nanomedicine.

## Introduction

In recent years, carbohydrate-appended nanoparticles have provided a rich platform for designing diagnostic and cell-imaging tools for biomedical applications.^[1]^ Though relatively weak binding with protein, carbohydrates play a pivotal role in fine-tuning biological activities.^[2]^ Hence, glyconanoparticles are a key construct to target lectins,^[3]^ protein toxins,^[4]^ cell-surface receptors,^[5]^ cell lines and tissues.^[6]^ Of the plethora of nano-structural materials employed in designing glyconanoparticles, gold nanoparticles (AuNPs) are arguably one of the most common, as AuNPs are easy to synthesis with different sizes, shapes, and functionalizations.^[7]^ Many successful glyco-AuNPs contain scaffold features, including α-fucosylamine and α-mannose functionalized gold nanoparticles targeting DC-SIGN and viral infection.^[8]^ Several lectins biosensors, and carbohydrate-carbohydrate interactions were established using gold nanoparticles.^[9]^ However, the major challenge will be acquiring a pure form of carbohydrate ligands to synthesize glyconano-particles, more specifically, the synthesis of glycosaminoglycans (GAGs) and sialic acid-based glyconanoparticles, which regulate major biological functions.

Among various GAGs, heparan sulfate (HS) and chondroitin sulfate (CS) offers attractive scaffolds due to their structural diversity and ability to demonstrate wide biological applications.^[10]^ Synthetic heparin-based nanoprobes were developed for various applications such as antimalarial drug delivery system,^[11]^ targeting glioma in the murine model,^[12]^ delivery of siRNA to treat lung cancer,^[13]^ targeted delivery of doxorubicin^[14]^ and targeting anti-metastasis of orthotopic breast cancer.^[15]^ In similar line various synthetic CS-based materials were developed to target cancer metastasis.^[16]^ CS-based liposomes were reported for co-delivery of doxorubicin and retinoic acid to treat lung metastasis,^[17]^ similarly CS-based dendrimers and star-like microfibers were developed to target growth factors.^[18]^ Despite all this progress, there are no HS- or CS-based nanomaterials in clinical trials. This is mainly due to the anticoagulation activity of native HS and strong binding affinity to more than one receptor. Therefore, it is critical to synthesize well-defined HS and CS sequences and study their activity. Herein, we report the synthesis and characterization of a panel of HS tetrasaccharides and CS disaccharides appended to fluorescent nanoprobes to study selective cell targeting and homing. Although HS- and CS-based nanoprobes have been studied independently as a cell targeting and drug-delivery system, to the best of our knowledge, comparative studies have never been explored with synthetic GAG-nanoprobes.

## Results and Discussion

At the structural level, CS is a heteropolysaccharide that comprises galactosamine-glucuronic acid (GluA) disaccharides repeating unit with different O-sulfation patterns.^[19]^ Based on their sulfation codes, they are further classified as CS–A, C, D and E, respectively. In contrast, HS consists of disaccharide repeating units of glucosamine-uronic acid, with a wide range of sulfation patterns, including N-sulfation and *O*-sulfation.^[20]^ Uronic acid can be in the form of l-iduronic acid (IdoA) or d-glucurnoic acid (GluA). Theoretically, there are 2304 possible tetrasaccharides, which allow fine-tuning the carbohydrate-protein interactions. Hence, synthetic GAG nanoprobes are extremely valuable scaffolds for constructing cell- and tissue-specific drug delivery probes. Recently, we reported the synthesis of a heparinoid-nanovehicle to target epidermal growth factor receptors (EGFR)-overexpressed cancer cells in 2D and 3D co-culture models.^[21]^ However, broadening the cell-specific homing of nanovehicles requires synthetic GAG nanoprobes of HS and CS epitopes. Therefore, we synthesized HS and CS ligands predominantly found in GAGs to improve the selectivity and specificity of homing nanoprobes in phenotypic cells.

Over the past decade, many research groups pioneered in carbohydrate chemistry developed elegant synthetic strategies to synthesize well-defined HS and CS oligosaccharides.^[22]^ Using these strategies with slight modifications in the protecting groups, we have synthesized three HS tetrasaccharides and four CS disaccharides and assembled them on gold nanoparticles. The synthesis of core monosaccharides (**1**–**4**) was achieved by standard literature procedure.^[23]^ The synthesis of disaccharide **5** involved the glycosylation between trichloroacetimidate donor **1** and thiotoluene acceptor **2** in the presence of silver trifluromethansulfonate (AgOTf) activation condition in CH_2_Cl_2_ at −40 °C to RT for 40 min to furnished selective α-glycosylation with 60% yield. Similarly, thioglycoside donor **2** and glucose acceptor 4 in the presence of *N*-Iodosuccinimide (NIS) and Trimethylsilyltrifluoromethanesulfonate (TMSOTf) at −20 °C to RT for 10 min yielded 78% of selective α-glycosylated disaccharide **6**.

**Scheme 1:**
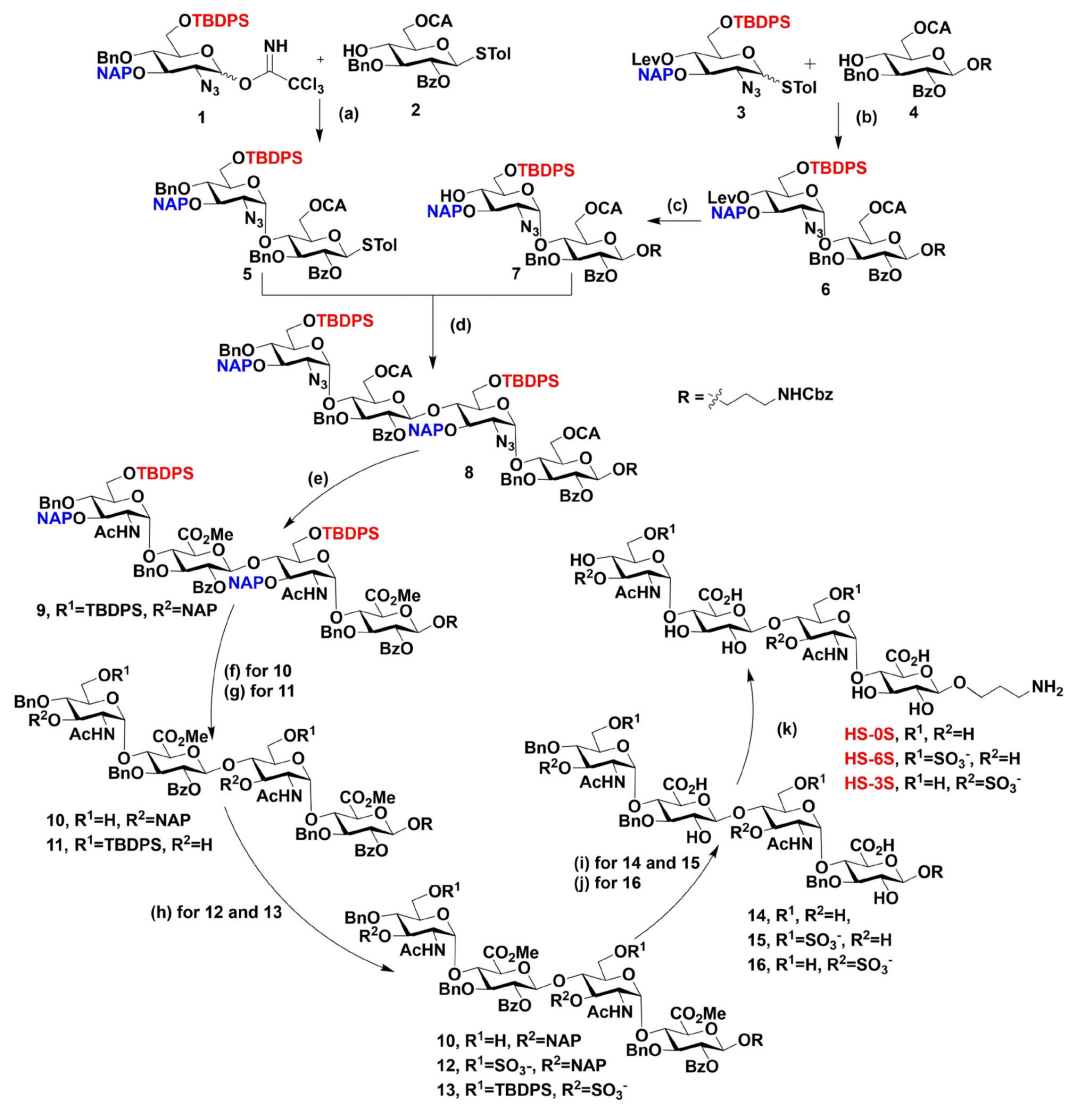
Reagents and conditions. a) AgOTf in CH_2_Cl_2_, −40°C-RT, 40 min, 6%; b) NIS, TMSOTf in CH_2_Cl_2_, −20°C to RT, 10 min, 78%; c) N_2_H_4_.H_2_O, AcOH in CH_2_Cl_2_, RT, 3 h, 7%; d) NIS, TMSOTf in CH_2_Cl_2_, −20°C to RT, 10 min, 72%; e) i: thiourea in Py/MeOH (1 : 1), 80°C, 2 h, 95%; ii: TEMPO, BAIB in CH_2_Cl_2_/H_2_O (2: 1), RT, 6 h; iii: MeI, K_2_CO_3_ in DMF, RT, 12 h, 62% (over two steps); iv: Zn dust, THF/AcOH/Ac_2_O (3 : 2 : 1), 0°C, 12 h, 79%; f) 70% HF·py in pyridine, 0°C, 24 h, 75%; g) DDQ in CH_2_Cl_2_/H_2_O (18 : 1), RT, 2 h, 70%; h) SO_3_·TMA in DMF, MW 100°C, 15 W, 15 min, 76% yield for **12**, 69% yield for **13**; i) 1 M LiOH in THF/H_2_O (1 : 1), RT, 18 h, 80% yield for **14**, 70% yield for **17**; j) i: 70% HF·py in pyridine, 0°C, 24 h, ii: 1 M LiOH in THF/H_2_O (1 : 1), RT, 18 h, 60% yield for **16** (over two steps); k) H_2_, Pd(OH)_2_ in H_2_O, RT, 36 h, 85% yield for **HS0S**, 75% yield for **HS6S**, 77% yield for **HS3S**.

With donor **5** and acceptor **6** in hand, we went ahead for the synthesis of tetrasaccharide. Initially, levulinic acid group of deprotected from **6** with hydrazine hydrate and its stereo-selective glycosylation with NIS and TMSOTf promoter in a CH_2_Cl_2_ solvent at −20 °C furnished compound **8** in 72% yield in 1 : 9 α/β selectivity. At this stage of the synthesis, a chloroacetate group of **8** was deprotected with thiourea, followed by 2,2,6,6-tetramethyl-1-piperidinyloxyl free radical (TEMPO) and [bis(acetoxy)iodo]benzene (BAIB)-mediated oxidation and esterification, further reduction of the azide group with zinc dust, followed by acetylation, resulted in the *N*-acetate derivative HS precursor **9**. Finally, the selective removal of *tert*-butyldiphenylsilyl (TBDPS) or 2-naphthylmethyl (NAP) deprotection of **9**, followed by sulfation using the microwave method. HS tetrasaccharides **10** and **11** were selectively O-sulfated under the microwave conditions using the sulfur trioxide trimeth-ylamine complex (SO_3_·TMA) complex in DMF at 100 °C for 15 min and followed by global deprotection provided HS tetrasaccharides **HS6S** and **HS3S** with 75 and 77% yield, respectively, whereas global deprotection of **9** produced in **HS0S** with 85% yield (Scheme 1).

**Figure 1:**
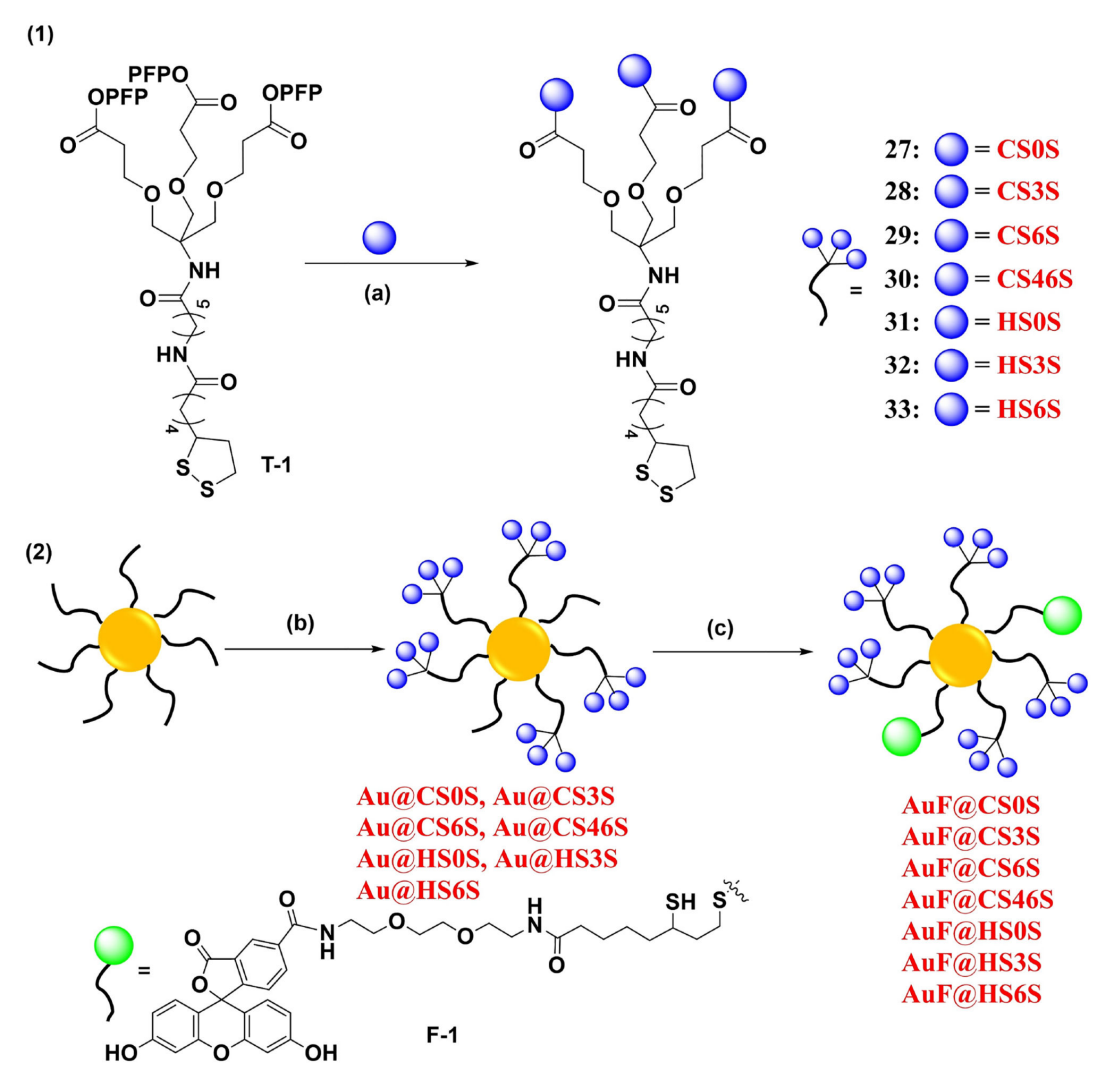
1) CS and HS conjugation to the tripod. 2) Schematic representation of HS- and CS-tripod-functionalized fluorescent AuNPs. Reagents and conditions: a) DMF, Et_3_N, RT, 5 h b) **27**–**33**, PBS buffer pH 7.4, 25°C, 24 h; c) **F-1**, PBS buffer pH 7.4, 25°C, 12 h.

**Scheme 2:**
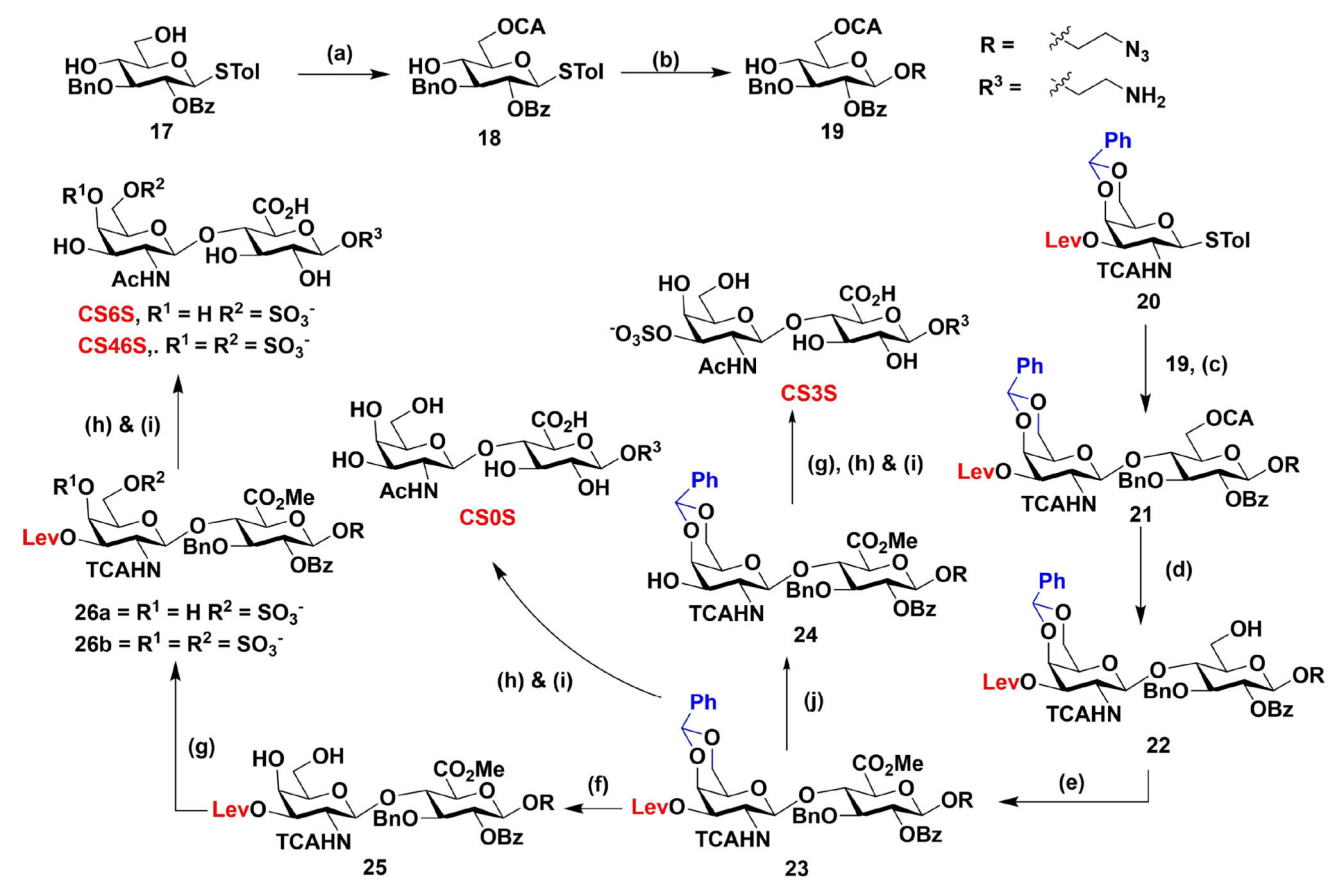
Reagents and conditions a) (ClAcO)_2_O, CH_2_Cl_2_/Py (4 : 1) −40°C, 1 h, 78%; b) azidoethanol, NIS, TMSOTf, CH_2_Cl_2_, 4 Å MS–20°C, 68%; c) NIS, TMSOTf, CH_2_Cl_2_, 4 Å MS 0°C, 81%; d) thiourea, Py/MeOH (1 : 1) 80°C, 2 h, 88%; e) i: TEMPO, BAIB, CH_2_Cl_2_/*t*BuOH/H_2_O (4 : 1 : 1), RT, 6 h; ii: MeI, K_2_CO_3_ DMF, RT, 12 h, 82%; f) PTSA, CH_2_Cl_2_/MeOH (2 : 1), RT, 6 h, 88%; g) SO_3_·TEA, DMF, MW 100°C, 15 W, 15 min, 66% of **26 a**, 82% of **26 b**; h) i: LiOH·H_2_O, THF/H_2_O (2 : 1), 80°C, 12 h; ii: Ac_2_O, TEA, MeOH, 0°C to RT, 12 h; i) Pd(OH)_2_, H_2_, H_2_O, RT, 24 h; j) AcOH/NH_2_NH_2_·H_2_O (2.5 : 1), THF/MeOH (10 : 1), RT, 1 h, 80%.

Next, we synthesized galactosamine donor **20** and glucose acceptor **17** from d-galactosamine and d-glucose in **6** and **7** steps, respectively, as reported in the literature.^[23b,24]^ The glucose donor **19** was further modified with 6-*O*-chloroacetate protection, followed by linker glycosylation that yielded accept- or **19** in 68% yield. Glycosylation of **20** and **19** in presence of NIS and TMSOTf promoter at RT yielded completely stereo-specific disaccharide precursor **21**. Then, chloroacetate deprotection of **21**, followed by subsequent oxidation and esterification, yielded **23** in 82% yield, which was subjected to a selective deprotection strategy to obtain CS disaccharide derivatives. In brief, delevulinilation of **23**, followed by sulfation with sulfur trioxide triethylamine complex (SO_3_·TEA) complex and subsequent hydrolysis, N-acetylation, and hydrogenolysis provided the final **CS3S**. Similarly, *para*-toluenesulfonic acid (PTSA) assisted benzylidene deprotection of **23**, followed by sulfation with appropriate equivalents of SO_3_·TEA complex (5 and 10 equiv.), followed by global deprotection, produced **CS6S** and **CS46S** derivatives (Scheme 2). **23** was subjected to saponification and subsequent global deprotection yielded **CS0S**. These GAG sequences were tethered on tripods (**T-1**), to achieve subsequent gold nanoparticle immobilization (Figure 1).

Spherical gold nanoparticles of 20 nm diameter are ideal for the designing of GAG nanoprobes as they are well-established for carbohydrate-mediated cell targeting studies.^[25]^

The size and shape of the nanostructure were confirmed by SEM and UV-visible spectroscopy (Figure S1). The AuNPs were treated with GAG tripods (**27**–**33**) and a fluorescent linker **F-1** in a step-by-step method to obtain fluorescent GAG nanoprobes (AuF@**CS0S**, AuF@**CS3S**, AuF@**CS6S**, AuF@**CS46S**, AuF@**HS0S**, AuF@**HS6S**, and AuF@**HS3S**). Zeta-potential and fluorescent spectroscopy measurements, fluorescent intensity coefficient confirmed the immobilization of the GAGs and the similar concentration of fluorescent probes on the nanostructure. Finally, the amount of sugar immobilized on the nanostructure was confirmed by the thio-detection kit (Table S1).^[26]^

To establish the influence of the HS and CS structures on cellular uptake and homing of nanoparticles, we conducted a fluorescent imaging experiment with primary and secondary cell lines. The choice of the specific cell line was based on the fact that cancer and neural cell lines express various endocytosis growth factor receptors and they use HS/CS ligands as co-receptors to mediate the endocytosis process.^[27]^

**Figure 2:**
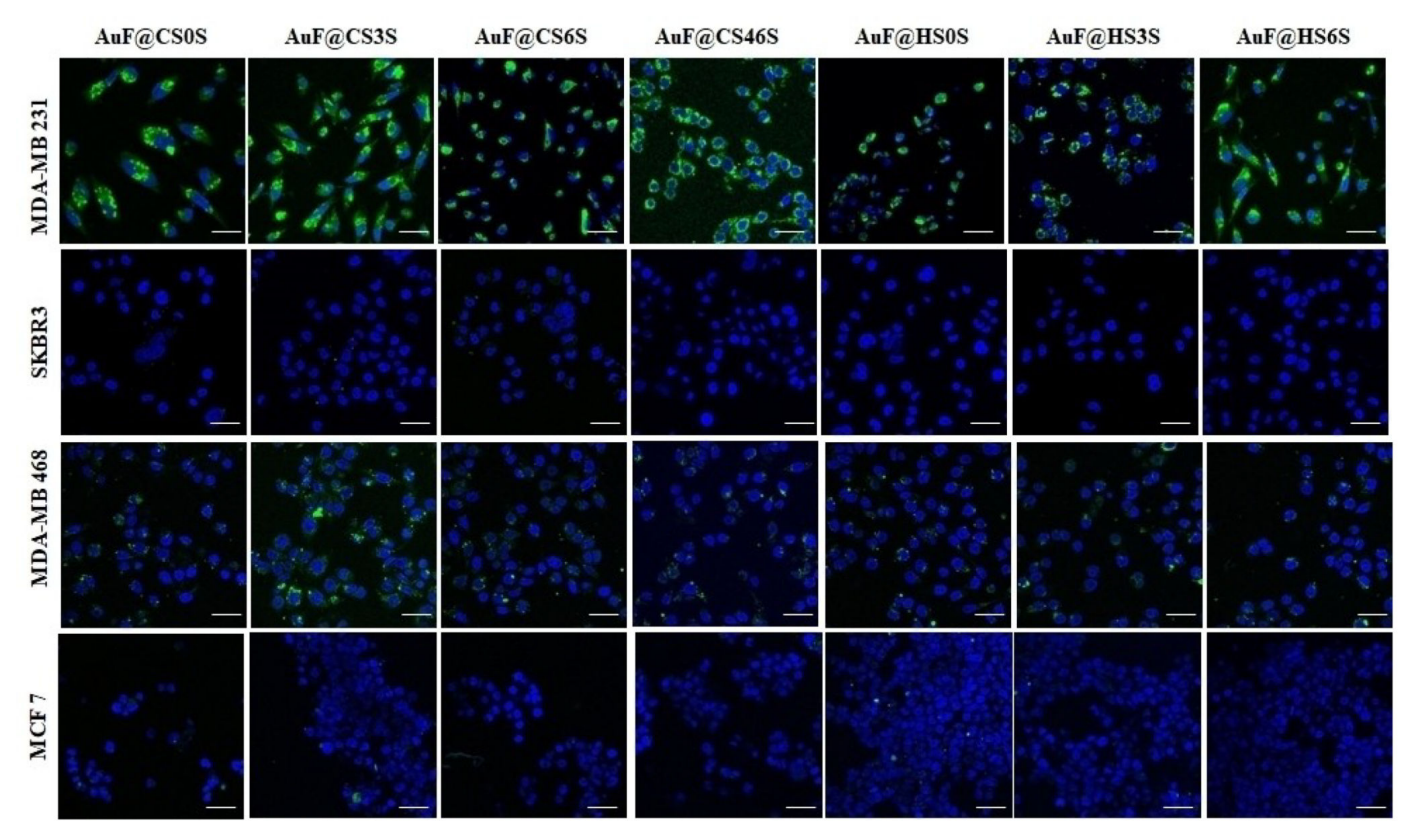
Confocal images of nanoparticle internalization by different cancer cell lines after 4 h; scale bars: 50 μm.

A cellular uptake assay was performed using the protocols reported in the literature.^[28]^ In brief, cells were seeded in an eight-well confocal imaging chamber and allowed to grow for 24 h at 37°C in a 5% CO_2_ incubator. GAG nanoprobes (5 μg mL^−1^) were added, and live imaging was performed after 2 and 4 h. To ensure consistency, we repeated all experiments in triplicate. We first used cancer cell lines MDA-MB-231, MDA-MB-468, T47D, MCF7, and SKBR3, which represent different aggression levels of breast cancer with a wide range of growth factor binding receptors.[29] After 4 h of incubation, fluorescence was observed in the triple-negative cancer cells (MDA-MB-231, T47D, and MDA-MB-468). Among them, the MDA-MB-231 cell line showed intense fluorescence with all of the GAG nanoprobes, while MCF7 and SKBR3 did not show any uptake, confirming that cancer cells with different levels of aggression behave differently with GAG nanoprobes. Although triple-negative cancer cells showed strong uptake of the GAG nanoprobes, there was no specificity, which suggested that glucuronic acid-based HS or CS nanoprobes are not suitable probes to target cancer cells (Figure 2).

**Figure 3:**
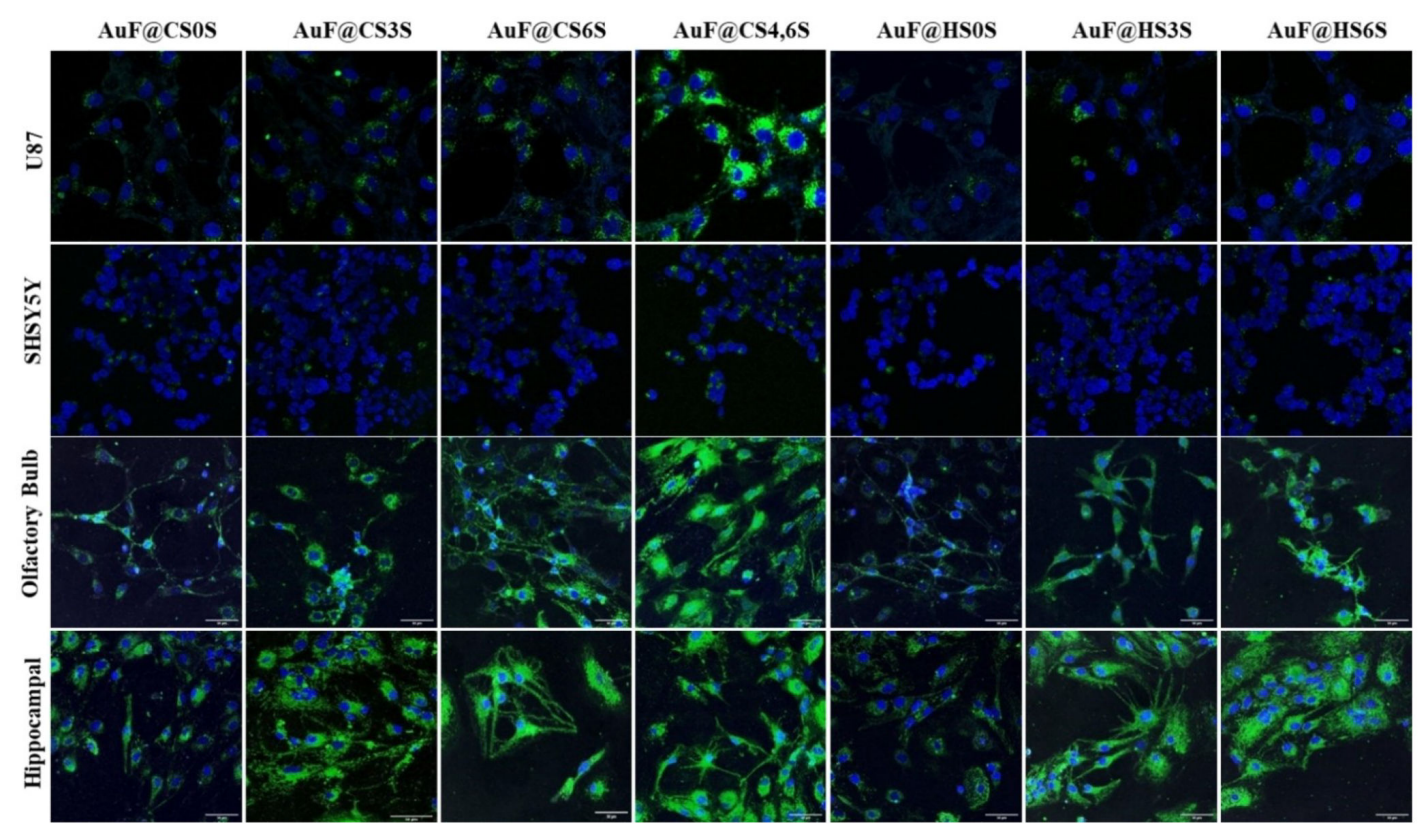
Confocal images of nanoparticle internalization by primary and secondary neural cell lines after 4 h; scale bars: 50 μm.

Next, we performed an in-vitro assay with glial cells U87 and neuroblastoma cells SH-SY5Y, as these are widely used to probe the molecular and cellular mechanisms of brain disorders. Interestingly, the U87 glial cells showed a pronounced uptake of AuF@**CS46S** as compared to the other GAG nanoprobes, while SH-SY5Y showed non-specific and poor uptake of the GAG nanoprobes (Figure 3). To show the rate of uptake of the GAG nanoprobes, a flow cytometry assay was performed. After 4 h, AuF@**CS46S** showed 37% uptake by the U87 cell line compared to the other GAG nanoprobes (10–12%; Figure S2). These findings suggest that AuF@**CS46S** binds to the cell-surface receptors through specific carbohydrate-protein inter-action and undergo endocytosis.

**Figure 4:**
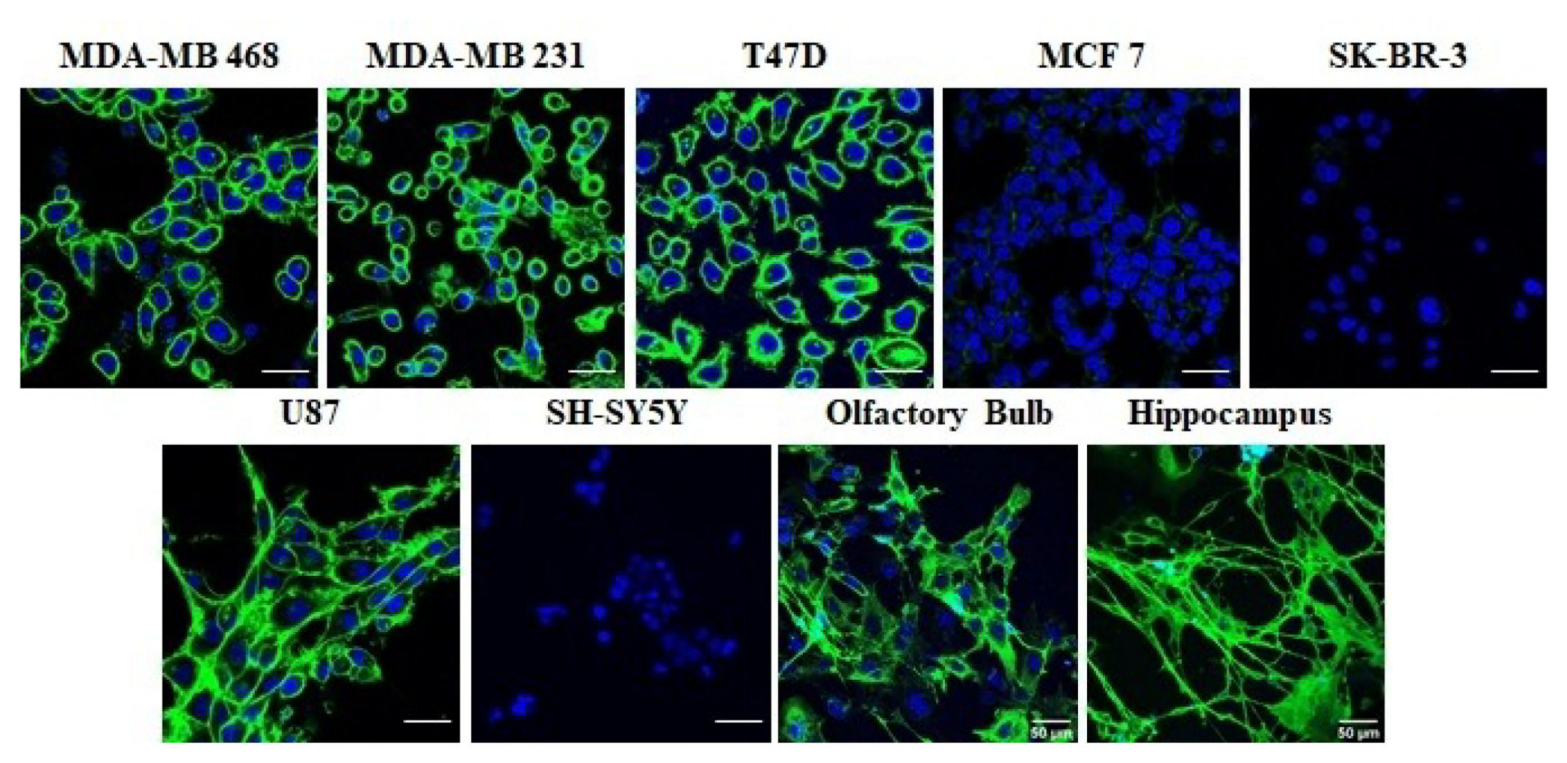
CD44 expression levels for different cancer and neural cell lines.

To further confirm the GAG nanoprobes’ targeting of the neural cells, we used two primary neuronal cells isolated from the hippocampal and olfactory bulb regions of newborn mice. Fluorescent imaging studies of the olfactory bulb sections showed an increased uptake of two- to three fold of AuF@**CS46S** as compared to the other ligands. While the hippocampal cells showed a similar trend with AuF@**CS46S** (Figure S4) uptake was less pronounced as compared to olfactory bulb cells. These findings indicate that glial cells and primary neurons might have a specific receptor for the **CS46S** ligand that mediated the uptake (Figure 3). To understand the mechanism of endocytosis of AuF@**CS46S**, we first investigated different GAGs binding receptors expressed on these cell lines. The literature survey hypothesized that CD44 a transmembrane GAG binding receptor ubiquitously expressed on cancer and neural cells, might be involved in this uptake mechanism. To validate CD44-mediated uptake of GAG-nanoprobes, we first investigated the expression level of CD44 on cancer and neuronal cell lines. All cells that took up GAG nanoprobes exhibited strong cell-surface expression of CD44 receptors. In contrast, SH-SY5Y, MCF-7, and SKBR3 showed no staining of FITC-anti-CD44 protein, indicating that CD44 might be a potential receptor for GAG-nanoprobes uptake (Figure 4).

**Figure 5:**
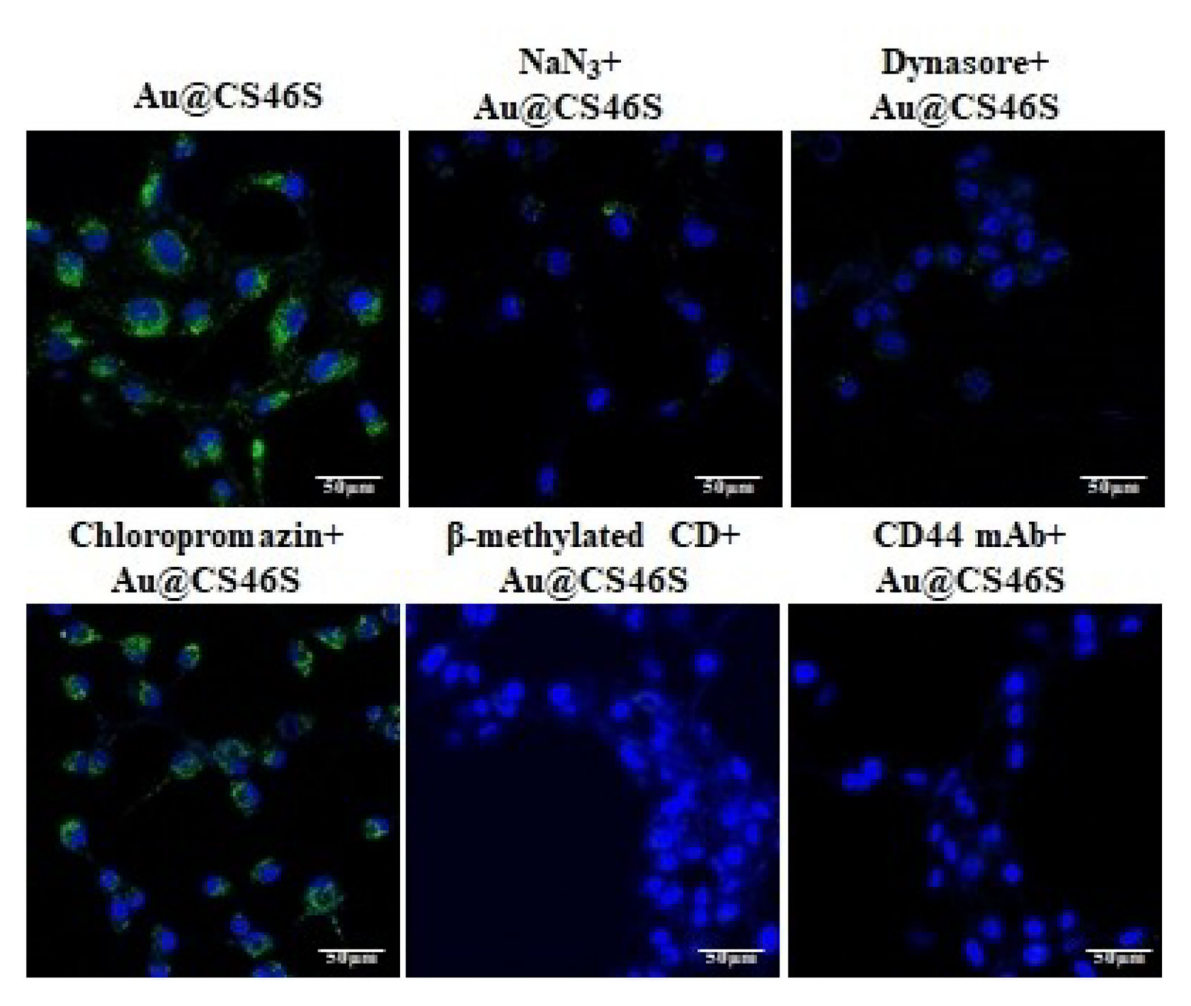
Confocal microscopy images for the uptake of AuF@**CS46S** by U87 cells in the presence of different endocytotic pathway inhibitors; *n* = 3; scale bars: 50 μm.

Next, we investigated receptor-mediated uptake of AuF@**CS46S** by the U87 cell line. To this end, we treated the cell lines with NaN_3_ solution for 30 mins, followed by treating AuF@**CS46S** for 4 h. We observed a substantial decrease in the cellular uptake of AuF@**CS46S**, indicating a receptor-mediated endocytic pathway. Next, we confirmed whether the uptake is dynamin-dependent by adding dynasore hydrate inhibitor into the cell culture medium. AuF@**CS46S** internalization was fully inhibited, revealing that the internalization mechanism follows either clathrin or caveolae-dependent pathway. Finally, we studied the effect of methyl-β-cyclodextrin (m-β-CD, inhibitor of caveolae-mediated endocytosis), chlorpromazine (inhibitor of clathrin-mediated endocytosis). As shown in Figure 5, cells pretreated with the caveolae inhibitor showed a considerable reduction in internalization, whereas chlorpromazine pre-treatment of cells exhibited a minor inhibition effect. Similarly cells pretreated with CD44 monoclonal antibody showed completely reduced uptake of AuF@**CS46S**. These findings revealed that AuF@**CS46S** was taken up by the CD44 receptor-mediated energy-dependent caveolae pathway. Similar studies with olfactory bulb and hippocampal cells showed the CD44-mediated encocytosis pathway (Figure S5). Although CD44 showed remarkable binding affinity to hyaluronic acid (HA) and CS ligands,^[30]^ in our results, we have shown that CD44-overexpressed cell lines can be targeted by using simple GalNAc(4,6-*O*-disulfate)β(1-3)GlcA disaccharide ligand conjugated nanoprobes more effectively than other sulphated CS-di or HS-tetrasaccharide ligands. Further, we demonstrated that HS ligands are potential negative control ligand, for CD44-mediated nanoparticles uptake studies. Finally, we have demonstrated efficient targeting of primary neural cells using AuF@**CS46S** nanoprobe.

## Conclusions

In summary, we have presented a new set of GAG nanoprobes composed of both HS and CS synthetic ligands that can give a broad picture of the cell-specific homing of nanoparticles and provide a novel opportunity for nanotherapy. More specifically, we have shown that CS-E is a potential ligand for designing nanoprobes to target CD44-overexpressed cancer cells and neurites.

## Experimental Section

### Primary neuronal culture

Wild-type mice (neonatal p0/p1, *N* = 36) were used to optimize and establish primary neuronal cultures of the murine olfactory bulb and hippocampus. All animal care and procedures were in accordance with the Institutional Animal Ethics Committee (IAEC) at IISER Pune and the Committee for the Purpose of Control and Supervision of Experiments on Animals (CPCSEA), Government of India.

### Preparation

The day prior to isolation, appropriately sized tissue culture plasticware was coated with a 1x poly-d-lysine solution for proper cell attachment to the plate. All dissection tools were sterilized prior to use and the tissue harvesting was performed under a stereomicroscope.

### Tissue harvesting

The pup was decapitated by using scissors, the skin and cranium were opened from the back of the neck to the nose in order to completely remove the skull. The entire brain was removed and placed onto a sterile petri dish with HBSS. The olfactory bulb appears as two small lobes on the rostral end of the brain, protruding from the cerebral hemispheres. The OB was dissected from the brain, the tissue was minced and collected in HBSS.

The cerebellum was removed from the caudal side of the brain and the brain was separated into two hemispheres by making an incision down the midline. The midbrain, thalamus, and hypothalamus tissue were removed by placing each of the hemispheres with their medial side facing up, allowing us to visualize the hippocampus and the cortex. The hippocampus is a flat, curved structure that begins in the distal part of the hemisphere and curves towards the ventral side. It is free from attachment towards its concave (caudal) side; the hippocampal tissue was separated by cutting along the convex outer side. Each of the brain hemispheres was utilized to harvest two hippocampi from one individual, and the tissue was minced on a spatula and collected in HBSS.

### Tissue dissociation

The OB and hippocampal tissues were allowed to settle in the HBSS. The supernatant is removed and 1 mL of 0.25% Trypsin is added for dissociation. The tissues were then placed in the incubator at 37°C and 5% CO_2_ for 15 min after trituration. Following the incubation, 3 mL of serum enriched neurobasal complete medium (10%, NB + FBS) at room temperature was added to each of the tubes containing the neuronal tissue. The neuronal cells were triturated and were subjected to centrifugation at 4°C and 300 *g* for 10 min. After separating the supernatant medium, 5 mL of wash buffer (3.3% HEPES + 1% PenStrep in HBSS) was added to the cells, and trituration was performed again. The cells were centrifuged at 4°C, 300 *g* for an additional 10 min before separating the supernatant from the pellet of cells.

### Cell plating

1 mL of NB + FBS medium was added to the cells, and trituration was performed to obtain a more homogeneous cellular suspension. The cells in the culture were counted using a hemocytometer and the cells were plated in the appropriate tissue culture plasticware, roughly 600000–800000 cells mL^−1^ of suspension. Proper recommendations were followed for the optimal cell count and the plating volume for different culture surfaces such as 24 well plates, 8-well plates, 35 mm coverslips, etc. Both the OB and hippocampal cells were plated in NB + FBS medium and placed in the incubator for approximately 8 h to allow for cellular attachment to the tissue culture surface. Following the attachment of cells, the appropriate volume (determined by the dimensions of the well) of NB + FBS medium was added to the primary OB neuronal culture and similarly, serum-free NB complete medium was added to the primary hippocampal neuronal culture as CM. The serum-enriched medium was removed completely after 24 h and replaced with NB complete medium. The primary cell culture was fed and maintained by replacing 50% of the NB medium after every 96 h.

### Secondary cell lines

MDA-MB 231, MDA-MB-468, T-47D, MCF-7, SK-BR-3 and SH-SY5Y cell lines were grown at 37°C in 5% CO_2_ in DMEM medium containing 10% fetal bovine serum and 0.1% streptomycin. U87 Grown at 37°C in 5% CO_2_ in MEM medium containing, 1% nonessential amino acids (NEAA), 2 mM glutamine, 1 mM sodium pyruvate, 10% fetal bovine serum and 0.1% streptomycin.

### Confocal Imaging

2 × 10^4^ cells were seeded 8 well plate in growth medium and incubated at 37°C overnight. The cells were incubated with CS/HS tripod-functionalized AuNPs (5 μg mL^−1^) for different time intervals of 2 and 4 h. Then the cells were washed with the PBS and fluorescent images were taken using Leica sp8 microscope.

### FACS analysis

U87 cells (2 × 10^6^ cells) were seeded in 96 well plates in MEM medium and incubated at 37°C overnight. The cells were pulsed with CS/HS tripod functionalized AuNPs (5 μg mL^−1^) for 4 h. The cells were washed with PBS to remove noninternalized AuNPs, trypsinzed, and finally resuspended in FACS buffer and preceded for analysis. Quantification of uptake was done by using Flowjo software.

### CD44 receptor expression level

All cells (2 × 10^6^ cells) were seeded in 8-well plates in respective growth medium and incubated at 37°C for overnight. The cells were pulsed with FITC-CD44 Monoclonal Antibody (IM7) for 4 h. Cells were then washed and nuclei were stained with Hoechst 33342 and taken for imaging.

### Cellular uptake mechanism studies

To study cellular internalization mechanism, U87 cells were grown in an 8-well chamber cover glass (1 × 10^4^ cells per well) overnight and treated with the specific inhibitor for 30 min followed by treatment with AuF@**CS46S** (5 μg mL^−1^) for 4 h at 37°C. For the energy dependent, dynamin-mediated, clathrin-mediated, caveolae-mediated and CD44 receptor-mediated study, cells were incubated for 30 min with NaN_3_ (50 mM), dynasore hydrate (50 μM), chlorpromazine (25 μM) methylated-β-cyclodextrin (10 mM) and CD44 monoclonal antibody (1 : 1000 dilution), respectively. After 4 h of treatment of AuF@**CS46S**, the cells were washed to remove unbound materials and taken for imaging.

## Supplementary Material

Supplementary Materials

## Data Availability

The data that support the findings of this study are available from the corresponding author upon reasonable request.

## References

[1] de la Fuente JM, Barrientos AG, Rojas TC, Rojo J, Cañada J, Fernández A, Penadés S (2001). Angew Chem Int Ed.

[2] Fasting C, Schalley CA, Weber M, Seitz O, Hecht S, Koksch B, Dernedde J, Graf C, Knapp EW, Haag R (2012). Angew Chem Int.

[3] Ma W, Liu HT, He XP, Zang Y, Li J, Chen GR, Tian H, Long YT (2014). Anal Chem.

[4] Schofield CL, Field RA, Russell DA (2007). Anal Chem.

[5] Budhadev D, Poole E, Nehlmeier I, Liu Y, Hooper J, Kalverda E, Akshath US, Hondow V, Turnbull WB, Pöhlmann S, Guo Y, Zhou D (2020). J Am Chem Soc.

[6] Schofield CL, Marín MJ, Rejzek M, Crocker PR, Field RA, Russell DA (2016). Analyst.

[7] Chaudhary PM, Sangabathuni S, Murthy RV, Paul A, Thulasiram HV, Kikkeri R (2015). Chem Commun.

[8] Arosio D, Chiodo F, Reina JJ, Marelli M, Penadés S, Van Kooyk Y, Garcia-Vallejo JJ, Bernardi A (2014). Bioconjugate Chem.

[9] Marradi M, Chiodo F, García I, Penadés S (2013). Chem Soc Rev.

[10] Chaudhary PM, Toraskar S, Yadav R, Hande A, Yellin RA, Kikkeri R (2019). Chem Asian J.

[11] Wang X, Xie Y, Jiang N, Wang J, Liang H, Liu D, Yang N, Sang X, Feng Y, Chen R, Chen Q (2021). ACS Appl Mater Interfaces.

[12] Samanta S, Joncour VL, Wegrzyniak O, Rangasami VK, Ali-Löytty H, Hong T, Selvaraju Ram K, Aberg O, Hilborn J, Laakkonen P, Varghese OP (2022). Adv Ther.

[13] Hao Y, Gao Y, Wu Y, An C (2019). Int J Mol Med.

[14] Du H, Liu M, Yu A, Ji J, Zhai G (2017). Int J Pharm.

[15] Sun H, Cao D, Liu Y, Wang H, Ke X, Ci T (2018). Biomater Sci.

[16] Zhang Z, Ma L, Luo J (2021). Pharmaceutica.

[17] Li H, Zhang P, Luo J, Hu D, Haung Y, Zhang Z-R, Fu Y, Gong T (2019). ACS Nano.

[18] Domínguez-Rodríguez P, Reina JJ, Gil-Caballero S, Eito PM, De Paz JL, Rojo J (2017). Chem Eur J.

[19] Lindahl U, Kusche-Gullberg M, Kjellen L (1998). J Biol Chem.

[20] Bedini E, Laezza A, Iadonisi A (2016). Eur J Org Chem.

[21] Jain P, Shanthamurthy C, Chaudhary PM, Kikkeri R (2021). Chem Sci.

[22] Mende M, Bednarek C, Wawryszyn M, Sauter P, Biskup MB, Schepers U, Bräse S (2016). Chem Rev.

[23] Jain P, Shanthamurthy C, Leviatan Ben-Arye S, Woods RJ, Kikkeri R, Padler-Karavani V (2021). Chem Sci.

[24] Zhang L, Xu P, Liu B, Yu B (2020). J Org Chem.

[25] Compostella F, Pitirollo O, Silvestri A, Polito L (2017). Beilstein J Org Chem.

[26] Toraskar S, Chaudhary PM, Kikkeri R (2022). ACS Chem Biol.

[27] Mandarini E, Tollapi E, Zanchi M, Depau L, Pini A, Brunetti J, Bracci L, Falciani C (2020). Int J Mol Sci.

[28] Sangabathuni S, Murthy RV, Chaudhary PM, Surve M, Banerjee A, Kikkeri R (2016). Nanoscale.

[29] Konecny GE, Pegram MD, Venkatesan N, Finn R, Yang G, Rahmeh M, Untch M, Rusnak DW, Spehar G, Mullin RJ, Keith B (2006). Cancer Res.

[30] Li M, Sun J, Zhang W, Zhao Y, Zhang S, Zhang S (2021). Carbohydr Polym.

