# Supplementary material for: Sulfation of Heparan and Chondroitin Sulfate Ligands Enables Cell-Specific Homing of Nanoprobes
Source: Chemistry. Author manuscript; Available in PMC 2024 May 27. (PMC7616003; doi:10.1002/chem.202202622)
Supplement: Supplementary Materials [file EMS196094-supplement-Supplementary_Materials.pdf]

Chemistry–A European Journal

Supporting Information

Sulfation of Heparan and Chondroitin Sulfate Ligands Enables Cell-Specific Homing of Nanoprobes

Sandhya Mardhekar, Balamurugan Subramani, Prasanna Samudra, Priyadharshini Srikanth, Virendrasinh Mahida, Preeti Ravindra Bhoge, Suraj Toraskar, Nixon M. Abraham,* and Raghavendra Kikkeri*

Table of contents

1. General information

2. Synthesis of HS tetrasaccharide analogs

3. Synthesis of CS disaccharide analogs

4. Synthesis of tripod GAG derivatives

5. Synthesis of gold nanoparticles

6. Synthesis of GAG-nanoprobes

7. Physical characterization of GAG nanoprobes

8. Reference

9. ^{1}H-NMR, ^{13}C and DEPT-135 NMR

1. **General Information**

All chemicals were reagent grade and used without further purification unless otherwise noted. Reactions were carried out in anhydrous solvents under a nitrogen atmosphere. Reaction progress was monitored by analytical thin-layer chromatography (TLC) on Merck silica gel 60 F_{254}. Spots on the TLC plate were visualized under UV light or dipping the TLC plate in CAM/ninhydrin solution followed by heating. Column chromatography was carried out using Fluka kieselgel 60 (230-400 mesh). ^{1}H and ^{13}C NMR spectra of compounds were measured with Bruker 400 MHz, Bruker 600 MHz and Jeol 400 MHz using residual solvents as an internal reference ($CDCl_3$ δH 7.26 ppm, δC 77.3 ppm, CD_3OD δH 3.31 ppm, δC 49.0 ppm, and D_2O δH 4.79 ppm). The chemical shifts (δ) are reported in ppm and coupling constants (J) in Hz. UV-visible measurements were performed with Evolution 300 UV-visible spectrophotometer (Thermo Fisher Scientific, USA). Fluorescence spectra were measured with FluoroMax-4 spectrofluorometer (Horiba Scientific, U.S.A.). CD44 (8E2) Mouse mAb and CD44 Monoclonal Antibody (IM7), FITC used for inhibition study and cell level expression study were purchased from cell signalling technology and biosciences respectively. All microscopy images were captured using Leica SP8 confocal microscope and processed using Image J software.

2. **Synthesis of HS tetrasaccharide analogs.**

Compound **1-3**, **T-1 and F-1** was prepared following a literature protocol.[1,2]

General Experimental Procedures

General procedure A. To a solution of TBDPS protected HS oligosaccharides (1 mmol) in pyridine (5 ml) was added 70 % HF.py (5 mmol) at 0 °C and stirred at room temperature for 12 h. After completion, the reaction mixture was diluted with EtOAc and washed with 1N HCl and brine. The organic layer was dried using anhydrous Na_2SO_4 and concentrated under reduced pressure. The residue of non sulfated sugars was purified by silica column chromatography in EtOAc/Hexane eluent and for sulfated sugars sephadex LH-20 column with MeOH eluent.

General procedure B. To a solution of NAP-protected HS oligosaccharides (1 mmol) in DCM/H_2O (18:1 v/v, 10 mL) was added DDQ (5 mmol) and stirred under nitrogen atmosphere at 0 °C for 3-4 h. After the reaction completion, the mixture was diluted with DCM and washed with brine. The organic layer was concentrated under reduced pressure and the residue was purified by silica gel column chromatography (EtOAc/Hexane). (DDQ is 2,3-Dichloro-5,6-dicyano-1,4-benzoquinone)

General procedure C. To a solution of 6-*O* or 3-*O*-hydroxyl group HS oligosaccharides (1 mmol) in DMF (5 mL) was added SO_3·NMe_3 (10 mmol per -OH) and microwaved at 100 °C, 15 min using a CEM Initiator synthesizer in sealed reaction vessels (average power 15 w). DMF was evaporated and the crude product was purified by silica column chromatography (MeOH/DCM).

General procedure D. To a solution of HS oligosaccharides (1 mmol) in a mixture of THF/H_2O (1:1 v/v, 4 ml) was added 1 M LiOH·H_2O (3 mmol) and stirred at RT for 15 h. After completion of the reaction, the reaction mixture was diluted with MeOH and neutralized using Dowex 50WX8 H^+ resin. The reaction was filtered and concentrated under reduced pressure and purified by bond elute C-18 column (H_2O/ACN).

General procedure E. To a solution of HS oligosaccharides (1 mmol) in H_2O (4 ml) was added $Pd(OH)_2$ (10% per wt) and stirred for 24 h under ahydrogen atmosphere. After reaction completion, the reaction mixture was diluted with H_2O, filtered and concentrated, and finally purified by bond elute C-18 column (H_2O/ACN).

Synthesis of Compound 4. To a solution of **2** (2.0 g, 3.59 mmol) and *N*-benzyloxycarbonyl-3-aminopropanol (0.90 g, 4.30 mmol) in anhydrous DCM (20 mL) under argon was added pre-activated 4 Å molecular sieves (1.0 g) and stirred at room temperature for 1 h. The reaction was cooled to -20 ^{o}C and mixed with NIS (0.88 g, 3.95 mmol) and TMSOTf (0.13 mL, 0.58 mmol). After completion of the reaction, the molecular sieves were filtered and organic layer was washed with sodium thiosulphate and brine and concentrated under reduced pressure. Purification was done by silica gel column chromatography (EtOAc/Hexane = 1/7, v/v) yielded 87% of compound **4**. ^{1}H NMR (400 MHz, $CDCl_3$) δ 8.02 (d, *J* = 7.4 Hz, 2H), 7.56 (t, *J* = 7.4 Hz, 1H), 7.42 (t, *J* = 7.7 Hz, 2H), 7.37 – 7.27 (m, 5H), 7.23 – 7.16 (m, 5H), 5.26 – 5.19 (m, 1H), 5.03 (s, 2H), 4.73 (d, *J* = 9.4 Hz, 1H), 4.60 (d, *J* = 9.4 Hz, 1H), 4.53 (d, *J* = 8.0 Hz, 1H), 4.46 (d, *J* = 4.6 Hz, 1H), 4.06 (s, 1H), 3.86 (m, 1H), 3.71 – 3.60 (m, 2H), 3.58 – 3.49 (m, 2H), 3.13 (m, 2H), 2.56 (d, *J* = 2.6 Hz, 1H), 1.70 (m, 2H). ^{13}C NMR (100 MHz, $CDCl_3$) δ 167.73, 165.32, 156.52, 137.68, 133.50, 129.83, 129.61, 128.68, 128.65, 128.58, 128.15, 101.22, 82.08, 74.82, 73.49, 69.89, 67.47, 66.53, 64.58, 40.81, 38.10, 29.51. HR-ESI-MS (*m/z*): $[M+Na]^+$, calculated for $C_{33}H_{36}ClNO_{10}Na$, 664.1925; found, 664.1933.

Synthesis of Compound 5. To a solution of **1** (1.80 g, 2.20 mmol) and **2** (0.98 g, 1.76 mmol) in anhydrous DCM (20 mL) was added pre-activated 4 Å molecular sieves (0.9 g) and stirred at RT for 1 h under nitrogen atmosphere. Then at -40 °C, AgOTf (3.96 g, 15.43 mmol) was added and stirred for 40 min. After the completion of reaction molecular sieves filtered and organic layer was washed with saturated brine solution and concentrated under reduced pressure. Purification was done by silica gel column chromatography (EtOAc/Hexane = 1/8, v/v), yielded 60% of **5**. ^{1}H NMR (400 MHz, $CDCl_3$) δ 8.13 (d, *J* = 8.2 Hz, 2H), 7.89 – 7.79 (m, 4H), 7.64 (dd, *J* = 18.5, 7.5 Hz, 5H), 7.52 (q, *J* = 7.5, 6.4 Hz, 5H), 7.47 – 7.40 (m, 2H), 7.40 – 7.30 (m, 9H), 7.30 – 7.18 (m, 8H), 7.12 (d, *J* = 7.3 Hz, 2H), 5.58 – 5.53 (m, 1H), 5.30 (t, *J* = 9.1 Hz, 1H), 5.07 (q, *J* = 10.8 Hz, 2H), 4.95 – 4.68 (m, 5H), 4.61 (m, 1H), 4.20 (m,1H), 4.01 (m, 3H), 3.94 – 3.65 (m, 6H), 3.62 (d, *J* = 9.5 Hz, 1H), 3.34 (dd, *J* = 10.2, 2.4 Hz, 1H), 2.36 (s, 3H), 1.07 (s, 9H). ^{13}C NMR (100 MHz, $CDCl_3$) δ 137.83, 135.91, 135.63, 135.19, 133.93, 133.46, 129.89, 129.81, 129.78, 129.56, 128.59, 128.53, 128.42, 128.32, 128.00, 127.78, 127.70, 127.64, 126.97, 126.11, 126.02, 98.03, 85.87, 84.59, 79.87, 76.22, 75.64, 75.23, 74.71, 73.67, 73.06, 72.63, 64.48, 63.33, 62.04, 40.50, 29.72, 26.91, 21.19, 19.33. HR-ESI-MS (*m/z*): $[M+Na]^+$, calculated for $C_{69}H_{70}ClN_3O_{11}SSiNa$, 1234.4087; found, 1234.4086.

Synthesis of Compound 6. To a solution of **3** (1.8 g, 2.28 mmol) and **4** (1.17 g, 1.48 mmol) in anhydrous DCM (20 mL) was added preactivated 4 Å molecular sieves and stirred at room temperature for 1 h. Then at -20 °C, NIS (0.10 g, 0.46 mmol) and TMSOTf (0.082 mL, 0.37 mmol) were rapidly added and stirred at the same temperature for 10 min. After the completion of the reaction molecular sieves were filtered, dissolved in DCM and washed with sodium thiosulphate and brine and concentrated under reduced pressure, purified by silica gel column chromatography (EtOAc/Hexane= 1/7, v/v) yielded 78% of compound **6**. ^{1}H NMR (400 MHz, $CDCl_3$) δ 8.03 (d, *J* = 7.3 Hz, 2H), 7.85 – 7.79 (m, 4H), 7.62 (m, 4H), 7.56 (t, *J* = 7.4 Hz, 1H), 7.48 – 7.37 (m, 7H), 7.36 (s, 1H), 7.33 (d, *J* = 4.5 Hz, 7H), 7.31 (s, 1H), 7.20 (d, *J* = 2.0 Hz, 2H), 7.17 (m, 2H), 5.58 (s, 1H), 5.29 (s, 1H), 5.23 (d, *J* = 10.5 Hz, 1H), 5.05 (s, 2H), 4.93 (d, *J* = 4.7 Hz, 1H), 4.83 (d, *J* = 11.4 Hz, 1H), 4.79 – 4.70 (m, 2H), 4.62 (m, 1H), 4.56 (d, *J* = 7.7 Hz, 1H), 4.17 (s, 1H), 4.02 (s, 2H), 3.91 (d, *J* = 2.3 Hz, 10H), 3.34 (m, 1H), 3.15 (m, 2H), 2.52 – 2.46 (m, 2H), 2.36 (s, 2H), 2.05 (s, 3H), 1.70 (q, *J* = 6.3 Hz, 2H), 1.01 (s, 9H). ^{13}C NMR (100 MHz, $CDCl_3$) δ 205.98, 171.17, 165.19, 156.45, 136.78, 135.72, 135.12, 133.49, 133.29, 133.14, 133.05, 129.75, 129.72, 128.60, 128.49, 128.43, 128.19, 128.03, 127.76, 127.64, 126.80, 126.10, 125.99, 125.94, 100.72, 97.48, 97.43, 82.93, 77.74,

74.75, 74.23, 73.74, 73.47, 73.34, 72.64, 72.53, 71.75, 70.17, 67.28, 67.14, 66.46, 64.62, 64.35, 62.90, 62.83, 62.18, 62.09, 40.56, 38.05, 37.66, 29.71, 29.69, 29.49, 27.91, 26.80, 19.29, 14.15. HR-ESI-MS (*m/z*): $[M+Na]^+$, calculated for $C_{71}H_{77}ClN_4O_{16}SiNa$, 1327.4690; found, 1327.4694.

Synthesis of Compound 7. To a solution of **6** (1.50 g, 1.14 mmol) in DCM (15 mL) under nitrogen was added glacial acetic acid (0.33 mL, 5.47 mmol) and $N_2H_4{\cdot}H_2O$ (0.18 mL, 5.58 mmol) and stirred for 12 h at RT. The reaction mixture was extracted with DCM and washed with brine. Organic layer was concentrated and purified using column chromatography (EtOAc/Hexane = 1/6, v/v) yielded 70% of **7**. ^{1}H NMR (400 MHz, $CDCl_3$) δ 8.03 (d, *J* = 7.2 Hz, 2H), 7.88 – 7.82 (m, 4H), 7.68 – 7.62 (m, 4H), 7.56 – 7.53 (m, 2H), 7.50 – 7.47 (m, 2H), 7.45 – 7.40 (m, 4H), 7.40 – 7.32 (m, 9H), 7.23 – 7.15 (m, 5H), 5.54 (d, *J* = 3.9 Hz, 1H), 5.31 – 5.25 (m, 1H), 5.05 (s, 5H), 4.78 (d, *J* = 10.4 Hz, 1H), 4.71 (d, *J* = 10.4 Hz, 1H), 4.62 (d, *J* = 11.5 Hz, 1H), 4.56 (d, *J* = 7.7 Hz, 1H), 4.18 (m, 1H), 3.99 (t, *J* = 8.6 Hz, 1H), 3.93 – 3.80 (m, 7H), 3.76 (m, 1H), 3.67 (m, 1H), 3.62 (dd, *J* = 8.6, 4.1 Hz, 1H), 3.57 – 3.50 (m, 1H), 3.27 – 3.06 (m, 3H), 2.65 (s, 1H), 1.70 (m, 2H), 1.05 (s, 9H). ^{13}C NMR (100 MHz, $CDCl_3$) δ 165.17, 156.46, 136.80, 135.68, 135.59, 135.48, 133.46, 133.36, 133.13, 130.02, 129.99, 129.74, 129.47, 128.59, 128.49, 128.40, 128.37, 128.06, 128.02, 127.89, 127.83, 127.77, 127.72, 126.94, 126.17, 126.05, 126.00, 100.67, 97.85, 82.99, 79.36, 75.28, 74.34, 73.84, 73.60, 72.57, 72.32, 72.15, 67.31, 64.43, 63.83, 62.59, 40.53, 38.09, 29.48, 26.92, 19.27. HR-ESI-MS (m/z): $[M+Na]^+$, calculated for $C_{66}H_{71}ClN_4O_{14}SiNa$, 1229.4322; found 1229.4324.

Synthesis of Compound 8. To a solution of **5** (1.50 g, 1.24 mmol), **7** (1.24 g, 1.02 mmol) in anhydrous DCM (20 mL) at -20 ^{o}C was added NIS (0.33 g, 1.48 mmol) and TMSOTf (0.02 mL, 0.13 mmol). Once reaction completed, molecular sieves were separated using celite filtration and organic layer was washed with sodium thiosulphate and brine. Finally, the organic layer was dried using anhydrous Na_2SO_4, concentrated, purified through silica gel column chromatography (EtOAc/Hexane = 1/7, v/v) yielded 72% of compound **8**. ^{1}H NMR (400 MHz, $CDCl_3$) δ 7.99 (d, *J* = 7.2 Hz, 2H), 7.86 – 7.76 (m, 8H), 7.76 – 7.70 (m, 4H), 7.66 – 7.60 (m, 6H), 7.56 – 7.49 (m, 6H), 7.49 – 7.44 (m, 4H), 7.42 (dd, *J* = 3.3, 1.5 Hz, 2H), 7.40 – 7.36 (m, 6H), 7.36 – 7.32 (m, 5H), 7.32 – 7.27 (m, 10H), 7.22 (t, *J* = 2.0 Hz, 2H), 7.20 – 7.15 (m, 7H), 7.14 – 7.09 (m, 2H), 5.55 (d, *J* = 3.8 Hz, 1H), 5.46 (d, *J* = 4.0 Hz, 1H), 5.38 (t, *J* = 8.7 Hz, 1H), 5.27 (d, *J* = 11.6 Hz, 1H), 5.21 – 5.15 (m, 1H), 5.08 (m, 5H), 4.90 – 4.80 (m, 4H), 4.74 (m, 2H), 4.61 (m, 2H), 4.43 (d, *J* = 7.8 Hz, 1H), 4.33 (d, *J* = 2.3 Hz, 2H), 4.18 (t, *J* = 9.3 Hz, 1H), 4.13 – 4.07 (m, 1H), 4.05 – 3.86 (m, 4H), 3.85 – 3.77 (m, 5H), 3.72 – 3.59 (m,

4H), 3.53 (d, J = 9.5 Hz, 2H), 3.50 – 3.44 (m, 2H), 3.41 (dd, J = 7.7, 4.7 Hz, 1H), 3.37 (s, 2H), 3.30 (d, J = 3.8 Hz, 1H), 3.29 – 3.25 (m, 1H), 3.25 – 3.18 (m, 2H), 3.14 (m, 1H), 3.10 – 3.02 (m, 1H), 1.66 (dt, J = 12.7, 7.1 Hz, 2H), 1.08 (s, 8H), 1.00 (s, 8H). ^{13}C NMR (100 MHz, $CDCl_3$) δ 165.18, 156.45, 137.33, 135.95, 135.91, 135.86, 135.70, 135.62, 133.69, 133.44, 133.24, 132.88, 130.28, 130.10, 129.76, 129.71, 129.43, 128.81, 128.66, 128.57, 128.53, 128.47, 128.37, 128.34, 128.14, 128.03, 128.00, 127.97, 127.90, 127.86, 127.78, 127.77, 127.74, 127.70, 127.59, 126.95, 126.60, 126.14, 126.03, 126.00, 125.96, 125.75, 100.56, 99.98, 98.19, 97.75, 83.28, 82.97, 79.49, 76.30, 75.61, 75.21, 75.01, 74.78, 74.65, 74.21, 74.11, 73.83, 73.27, 73.05, 72.48, 72.17, 72.08, 67.23, 64.55, 63.26, 62.99, 62.53, 61.96, 60.84, 40.17, 29.71, 29.44, 26.98, 26.89, 19.39, 19.30. HR-ESI-MS (*m/z*): $[M+Na]^+$, calculated for $C_{128}H_{133}Cl_2N_7O_{25}Si_2Na$: 2316.8164; found 2316.8170.

Synthesis of Compound 9. Deprotection of chloroacetate: To a solution of **8** (1.30 g, 0.56 mmol) in pyridine/MeOH (1:1 v/v, 15 ml) at 80 °C was added thiourea (0.16 g, 2.21 mmol) and stirred under nitrogen atmosphere for 6 h. After completion of the reaction, the solvent was evaporated, dissolved in EtOAc and washed with 1N HCl and brine solution. The organic layer was dried using anhydrous Na_2SO_4, filtered, concentrated and purified through silica gel column chromatography (EtOAc/Hexane = 1/6, v/v), yielded 95% of **de-chloroacetate HS tetrasaccharide precursor.** . ^{1}H NMR (400 MHz, $CDCl_3$) δ 8.02 (d, J = 7.5 Hz, 2H), 7.84 – 7.73 (m, 10H), 7.73 – 7.67 (m, 3H), 7.67 – 7.60 (m, 4H), 7.59 – 7.52 (m, 3H), 7.52 – 7.44 (m, 8H), 7.44 – 7.34 (m, 10H), 7.32 (d, J = 5.5 Hz, 6H), 7.30 – 7.28 (m, 4H), 7.26 (s, 1H), 7.24 (s, 1H), 7.22 (d, J = 4.1 Hz, 4H), 7.19 (s, 1H), 7.17 (m, 7H), 5.59 (d, J = 3.9 Hz, 1H), 5.50 (d, J = 3.7 Hz, 1H), 5.38 (t, J = 8.6 Hz, 1H), 5.27 – 5.18 (m, 2H), 5.08 – 5.01 (m, 5H), 4.95 – 4.73 (m, 6H), 4.65 (m, 2H), 4.50 (d, J = 7.8 Hz, 1H), 4.18 – 4.08 (m, 2H), 4.02 – 3.70 (m, 13H), 3.58 – 3.44 (m, 4H), 3.40 – 3.24 (m, 6H), 3.23 – 3.08 (m, 3H), 1.68 (m, 2H), 1.06 (s, 9H), 1.04 (s, 9H). ^{13}C NMR (100 MHz, $CDCl_3$) δ 165.26, 165.09, 156.47, 138.08, 137.48, 136.73, 135.99, 135.92, 135.72, 135.68, 135.60, 135.37, 133.82, 133.55, 133.43, 133.37, 133.27, 133.13, 132.95, 132.43, 130.16, 129.96, 129.88, 129.79, 129.75, 129.59, 128.64, 128.60, 128.53, 128.48 (d, J = 1.9 Hz), 128.41, 128.33, 128.26, 128.09, 128.03, 127.86, 127.79, 127.72, 126.95, 126.52, 126.13, 126.08, 126.06, 126.02, 125.81, 101.00, 100.06, 97.92, 97.36, 83.63, 83.32, 79.85, 78.07, 77.78, 76.07, 75.66, 75.58, 75.14, 74.87, 74.55, 74.39, 74.00, 73.19, 72.70, 72.16, 72.05, 67.46, 66.50, 63.41, 62.97, 62.06, 61.77, 61.35, 60.89, 38.11, 29.52, 27.03, 26.90. HR-ESI-MS (*m/z*): $[M+H]^+$, calculated for $C_{124}H_{131}N_7O_{23}Si_2$, 2142.8508; found, 2142.8509.

Oxidation and esterification. To a solution of **de-chloroacetate HS tetrasaccharide precursor** (1.10 g, 0.52 mmol) in DCM/H_2O (2:1, 12 mL) was added 2,2,6,6-tetramethyl-1-piperidinyloxyl free radical (TEMPO) (0.06 g, 0.41 mmol), and [Bis(acetoxy)iodo] benzene (BAIB) (1.67 g, 5.2 mmol) and stirred at RT for 6 h. The organic layer was washed with saturated NH_4Cl solution and concentrated. Then, the crude product was dissolved in DMF (10 mL) and mixed with K_2CO_3 (0.21 g, 0.001 mmol), MeI (0.09 mL, 0.001 mmol), and stirred overnight at RT. On completion of reaction, reaction mixture extracted with EtOAc and brine. The organic layer was concentrated and purified through silica gel column chromatography (EtOAc/Hexane = 1/8, v/v) to get compound **oxidized and esterified HS precursor** in 62% yield. ^{1}H NMR (400 MHz, $CDCl_3$) δ 8.01 (d, *J* = 7.3 Hz, 2H), 7.86 – 7.73 (m, 13H), 7.70 – 7.62 (m, 7H), 7.60 – 7.37 (m, 19H), 7.35 – 7.27 (m, 18H), 7.18 (m, 14H), 5.49 (d, *J* = 3.7 Hz, 1H), 5.47 – 5.41 (m, 2H), 5.35 – 5.26 (m, 2H), 5.08 (d, *J* = 3.5 Hz, 2H), 5.04 (s, 3H), 4.99 (t, *J* = 6.0 Hz, 1H), 4.97 – 4.91 (m, 2H), 4.84 (m, 2H), 4.76 (m, 2H), 4.68 (d, *J* = 10.4 Hz, 1H), 4.57 – 4.51 (m, 2H), 4.27 – 3.87 (m, 12H), 3.85 – 3.67 (m, 6H), 3.44 (m, 2H), 3.33 (m, 1H), 3.23 (d, *J* = 3.9 Hz, 1H), 3.20 (s, 3H), 3.09 (m, 4H), 2.89 (s, 3H), 1.66 (m, 2H). ^{13}C NMR (100 MHz,$CDCl_3$) δ 168.36, 167.66, 165.14, 164.68, 156.41, 138.36, 137.27, 137.20, 136.85, 135.94, 135.90, 135.70, 135.63, 135.60, 135.33, 133.92, 133.59, 133.53, 133.50, 133.39, 133.35, 133.12, 133.03, 132.93, 132.29, 130.21, 129.99, 129.75, 129.69, 129.61, 129.37, 128.99, 128.69, 128.60, 128.47, 128.44, 128.40, 128.39, 128.30, 128.22, 128.17, 128.02, 128.00, 127.98, 127.87, 127.83, 127.74, 127.70, 127.65, 127.52, 127.48, 127.35, 126.97, 126.80, 126.11, 126.07, 126.00, 125.64, 125.58, 100.84, 100.06, 98.03, 97.26, 82.94, 82.46, 79.73, 77.84, 77.26, 76.24, 75.76, 75.59, 75.04, 74.91, 74.88, 74.68, 74.28, 73.91, 73.65, 73.52, 73.48, 72.39, 71.54, 67.31, 66.37, 63.49, 62.61, 61.80, 60.50, 52.33, 51.77, 38.02, 29.72, 29.34, 19.42, 19.40. HR-ESI-MS (*m/z*): $[M+2H]^+$, calculated for $C_{126}H_{131}N_7O_{25}Si_2$, 2199.8890; found, 2199.8898.

Azide to *N*-acetate conversion. O**xidized and esterified HS precursor** (0.9 g, 0.40 mmol) and zinc dust (1.07 g, 0.01 mmol) was dissolved in mixture of THF/AcOH/Ac_2O (3:2:1, 12 ml) and stirred at RT for 12 h. Then solvent evaporated, and the residue was coevaporated with MeOH, Further residue was dissolved in EtOAc and washed with saturated $NaHCO_3$ and brine. Crude obtained on concentration of EtOAc layer was purified by column chromatography (EtOAc/Hexane= 1/6, v/v) to afford 79% of **9**. ^{1}H NMR (400 MHz, $CDCl_3$) δ 8.02 (d, *J* = 7.6 Hz, 2H), 7.83 (d, *J* = 7.5 Hz, 2H), 7.71 – 7.65 (m, 7H), 7.61 (d, *J* = 6.7 Hz,

2H), 7.55 (t, *J* = 7.2 Hz, 2H), 7.41 (m, 11H), 7.32 (dd, *J* = 7.1, 2.7 Hz, 14H), 7.22 (dt, *J* = 5.3, 2.4 Hz, 6H), 7.17 (d, *J* = 2.8 Hz, 1H), 7.14 (dd, *J* = 5.9, 3.2 Hz, 3H), 6.39 (d, *J* = 8.0 Hz, 1H), 5.92 (m, 1H), 5.45 – 5.41 (m, 1H), 5.29 (t, *J* = 7.8 Hz, 1H), 5.05 (d, *J* = 8.2 Hz, 4H), 4.97 (d, *J* = 3.2 Hz, 2H), 4.81 (d, *J* = 7.9 Hz, 1H), 4.76 (m, 1H), 4.68 (d, *J* = 10.4 Hz, 1H), 4.61 (m, 3H), 4.54 (d, *J* = 7.0 Hz, 1H), 4.08 (t, *J* = 8.6 Hz, 4H), 4.00 (m, 1H), 3.95 (d, *J* = 9.4 Hz, 1H), 3.87 (m, 3H), 3.80 (m, 4H), 3.74 – 3.61 (m, 6H), 3.44 (s, 3H), 3.32 (d, *J* = 9.8 Hz, 1H), 3.28 (s, 3H), 3.16 – 3.09 (m, 2H), 3.06 (s, 1H), 2.95 (d, *J* = 3.6 Hz, 1H), 1.66 (d, *J* = 5.8 Hz, 2H), 1.50 (s, 3H), 1.36 (s, 3H), 1.09 (d, *J* = 3.8 Hz, 18H). ^{13}C NMR (100 MHz, $CDCl_3$) δ 172.85, 170.97, 168.32, 167.75, 165.53, 165.26, 156.84, 139.03, 136.78, 136.33, 136.29, 136.12, 136.09, 134.30, 134.26, 134.06, 133.83, 133.47, 133.03, 130.46, 130.42, 130.24, 130.20, 130.14, 130.02, 129.64, 129.30, 129.22, 129.17, 129.10, 129.08, 129.02, 128.95, 128.90, 128.42, 128.36, 128.21, 128.14, 101.55, 100.82, 100.22, 99.30, 81.94, 81.59, 78.58, 78.52, 76.68, 75.86, 75.51, 75.37, 75.27, 74.93, 74.06, 73.51, 73.11, 71.96, 70.17, 67.77, 66.85, 62.80, 61.72, 55.19, 53.67, 53.47, 52.92, 38.36, 30.15, 29.79, 27.48, 27.40, 23.11, 22.58, 19.99, 19.83. HR-ESI-MS (*m/z*): $[M+2H]^+$, calculated for $C_{130}H_{139}N_3O_{27}Si_2$: 2231.9291; found, 2231.9293.

Synthesis of Compound 10. 9 (0.22 mmol) was subjected to general procedure A to accomplish as TBDPS deprotection to yield 75 % of **10**. ^{1}H NMR (400 MHz, $CDCl_3$) δ 8.04 (d, *J* = 7.5 Hz, 2H), 7.96 (d, *J* = 7.5 Hz, 2H), 7.82 (d, *J* = 8.9 Hz, 2H), 7.79 (s, 2H), 7.75 (t, *J* = 5.2 Hz, 3H), 7.70 (s, 1H), 7.60 (t, *J* = 7.4 Hz, 1H), 7.53 (d, *J* = 7.3 Hz, 1H), 7.50 – 7.44 (m, 5H), 7.44 – 7.39 (m, 5H), 7.37 (s, 1H), 7.33 (d, *J* = 3.2 Hz, 9H), 7.05 (m, 10H), 5.67 (m, 1H), 5.51 – 5.43 (m, 2H), 5.24 (t, *J* = 7.2 Hz, 1H), 5.14 – 5.07 (m, 2H), 5.06 – 4.92 (m, 6H), 4.86 (m, 1H), 4.76 (m, 1H), 4.71 – 4.66 (m, 2H), 4.57 (m, 3H), 4.42 (m, 2H), 4.27 (m, 1H), 4.19 – 4.13 (m, 2H), 4.09 (m, 1H), 4.04 (m, 1H), 3.92 (m, 1H), 3.88 – 3.81 (m, 3H), 3.79 (d, *J* = 6.5 Hz, 1H), 3.76 (s, 1H), 3.69 (m, 3H), 3.65 – 3.61 (m, 2H), 3.55 (m, 2H), 3.46 (d, *J* = 2.8 Hz, 6H), 3.29 (m, 1H), 3.18 – 3.09 (m, 2H), 2.12 (t, *J* = 6.2 Hz, 1H), 2.04 (s, 1H), 1.68 (d, *J* = 5.7 Hz, 2H), 1.42 (s, 3H), 1.40 (s, 3H). ^{13}C NMR (100 MHz, $CDCl_3$) δ 170.25, 170.06, 168.43, 167.94, 165.02, 164.96, 156.55, 137.87, 136.73, 136.42, 136.38, 136.36, 135.71, 133.84, 133.61, 133.32, 133.22, 132.91, 132.82, 129.69, 129.65, 129.13, 128.96, 128.85, 128.61, 128.58, 128.54, 128.49, 128.27, 128.21, 128.01, 127.99, 127.93, 127.66, 127.63, 126.53, 126.36, 126.25, 126.10, 126.04, 126.01, 125.89, 125.81, 100.73, 100.69, 98.97, 98.35, 81.19, 81.14, 80.11, 77.84, 77.28, 77.25, 75.69, 75.51, 75.17, 75.04, 74.82, 74.62, 74.58, 74.19, 73.68, 73.43, 73.35, 72.23, 67.44, 66.47, 61.62, 60.04, 52.80, 52.61, 52.57, 52.08, 38.18,

29.25, 22.81, 22.68. HR-ESI-MS (*m/z*): $[M+2H]^{2+}$, calculated for $C_{98}H_{103}N_3O_{27}$, 1755.6935; found, 1755.6936.

Synthesis of Compound 11. **9** (0.22 mmol) was subjected to general procedure B to accomplished selective NAP deprotection to yield 70% of **11**. ^{1}H NMR (400 MHz, $CDCl_3$) δ 8.02 (d, *J* = 7.6 Hz, 2H), 7.83 (d, *J* = 7.5 Hz, 2H), 7.73 – 7.64 (m, 6H), 7.61 (d, *J* = 6.7 Hz, 2H), 7.55 (t, *J* = 7.2 Hz, 2H), 7.41 (m, 11H), 7.32 (dd, *J* = 7.1, 2.7 Hz, 14H), 7.22 (dt, *J* = 5.3, 2.4 Hz, 6H), 7.17 (d, *J* = 2.8 Hz, 1H), 7.14 (dd, *J* = 5.9, 3.2 Hz, 3H), 6.39 (d, *J* = 8.8 Hz, 1H), 5.92 (m, 1H), 5.48 – 5.38 (m, 1H), 5.29 (t, *J* = 7.8 Hz, 1H), 5.05 (d, *J* = 8.2 Hz, 4H), 4.97 (d, *J* = 3.2 Hz, 2H), 4.81 (d, *J* = 7.9 Hz, 1H), 4.76 (m, 1H), 4.68 (m, 1H), 4.61 (m, 3H), 4.54 (d, *J* = 7.0 Hz, 1H), 4.08 (t, *J* = 8.6 Hz, 4H), 4.00 (m, 1H), 3.95 (m, 1H), 3.87 (m, 3H), 3.80 (m, 4H), 3.74 – 3.61 (m, 6H), 3.44 (s, 3H), 3.32 (m, 1H), 3.28 (s, 3H), 3.16 – 3.09 (m, 2H), 3.06 (s, 1H), 2.95 (d, *J* = 3.6 Hz, 1H), 1.66 (d, *J* = 5.8 Hz, 2H), 1.50 (s, 3H), 1.36 (s, 3H), 1.09 (d, *J* = 3.8 Hz, 18H). ^{13}C NMR (100 MHz, $CDCl_3$) δ 172.85, 170.97, 168.32, 167.75, 165.53, 165.26, 156.84, 139.03, 136.78, 136.33, 136.29, 136.12, 136.09, 134.30, 134.26, 134.06, 133.83, 133.47, 133.03, 130.46, 130.42, 130.24, 130.20, 130.14, 130.02, 129.64, 129.30, 129.22, 129.17, 129.10, 129.08, 129.02, 128.95, 128.90, 128.42, 128.36, 128.21, 128.14, 101.55, 100.82, 100.22, 99.30, 81.94, 81.59, 78.58, 78.52, 76.68, 75.86, 75.51, 75.37, 75.27, 74.93, 74.06, 73.51, 73.11, 71.96, 70.17, 67.77, 66.85, 62.80, 61.72, 55.19, 53.67, 53.47, 52.92, 38.36, 30.15, 29.79, 27.48, 27.40, 23.11, 22.58. HR-ESI-MS (*m/z*): $[M+2H]^{2+}$, calculated for $C_{108}H_{125}N_3O_{27}Si_2$, 1951.8039; found, 1951.8036.

Synthesis of 12. **10** (0.17 mmol) was subjected to general procedure C to accomplish 6-O-sulfation to yield 76% of **12**. ^{1}H NMR (400 MHz, Methanol-d_4) δ 8.27 (d, *J* = 7.5 Hz, 2H), 7.99 (d, *J* = 7.7 Hz, 2H), 7.90 (d, *J* = 6.0 Hz, 1H), 7.83 – 7.79 (m, 4H), 7.77 (d, *J* = 6.6 Hz, 2H), 7.71 (s, 1H), 7.64 (t, *J* = 7.3 Hz, 1H), 7.56 (m, 3H), 7.48 – 7.37 (m, 8H), 7.31 (s, 7H), 7.26 (d, *J* = 7.5 Hz, 3H), 7.10 – 7.08 (m, 3H), 7.07 – 7.04 (m, 3H), 6.99 – 6.94 (m, 4H), 5.39 – 5.30 (m, 4H), 5.25 (m, 1H), 5.12 (m, 1H), 5.00 (s, 2H), 4.97 (s, 1H), 4.79 (m, 1H), 4.70 (m, 2H), 4.60 (d, *J* = 3.8 Hz, 2H), 4.50 (m, 3H), 4.39 (m, 1H), 4.34 – 4.21 (m, 5H), 4.15 (m, 1H), 4.12 – 3.97 (m, 5H), 3.78 (m, 2H), 3.70 (m, 1H), 3.67 – 3.59 (m, 5H), 3.47 (s, 4H), 3.37 (m, 1H), 3.17 (s, 3H), 3.01 (m, 2H), 1.83 (s, 3H), 1.80 (s, 3H), 1.63 – 1.56 (m, 2H). ^{13}C NMR (100 MHz, Methanol-d_4) δ 172.69, 172.63, 170.29, 169.10, 165.37, 165.07, 157.31, 148.07,

138.61, 137.87, 137.31, 137.30, 137.03, 133.40, 133.25, 129.48, 129.45, 129.38, 129.34, 128.50, 128.31, 128.07, 127.84, 127.81, 127.74, 127.61, 127.55, 127.49, 127.33, 127.30, 127.19, 124.46, 100.77, 100.15, 96.93, 96.87, 82.15, 81.79, 78.08, 77.71, 76.02, 74.95, 74.82, 74.58, 74.51, 74.42, 74.39, 73.98, 73.55, 73.38, 72.66, 72.49, 67.03, 65.88, 60.34, 58.81, 53.68, 53.27, 52.67, 51.94, 37.17, 29.36, 21.66, 21.59. HR-ESI-MS (*m/z*): $[M]^+$, calculated for $C_{98}H_{101}N_3O_{33}S_2^{2-}$, 956.2902; found, 956.2906.

Synthesis of 13. 11 (0.15 mmol) was subjected to general procedure C to accomplish 3-O-sulfation to yield 69% of **13**. ^{1}H NMR (400 MHz, $CDCl_3$) δ 8.07 (d, *J* = 7.8 Hz, 2H), 7.89 (d, *J* = 7.5 Hz, 2H), 7.81 (d, *J* = 3.8 Hz, 2H), 7.74 (d, *J* = 6.9 Hz, 2H), 7.72 (s, 1H), 7.70 (s, 2H), 7.69 (s, 1H), 7.63 (d, *J* = 8.8 Hz, 2H), 7.57 (d, *J* = 4.1 Hz, 3H), 7.50 (s, 2H), 7.49 – 7.46 (m, 3H), 7.44 (d, *J* = 6.5 Hz, 3H), 7.40 (d, *J* = 7.6 Hz, 4H), 7.36 (d, *J* = 5.7 Hz, 5H), 7.36 – 7.27 (m, 7H), 7.23 – 7.20 (m, 2H), 7.15 (dd, *J* = 5.9, 2.4 Hz, 7H), 5.69 – 5.66 (m, 1H), 5.55 (d, *J* = 3.3 Hz, 1H), 5.43 (m, 2H), 5.18 (m, 1H), 5.04 (s, 2H), 4.98 – 4.92 (m, 1H), 4.83 (m, 1H), 4.78 (d, *J* = 7.4 Hz, 1H), 4.71 (d, *J* = 10.7 Hz, 1H), 4.64 (m, 2H), 4.57 (M, 1H), 4.54 – 4.48 (m, 1H), 4.18 (t, *J* = 9.1 Hz, 2H), 4.12 (m, 4H), 4.08 (s, 1H), 4.01 (m, 3H), 3.94 (m, 1H), 3.92 – 3.87 (m, 2H), 3.81 (m, 2H), 3.68 (s, 3H), 3.63 (s, 1H), 3.52 (m, 2H), 3.27 (m, 1H), 3.19 (s, 3H), 3.05 (q, *J* = 6.2 Hz, 2H), 1.94 (d, *J* = 3.7 Hz, 6H), 1.67 – 1.62 (m, 2H), 1.13 (s, 9H), 1.11 (s, 9H). ^{13}C NMR (100 MHz, $CDCl_3$) δ 176.59, 176.41, 173.94, 173.92, 169.43, 168.89, 161.23, 142.85, 141.32, 141.27, 140.94, 139.67, 139.53, 139.31, 137.49, 137.25, 137.11, 136.65, 136.26, 134.13, 133.84, 133.52, 133.41, 133.34, 133.29, 132.93, 132.88, 132.44, 132.29, 132.00, 131.98, 131.84, 131.79, 131.68, 131.59, 131.52, 131.47, 131.43, 131.34, 131.25, 130.96, 104.61, 103.84, 100.75, 100.66, 86.23, 85.79, 81.15, 79.96, 78.66, 78.42, 78.35, 78.25, 77.91, 77.68, 77.62, 77.47, 76.92, 76.61, 76.15, 73.48, 70.94, 69.81, 68.95, 65.91, 64.71, 58.26, 57.20, 56.69, 55.68, 41.07, 36.56, 34.35, 33.28, 30.14, 29.98, 25.63, 25.61. HR-ESI-MS (*m/z*): [M], calculated for $C_{108}H_{121}N_3O_{33}S_2Si_2^{2-}$, 1054.3453; found, 1054.3454.

Synthesis of HS0S. The saponification and hydrogenolysis of compound **12** (0.017 mmol) using generation procedures D and E yielded 85% of **HS0S**. ^{1}H NMR (400 MHz, D_2O) δ 5.29 (dd, *J* = 5.4, 3.8 Hz, 2H), 4.46 (m, 2H), 3.95 (m, 3H), 3.82 (m, 3H), 3.77 – 3.59 (m, 13H), 3.56 – 3.51 (m, 1H), 3.45 (d, *J* = 9.2 Hz, 1H), 3.32 – 3.23 (m, 2H), 3.06 (t, *J* = 6.9 Hz, 2H), 1.96 (d, *J* = 3.6 Hz, 5H), 1.91 (s, 2H). ^{13}C NMR (100 MHz, D_2O) δ 176.90, 176.86, 104.72, 103.73, 100.24, 99.92, 82.14, 79.49, 79.31, 79.14, 78.63, 78.37, 75.86, 75.81, 75.66, 74.50,

74.02, 70.61, 70.47, 62.35, 61.89, 55.22, 54.73, 40.08, 29.21, 24.68, 24.61. HR-ESI-MS (*m/z*): $[M-H]^+$, calculated for $C_{31}H_{51}N_3O_{23}$, 833.2913; found, 833.2901.

Synthesis of HS6S. The saponification and hydrogenolysis of compound **12** (0.017 mmol) using generation procedures D and E yielded 75% of **HS6S**. ^{1}H NMR (400 MHz, D_2O) δ 5.28 (t, *J* = 3.2 Hz, 2H), 4.55 (d, *J* = 7.9 Hz, 1H), 4.44 (d, *J* = 8.0 Hz, 1H), 4.35 (s, 1H), 4.24 (m, 1H), 4.11 (m, 2H), 3.98 (d, *J* = 9.5 Hz, 1H), 3.91 (d, *J* = 9.3 Hz, 1H), 3.87 (d, *J* = 5.5 Hz, 1H), 3.85 (d, *J* = 5.4 Hz, 2H), 3.76 (d, *J* = 9.7 Hz, 3H), 3.71 (m, 3H), 3.66 (d, *J* = 5.2 Hz, 2H), 3.62 (d, *J* = 9.6 Hz, 2H), 3.49 (m, 1H), 3.28 (q, *J* = 8.4 Hz, 2H), 3.07 (t, *J* = 6.8 Hz, 2H), 1.97 (s, 3H), 1.96 (s, 3H), 1.94 – 1.90 (m, 2H). ^{13}C NMR (100 MHz, D_2O) δ 174.34, 173.02, 102.22, 101.92, 97.67, 97.33, 77.44, 76.74, 76.65, 76.03, 75.80, 74.99, 74.58, 73.26, 73.17, 70.40, 70.36, 69.05, 69.03, 68.79, 67.99, 66.34, 65.71, 53.41, 53.12, 37.56, 26.66, 21.92. HR-ESI-MS (*m/z*): [M], calculated for $C_{31}H_{49}N_3O_{29}S^{2-}$, 495.5952; found, 495.5948.

Synthesis of Compound HS3S. The saponification and hydrogenolysis of compound **13** (0.023 mmol) using generation procedure D and E yielded 77% of **HS3S**. ^{1}H NMR (400 MHz, D_2O) δ 5.39 (t, J = 4.1 Hz, 2H), 4.62 (d, J = 7.8 Hz, 1H), 4.56 (d, J = 9.7 Hz, 1H), 4.51 (m, 1H), 4.48 – 4.42 (m, 1H), 4.09 (m, 2H), 4.03 – 3.95 (m, 3H), 3.93 (d, J = 2.6 Hz, 1H), 3.89 – 3.80 (m, 7H), 3.75 (q, J = 7.6, 6.4 Hz, 4H), 3.69 (d, J = 8.9 Hz, 1H), 3.42 (t, J = 8.5 Hz, 1H), 3.33 (s, 1H), 3.16 (t, J = 6.6 Hz, 2H), 2.03 (s, 3H), 2.02 (s, 3H), 1.99 (d, J = 6.2 Hz, 2H). ^{13}C NMR (100 MHz, D_2O) δ 176.90, 176.86, 104.72, 103.73, 100.24, 99.92, 82.14, 79.49, 79.31, 79.14, 78.63, 78.37, 75.86, 75.81, 75.66, 74.50, 74.02, 70.61, 70.47, 62.35, 61.89, 55.22, 54.73, 40.08, 29.21, 24.68, 24.61. HR-ESI-MS (*m/z*): [M], calculated for $C_{31}H_{49}N_3O_{29}S_2^{2-}$, 495.5952; found, 495.5959.

3. **Synthesis of CS disaccharide analogs**

Synthesis of galactosamine building block

Commercially available D-galactosamine was used to prepare the required building block **22** in 6 steps. In the first step all the hydroxyl groups were protected with a labile acetyl group, and amine was protected with base sensitive trichloroacetate (TCA) group in a one-pot strategy. The obtained compound **b** was dissolved in DCM and thioglycosylated with *p*-thiocresol and $BF_3.Et_2O$ to obtain a semi solid compound. The compound was purified by column chromatography. Then, the acetate group was deprotected in sodium methoxide, followed by benzylidene protection of 4 and 6-OH group of **d**. The 3-OH group of **e** was protected with levulinic acid group to get the final donor **20.**

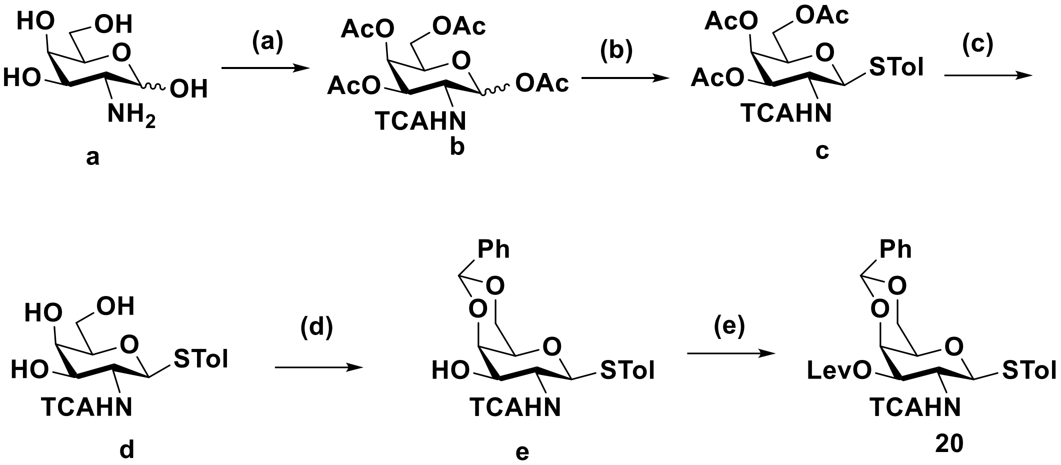

Scheme1. (a) Trichloroacetic anhydride, NaOMe, MeOH, 0 °C - RT, 1 h; Ac_2O, Py, 0 °C - RT, 24 h 91 % (b) *p*-Thiocresol, $BF_3.OEt_2$ DCM, 0 °C - RT 12 h, 76 %; (c) NaOMe, MeOH, RT, 2 h, 74 %; (d) $PhCH(OMe)_2$, PTSA, ACN, RT, 12 h, 76 %; (e) Levulinic acid (Lev-OH), DCC, DCM, DMAP, 1 h, 80 %;

Synthesis of Glucose building block

We synthesized D-glucose-based building block in 10 steps using some standard reported procedures. Briefly, all the hydroxyl groups of glucose were protected with base labile acetyl group using pyridine and acetic anhydride as a reagent. The compound was then dissolved in dry DCM, and p-thiocresol was added to the solution and stirred at 0 °C. Subsequently, mild lewis acid $BF_3.Et_2O$ was added dropwise to the reaction mixture. The reaction mixture was quenched and extracted with $NaHCO_3$ and purified by column chromatography to yield compound **9** as a white solid. The compound **h** was dissolved in methanol and basified with sodium methoxide to remove all acetate protecting groups. The selective 4 and 6-OH group protection using benzylidene and 3-OH by benzyl group was carried out as reported in standard dibutyl tinoxide reaction, followed by benzyl bromide treatment in the presence of CsF. Finally, 2-OH of **k** was protected with benzoyl group, resulted fully protected glucose building block **l** in moderate to good yield. The compound **l** was later subjected to benzylidene deprotection and selective 6-OH chloroacetate protection and followed by azidoethanol linker glycosylation yielded desired glucose **19** acceptor for further CS synthesis.

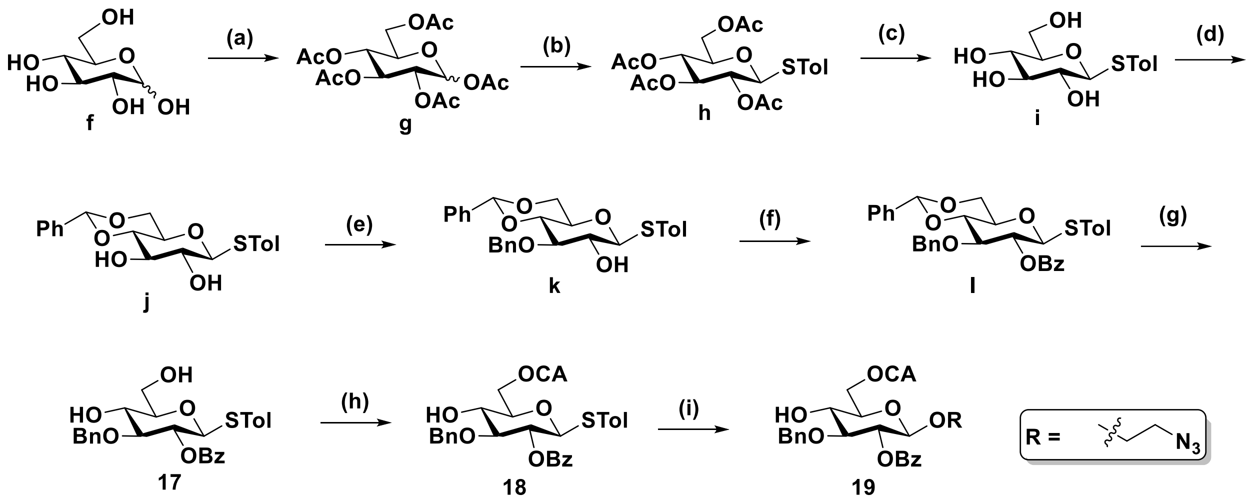

Scheme 2. (a) Ac_2O, Py 0 °C - RT 24 h, 95 %; (b) *p*-Thiocresol, $BF_3.OEt_2$ DCM, 0 °C - RT 12 h, 72 %; (c) NaOMe MeOH, RT, 2 h, 84 %; (d) $PhCH(OMe)_2$ PTSA, ACN, RT 8 h, 69 %; (e) Bu_2SnO, Bn-Br, CsF, Toluene, 120 °C, 18 h, 65 %; (f) Bz-Cl, DMAP, DCM:Py (4:1) 0 °C - RT 4 h, 81 %; (g) PTSA, DCM/MeOH (2:1) RT, 8 h , 82 %; (h) $(ClAc)_2O$, DCM/Py (4:1) -40 °C, 1 h, 78 %; (i) Azidoethanol, NIS, TMSOTf, DCM, 4Å MS -20 °C, 68 %.

Synthesis of chondroitin sulfate disaccharide analogs.

The CS disaccharide analogs have been synthesized from the previously synthesized building blocks **20**, and **19**. As shown in **scheme 2**, we first glycosylate the galactosamine donor **20** and glucose acceptor **19** was glycosylated NIS and TMSOTf promotor at -40 ºC yielded regioselective beta-glycosylated 81% of disaccharide precursor **21**. The compound **21** was subjected to selective chloroacetate deprotection using thiourea to convert glucose to glucuronic acid moiety. Then, oxidation of primary alcohol with a catalytic 2,2,6,6-tetramethyl-1-piperidinyloxyl free radical (TEMPO) and [bis(acetoxy)iodo]benzene (BAIB) and methyl esterification yielded CS disaccharide precursor **23**. The compound **23** wassubjected to global deprotection resulted in the final compound **CS0S** also same compound was subjected to benzylidene deprotection in the presence of PTSA yielded 4-and 6-OH free CS derivative **25**. The stoichiometric mixing of **25** and sulfation reagent sulfur trioxide:triethylamine complex in DMF and heating at 40 ºC for 72 hours resulted in desired sulfation patterns, Finally, global deprotection and hydrogenolysis yielded desired disaccharide final compounds **CS46S** and **CS6S**. The **CS3S** was synthesized by selective lev

deprotection of **23** in the presence of hydrazine.hydrate, followed by sulfation, global deprotection and hydrogenolysis.

Experimental procedure:

Synthesis of compound b: D-Galactosamine **a** (15 g, 69.76 mmol) was dissolved in MeOH (100 mL), NaOMe (5.6 g, 104.65 mmol) and Chloroacetic anhydride (15.29 mL, 83.72 mmol) were added at 0 °C drop wise. After 1 h the reaction mixture was neutralized with Amberlite® IR-120H resin and filtered, concentrated under reduced pressure. The crude product was (22.5 g, 55.55 mmol) was dissolved in pyridine (80 mL) and dropwise addition of acetic anhydride (52.67 mL, 557.27 mmol) at 0 °C. The reaction mixture allows stirring at room temperature for 24 h and concentrated under reduced pressure. The residue was extracted with EtOAc and washed with 2 N HCl (3x). The combined organic layer was dried over Na_2SO_4, filtered, and concentrated. The crude product was purified by column chromatography (EtOAc/Hexane = 1/2, v/v) to afford as mixture of α and β diastereomers (α:β = 1:4) **b** (31.8 g, 91 %) as pale yellow viscous liquid. ^{1}H NMR (400 MHz, $CDCl_3$) δ 6.67 (d, *J* = 8.7 Hz, 1H), 6.31 (d, *J* = 3.7 Hz, 1H), 5.45 (dd, *J* = 3.2, 1.1 Hz, 1H), 5.33 (dd, *J* = 11.4, 3.2 Hz, 1H), 4.61 –4.55 (m, 1H), 4.27 – 4.24 (m, 1H), 4.13 – 4.04 (m, 2H), 2.18 (s, 3H), 2.16 (s, 3H), 2.02 (s, 3H), 2.01 (s, 3H). ^{13}C NMR (100 MHz, $CDCl_3$) δ 171.12, 170.34, 170.04, 168.51, 162.07, 91.93, 90.29, 68.79, 67.57, 66.54, 61.15, 49.35, 20.78, 20.65, 20.64, 20.62. HRMS (ESI) m/z: calc'd for $[M+H]^+$ $C_{16}H_{21}Cl_3O_{10}NS$: 492.0231; Found: 492.0235.

Synthesis of compound c: Compound **b** (20 g, 40.73 mmol) was dissolved in anhydrous DCM (100 mL) and *p*-thiocresol (7.58 g, 61.09 mmol) was added under nitrogen atmospheric condition. Borontrifluoride.diethyletherate (15.08 mL, 122.19 mmol) was added drop wise at 0 ºC. After string 12 h the reaction mixture was quenched by triethylamine, and extracted with DCM (3x). The combined organic layer washed with $NaHCO_3$ and brine solution, dried over Na_2SO_4 and concentrated under reduced pressure. The crude product was purified by column chromatography (EtOAc/Hexane = 1/2, v/v) to afford **c** (17.2 g, 76 %) as white solid. ^{1}H NMR (400 MHz, $CDCl_3$) δ 7.46 – 7.43 (m, 2H), 7.18 – 7.13 (m, 2H), 6.81 (d, *J* = 9.0 Hz, 1H), 5.42 (dd, *J* = 3.3, 1.1 Hz, 1H), 5.31 (dd, *J* = 10.8, 3.3 Hz, 1H), 4.91 (d, *J* = 10.3 Hz, 1H), 4.24 – 4.11 (m, 3H), 3.99 – 3.95 (m, 1H), 2.37 (s, 3H), 2.15 (s, 3H), 2.07 (s, 3H), 2.00 (s, 3H). ^{13}C NMR (100 MHz, $CDCl_3$) δ 170.56, 170.47, 170.22, 161.83, 138.83, 133.46, 129.84,

128.37, 92.35, 87.11, 74.69, 70.69, 66.93, 61.73, 51.41, 21.29, 20.79, 20.74, 20.63. HRMS (ESI) m/z: calc'd for $[M+Na]^+$ $C_{21}H_{24}Cl_3O_8NSNa$: 578.0186; Found: 578.0182.

Synthesis of compound d: Compound **c** (15.5 g, 27.92 mmol) was dissolved in methanol (150 mL) and NaOMe (4.52 g, 83.78 mmol) was added at RT. After string 2 h the reaction mixture was neutralized with Amberlite® IR-120H resin and filtered, concentrated under reduced pressure. The crude product was purified by column chromatography (MeOH/DCM, 1/8) to afford **d** (8.9 g, 74 %) as white solid. ^{1}H NMR (400 MHz, CD_3OD) δ 7.39 – 7.37 (m, 2H), 7.08 (d, *J* = 8.1 Hz, 2H), 4.80 (d, *J* = 10.4 Hz, 1H), 4.03 (t, *J* = 10.3 Hz, 1H), 3.87 (d, *J* = 3.0 Hz, 1H), 3.78 – 3.68 (m, 3H), 3.53 – 3.50 (m, 1H), 2.27 (s, 3H). ^{13}C NMR (100 MHz, CD_3OD) δ 162.87, 137.36, 131.71, 130.83, 129.25, 87.76, 79.42, 72.05, 68.53, 61.25, 53.45, 19.77. HRMS (ESI) m/z: calc'd for $[M+Na]^+$ $C_{15}H_{18}Cl_3O_5NSNa$: 451.9869; Found: 451.9871.

Synthesis of compound e: Compound **d** (8.3 g, 19.34 mmol) was dissolved in acetonitrile (100 mL) and benzaldehyde dimethyl acetal (4.35 mL, 29.02 mmol) and *p*-Toluenesulfonic acid (0.73 g, 3.86 mmol) were added at RT. After string 12 h, the reaction mixture was quenched by triethylamine (up to P^H ~ 7) and concentrated under reduced pressure. The crude product was purified by column chromatography (EtOAc/Hexane = 1/1, v/v) to afford **e** (7.6 g, 76 %) as white solid. ^{1}H NMR (400 MHz, $CDCl_3$) δ 7.56 – 7.53 (m, 2H), 7.43 – 7.34 (m, 5H), 7.10 (d, *J* = 7.9 Hz, 1H), 6.78 (d, *J* = 7.6 Hz, 1H), 5.53 (s, 1H), 5.04 (d, *J* = 10.0 Hz, 1H), 4.39 (dd, *J* = 12.5, 1.5 Hz, 1H), 4.23 – 4.22 (m, 1H), 4.19 – 4.09 (m, 1H), 4.04 (dd, *J* = 12.5, 1.6 Hz, 1H), 3.72 – 3.65 (m, 1H), 3.60 – 3.58 (m, 1H), 2.64 (d, *J* = 10.4 Hz, 1H), 2.35 (s, 3H). ^{13}C NMR (100 MHz, $CDCl_3$) δ 161.95, 138.86, 137.45, 134.48, 129.85, 129.43, 128.24, 126.67, 126.55, 101.32, 92.46, 83.66, 74.98, 70.44, 70.09, 69.28, 54.15, 21.31. HRMS (ESI) m/z: calc'd for $[M+H]^+$ $C_{22}H_{23}Cl_3O_5NS$: 518.0363; Found: 518.0358.

Synthesis of compound 20: Compound **e** (9.5 g, 18.37 mmol) was dissolved in anhydrous DCM (80 mL). The reaction misture was cooled to 0 ºC and DCC (6.82 mL, 45.45 mmol) and DMAP (1.93 g, 11.36 mmol) and Levulinic acid (2.25 mL, 22.05 mmol) were added at the same temperature. After string 1 h, the reaction mixture was filtered on celite bed and concentrated under reduced pressure. The crude product was purified by column chromatography (EtOAc/Hexane = 1/1, v/v) to afford **20** (9.1 g, 80 %) as white solid. ^{1}H

NMR (400 MHz, $CDCl_3$) δ 7.56 – 7.53 (m, 2H), 7.43 – 7.34 (m, 5H), 7.10 (d, *J* = 7.9 Hz, 1H), 6.78 (d, *J* = 7.6 Hz, 1H), 5.53 (s, 1H), 5.04 (d, *J* = 10.0 Hz, 1H), 4.39 (dd, *J* = 12.5, 1.5 Hz, 1H), 4.23 – 4.22 (m, 1H), 4.19 – 4.09 (m, 1H), 4.04 (dd, *J* = 12.5, 1.6 Hz, 1H), 3.72 – 3.65 (m, 1H), 3.60 – 3.58 (m, 1H), 2.64 (d, *J* = 10.4 Hz, 1H), 2.35 (s, 3H). ^{13}C NMR (100 MHz, $CDCl_3$) δ 161.95, 138.86, 137.45, 134.48, 129.85, 129.43, 128.24, 126.67, 126.55, 101.32, 92.46, 83.66, 74.98, 70.44, 70.09, 69.28, 54.15, 21.31. HRMS (ESI) m/z: calc'd for $[M+H]^+$ $C_{27}H_{29}Cl_3O_7NS$: 616.0730; Found: 616.0732.

Synthesis of compound g: D-Glucose **f** (50 g, 277.7 mmol) was dissolved in pyridine (200 mL) and cooled the reaction at 0 ºC. Ac_2O (262 mL, 2777.7 mmol) was added to the reaction mixture dropwise and slowly allowed to room temperature. After 24 h stirring, the solvent concentrated. The mixture was diluted with EtOAc and washed with 1 N HCl solution (3x). The organic layer dried over Na_2SO_4, filtered and concentrated under reduced pressure. The crude product was purified by column chromatography (EtOAc/Hexane = 1/3 v/v) to afford **g** (103.4 g, 95 %) as a white solid. ^1H NMR (400 MHz, $CDCl_3$) δ 6.34 (d, *J* = 3.7 Hz, 1H), 5.51 – 5.46 (m, 1H), 5.20 – 5.07 (m, 2H), 4.33 – 4.23 (m, 1H), 4.18 – 4.06 (m, 2H), 2.19 (s, 3H), 2.10 (s, 3H), 2.05 (s, 3H), 2.04 (s, 3H), 2.03 (s, 3H). ^{13}C NMR (100 MHz, $CDCl_3$) δ 170.61, 170.61, 170.21, 169.64, 169.38, 168.74, 89.05, 69.81, 69.18, 67.88, 61.45, 20.87, 20.69, 20.66, 20.56, 20.44. HRMS (ESI) m/z: calc'd for $[M+Na]^+$ $C_{16}H_{22}O_{11}Na$: 413.1060; Found: 413.1059.

Synthesis of compound h: Compound **g** (30 g, 76.9 mmol) was dissolved in anhydrous DCM (150 mL) and *p*-thiocresol (11.4 g, 92.3 mmol) was added then cooled the reaction at 0 ºC. BF_3.OEt (28.4 mL, 230 mmol) was added to the reaction mixture dropwise and allowed to room temperature. After 12 h stirring, the reaction mixture was washed with aq. $NaHCO_3$ solution (2x). The combined organic layer dried over Na_2SO_4, filtered and concentrated under reduced pressure. The crude product was purified by column chromatography (EtOAc/Hexane = 1/3 v/v) to afford **h** (25.3 g, 72 %) as a white solid.^1H NMR (400 MHz, $CDCl_3$) δ 7.38 (dd, *J* = 8.2, 2.0 Hz, 2H), 7.11 (d, *J* = 8.0 Hz, 2H), 5.20 (t, *J* = 9.4 Hz, 1H), 5.01 (t, *J* = 9.8 Hz, 1H), 4.92 (t, *J* = 9.7 Hz, 1H), 4.63 (d, *J* = 10.1 Hz, 1H), 4.24 – 4.13 (m, 2H), 3.69 (ddt, *J* = 9.0, 4.2, 2.0 Hz, 1H), 2.34 (s, 3H), 2.08 (s, 3H), 2.07 (s, 3H), 2.00 (s, 3H), 1.97 (zs, 3H). ^{13}C NMR (100 MHz, $CDCl_3$) δ 170.51, 170.50, 170.13, 170.11, 169.35, 169.19, 138.74, 133.80, 129.65, 127.51, 85.74, 75.71, 73.99, 69.89, 68.17, 62.09, 21.17,

20.74, 20.71, 20.57, 20.55. HRMS (ESI) m/z: calc'd for $[M+Na]^+$ $C_{21}H_{26}O_9SNa$: 413.1195; Found: 477.1195.

Synthesis of compound i: Compound **h** (26 g, 57.2 mmol) was dissolved in MeOH (250 mL) and NaOMe (12.3 g, 229.0 mmol) was added at room temperature. After 2 h stirring, the reaction mixture was quenched with amberlite™ IR-120(H) resin. The resin was filtered and the solvent was concentrated under reduced pressure. The crude product was purified by column chromatography (MeOH/DCM, 1/9) to afford **i** (13.9 g, 84 %) as a white solid.^{1}H NMR (400 MHz, CD_3OD) δ 7.53 – 7.42 (m, 2H), 7.20 – 7.07 (m, 2H), 4.53 (d, *J* = 9.7 Hz, 1H), 3.87 (dd, *J* = 12.1, 1.7 Hz, 1H), 3.72 – 3.64 (m, 1H), 3.38 (d, *J* = 9.3 Hz, 1H), 3.30 (ddd, *J* = 5.5, 3.9, 2.1 Hz, 2H), 3.20 (dd, *J* = 9.7, 8.7 Hz, 1H), 2.33 (s, 3H). ^{13}C NMR (100 MHz, CD_3OD) δ 141.30, 136.05, 133.71, 133.08, 92.18, 84.54, 82.19, 76.21, 73.89, 65.43, 23.66. HRMS (ESI) m/z: calc'd for $[M+Na]^+$ $C_{13}H_{18}O_5SNa$: 309.0773; Found: 309.0777.

Synthesis of compound j: Compound **i** (20 g, 69.9 mmol) was dissolved in ACN (150 mL) and benzylidene dimethyl acetal (15.7 mL, 104.8 mmol) and PTSA (2.6 g, 13.9 mmol) were added at room temperature. After 8 h stirring, the reaction mixture was quenched with Et_3N and washed with aq. $NaHCO_3$ solution (2x). The combined organic layer dried over Na_2SO_4, filtered and concentrated under reduced pressure. The crude product was purified by column chromatography (MeOH/DCM, 1/9.5) to afford **j** (18.2 g, 69 %) as a white solid.^{1}H NMR (400 MHz, $CDCl_3$) δ 7.53 – 7.41 (m, 4H), 7.42 – 7.34 (m, 3H), 7.20 – 7.13 (m, 2H), 5.52 (s, 1H), 4.55 (d, *J* = 9.7 Hz, 1H), 4.37 (dd, *J* = 10.5, 4.3 Hz, 1H), 3.86 – 3.69 (m, 2H), 3.55 – 3.37 (m, 3H), 3.21 (d, *J* = 2.3 Hz, 1H), 2.97 (d, *J* = 2.6 Hz, 1H), 2.38 (s, 3H). ^{13}C NMR (100 MHz, $CDCl_3$) δ 138.82, 136.90, 133.65, 129.90, 129.34, 128.38, 127.36, 126.33, 101.91, 88.68, 80.22, 74.51, 72.51, 70.49, 68.58, 21.21. HRMS (ESI) m/z: calc'd for $[M+Na]^+$ $C_{20}H_{22}O_5SNa$: 397.1086; Found: 397.1085.

Synthesis of compound k: Compound **j** (15 g, 40.1 mmol) was dissolved in toluene (150 mL) and dibutyltinoxide (10.9 g, 44.1 mmol) was added at room temperature then reflex at 120 ºC. After 6 h stirring, the reaction mixture was cooled to ambient temperature and concentrated under reduced pressure. The crude product was kept in high vacuum for 2 h and dissolved in DMF:Toluene (v/v 1/1, 200ml). Benzyl bromide (5.2 mL, 44.1 mmol) and CsF (6.7 g, 44.1 mmol) were added at room temperature then reflex at 120 ºC. After 12 h reflex the reaction mixture was cooled to ambient temperature. The solvents were evaporated then

diluted with EtOAc and washed with brine solution (3x). The organic layer dried over Na_2SO_4, filtered and concentrated under reduced pressure. The crude product was purified by column chromatography (EtOAc/Hexane = 1/5, v/v) to afford compound **k** (12.1 g, 65 %) as a white solid. ^{1}H NMR (400 MHz, $CDCl_3$) δ 7.52 – 7.41 (m, 4H), 7.41 – 7.26 (m, 8H), 7.13 (d, *J* = 8.0 Hz, 2H), 5.56 (s, 1H), 4.94 (d, *J* = 11.5 Hz, 1H), 4.79 (d, *J* = 11.5 Hz, 1H), 4.56 (d, *J* = 9.6 Hz, 1H), 4.38 (dd, *J* = 10.5, 4.9 Hz, 1H), 3.78 (t, *J* = 10.3 Hz, 1H), 3.73 – 3.58 (m, 2H), 3.49 (td, *J* = 9.7, 4.8 Hz, 2H), 2.62 (bs, 1H), 2.34 (s, 3H). ^{13}C NMR (100 MHz, $CDCl_3$) δ 138.86, 138.29, 137.29, 133.93, 129.94, 129.13, 128.58, 128.38, 128.24, 128.00, 127.30, 126.11, 101.32, 88.66, 81.71, 81.22, 74.93, 72.20, 70.80, 68.73, 21.30. HRMS (ESI) m/z: calc'd for $[M+H]^+$ $C_{27}H_{29}O_5S$: 465.1736; Found: 465.1735.

Synthesis of compound l: Compound **k** (10 g, 21.5 mmol) was dissolved in mixture of DCM:Pyridine (4/2, v/v, 100 mL) and cooled the reaction mixture at 0 °C. Dimethyl aminopyridine (DMAP) (0.52 g, 4.3 mmol) and benzoyl chloride (7.5 mL, 64.6 mmol) were added at the same room temperature. After 4 h stirring, the reaction mixture was washed with aq. $NaHCO_3$ solution (2x) and 1 N HCl solution (3x). The combined organic layer dried over Na_2SO_4, filtered and concentrated under reduced pressure. The crude product was purified by column chromatography (EtOAc/Hexane = 1/5, v/v) to afford **l** (9.9 g, 81 %) as a white solid. ^{1}H NMR (400 MHz, $CDCl_3$) δ 8.07 – 7.97 (m, 2H), 7.66 – 7.55 (m, 1H), 7.54 – 7.42 (m, 4H), 7.45 – 7.34 (m, 5H), 7.15 – 7.02 (m, 7H), 5.60 (s, 1H), 5.26 (dd, *J* = 9.9, 8.7 Hz, 1H), 4.82 – 4.75 (m, 2H), 4.66 (d, *J* = 11.9 Hz, 1H), 4.41 (dd, *J* = 10.5, 5.0 Hz, 1H), 3.93 – 3.73 (m, 3H), 3.55 (td, *J* = 9.7, 5.0 Hz, 1H), 2.32 (s, 1H). ^{13}C NMR (100 MHz, $CDCl_3$) δ 165.13, 138.65, 137.77, 137.25, 133.77, 133.32, 130.03, 129.91, 129.77, 129.16, 128.50, 128.40, 128.26, 128.18, 127.68, 126.10, 101.34, 87.26, 81.51, 79.42, 74.35, 72.08, 70.65, 68.70, 21.28. HRMS (ESI) m/z: calc'd for $[M+H]^+$ $C_{34}H_{33}O_6S$: 569.1998; Found: 569.1998.

Synthesis of compound 17: Compound **l** (9.7 g, 17.0 mmol) was dissolved in mixture of DCM:MeOH (2/1, v/v, 90 mL) and PTSA (4.8 g, 25.6 mmol) was added at room temperature. After 8 h stirring, the reaction mixture was quenched with Et_3N then washed with aq. $NaHCO_3$ solution (2x). The combined organic layer dried over Na_2SO_4, filtered and concentrated under reduced pressure. The crude product was purified by column chromatography (EtOAc/Hexane = 1/3, v/v) to afford **17** (6.7 g, 82 %) as a white solid. ^{1}H NMR (400 MHz, $CDCl_3$) δ 8.09 – 8.04 (m, 2H), 7.62 –7.58 (m, 1H), 7.50 – 7.44 (m, 2H), 7.34 – 7.29 (m, 2H), 7.21 –7.06 (m, 5H), 7.07 (d, *J* = 7.9 Hz, 2H), 5.25 – 5.19 (m, 1H), 4.80 –

4.52 (m, 3H), 3.92 (dd, *J* = 11.9, 3.3 Hz, 1H), 3.79 (dd, *J* = 12.0, 5.0 Hz, 1H), 3.74 – 3.66 (m, 2H), 3.46 – 3.42 (m, 1H), 2.80 (bs, 0H), 2.30 (s, 3H). ^{13}C NMR (100 MHz, $CDCl_3$) δ 165.24, 138.39, 137.69, 133.36, 133.14, 129.90, 129.76, 129.73, 128.68, 128.52, 128.05, 127.99, 86.65, 83.87, 79.44, 74.81, 72.40, 70.31, 62.56, 21.15. HRMS (ESI) m/z: calc'd for $[M+Na]^+$ $C_{27}H_{28}O_6SNa$: 503.1504; Found: 503.1501.

Synthesis of compound 18: Compound **17** (5.6 g, 11.6 mmol) was dissolved in anhydrous DCM (50 mL) and cooled the reaction mixture at -40 ºC. Chloroacetic anhydride (1.79 g, 10.5 mmol) was added at the same room temperature. After 1 h stirring, the reaction mixture was allowed to ambient temperature and washed with 1 N HCl solution (3x). The combined organic layer dried over Na_2SO_4, filtered and concentrated under reduced pressure. The crude product was purified by column chromatography (EtOAc/Hexane = 1/5, v/v) to afford **18** (5.1 g, 78 %) as a white solid. ^{1}H NMR (400 MHz, $CDCl_3$) δ 8.15 – 8.07 (m, 2H), 7.68 – 7.60 (m, 1H), 7.56 – 7.46 (m, 2H), 7.41 – 7.33 (m, 2H), 7.28 – 7.16 (m, 5H), 7.16 – 7.08 (m, 2H), 5.24 (dd, *J* = 10.0, 8.9 Hz, 1H), 4.79 – 4.58 (m, 3H), 4.57 (dd, *J* = 11.9, 1.7 Hz, 1H), 4.54 – 4.43 (m, 1H), 4.13 – 4.08 (m, 10H), 3.76 – 3.65 (m, 1H), 3.69 – 3.58 (m, 2H), 2.62 (d, *J* = 2.2 Hz, 1H), 2.35 (s, 3H). ^{13}C NMR (100 MHz, $CDCl_3$) δ 167.45, 165.18, 138.45, 137.52, 133.51, 133.41, 129.89, 129.72, 129.58, 128.59, 128.55, 128.43, 128.11, 128.10, 86.53, 83.73, 74.97, 72.15, 69.77, 64.78, 40.74, 21.17. HRMS (ESI) m/z: calc'd for $[M+Na]^+$ $C_{29}H_{29}O_7SNa$: 579.1220; Found: 579.1218.

Synthesis of compound 19: Compound **18** (1.5 g, 2.69 mmol) and azidoethanol (0.35 g, 4.04 mmol) were dissolved in anhydrous DCM (20 mL) with 4Å molecular sieves and stirred at room temperature for 2 h. The reaction mixture cooled at -40 ºC and NIS (0.72 g, 3.23 mmol) and TMSOTf (97 µL, 0.53 mmol) were added at the same room temperature. After 30 min, the reaction mixture was quenched Et_3N then allowed to ambient temperature and the molecular sieves was filtered on celite bed. The reaction mixture was washed with aq. $Na_2S_2O_3$ solution (2x). The combined organic layer dried over Na_2SO_4, filtered and concentrated under reduced pressure. The crude product was purified by column chromatography (EtOAc/Hexane = 1/5, v/v) to afford **19** (0.95 g, 68 %) as a white solid. ^{1}H NMR (400 MHz, $CDCl_3$) δ 8.08 – 8.04 (m, 2H), 7.62 – 7.56 (m, 1H), 7.48 – 7.44 (m, 1H), 7.24 – 7.18 (m, 5H), 5.29 – 5.25 (m, 1H), 4.78 – 4.59 (m, 3H), 4.56 – 4.44 (m, 2H), 4.12 (s, 2H), 3.99 – 3.94 (m, 1H), 3.72 – 3.57 (m, 4H), 3.42 – 3.36 (m, 1H), 3.30 – 3.25 (m, 1H), 2.64

(bs, 1H). ^{13}C NMR (100 MHz, $CDCl_3$) δ 167.59, 165.17, 137.59, 133.29, 129.80, 129.71, 128.59, 128.46, 128.09, 101.21, 82.12, 74.75, 73.51, 73.20, 69.79, 68.10, 64.49, 50.67, 40.76. HRMS (ESI) m/z: calc'd for $[M+Na]^+$ $C_{24}H_{26}O_8ClN_3SNa$: 542.1306; Found: 542.1310.

Synthesis of compound 21: Compound (Glycosyl donor) **20** (1 g, 1.62 mmol) and (glycosyl acceptor) **21** (0.75 g, 1.46 mmol) were dissolved in anhydrous DCM (20 mL) with freshly dried 4 Å MS and stirred at RT for 1 h. Cooled the reaction mixture at -40 °C and NIS (0.43 g, 1.95 mmol) and TMSOTf (58 µL, 0.32 mmol) were added at the same temperature. After consumption of donor, the reaction mixture was quenched with Et_3N and filtered on celite bed and the reaction mixture was washed with aq. $Na_2S_2O_3$ solution (2x). The combined organic layaer was dried over Na_2SO_4 and concentrated under reduced pressure. The crude product was purified by column chromatography (EtOAc/Hexane = 1/1, v/v) to afford **21** (1.2 g, 81 %) as white foam. ^{1}H NMR (400 MHz, $CDCl_3$) δ 8.00 – 7.92 (m, 2H), 7.59 – 7.53 (m, 1H), 7.48 – 7.39 (m, 4H), 7.31 – 7.29 (m, 1H), 7.17 – 7.11 (m, 3H), 7.08 – 6.97 (m, 3H), 5.50 (s, 1H), 5.29 – 5.23 (m, 1H), 5.19 – 5.15 (m, 1H), 5.00 (d, *J* = 11.8 Hz, 1H), 4.84 (d, *J* = 8.2 Hz, 1H), 4.75 (d, *J* = 11.8 Hz, 1H), 4.62 (d, *J* = 7.7 Hz, 1H), 4.57 – 4.45 (m, 2H), 4.43 – .35 (m, 1H), 4.28 – 4.19 (m, 2H), 4.13 (d, *J* = 2.1 Hz, 2H), 4.02 (dd, *J* = 9.6, 8.2 Hz, 1H), 3.96 – 3.89 (m, 3H), 3.69 – 3.60 (m, 2H), 3.39 – 3.32 (m, 2H), 3.29v – 3.23 (m, 1H), 2.73 – 2.66 (m, 2H), 2.58 – 2.54 (m, 1H), 2.07 (s, 3H). ^{13}C NMR (100 MHz, $CDCl_3$) δ 206.51, 172.60, 167.45, 165.14, 162.18, 138.14, 137.54, 133.12, 129.83, 129.74, 129.08, 128.45, 128.33, 128.23, 128.15, 128.04, 127.99, 127.93, 127.83, 127.27, 126.36, 126.33, 126.25, 100.93, 100.90, 100.60, 92.46, 80.51, 77.26, 74.64, 72.85, 72.82, 72.79, 70.38, 68.70, 68.12, 66.45, 63.98, 52.99, 50.61, 40.84, 37.76, 29.71, 28.11. HRMS (ESI) m/z: calc'd for $[M+H]^+$ $C_{44}H_{47}O_{15}Cl_4N_4$: 1011.1792; Found: 1011.1787.

Synthesis of compound 22: Compound **21** (0.85 g, 0.84 mmol) was dissolved in mixture of Pyridine:MeOH [(v/v), (1:1), 20 mL] and thiourea (0.12 g, 1.68 mmol) was added. The reaction mixture was reflux at 80 °C for 2 h. The mixture was allow to ambient temperature and diluated with EtOAc and washed with 1N HCl solution (3x) and brine solution (2x). The combined organic layer dried over Na_2SO_4, filtered and concentrated under reduced pressure. The crude product was purified by column chromatography (EtOAc/Hexane = 1/0.5, v/v) to afford **22** (0.69 g, 88 %) as a white solid. ^{1}H NMR (400 MHz, $CDCl_3$) δ 8.01 – 7.94 (m, 2H), 7.60 – 7.54 (m, 1H), 7.48 – 7.40 (m, 5H), 7.33 – 7.26 (m, 3H), 7.17 – 7.12 (m, 2H), 7.05 –

7.00 (m, 1H), 6.93 (dd, *J* = 8.2, 6.8 Hz, 2H), 5.44 (s, 1H), 5.25 – 5.17 (m, 2H), 5.09 (d, *J* = 11.8 Hz, 1H), 4.95 (d, *J* = 8.4 Hz, 1H), 4.74 (d, *J* = 11.8 Hz, 1H), 4.60 (d, *J* = 7.9 Hz, 1H), 4.48 (dt, *J* = 11.3, 8.8 Hz, 1H), 4.28 – 4.10 (m, 3H), 3.97 – 3.81 (m, 5H), 3.63 (ddd, *J* = 10.8, 6.9, 3.9 Hz, 1H), 3.50 – 3.26 (m, 4H), 3.12 (s, 1H), 2.77 – 2.52 (m, 4H), 2.04 (s, 3H). ^{13}C NMR (100 MHz, $CDCl_3$) δ 206.46, 172.64, 165.44, 162.19, 138.38, 137.74, 133.13, 129.89, 129.80, 128.99, 128.33, 128.30, 128.10, 127.97, 127.16, 126.39, 101.11, 100.85, 100.74, 92.72, 80.32, 76.39, 75.22, 75.05, 73.03, 72.83, 70.84, 68.80, 68.07, 66.26, 60.73, 52.83, 50.71, 37.78, 29.70, 28.26. HRMS (ESI) m/z: calc'd for $[M+H]^+$ $C_{42}H_{45}O_{14}Cl_3N_4$: 957.1896; Found: 957.1899.

Synthesis of compound 23: Compound **22** (0.65 g, 0.69 mmol) is dissolved in mixture of DCM/^{t}BuOH/H_2O [(4:1:1), (v/v/v), 20 mL] were added 2,2,6,6-tetramethyl-1-piperidinyloxyl free radical (TEMPO, 89.9 mg, 0.17 mmol) and bis(acetoxy)iodobenzene (BAIB, 0.31 g, 1.39 mmol) at room temperature. After 6 h, the mixture was quenched with saturated NH_4Cl solution and extracted with DCM. The organic layer was dried over Na_2SO_4, filtered, and concentrated under reduced pressure. The residue was taken as such for next step without further purification. The crude was dissolved in DMF (10 mL) and K_2CO_3 (0.28 g, 2.08 mmol) methyl iodide (0.38 mL, 6.25 mmol) were added at the room temperature. After string 12 h, the reaction mixture diluted with ethyl acetate and washed with brine solution (3x), the combined organic layer was dried over Na_2SO_4, filtered and concentrated under reduced pressure. The crude product was purified by column chromatography (EtOAc/Hexane = 1/1 v/v) to afford **23** (0.55 g, 82 %) as white solid. ^1H NMR (400 MHz, $CDCl_3$) δ 7.97 – 7.92 (m, 2H), 7.59 – 7.54 (m, 1H), 7.50 – 7.39 (m, 4H), 7.30 – 7.28 (m, 2H), 7.20 – 7.15 (m, 2H), 7.07 – 6.93 (m, 4H), 5.52 (s, 1H), 5.23 (dd, *J* = 8.8, 6.8 Hz, 1H), 5.15 – 5.00 (m, 3H), 4.76 – 4.61 (m, 2H), 4.48 (dt, *J* = 11.2, 8.7 Hz, 1H), 4.35 – 4.18 (m, 3H), 4.08 – 3.86 (m, 4H), 3.84 (s, 3H), 3.67 (ddd, *J* = 11.0, 7.0, 4.2 Hz, 1H), 3.51 (q, *J* = 1.5 Hz, 1H), 3.42 – 3.26 (m, 2H), 2.83 – 2.55 (m, 4H), 2.05 (s, 3H). ^{13}C NMR (100 MHz, $CDCl_3$) δ 206.43, 172.47, 170.06, 165.18, 161.97, 138.02, 137.68, 133.14, 129.83, 129.65, 129.02, 128.36, 128.33, 128.12, 128.00, 127.32, 126.41, 101.34, 100.95, 100.74, 92.64, 79.74, 78.56, 75.44, 74.17, 73.16, 72.96, 71.54, 68.66, 68.38, 66.70, 53.11, 52.40, 50.56, 37.73, 29.70, 28.20. HRMS (ESI) m/z: calc'd for $[M+Na]^+$ $C_{43}H_{45}O_{15}Cl_3N_4Na$: 985.1845; Found: 985.1841.

Synthesis of compound 24: Compound **23** (0.1 g, 0.1 mmol) was dissolved in mixture of THF/MeOH [(v/v), (10/1), xx mL]. Hydrazine hydrate/acetic acid [(v/v), (2.5/1)] were

added in mixture THF:MeOH (5:1) [(v/v), (10/1)], the resulting solution was added to reaction mixture at room temperature. After 1 h the reaction mixture was diluted with EtOAc and washed with aq. $NaHCO_3$ solution (3x) and brine solution (2x). The combined organic layer was dried over Na_2SO_4, filtered and concentrated under reduced pressure. The crude product was purified by column chromatography (EtOAc/Hexane = 1/0.7, v/v) to afford **24** (72 mg, 80 %) as white solid.^{1}H NMR (400 MHz, $CDCl_3$) δ 7.99 – 7.91 (m, 2H), 7.61 – 7.50 (m, 1H), 7.50 – 7.36 (m, 5H), 7.34 – 7.24 (m, 3H), 7.19 (d, *J* = 6.6 Hz, 2H), 7.07 – 6.95 (m, 2H), 5.55 (s, 1H), 5.23 (dd, *J* = 8.8, 7.0 Hz, 1H), 5.09 (d, *J* = 11.4 Hz, 1H), 4.90 (d, *J* = 8.4 Hz, 1H), 4.72 – 4.61 (m, 2H), 4.28 – 4.15 (m, 4H), 4.08 – 3.90 (m, 4H), 3.89 – 3.79 (m, 4H), 3.80 – 3.74 (m, 1H), 3.74 – 3.61 (m, 1H), 3.48 (s, 1H), 3.38 – 3.27 (m, 2H), 2.77 (d, *J* = 10.6 Hz, 1H). ^{13}C NMR (100 MHz, $CDCl_3$) δ 170.60, 165.30, 163.22, 138.05, 137.65, 133.30, 129.99, 129.91, 129.68, 129.29, 128.50, 128.46, 128.36, 128.32, 128.13, 127.50, 126.43, 126.37, 101.42, 101.30, 100.80, 92.81, 79.99, 79.02, 75.78, 74.91, 74.18, 73.25, 72.46, 68.77, 68.59, 66.98, 55.64, 53.28, 50.64. HRMS (ESI) m/z: calc'd for $[M+H]^+$ $C_{38}H_{40}O_{13}Cl_3N_4$: 865.1657; Found: 865.1651.

Synthesis of benzylidene deprotected 25: Compound **23** (0.5 g, 0.51 mmol) was dissolved in mixture of DCM/MeOH [(2/1), (v/v), 12 mL] and *p*-Toluenesulfonic acid (PTSA, 0.14 g, 0.77 mmol) was added at RT. After 6 h stirring, the mixture was quenched by triethylamine (up to PH ~ 7) and concentrated under reduced pressure. The crude product was purified by column chromatography (EtOAc/Hexane = 1/0.5, v/v) to afford **benzylidene deprotected 25** (0.4 g, 88 %) as white solid. ^{1}H NMR (400 MHz, $CDCl_3$) δ 8.04 – 7.92 (m, 2H), 7.62 – 7.54 (m, 1H), 7.50 – 7.40 (m, 2H), 7.27 – 7.10 (m, 5H), 7.03 (d, *J* = 9.0 Hz, 1H), 5.27 – 5.21 (m, 1H), 4.98 – 4.88 (m, 2H), 4.75 – 4.65 (m, 2H), 4.42 (dt, *J* = 11.0, 8.7 Hz, 1H), 4.28 (t, *J* = 8.9 Hz, 1H), 4.12 – 4.04 (m, 2H), 3.99 (ddt, *J* = 10.7, 5.6, 3.5 Hz, 1H), 3.88 (d, *J* = 3.5 Hz, 1H), 3.85 (d, *J* = 3.5 Hz, 3H), 3.74 – 3.58 (m, 3H), 3.58 – 3.50 (m, 1H), 3.37 (qdd, *J* = 13.3, 6.3, 3.9 Hz, 2H), 3.14 (s, 1H), 2.85 – 2.71 (m, 2H), 2.67 – 2.52 (m, 2H), 2.22 (s, 3H). ^{13}C NMR (100 MHz, $CDCl_3$) δ 207.86, 172.25, 169.72, 165.25, 162.17, 137.70, 133.26, 129.79, 129.55, 128.50, 128.41, 128.27, 128.24, 128.00, 127.95, 127.89, 127.76, 101.27, 100.58, 92.65, 79.66, 77.50, 75.42, 74.69, 74.12, 73.79, 72.94, 68.39, 67.12, 62.23, 60.42, 53.10, 52.43, 50.54, 38.04, 29.84, 28.14, 21.07, 14.20. HRMS (ESI) m/z: calc'd for $[M+H]^+$ $C_{36}H_{41}O_{15}Cl_3N_4$: 897.1532; Found: 897.1533.

General Procedure for sulfation reaction

The respective compound (1 eq) was dissolved in DMF (2 mL) and $SO_3 \cdot NEt_3$ (10 mmol per -OH) and microwaved at 100 °C, 15 min using a CEM Initiator synthesizer in sealed reaction vessels (average power 15 w). The solvent was evaporated and purified by silica column chromatography and lyophilized to afford corresponding sulfated compounds.

General procedure for hydrolysis and hydrogenolysis reaction

The respective compound **sulfated or non-sulfated CS disaccharide precursor** (1 eq.) and lithium hydroxide (5 eq per ester) were dissolved in the mixture of 1,4-dioxane and water [2/1 (v/v), 6 mL] and refluxed at 80 °C for 12 h. After the reaction completion, the mixture was cooled to room temperature and neutralized with amberlite® IR 120 resin, filtered, and concentrated under reduced pressure. The residue was purified by bond elute using water then lyophilized to get corresponding hydrolysis compounds which was proceeded for N-acetylation reaction. The crude product was dissolved in MeOH (3 mL) and cooled at 0 °C. Triethylamine (10 eq per amine) and Ac_2O (10 eq per amine) were added. After 12 h, solvent was concentrated and purified by bond elute using water then lyophilized to get corresponding compounds **23, 24, 26a** and **26b** which are also proceeded for the hydrogenolysis, using $Pd(OH)_2$ on charcoal with hydrogen gas in H_2O (3 mL). After 12 - 24 h (depend on the compounds), the mixture was filtered on Whatman 42 filter paper. The residue was purified by bond elute using water then lyophilized to get corresponding compounds **CS0S**, **CS3S**, **CS46S** and **CS6S.**

Synthesis of 3-O-sulfated CS precursor: Compound **24** (40 mg, 0.04 mmol) followed the general procedure for sulfation reaction to afford **3-O-sulfated CS precursor** (32 mg, 73%) as a white solid. ^1H NMR (400 MHz, CD_3OD) δ 7.99 – 7.94 (m, 2H), 7.62 (ddt, *J* = 8.8, 7.8, 1.3 Hz, 1H), 7.52 – 7.44 (m, 4H), 7.27 – 7.18 (m, 3H), 7.17 – 7.12 (m, 2H), 7.04 – 6.98 (m, 1H), 6.93 – 6.88 (m, 2H), 5.66 (s, 1H), 5.18 – 5.09 (m, 2H), 4.98 (d, *J* = 8.2 Hz, 1H), 4.77 – 4.64 (m, 3H), 4.41 – 4.30 (m, 3H), 4.20 – 4.11 (m, 2H), 4.00 – 3.90 (m, 2H), 3.86 (s, 3H), 3.75 – 3.62 (m, 2H), 3.35 (d, *J* = 3.5 Hz, 1H), 3.28 (ddd, *J* = 13.4, 5.6, 3.5 Hz, 1H). ^{13}C NMR (100 MHz, CD_3OD) δ 169.21, 165.54, 163.22, 138.39, 138.24, 133.00, 129.89, 129.55, 128.35, 128.30, 128.16, 127.62, 127.54, 126.96, 126.26, 100.95, 100.77, 100.20, 80.07, 76.85, 75.03, 74.26, 73.75, 72.73, 68.62, 68.50, 66.77, 52.90, 52.23, 50.38, 48.55. HRMS (ESI) m/z: calc'd for $[M+H]^-$ $C_{38}H_{39}O_{16}Cl_3N_4S^-$: 944.1153; Found: 944.1157.

Synthesis of compound 26a: Compound **25** (35 mg, 0.03 mmol) followed the general procedure for sulfation reaction to afford **26a** (25 mg, 66 %) as a white solid. ^1H NMR (400

MHz, CD_3OD) δ 8.07 – 7.98 (m, 2H), 7.67 – 7.59 (m, 1H), 7.54 – 7.46 (m, 2H), 7.29 – 7.22 (m, 2H), 7.20 – 7.10 (m, 3H), 5.15 (t, *J* = 7.6 Hz, 1H), 5.10 (dd, *J* = 11.2, 3.2 Hz, 1H), 4.95 – 4.90 (m, 3H), 4.68 (d, *J* = 11.4 Hz, 1H), 4.40 (t, *J* = 7.6 Hz, 1H), 4.36 – 4.30 (m, 1H), 4.27 – 4.12 (m, 4H), 4.01 – 3.92 (m, 2H), 3.89 (ddd, *J* = 7.0, 5.8, 1.1 Hz, 1H), 3.83 (s, 3H), 3.69 (ddd, *J* = 11.0, 7.4, 3.6 Hz, 1H), 3.44 – 3.33 (m, 2H), 3.32 – 3.27 (m, 1H), 2.87 – 2.74 (m, 2H), 2.67 – 2.54 (m, 2H), 2.18 (s, 3H). ^{13}C NMR (100 MHz, CD_3OD) δ 208.05, 172.16, 169.19, 165.53, 162.97, 138.01, 132.96, 129.78, 129.53, 128.13, 127.99, 127.77, 127.70, 127.10, 100.52, 99.45, 92.69, 79.24, 76.05, 74.58, 73.78, 72.83, 72.78, 72.72, 68.36, 65.27, 64.89, 52.43, 52.10, 50.31, 37.14, 29.35, 28.36, 27.53. HRMS (ESI) m/z: calc'd for $[M+H]^-$ $C_{36}H_{41}O_{14}Cl_3N_4S^-$: 954.1208; Found: 954.1215.

Synthesis of compound 26b: Compound **25** (30 mg, 0.034 mmol) followd the gerenral produre for sulfation reaction to afford **26b** (29 mg, 82 %) as a white solid. ^1H NMR (400 MHz, CD_3OD) δ 8.06 – 7.99 (m, 2H), 7.67 – 7.59 (m, 1H), 7.54 – 7.47 (m, 2H), 7.28 – 7.22 (m, 2H), 7.17 – 7.09 (m, 3H), 5.18 (dd, *J* = 8.1, 3.3 Hz, 1H), 5.16 – 5.13 (m, 1H), 5.00 – 4.89 (m, 5H), 4.68 (d, *J* = 11.4 Hz, 1H), 4.48 – 4.40 (m, 2H), 4.31 – 4.19 (m, 3H), 4.11 – 4.05 (m, 1H), 4.03 – 3.92 (m, 2H), 3.83 (s, 3H), 3.73 – 3.66 (m, 2H), 3.41 – 3.35 (m, 2H), 3.32 – 3.25 (m, 1H), 2.91 – 2.70 (m, 2H), 2.62 (t, *J* = 6.5 Hz, 2H), 2.16 (s, 3H). ^{13}C NMR (100 MHz, CD_3OD) δ 208.32, 172.59, 169.12, 165.58, 162.93, 138.02, 132.98, 129.79, 129.51, 128.16, 127.74, 127.00, 100.63, 99.11, 92.65, 79.23, 76.00, 74.49, 73.89, 72.76, 72.60, 71.42, 70.51, 68.38, 52.51, 52.13, 50.32, 37.14, 31.67, 29.50, 29.35, 29.07, 28.38, 27.72, 22.34, 13.05. HRMS (ESI) m/z: calc'd for $[M]^{2-}$ $C_{36}H_{39}O_{21}Cl_3N_4S_2^{2-}$: 516.0312; Found: 516.0317.

Synthesis of compound CS0S: Compound **23** (30 mg, 0.034 mmol) followed the general procedure for hydrolysis and hydrogenolysis reactions to afford **CS0S** [7 mg, 46 %, (over all three reaction)] as a white solid. ^1H NMR (400 MHz, D_2O) δ 4.46 (d, *J* = 7.9 Hz, 1H), 4.41 (d, *J* = 8.4 Hz, 1H), 4.00 (dt, *J* = 10.9, 5.2 Hz, 1H), 3.90 (t, *J* = 5.1 Hz, 1H), 3.86 – 3.83 (m, 1H), 3.82 – 3.78 (m, 1H), 3.75 – 3.69 (m, 3H), 3.66 – 3.60 (m, 2H), 3.57 (ddd, *J* = 8.8, 6.5, 2.2 Hz, 1H), 3.33 (dd, *J* = 9.4, 7.9 Hz, 1H), 3.18 (t, *J* = 5.2 Hz, 2H), 1.97 (s, 3H). ^{13}C NMR (150 MHz, D_2O) δ 174.97, 173.80, 102.09, 101.08, 79.46, 75.88, 75.21, 73.74, 72.48, 70.90, 67.69, 65.90, 61.02, 52.30, 39.36, 22.39. HRMS (ESI) m/z: calc'd for $[M+H]^+$ $C_{16}H_{29}O_{12}N_2$: 441.1720; Found: 441.1723.

Synthesis of compound CS3S: Compound **3-O-sulfated CS precursor** (30 mg, 0.031 mmol) followd the general procedure for hydrolysis and hydrogenolysis reactions to afford **CS3S** [7 mg, 42 %, (over all three reaction)] as a white solid. ^{1}H NMR (600 MHz, D_2O) δ 4.51 (d, *J* = 8.4 Hz, 1H), 4.43 (d, *J* = 7.9 Hz, 1H), 4.31 – 4.27 (m, 1H), 4.14 (d, *J* = 3.1 Hz, 1H), 3.99 – 3.92 (m, 2H), 3.87 (ddd, *J* = 11.7, 5.7, 4.0 Hz, 1H), 3.74 – 3.68 (m, 3H), 3.67 – 3.64 (m, 2H), 3.54 (t, *J* = 9.1 Hz, 1H), 3.31 (dd, *J* = 9.5, 7.9 Hz, 1H), 3.21 – 3.14 (m, 2H), 1.94 (s, 3H). ^{13}C NMR (150 MHz, D_2O) δ 174.81, 174.33, 102.10, 100.53, 79.51, 77.78, 76.36, 74.77, 73.76, 72.54, 66.18, 65.90, 60.92, 50.40, 39.40, 22.44. HRMS (ESI) m/z: calc'd for $[M+H]^-$ $C_{16}H_{27}O_{15}N_2S^-$: 519.1138; Found: 519.1142.

Synthesis of compound CS6S: Compound **26a** (20 mg, 0.034 mmol) followd the general procedure for hydrolysis and hydrogenolysis reactions to afford **CS6S** [6 mg, 50 %, (over all three reaction)] as a white solid. ^{1}H NMR (400 MHz, D_2O) δ 4.44 (dd, *J* = 8.2, 6.2 Hz, 2H), 4.18 – 4.13 (m, 2H), 4.02 – 3.96 (m, 1H), 3.92 – 3.86 (m, 3H), 3.83 (dd, *J* = 10.9, 8.4 Hz, 1H), 3.68 – 3.63 (m, 3H), 3.55 (ddd, *J* = 8.9, 5.4, 3.2 Hz, 1H), 3.33 (dd, *J* = 9.4, 7.9 Hz, 1H), 3.17 (t, *J* = 5.1 Hz, 2H), 1.96 (s, 3H). ^{13}C NMR (150 MHz, D_2O) δ 174.96, 174.07, 102.03, 101.46, 80.78, 76.37, 73.86, 72.69, 72.47, 70.73, 67.37, 67.14, 65.80, 52.10, 39.38, 22.43. HRMS (ESI) m/z: calc'd for $[M+H]^-$ $C_{16}H_{27}O_{15}N_2S^-$: 519.1138; Found: 519.1136.

Synthesis of compound CS46S: Compound **26b** (25 mg, 0.024 mmol) followed the general procedure for hydrolysis and hydrogenolysis reactions to afford **CS46S** [6 mg, 41 %, (over all three reaction)] as a white solid. ^{1}H NMR (400 MHz, D_2O) δ 4.61 – 4.58 (m, 1H), 4.55 (d, *J* = 8.0 Hz, 1H), 4.33 (dd, *J* = 11.3, 3.4 Hz, 1H), 4.29 – 4.24 (m, 1H), 4.15 – 4.06 (m, 2H), 4.01 – 3.95 (m, 1H), 3.93 – 3.89 (m, 2H), 3.77 (d, *J* = 3.2 Hz, 2H), 3.66 (ddd, *J* = 8.8, 5.5, 3.0 Hz, 1H), 3.43 (dd, *J* = 9.4, 7.9 Hz, 2H), 3.26 (t, *J* = 5.1 Hz, 2H), 2.05 (s, 3H). ^{13}C NMR (150 MHz, D_2O) δ 174.95, 173.86, 101.99, 101.56, 81.38, 76.26, 75.32, 73.98, 72.41, 72.26, 69.76, 67.74, 65.78, 52.39, 39.38, 22.44. HRMS (ESI) m/z: calc'd for $[M+H]^{2-}$ $C_{16}H_{26}O_{18}N_2S_2^{2-}$: 299.0317; Found: 299.0323.

1. **Synthesis of tripod GAG derivatives.**

General procedure for glycan conjugation on tripod:

Respective glycan (3.5 eq) and pentafluorophenol tripod **T-1** (1 eq) were dissolved in anhydrous DMF (400 µl) and triethylamine was added (3 eq per PFP active ester). The

resulting mixture was stirred at room temperature. After 5 h the reaction mixture was concentrated and purified by bond elute column (water as eluent) followed by lyophilization to afford corresponding glycan conjugated tripod.

Synthesis of 27: The general procedure for glycan conjugation on tripod was followed to afford **27** (1.1 mg, 45 %) as a white solid. ^{1}H NMR (400 MHz, D_2O) δ 4.52 (d, *J* = 8.5 Hz, 1H), 4.41 (t, *J* = 7.8 Hz, 2H), 4.27 (d, *J* = 3.2 Hz, 1H), 3.99 – 3.47 (m, 30H), 3.41 – 3.26 (m, 5H), 3.20 – 3.00 (M, 7H), 2.57 – 2.39 (m, 8H), 2.16 (q, *J* = 8.2, 7.8 Hz, 5H), 2.03 – 1.85 (m, 7H), 1.71 – 1.64 (M, 2H), 1.58 – 1.41 (m, 8H), 1.36 – 1.31 (m, 2H), 1.27 – 1.23 (m, 2H). HRMS (ESI) m/z: calc'd for $[M+H]^+$ $C_{75}H_{124}O_{44}N_8S_2$: 1906.5031; Found: 1906.5028.

Synthesis of 28: The general procedure for glycan conjugation on tripod was followed to afford **28** (1.5 mg, 47 %) as a white solid. ^{1}H NMR (400 MHz, D_2O) δ 4.55 (d, *J* = 8.4 Hz, 1H), 4.45 – 4.37 (m, 2H), 4.35 – 4.26 (m, 2H), 4.19 (dd, *J* = 17.6, 3.2 Hz, 2H), 4.06 (dd, *J* = 11.0, 8.4 Hz, 1H), 3.99 – 3.92 (m, 2H), 3.87 – 3.83 (m, 2H), 3.80 – 3.48 (m, 18H), 3.38 – 3.26 (m, 4H), 3.19 – 3.06 (m, 4H), 2.55 (t, *J* = 5.9 Hz, 2H), 2.46 – 2.38 (m, 4H), 2.19 – 2.12 (m, 3H), 2.01 – 1.85 (m, 7H), 1.71 – 1.64 (m, 2H), 1.60 – 1.42 (m, 6H), 1.37 – 1.29 (m, 2H), 1.25 – 1.20 (m, 3H). HRMS (ESI) m/z: calc'd for $[M]^{3-}$ $C_{75}H_{121}O_{53}N_8S_5^{3-}$: 713.8546; Found: 713.8541.

Synthesis of 29: The general procedure for glycan conjugation on tripod was followed to afford **29** (0.9 mg, 50 %) as a white solid. ^{1}H NMR (400 MHz, D_2O) δ 4.54 (d, *J* = 8.5 Hz, 1H), 4.46 (dd, *J* = 11.2, 8.2 Hz, 2H), 4.27 (d, *J* = 3.3 Hz, 1H), 4.16 (dd, *J* = 6.3, 3.1 Hz, 2H), 3.97 – 3.79 (m, 7H), 3.78 – 3.51 (m, 18H), 3.40 – 3.26 (m, 5H), 3.25 – 3.04 (m, 7H), 2.56 (t, *J* = 5.9 Hz, 3H), 2.47 – 2.38 (m, 4H), 2.19 – 2.11 (m, 4H), 2.02 – 1.86 (m, 7H), 1.71 – 1.64 (m, 2H), 1.59 – 1.52 (m, 3H), 1.46 (dt, *J* = 20.5, 8.1 Hz, 5H), 1.36 – 1.31 (m, 2H), 1.26 – 1.21 (m, 2H). HRMS (ESI) m/z: calc'd for $[M]^{3-}$ $C_{75}H_{121}O_{53}N_8S_5^{3-}$: 713.8546; Found: 713.8549.

Synthesis of 30: The general procedure for glycan conjugation on tripod was followed to afford **30** (1.2 mg, 52 %) as a white solid. ^{1}H NMR (400 MHz, D_2O) δ 4.60 (d, *J* = 8.1 Hz, 1H), 4.47 (d, *J* = 8.1 Hz, 2H), 4.31 – 4.13 (m, 4H), 4.04 (d, *J* = 8.2 Hz, 1H), 3.99 – 3.80 (m, 5H), 3.77 – 3.56 (m, 13H), 3.31 (t, *J* = 18.8 Hz, 4H), 3.21 – 3.06 (m, 7H), 2.60 – 2.52 (m, 3H), 2.43 (dt, *J* = 12.7, 5.6 Hz, 4H), 2.16 (q, *J* = 7.6 Hz, 4H), 1.99 – 1.88 (m, 7H), 1.68 (s, 2H), 1.58 – 1.42 (m, 7H), 1.38 – 1.26 (m, 5H). HRMS (ESI) m/z: calc'd for $[M]^{6-}$ $C_{75}H_{118}O_{62}N_8S_8^{6-}$: 396.4021; Found: 396.4027.

Synthesis of 31: The general procedure for glycan conjugation on tripod was followed to afford **31** (1.2 mg, 56 %) as a white solid. ^{1}H NMR (400 MHz, D_2O) δ 5.34 – 5.29 (m, 2H), 4.52 (d, *J* = 7.7 Hz, 1H), 4.38 (d, *J* = 9.2 Hz, 2H), 4.27 (d, *J* = 8.9 Hz, 2H), 4.10 (t, *J* = 12.6 Hz, 3H), 3.91 (d, *J* = 11.2 Hz, 1H), 3.88 – 3.75 (m, 7H), 3.73 – 3.57 (m, 14H), 3.54 – 3.46 (m, 2H), 3.29 – 3.06 (m, 9H), 2.40 (d, *J* = 11.5 Hz, 3H), 2.19 – 2.11 (m, 3H), 1.96 (d, *J* = 4.5 Hz, 6H), 1.93 – 1.89 (m, 1H), 1.77 – 1.64 (m, 4H), 1.60 – 1.39 (m, 6H), 1.37 – 1.31 (m, 2H), 1.29 – 1.22 (m, 2H).). HRMS (ESI) m/z: calc'd for $[M]^{6-}$ $C_{120}H_{187}O_{95}N_{11}S_8^{6-}$: 593.1329; Found: 593.1338.

Synthesis of 32: The general procedure for glycan conjugation on tripod was followed to afford **32** (0.95 mg, 51 %) as a white solid.^{1}H NMR (600 MHz, D_2O) δ 5.37 (d, *J* = 11.1 Hz, 2H), 4.62 (d, *J* = 8.1 Hz, 1H), 4.55 – 4.48 (m, 1H), 4.46 – 4.40 (m, 1H), 4.21 – 4.12 (m, 1H), 4.11 – 3.98 (m, 4H), 3.97 – 3.74 (m, 15H), 3.68 (q, *J* = 13.7, 12.0 Hz, 9H), 3.44 – 3.38 (m, 1H), 3.28 (ddd, *J* = 37.7, 18.8, 10.3 Hz, 4H), 3.18 (dt, *J* = 14.0, 7.0 Hz, 3H), 2.66 – 2.60 (m, 2H), 2.51 – 2.45 (m, 3H), 2.28 – 2.19 (m, 3H), 2.02 (d, *J* = 6.8 Hz, 6H), 1.83 – 1.72 (m, 3H), 1.62 (dq, *J* = 19.3, 6.4, 5.6 Hz, 3H), 1.53 (dt, *J* = 29.4, 6.3 Hz, 4H), 1.45 – 1.37 (m, 2H), 1.35 – 1.28 (m, 2H). HRMS (ESI) m/z: calc'd for $[M]^{6-}$ $C_{120}H_{187}O_{95}N_{11}S_8^{6-}$: 593.1329; Found: 593.1335.

Synthesis of 33: The general procedure for glycan conjugation on tripod was followed to afford **33** (1.1 mg, 58 %) as a white solid.^{1}H NMR (400 MHz, D_2O) δ 5.31 (s, 1H), 5.28 (d, *J* = 3.6 Hz, 1H), 4.49 (d, *J* = 7.9 Hz, 1H), 4.41 (d, *J* = 7.9 Hz, 1H), 3.92 (dd, *J* = 15.3, 9.5 Hz, 2H), 3.82 (dt, *J* = 9.0, 5.1 Hz, 5H), 3.73 (dt, *J* = 20.7, 9.5 Hz, 9H), 3.67 – 3.61 (m, 8H), 3.61 – 3.53 (m, 6H), 3.46 – 3.40 (m, 2H), 3.33 – 3.08 (m, 9H), 2.40 (t, *J* = 5.7 Hz, 3H), 2.15 (dt, *J* = 11.5, 6.7 Hz, 2H), 1.97 (d, *J* = 2.7 Hz, 6H), 1.93 – 1.88 (m, 1H), 1.78 – 1.70 (m, 4H), 1.59 – 1.52 (m, 2H), 1.51 – 1.39 (m, 3H), 1.38 – 1.29 (m, 2H), 1.28 – 1.21 (m, 3H). HRMS (ESI) m/z: calc'd for $[M]^{6-}$ $C_{120}H_{194}O_{77}N_{11}S_2$: 3085.1044; Found: 3085.1037.

2. **Synthesis of gold nanoparticles.**

Sphere shape gold nanoparticles were synthesized according to the previously reported procedure.[3] In detail, to the boiling solution of 300 µl of 1% chloroauric acid in 30 mL of distilled water added 300 µl of 1% citric acid was added to get 25 nm of sphere gold nanoparticles. Resulting solution was refluxed until the colour of the boiling solution changes from dark purple to the red vine. The nanoparticles solution was cooled, centrifuged, and the

pellet was washed with distilled water. The nanoparticles were characterized by HRTEM and UV-vis spectrophotometry.

3. **Synthesis of GAG-nanoprobes.**

Thiol-modified tripodal CS and HS saccharides were conjugated directly onto the surface of spherical AuNPs using a simple ligand exchange method under sterile condition. Briefly, Spherical AuNPs (1000 µg) were dispersed in PBS buffer pH 7.4 (1 mL, 0.1 M), to this tripod (**29-35** 500 µg) was added. The resulting solution was kept at 25 ºC for 24 h with constant shaking. Finally solution was centrifuged and pellet was washed three times with Mili Q water followed by purification using 3 KD cuttoff centrifugal filtration to get **Au@CS0S, Au@CS3S, Au@CS6S, Au@CS46S, Au@HS0S, Au@HS3S, Au@HS6S**. A change in zeta potential confirmed functionalization of the tripod on AuNP surface. Qunatification of antigen loading on AuNP surface was determined by a thiol detection kit (Cayman chemicals, USA).

Further **Au@CS0S, Au@CS3S, Au@CS6S, Au@CS46S, Au@HS0S, Au@HS3S, Au@HS6S** AuNPs were functionalized with fluorescent linker by adding fluorescent linker F-1 (15 µg) to solution of these AuNPs in PBS buffer pH 7.4 (1 mL, 0.1 M). The resulting solution was kept at 25 ºC for 12 h under constant shaking. Solution was centrifuged and pellet was washed three times with Mili Q water followed by purification using 3 KD cuttoff centrifugal filtration to get **AuF@CS0S, AuF@CS3S, AuF@CS6S, AuF@CS46S, AuF@HS0S, AuF@HS3S, AuF@HS6S**.

4. **Physical characterization of glyco-nanoparticles:**

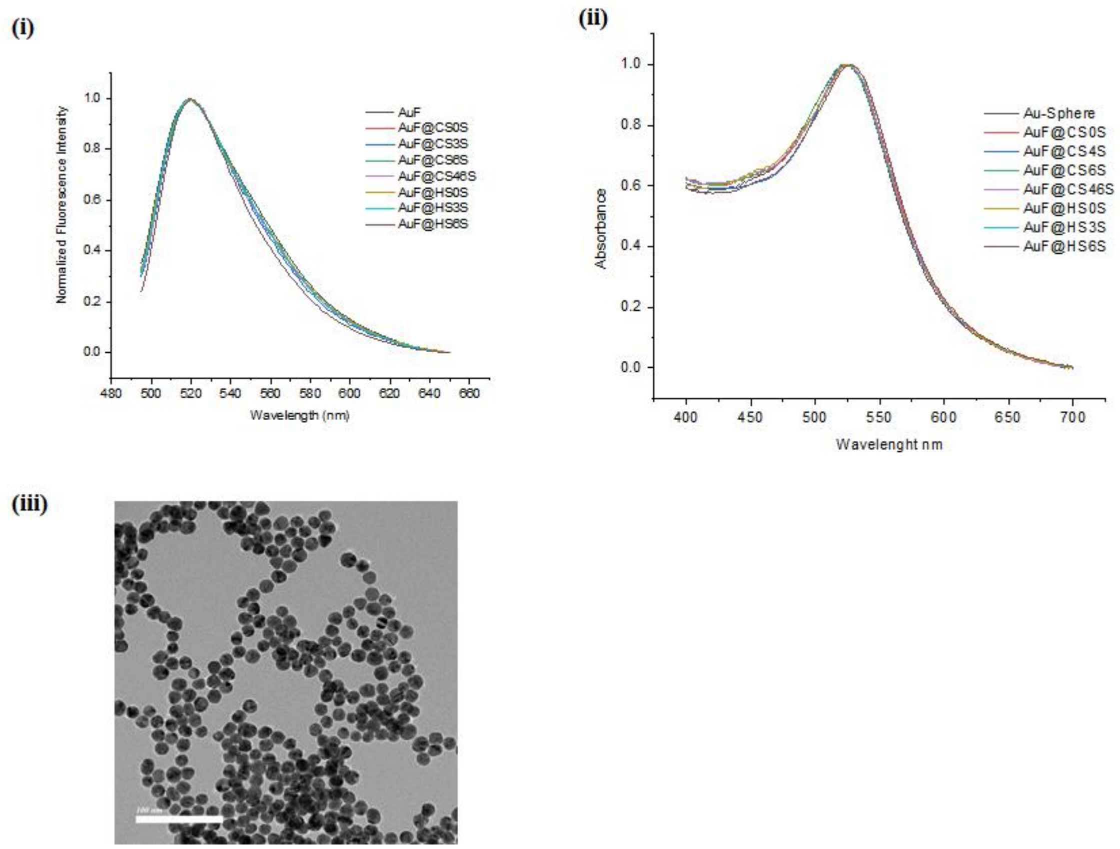

Figure S1. (i) UV-visible spectra of HS and CS-tripod functionalized fluorescent AuNPs; (ii) Fluorescence spectra of HS and CS-tripod functionalized fluorescent AuNPs; (iii) TEM image of AuNP.

Sr. No.	AuNPs	Sugar Concentration(mg/mL)	Zeta potential ζ (mv)
1	Au		-3.05
3	AuF@CS0S	0.31 ± 0.03	-8.6
5	AuF@CS3S	0.34 ± 0.04	-10.46
7	AuF@CS6S	0.35 ± 0.03	-10.3
9	AuF@CS46S	0.35 ± 0.02	-11.7
11	AuF@HS0S	0.31 ± 0.04	-8.9
13	AuF@HS3S	0.32 ± 0.05	-10.9
15	AuF@HS6S	0.31 ± 0.05	-10.8

Table S1. Quantification of sugar concentration on nanoparticle surface and zeta potential HS and CS-tripod functionalized fluorescent AuNPs

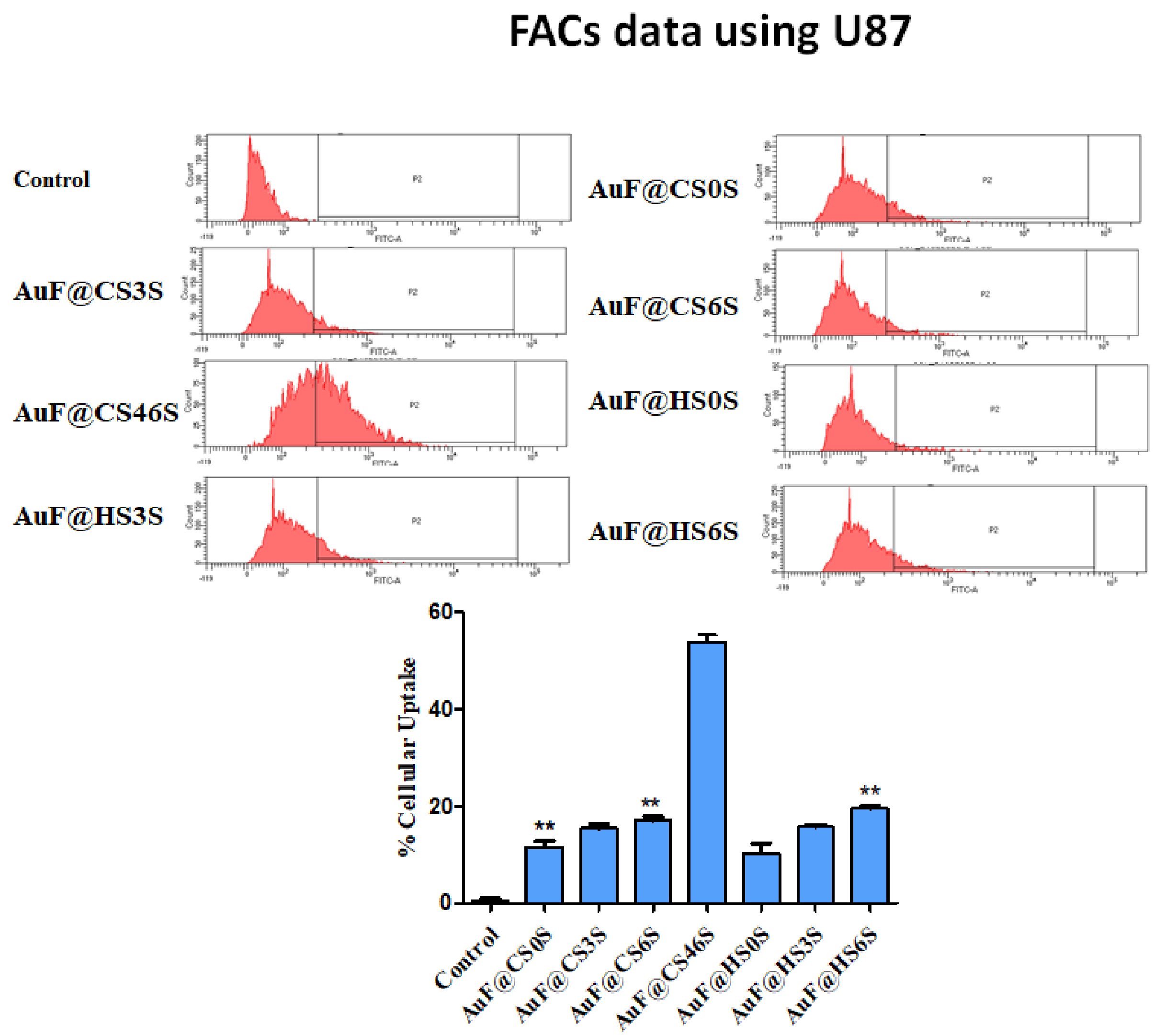

Figure S2: FACs analysis of uptake of HS and CS-tripod functionalized fluorescent AuNPs by U87 cells. Asterisks indicate statistically significant differences (**$p < 0.001$). Statistical analysis is between **Au@CS46S** and different AuNPs performed using a two-tailed Student t test.

Purity of primary cell culture. For ensuring the purity of the primary cultures and characterizing the cells, the following steps were performed:

1. Using serum-controlled culture media:

By limiting the concentration of FBS in the plating and feeding media for the cell culture, we were able to inhibit the proliferation of glial cells in the culture. The optimal FBS

concentration for culture media was determined after several iterations of testing and 2% FBS in Neurobasal complete media was found to be the most effective serum concentration for the growth of primary neurons in the culture without contamination by other cell types.

2. Characterization using neuronal markers:

The primary neuronal cultures of the olfactory bulb and hippocampus were established using pre-established protocols that were developed and tested in the LNCB at IISER Pune. The cell cultures had already been characterized using neuronal markers. The neuronal cultures were grown on sterile coverslips and fixed using 4%PFA. After fixation of the neurons, the non-specific sites were blocked using a blocking buffer consisting of normal goat serum and triton-x. Anti-MAP-2 Antibody (1:1500, ab92434), a widely used neuronal cytoskeletal marker was added to the cells and kept overnight at 4°C. Goat anti-chicken Alexa Fluor 647 was used as the secondary antibody (1:500). This antibody staining was performed specifically to visualize the morphology of neurites (Figure 1) and DAPI(4′,6-diamidino-2-phenylindole) staining was performed to visualize the cellular nuclei. In this study, we were not required to perform immuno-cytochemistry and hence the immunocharacterization process was not repeated. But the morphological differences in the isolated neurons were verified and multiple cell cultures were established before arriving at the optimal cell morphology and ensuring uncontaminated cell growth in the primary neuronal culture. The same pre-tested protocols for neuron isolation were followed for the purpose of our study and each step was performed with utmost precision. The experiments involved in our study were only performed after achieving optimal neuronal cell growth without contamination by other cell types.

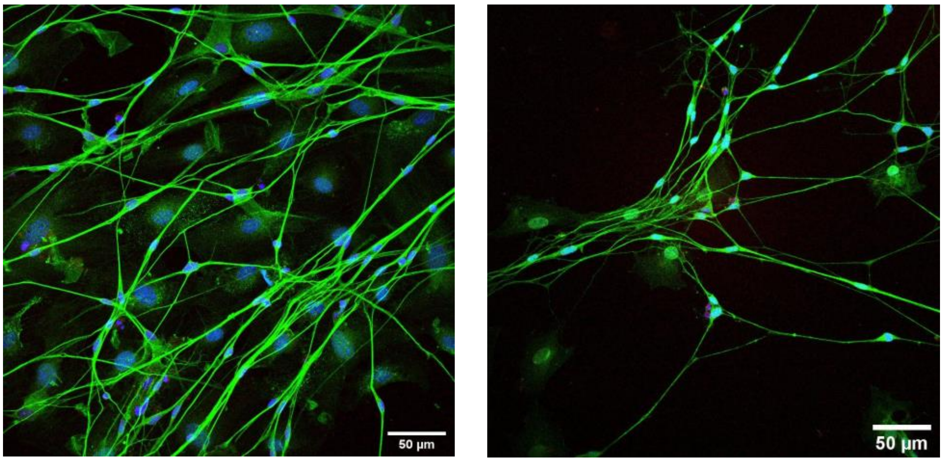

Figure S3: Olfactory bulb primary neuronal cultures stained with MAP2 antibody (green) and DAPI (blue)

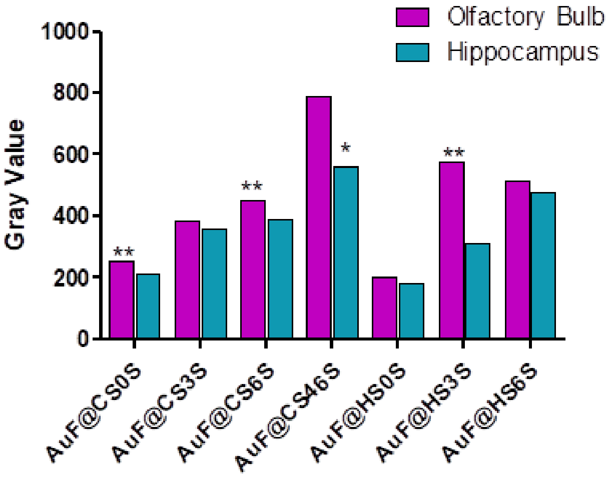

Figure S4: Plot for mean cellular uptake intensity (gray value) per neuron for HS and CS-tripod functionalized fluorescent AuNPs in olfactory bulb and hippocampus primary neural cells. Asterisks indicate statistically significant differences (**$p < 0.01$, *$p < 0.1$). Statistical analysis is between **Au@CS46S** Olfactory bulb and different AuNPs

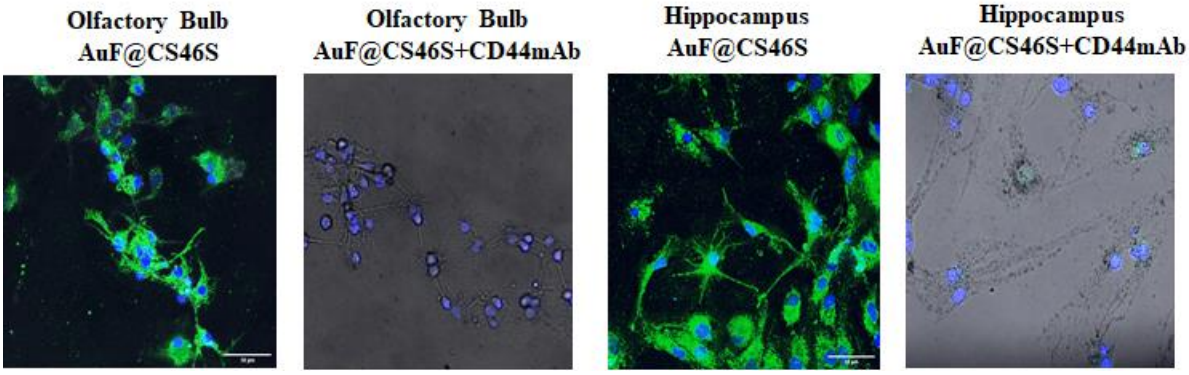

Figure S5. Confocal microscopy images of **AuF@CS46S** in the presence of CD44 monoclonal antibody in olfactory bulb and hippocampus primary neural cells (n = 3) (scale bar: 50 μm).

5. References:

1. Anand, S.; Madhukar S.; Raigawali, R.; Mohanta, N.; Jain, P.; D. shnthamurthy, C.; Gnanapraksam, B.; Kikkeri, R. Continuous-flow accelerate sulfation of heparan sulfate intermediates. *Org. Lett*. **2020**, 22, 3402-3406.
2. Toraskar, S.; Madhukar Chaudhary, P.; Kikkeri, R. The shape of nanostructures encodes immunomodulation of carbohydrate antigen and vaccine development. *ACS Chem Biol*. **2022**, 17, 1122-1130.
3. Sangabathuni, S.; Murthy, R. V.; Chaudhary, P. M.; Surve, M.; Banerjee, A.; Kikkeri, R. Glyco-gold nanoparticle shapes enhance carbohydrate-protein interactions in mammalian cells. *Nanoscale* **2016**, *8*, 12729-12735.

6. ^{1}H-NMR, ^{13}C and DEPT-135 NMR

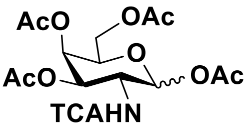

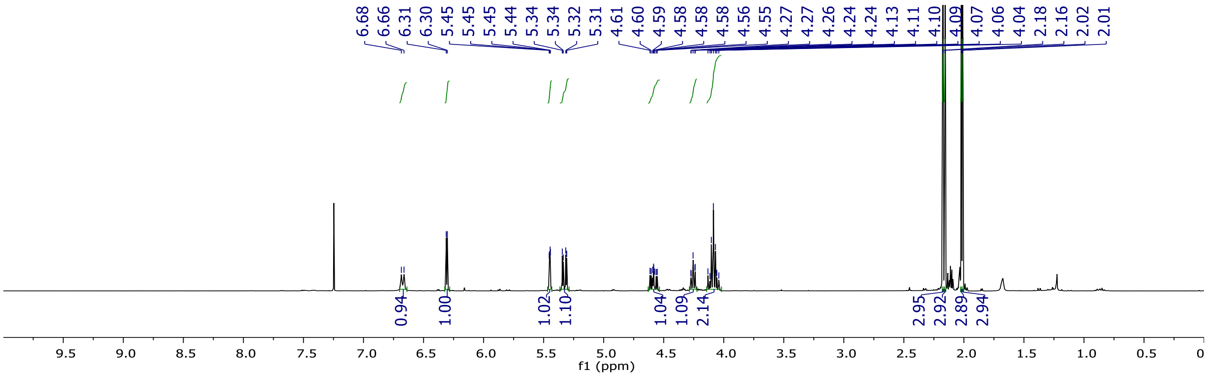

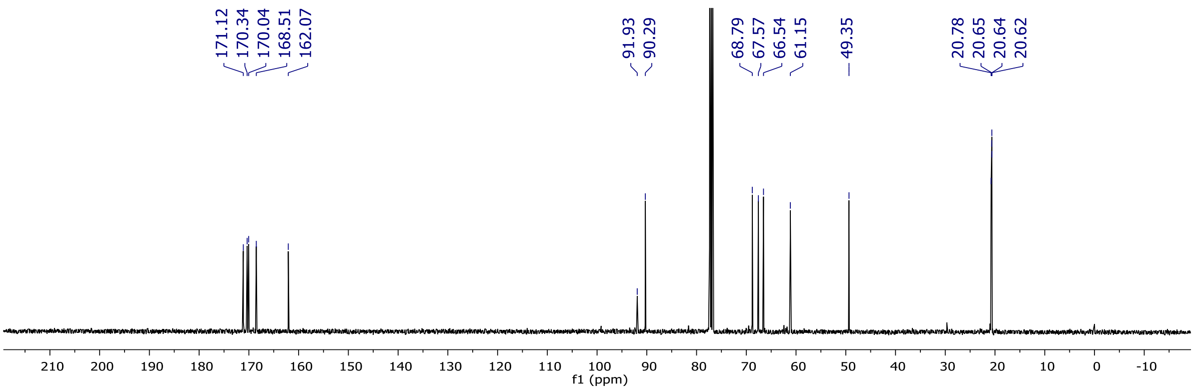

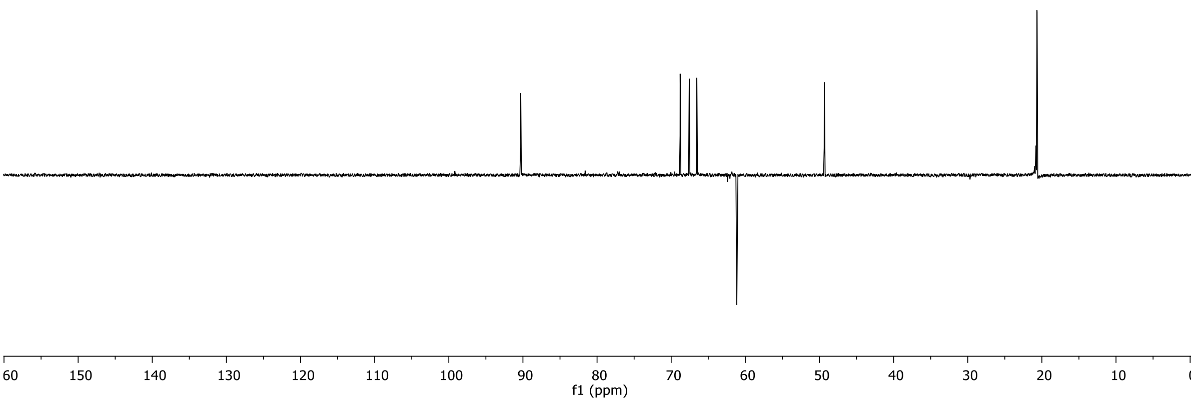

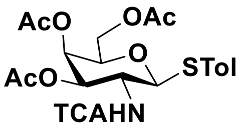
AcO
OAc
O
AcO
STol
TCAHN

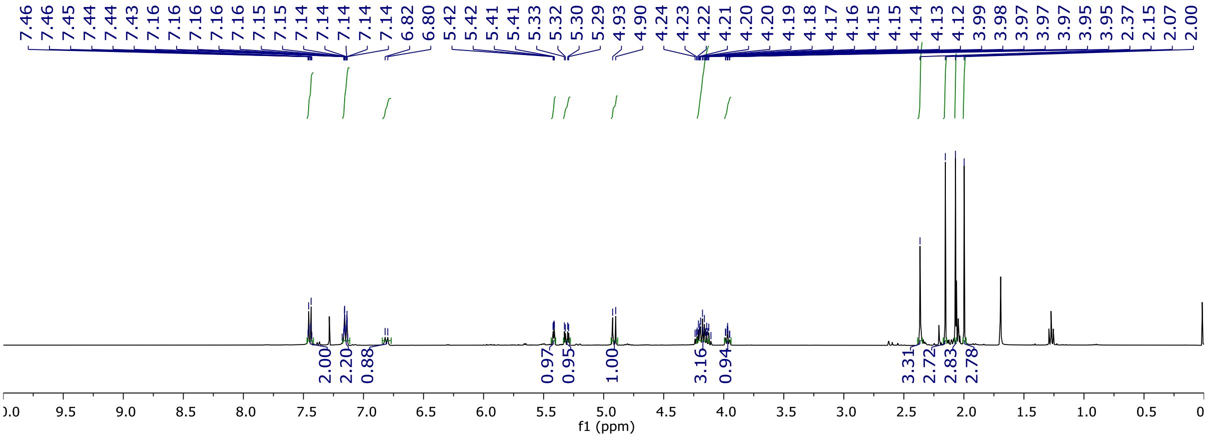
7.46 7.46 7.45 7.44 7.44 7.43 7.16 7.16 7.16 7.16 7.15 7.15 7.14 7.14 7.14 7.14 7.14 6.82 6.80 5.42 5.42 5.41 5.33 5.32 5.30 5.29 4.93 4.90 4.24 4.23 4.22 4.21 4.20 4.20 4.19 4.18 4.17 4.16 4.15 4.15 4.14 4.13 4.12 3.99 3.98 3.97 3.97 3.95 3.95 2.37 2.15 2.07 2.00
2.00 2.20 0.88 0.97 0.95 1.00 3.16 0.94 3.31 2.72 2.83 2.78
f1 (ppm)

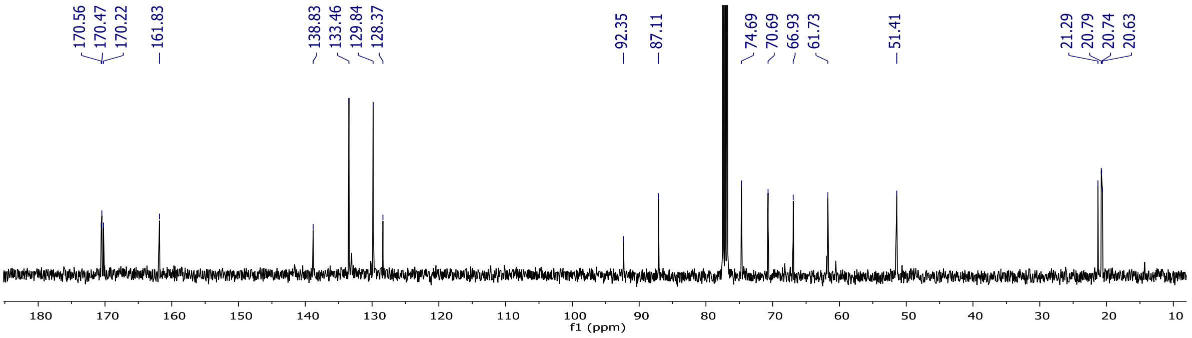
170.56 170.47 170.22 161.83 138.83 133.46 129.84 128.37 92.35 87.11 74.69 70.69 66.93 61.73 51.41 21.29 20.79 20.74 20.63
f1 (ppm)

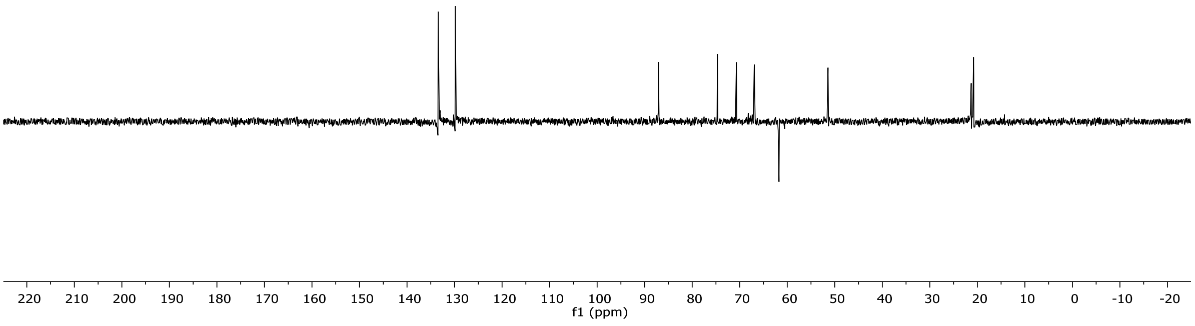
f1 (ppm)

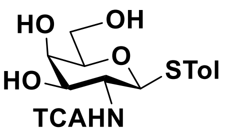
HO
OH
O
STol
HO
TCAHN

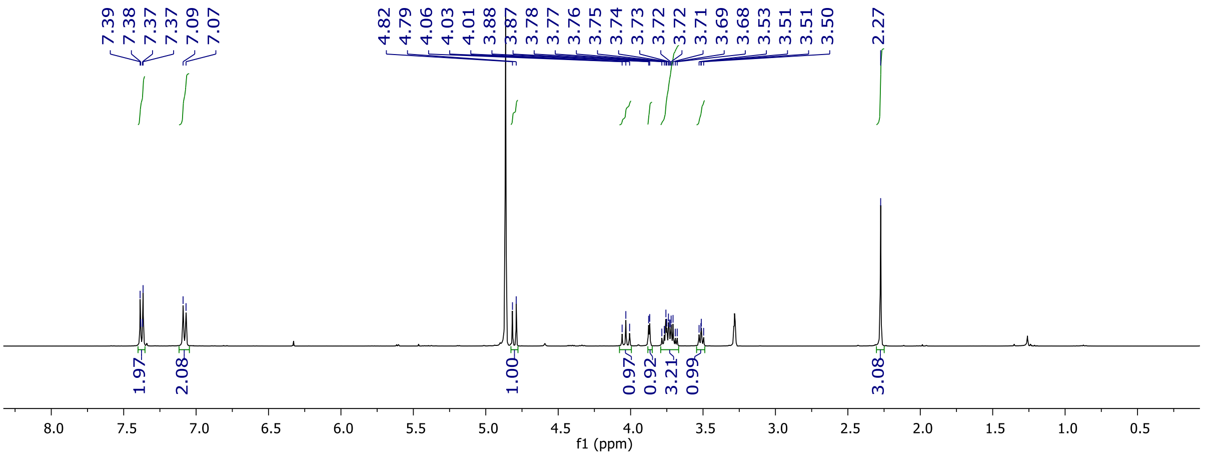
7.39
7.38
7.37
7.37
7.09
7.07
4.82
4.79
4.06
4.03
4.01
3.88
3.87
3.78
3.77
3.76
3.75
3.74
3.73
3.72
3.72
3.71
3.69
3.68
3.53
3.51
3.51
3.50
2.27
1.97
2.08
1.00
0.97
0.92
3.21
0.99
3.08
8.0 7.5 7.0 6.5 6.0 5.5 5.0 4.5 4.0 3.5 3.0 2.5 2.0 1.5 1.0 0.5
f1 (ppm)

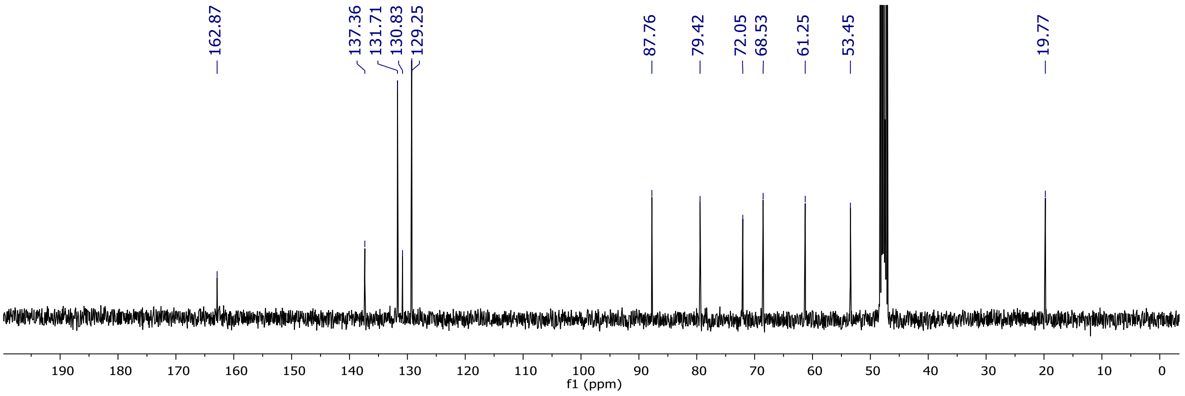
162.87
137.36
131.71
130.83
129.25
87.76
79.42
72.05
68.53
61.25
53.45
19.77
190 180 170 160 150 140 130 120 110 100 90 80 70 60 50 40 30 20 10 0
f1 (ppm)

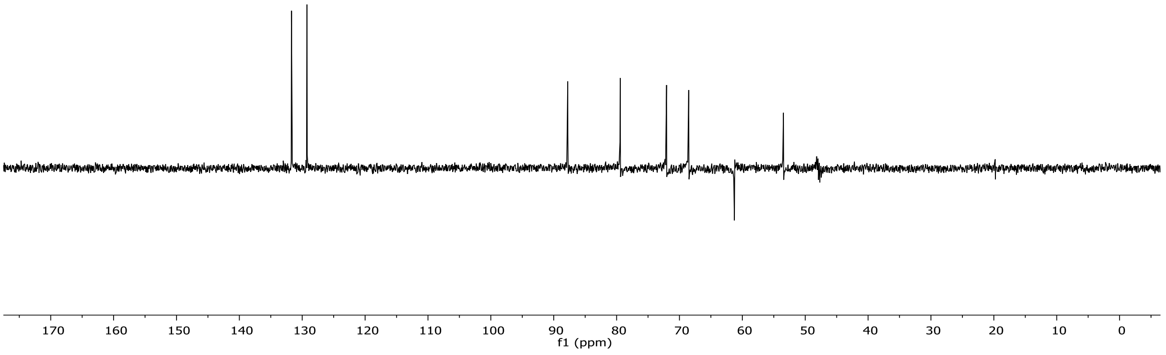
170 160 150 140 130 120 110 100 90 80 70 60 50 40 30 20 10 0
f1 (ppm)

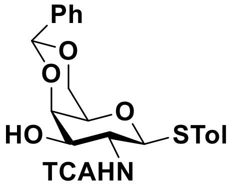
Ph
O
O
O
HO
STol
TCAHN

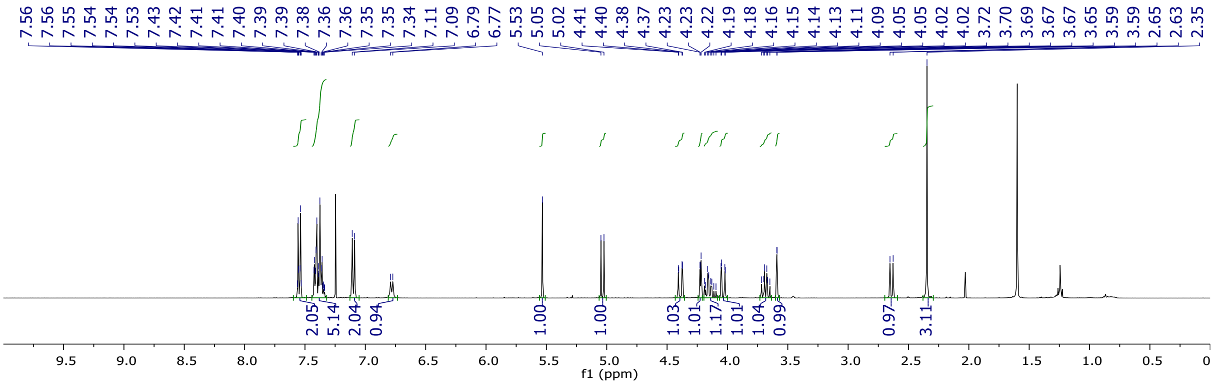
7.56 7.56 7.55 7.54 7.54 7.53 7.43 7.42 7.41 7.41 7.40 7.39 7.39 7.38 7.36 7.36 7.35 7.35 7.34 7.11 7.09 6.79 6.77 5.53 5.05 5.02 4.41 4.40 4.38 4.37 4.23 4.23 4.22 4.19 4.18 4.16 4.15 4.14 4.13 4.11 4.09 4.05 4.05 4.02 4.02 3.72 3.70 3.69 3.67 3.67 3.65 3.59 3.59 2.65 2.63 2.35
2.05 5.14 2.04 0.94 1.00 1.00 1.03 1.01 1.17 1.01 1.04 0.99 0.97 3.11
9.5 9.0 8.5 8.0 7.5 7.0 6.5 6.0 5.5 5.0 4.5 4.0 3.5 3.0 2.5 2.0 1.5 1.0 0.5 0
f1 (ppm)

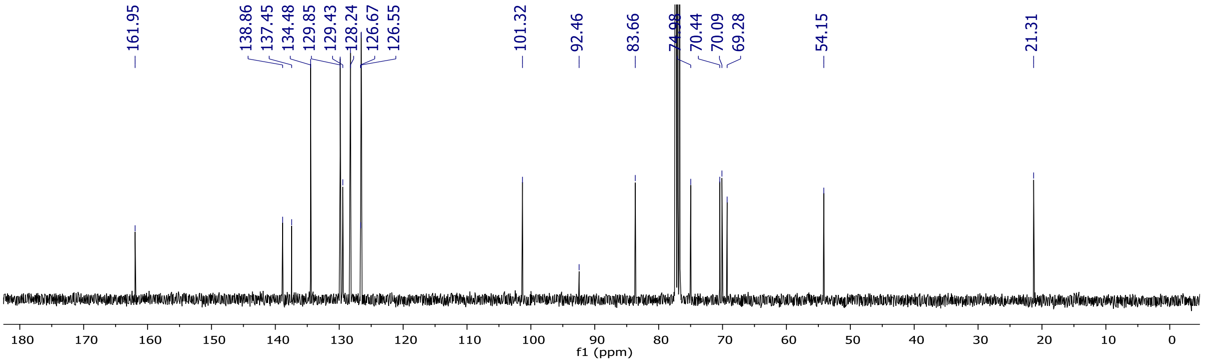
161.95
138.86 137.45 134.48 129.85 129.43 128.24 126.67 126.55
101.32
92.46
83.66
74.98
70.44 70.09 69.28
54.15
21.31
180 170 160 150 140 130 120 110 100 90 80 70 60 50 40 30 20 10 0
f1 (ppm)

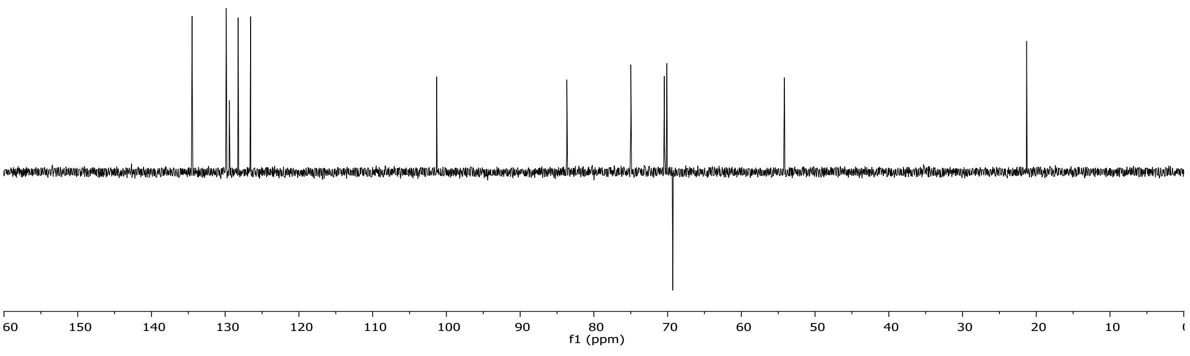
150 140 130 120 110 100 90 80 70 60 50 40 30 20 10
f1 (ppm)

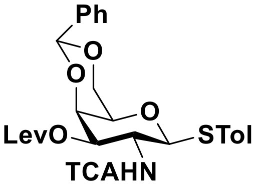
Ph
O
O
O
LevO
STol
TCAHN

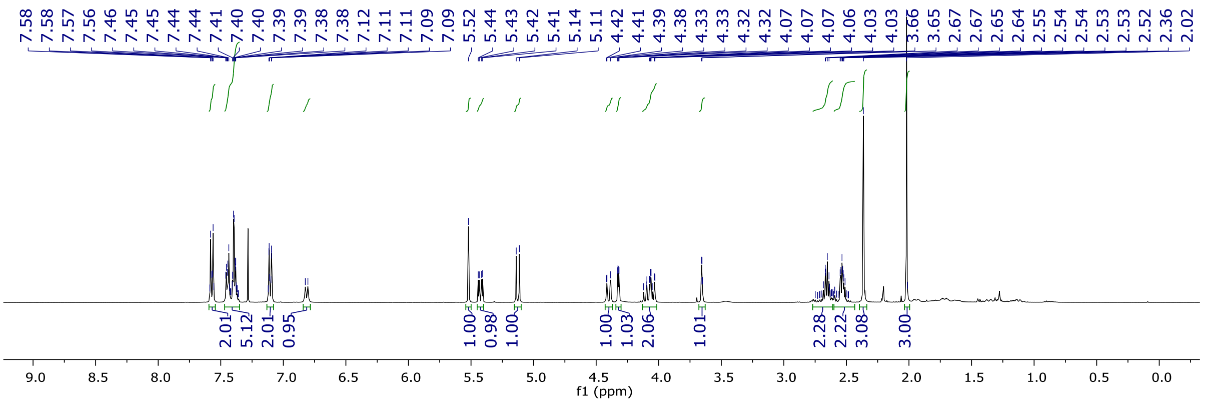
7.58 7.58 7.57 7.56 7.46 7.45 7.45 7.44 7.44 7.41 7.40 7.40 7.39 7.39 7.38 7.38 7.12 7.11 7.11 7.09 7.09 5.52 5.44 5.43 5.42 5.41 5.14 5.11 4.42 4.41 4.39 4.38 4.33 4.33 4.32 4.32 4.07 4.07 4.07 4.06 4.03 4.03 3.66 3.65 2.67 2.67 2.65 2.64 2.55 2.54 2.54 2.53 2.53 2.52 2.36 2.02
2.01 5.12 2.01 0.95 1.00 0.98 1.00 1.00 1.03 2.06 1.01 2.28 2.22 3.08 3.00
9.0 8.5 8.0 7.5 7.0 6.5 6.0 5.5 5.0 4.5 4.0 3.5 3.0 2.5 2.0 1.5 1.0 0.5 0.0
f1 (ppm)

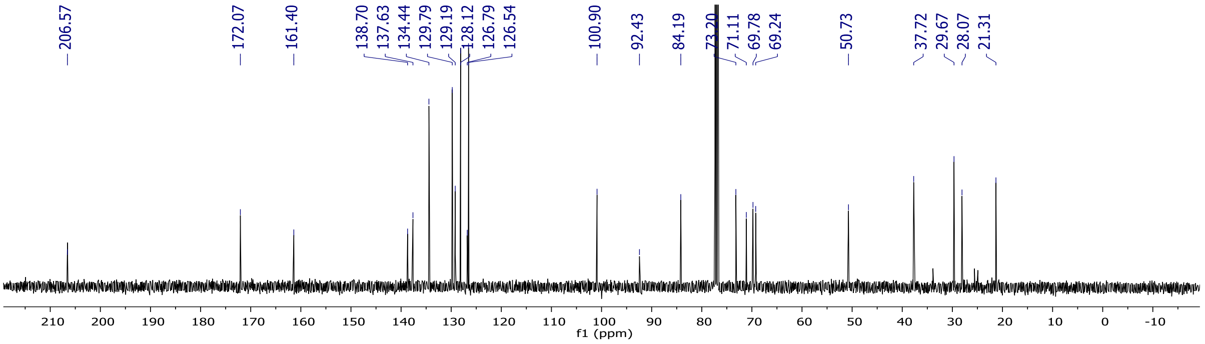
206.57 172.07 161.40 138.70 137.63 134.44 129.79 129.19 128.12 126.79 126.54 100.90 92.43 84.19 73.20 71.11 69.78 69.24 50.73 37.72 29.67 28.07 21.31
210 200 190 180 170 160 150 140 130 120 110 100 90 80 70 60 50 40 30 20 10 0 -10
f1 (ppm)

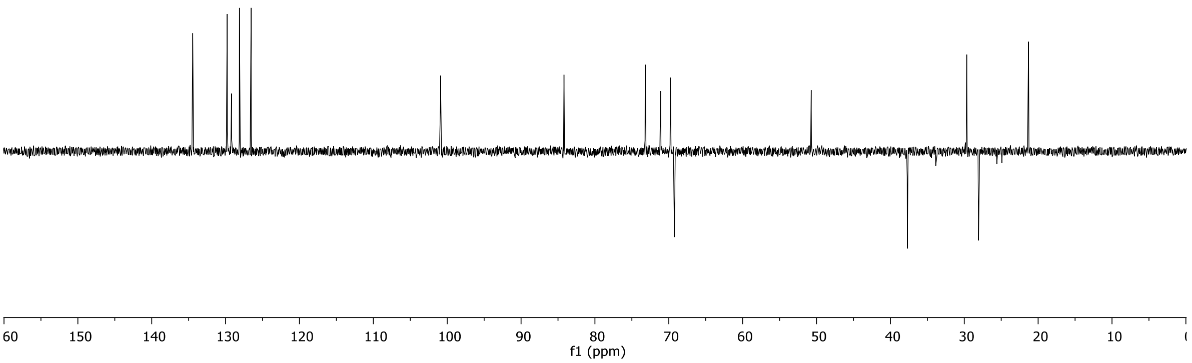
150 140 130 120 110 100 90 80 70 60 50 40 30 20 10
f1 (ppm)

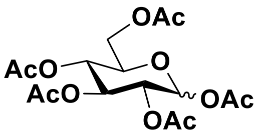
OAc
AcO
AcO
O
OAc
OAc

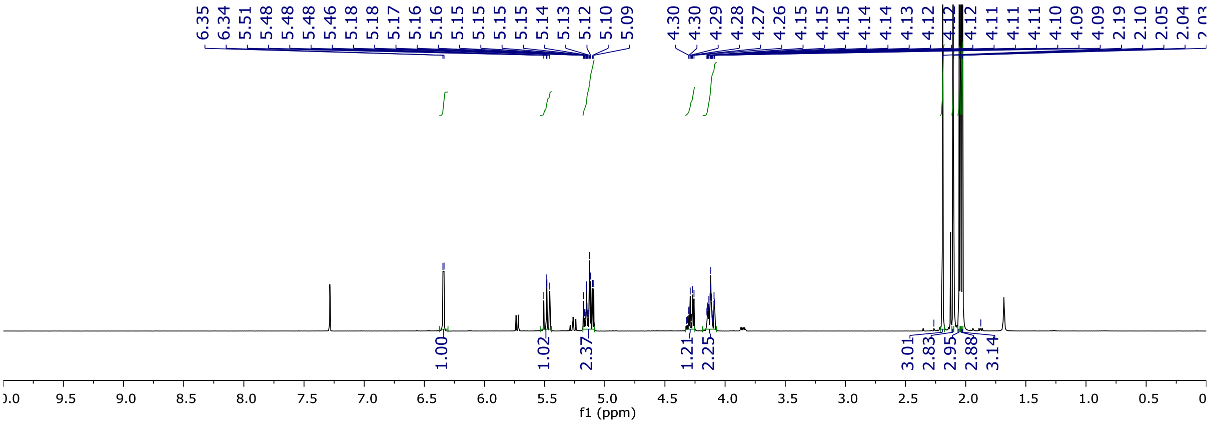
6.35 6.34 5.51 5.48 5.48 5.48 5.46 5.18 5.18 5.17 5.16 5.15 5.15 5.15 5.15 5.14 5.13 5.12 5.10 5.09 4.30 4.30 4.29 4.28 4.27 4.26 4.15 4.15 4.14 4.14 4.13 4.12 4.12 4.12 4.11 4.11 4.11 4.10 4.09 4.09 2.19 2.10 2.05 2.04
1.00 1.02 2.37 1.21 2.25 3.01 2.83 2.95 2.88 3.14
9.5 9.0 8.5 8.0 7.5 7.0 6.5 6.0 5.5 5.0 4.5 4.0 3.5 3.0 2.5 2.0 1.5 1.0 0.5
f1 (ppm)

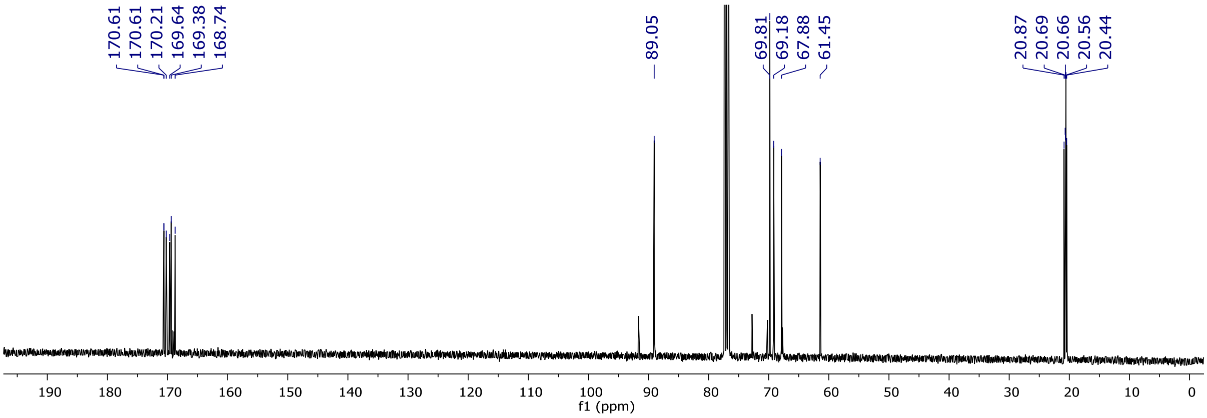
170.61 170.61 170.21 169.64 169.38 168.74
89.05
69.81 69.18 67.88 61.45
20.87 20.69 20.66 20.56 20.44
190 180 170 160 150 140 130 120 110 100 90 80 70 60 50 40 30 20 10 0
f1 (ppm)

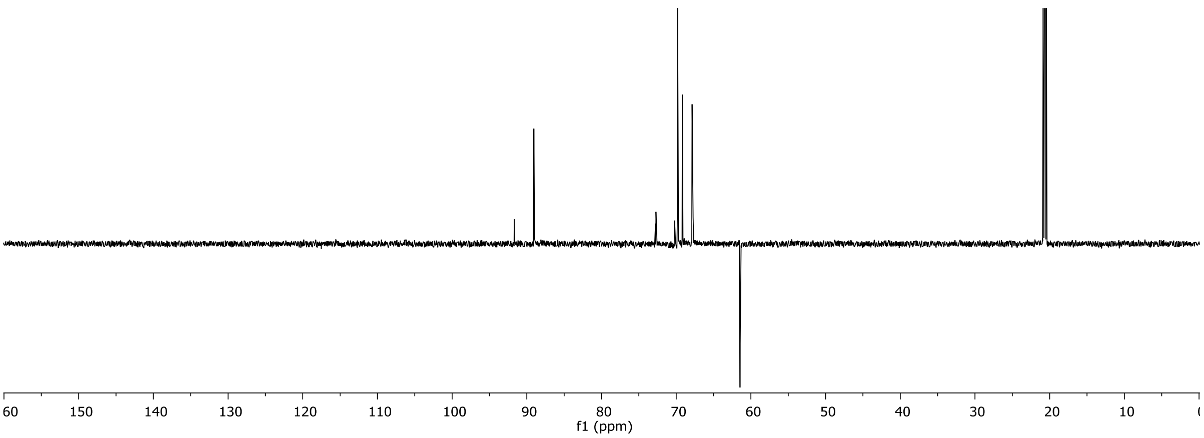
150 140 130 120 110 100 90 80 70 60 50 40 30 20 10
f1 (ppm)

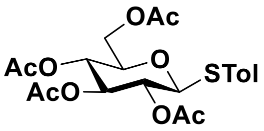
OAc
AcO
O
STol
AcO
OAc

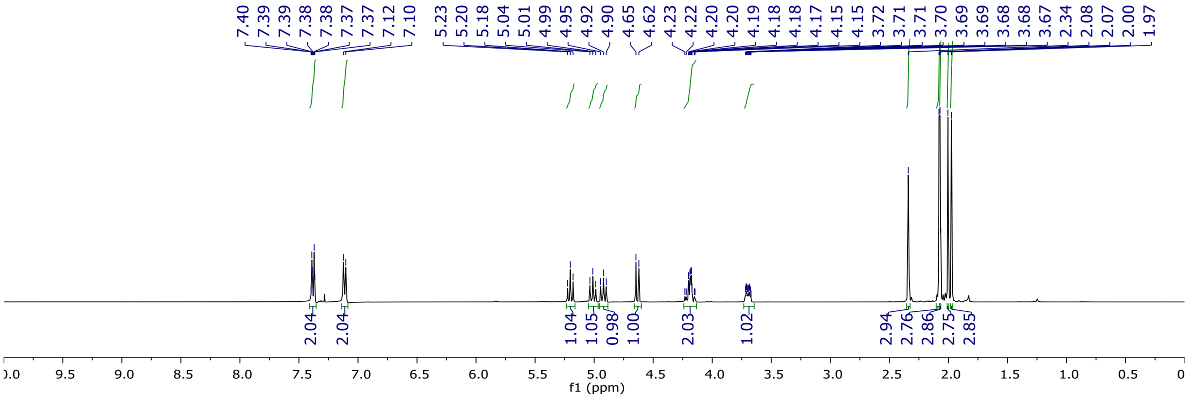
7.40 7.39 7.39 7.38 7.38 7.37 7.37 7.12 7.10 5.23 5.20 5.18 5.04 5.01 4.99 4.95 4.92 4.90 4.65 4.62 4.23 4.22 4.20 4.20 4.19 4.18 4.18 4.17 4.15 4.15 3.72 3.71 3.71 3.70 3.69 3.68 3.68 3.67 2.34 2.08 2.07 2.00 1.97
2.04 2.04 1.04 1.05 0.98 1.00 2.03 1.02 2.94 2.76 2.86 2.75 2.85
f1 (ppm)

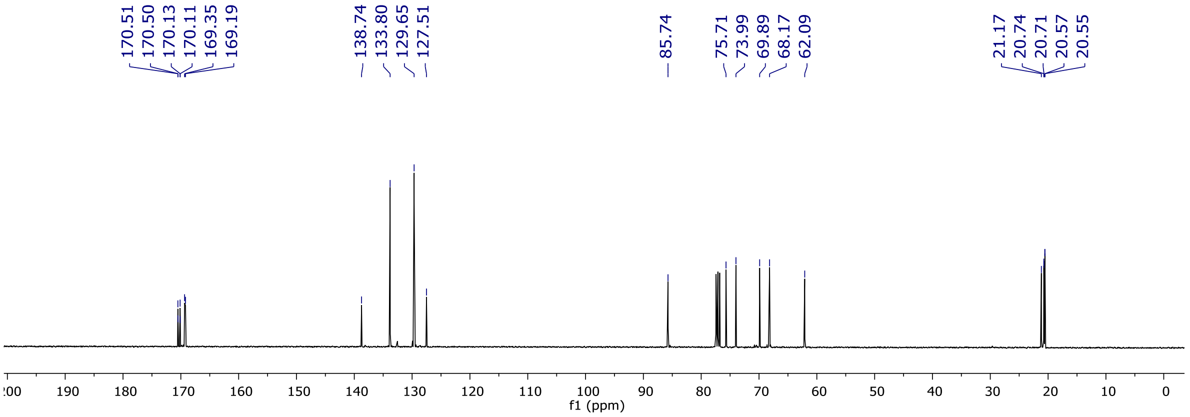
170.51 170.50 170.13 170.11 169.35 169.19 138.74 133.80 129.65 127.51 85.74 75.71 73.99 69.89 68.17 62.09 21.17 20.74 20.71 20.57 20.55
f1 (ppm)

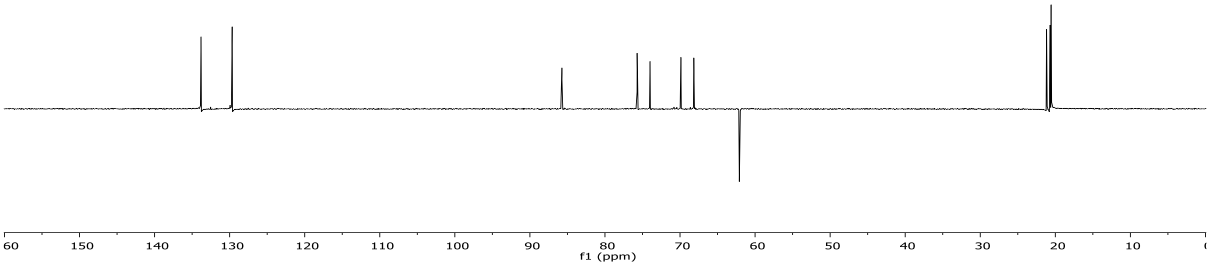
f1 (ppm)

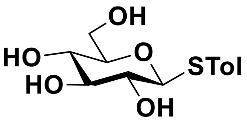
OH
HO
O
STol
HO
OH

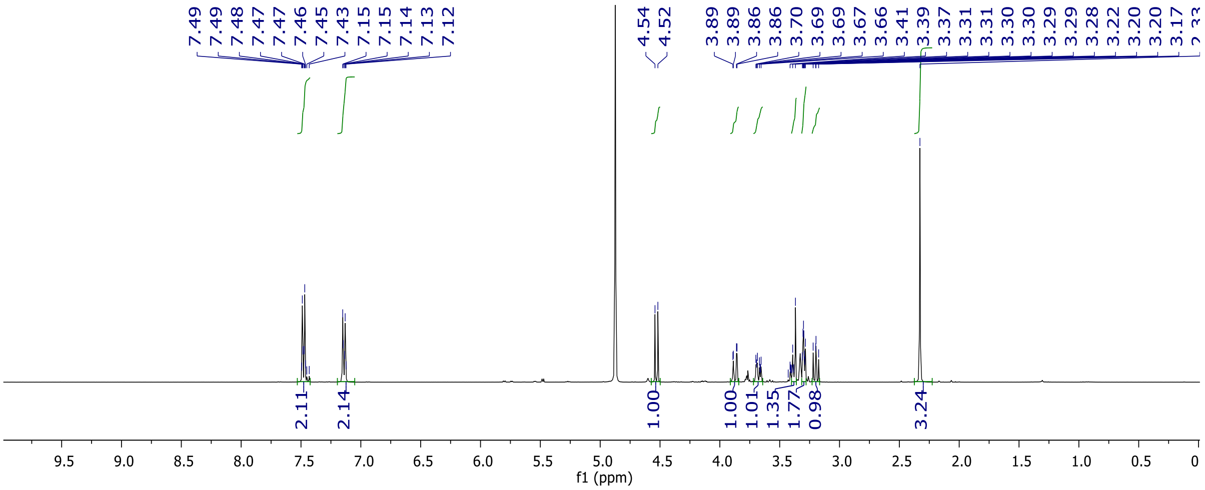
7.49 7.49 7.48 7.47 7.47 7.46 7.45 7.43 7.15 7.15 7.14 7.13 7.12
4.54 4.52
3.89 3.89 3.86 3.86 3.70 3.69 3.67 3.66 3.41 3.39 3.37 3.31 3.31 3.30 3.29 3.29 3.28 3.22 3.20 3.20 3.17
2.11 2.14 1.00 1.00 1.01 1.35 1.77 0.98 3.24
9.5 9.0 8.5 8.0 7.5 7.0 6.5 6.0 5.5 5.0 4.5 4.0 3.5 3.0 2.5 2.0 1.5 1.0 0.5 0
f1 (ppm)

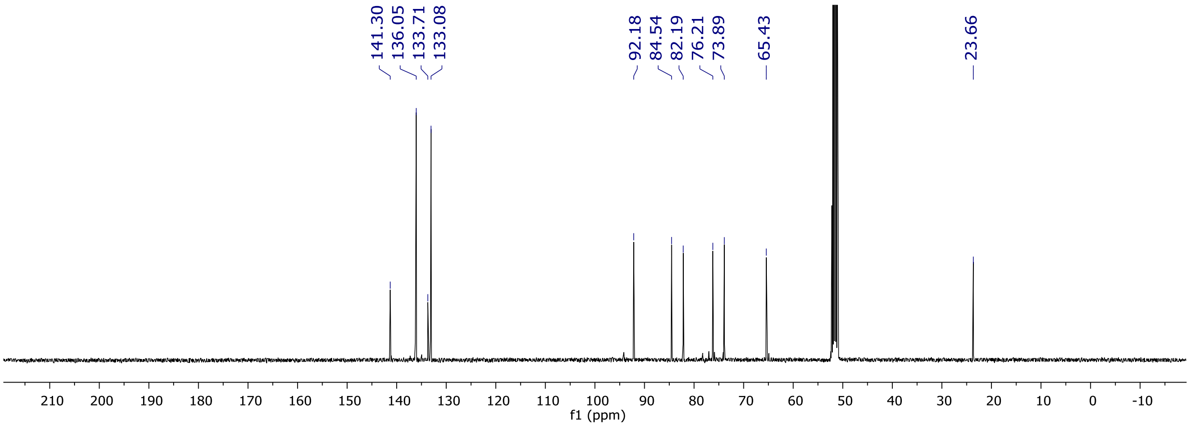
141.30 136.05 133.71 133.08
92.18 84.54 82.19 76.21 73.89
65.43
23.66
210 200 190 180 170 160 150 140 130 120 110 100 90 80 70 60 50 40 30 20 10 0 -10
f1 (ppm)

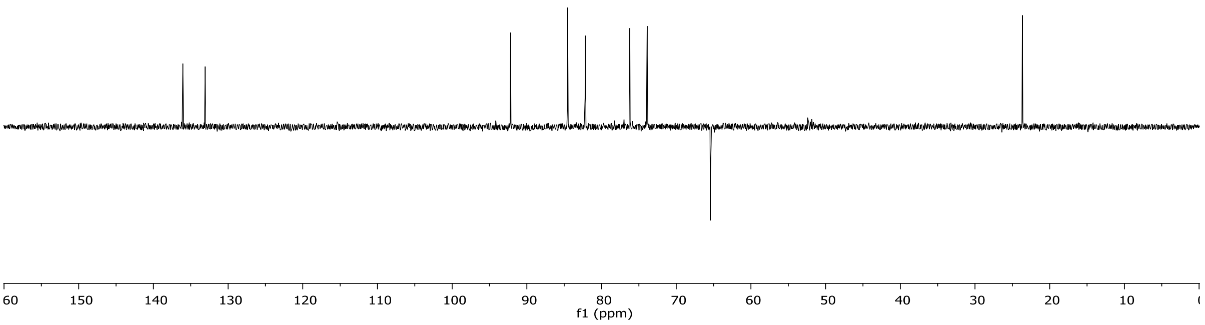
150 140 130 120 110 100 90 80 70 60 50 40 30 20 10
f1 (ppm)

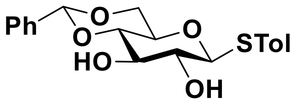
Ph
O
O
O
HO
OH
STol

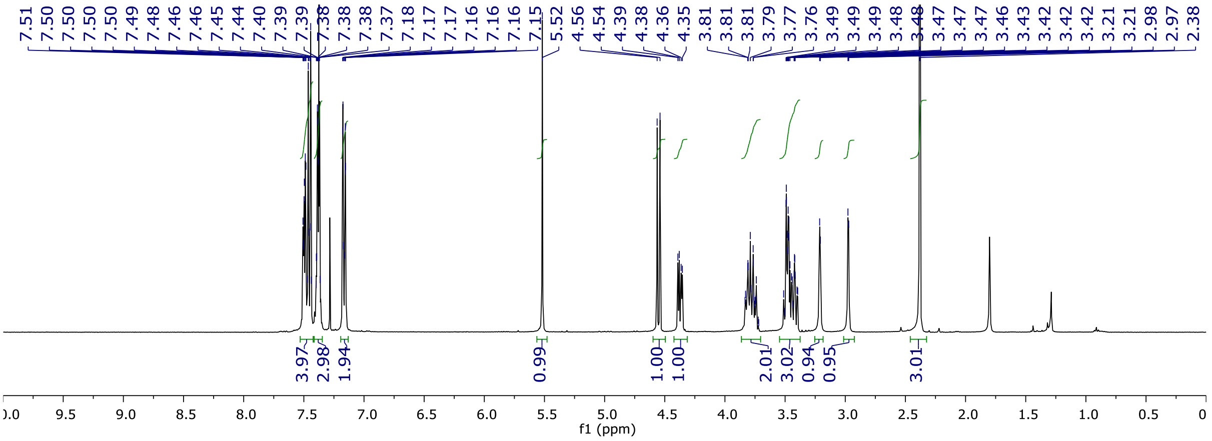
7.51
7.50
7.50
7.50
7.50
7.49
7.48
7.46
7.46
7.45
7.44
7.40
7.39
7.39
7.38
7.38
7.38
7.37
7.18
7.17
7.17
7.16
7.16
7.15
5.52
4.56
4.54
4.39
4.38
4.36
4.35
3.81
3.81
3.81
3.79
3.77
3.76
3.49
3.49
3.49
3.48
3.48
3.47
3.47
3.47
3.46
3.43
3.42
3.42
3.42
3.21
3.21
2.98
2.97
2.38
3.97
2.98
1.94
0.99
1.00
1.00
2.01
3.02
0.94
0.95
3.01
f1 (ppm)

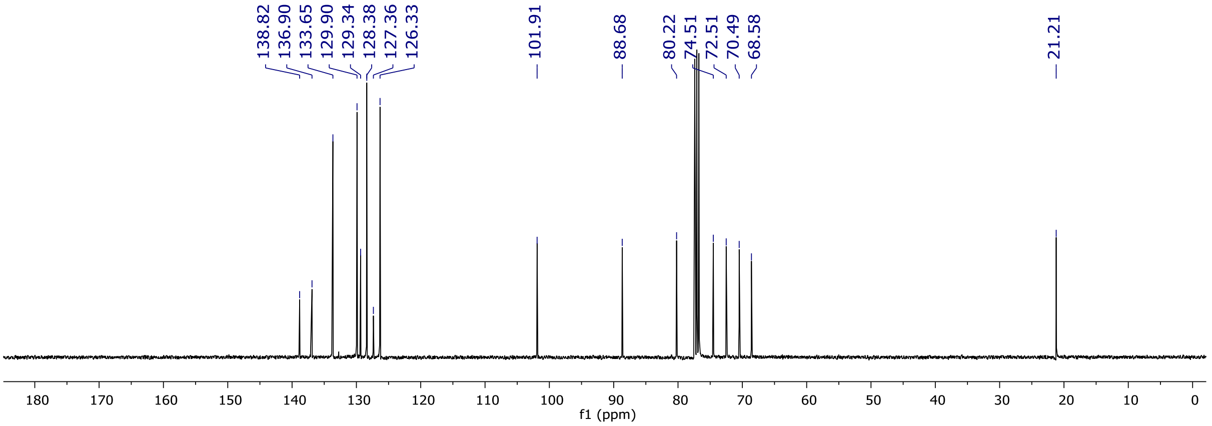
138.82
136.90
133.65
129.90
129.34
128.38
127.36
126.33
101.91
88.68
80.22
74.51
72.51
70.49
68.58
21.21
f1 (ppm)

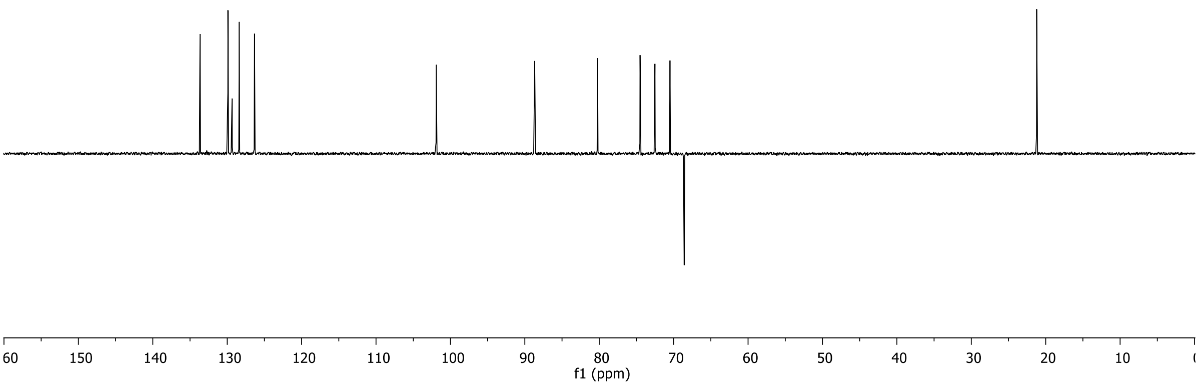
f1 (ppm)

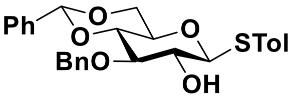
Ph
O
O
O
STol
BnO
OH

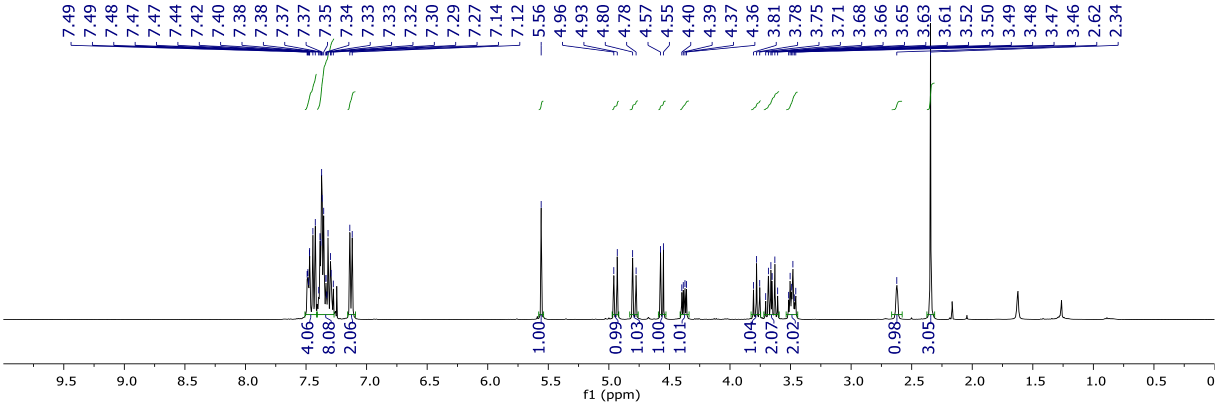
7.49 7.49 7.48 7.47 7.47 7.44 7.42 7.40 7.38 7.38 7.37 7.37 7.35 7.34 7.33 7.33 7.32 7.30 7.29 7.27 7.14 7.12 5.56 4.96 4.93 4.80 4.78 4.57 4.55 4.40 4.39 4.37 4.36 3.81 3.78 3.75 3.71 3.68 3.66 3.65 3.63 3.61 3.52 3.50 3.49 3.48 3.47 3.46 2.62 2.34
4.06 8.08 2.06 1.00 0.99 1.03 1.00 1.01 1.04 2.07 2.02 0.98 3.05
9.5 9.0 8.5 8.0 7.5 7.0 6.5 6.0 5.5 5.0 4.5 4.0 3.5 3.0 2.5 2.0 1.5 1.0 0.5 0
f1 (ppm)

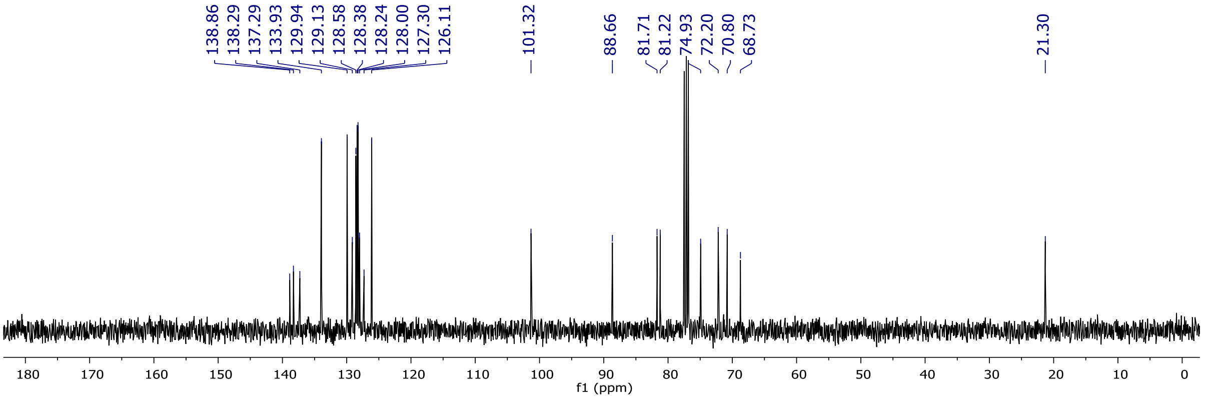
138.86 138.29 137.29 133.93 129.94 129.13 128.58 128.38 128.24 128.00 127.30 126.11 101.32 88.66 81.71 81.22 74.93 72.20 70.80 68.73 21.30
180 170 160 150 140 130 120 110 100 90 80 70 60 50 40 30 20 10 0
f1 (ppm)

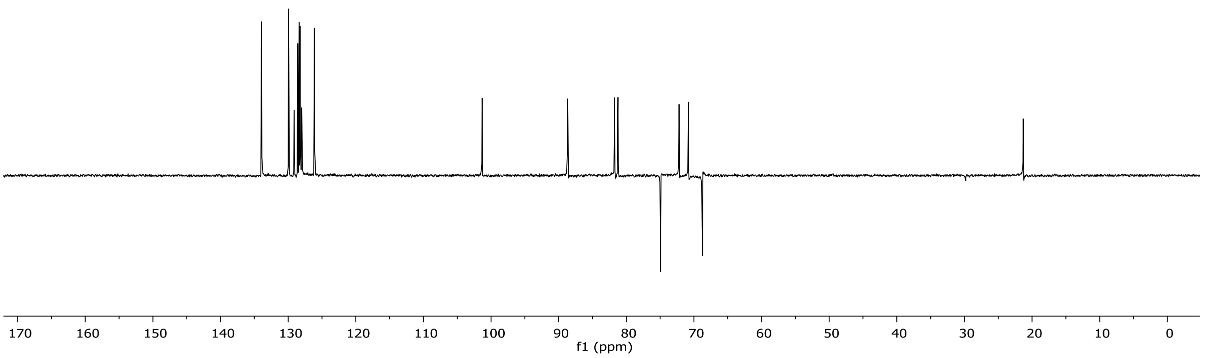
170 160 150 140 130 120 110 100 90 80 70 60 50 40 30 20 10 0
f1 (ppm)

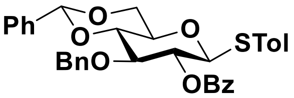
Ph
O
O
O
BnO
STol
OBz

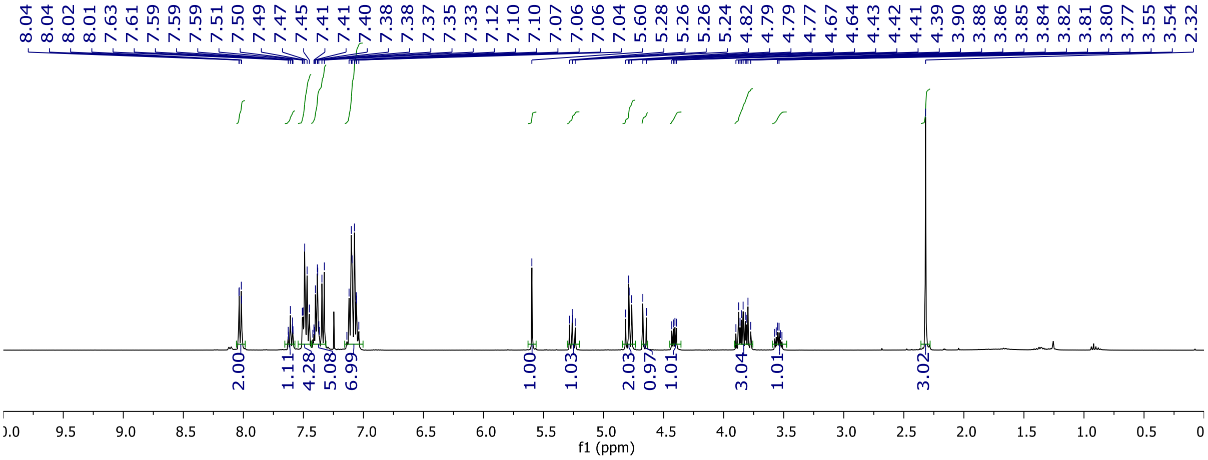
8.04 8.04 8.02 8.01 7.63 7.61 7.59 7.59 7.59 7.51 7.50 7.49 7.47 7.45 7.41 7.41 7.40 7.38 7.38 7.37 7.35 7.33 7.12 7.10 7.10 7.07 7.06 7.06 7.04 5.60 5.28 5.26 5.26 5.24 4.82 4.79 4.79 4.77 4.67 4.64 4.43 4.42 4.41 4.39 3.90 3.88 3.86 3.85 3.84 3.82 3.81 3.80 3.77 3.55 3.54 2.32
2.00 1.11 4.28 5.08 6.99 1.00 1.03 2.03 0.97 1.01 3.04 1.01 3.02
f1 (ppm)

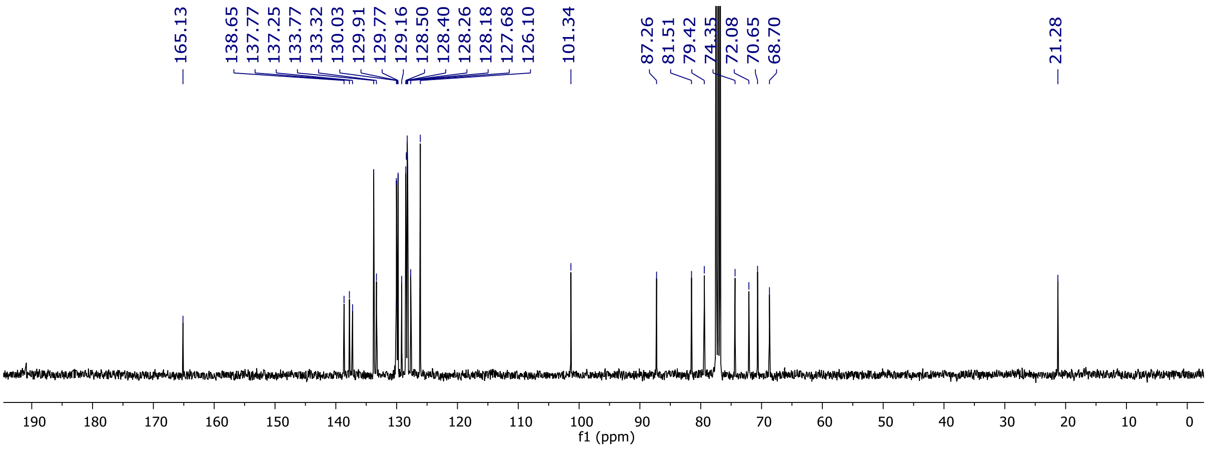
165.13
138.65 137.77 137.25 133.77 133.32 130.03 129.91 129.77 129.16 128.50 128.40 128.26 128.18 127.68 126.10
101.34
87.26 81.51 79.42 74.35 72.08 70.65 68.70
21.28
f1 (ppm)

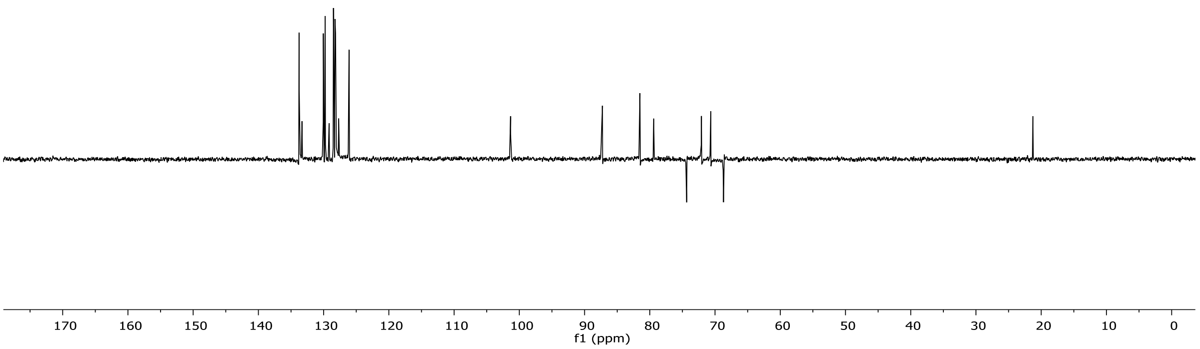
f1 (ppm)

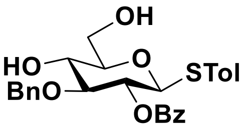
OH
HO
O
STol
BnO
OBz

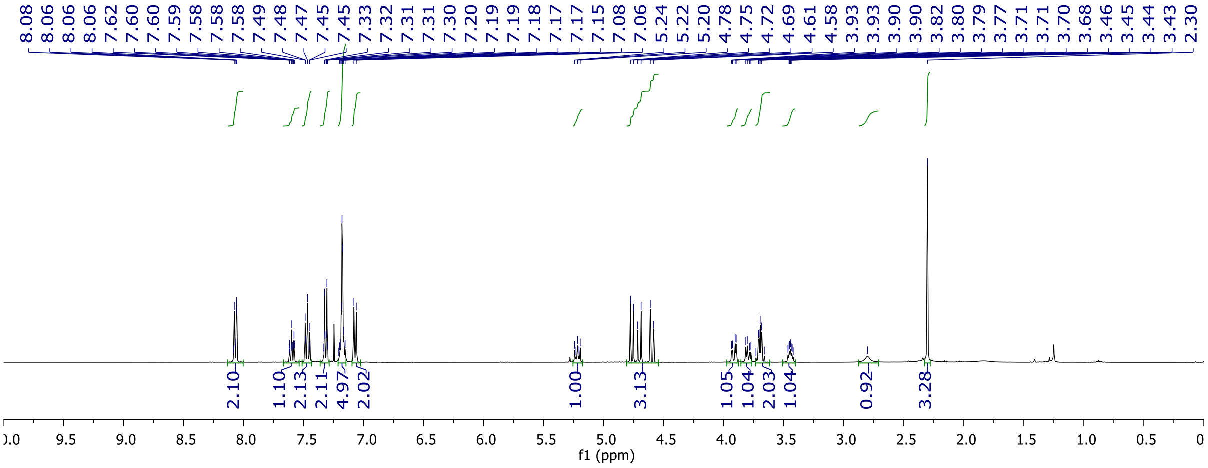
8.08 8.06 8.06 8.06 7.62 7.60 7.60 7.59 7.58 7.58 7.58 7.49 7.48 7.47 7.45 7.45 7.33 7.32 7.31 7.31 7.30 7.20 7.19 7.19 7.18 7.17 7.17 7.15 7.08 7.06 5.24 5.22 5.20 4.78 4.75 4.72 4.69 4.61 4.58 3.93 3.93 3.90 3.90 3.82 3.80 3.79 3.77 3.71 3.71 3.70 3.68 3.46 3.45 3.44 3.43 2.30
2.10
1.10
2.13
2.11
4.97
2.02
1.00
3.13
1.05
1.04
2.03
1.04
0.92
3.28
f1 (ppm)

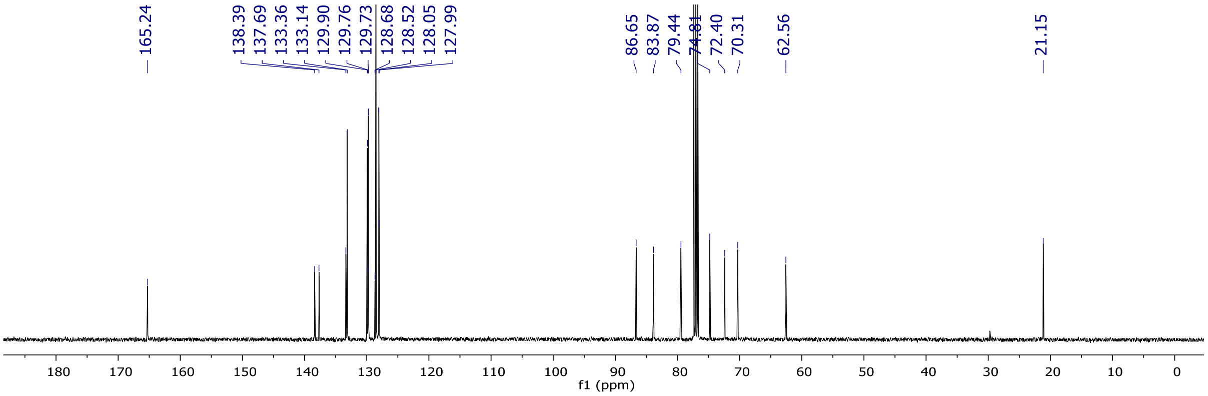
165.24
138.39 137.69 133.36 133.14 129.90 129.76 129.73 128.68 128.52 128.05 127.99
86.65 83.87 79.44 74.81 72.40 70.31
62.56
21.15
f1 (ppm)

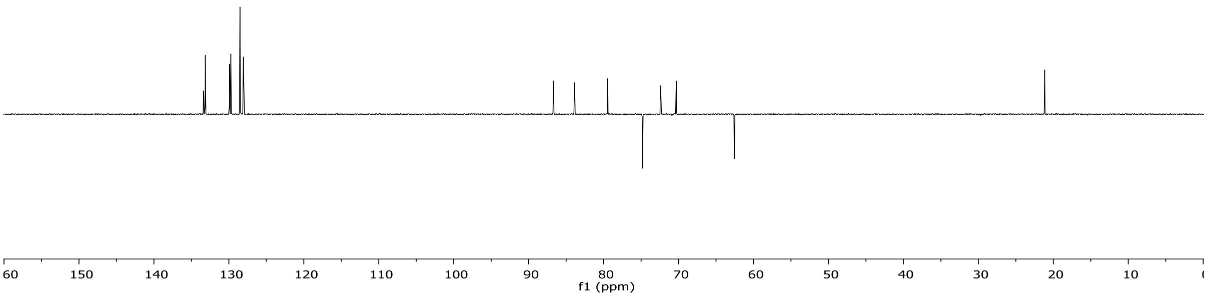
f1 (ppm)

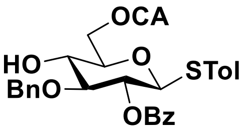
OCA
HO
O
STol
BnO
OBz

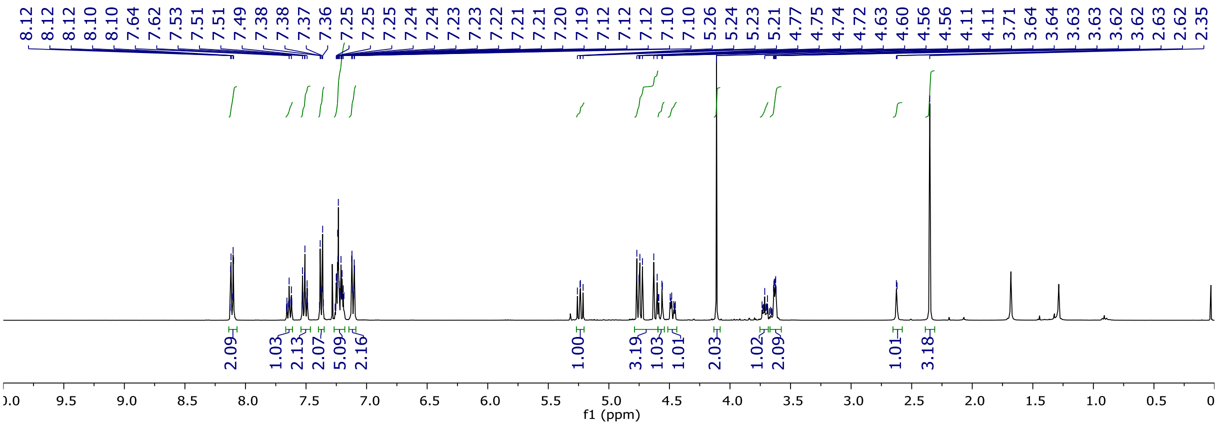
8.12 8.12 8.12 8.10 8.10 7.64 7.62 7.53 7.51 7.51 7.49 7.38 7.38 7.37 7.36 7.25 7.25 7.25 7.24 7.24 7.23 7.23 7.22 7.21 7.21 7.20 7.19 7.12 7.12 7.12 7.10 7.10 5.26 5.24 5.23 5.21 4.77 4.75 4.74 4.72 4.63 4.60 4.56 4.56 4.11 4.11 3.71 3.64 3.64 3.63 3.63 3.62 2.63 2.62 2.35
2.09 1.03 2.13 2.07 5.09 2.16 1.00 3.19 1.03 1.01 2.03 1.02 2.09 1.01 3.18
f1 (ppm)

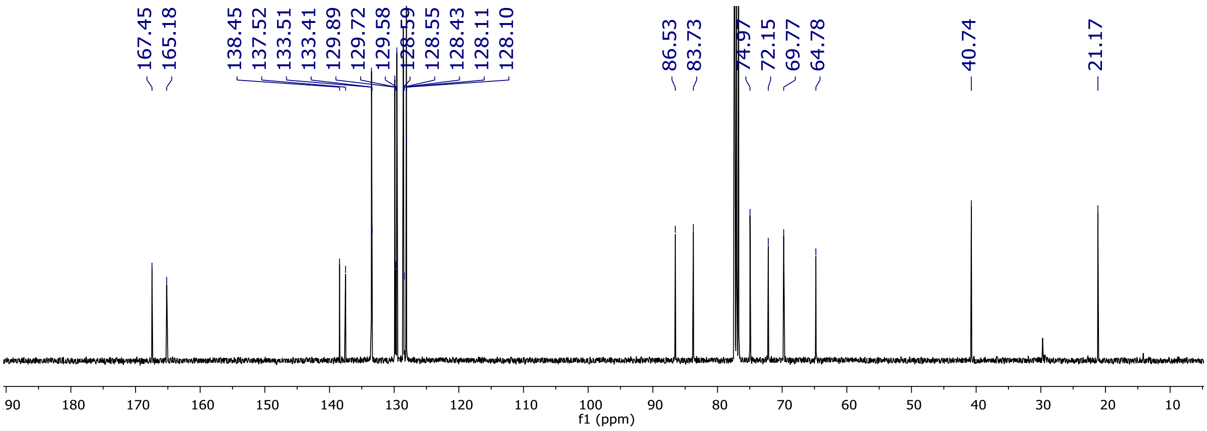
167.45 165.18 138.45 137.52 133.51 133.41 129.89 129.72 129.58 128.59 128.55 128.43 128.11 128.10 86.53 83.73 74.97 72.15 69.77 64.78 40.74 21.17
f1 (ppm)

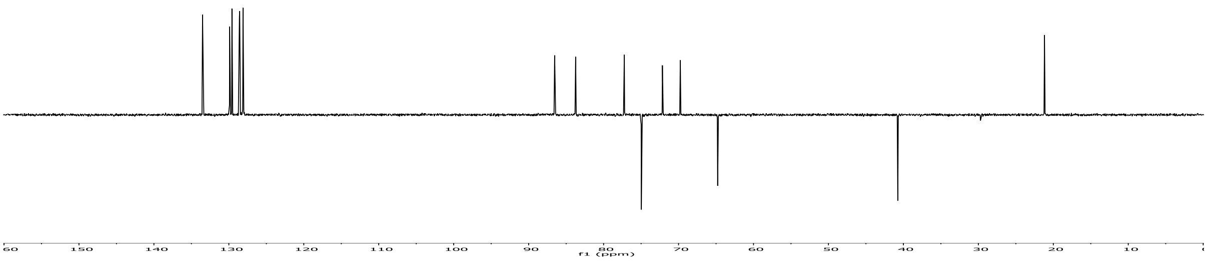

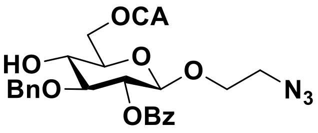
OCA
HO
O
O
BnO
OBz
N3

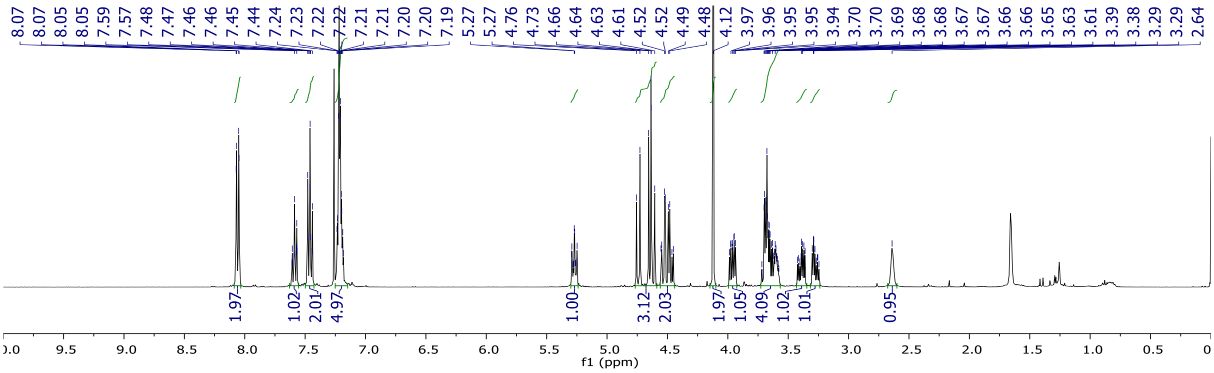
8.07 8.07 8.05 8.05 7.59 7.57 7.48 7.47 7.46 7.46 7.45 7.44 7.24 7.23 7.22 7.22 7.21 7.21 7.20 7.20 7.19 5.27 5.27 4.76 4.73 4.66 4.64 4.63 4.61 4.52 4.52 4.49 4.48 4.12 3.97 3.96 3.95 3.95 3.94 3.70 3.70 3.69 3.68 3.68 3.67 3.67 3.66 3.66 3.65 3.63 3.61 3.39 3.38 3.29 3.29 2.64
1.97 1.02 2.01 4.97 1.00 3.12 2.03 1.97 1.05 4.09 1.02 1.01 0.95
f1 (ppm)

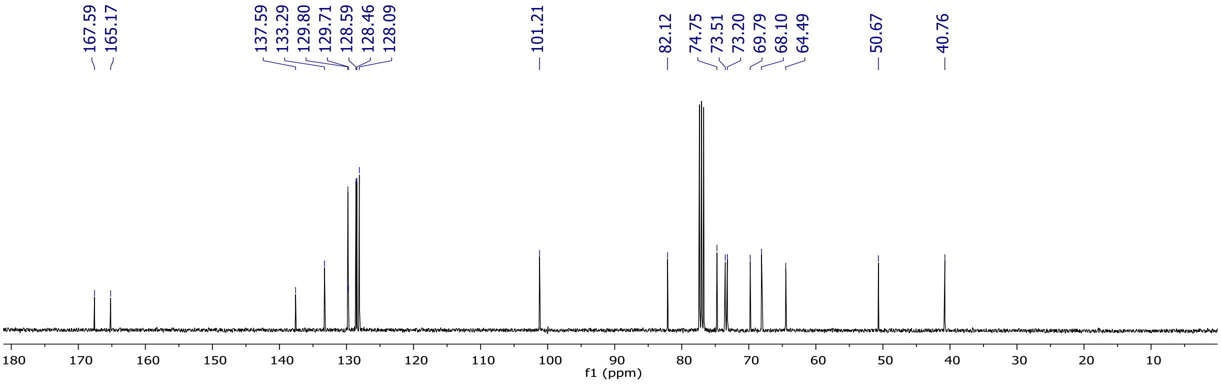
167.59 165.17 137.59 133.29 129.80 129.71 128.59 128.46 128.09 101.21 82.12 74.75 73.51 73.20 69.79 68.10 64.49 50.67 40.76
f1 (ppm)

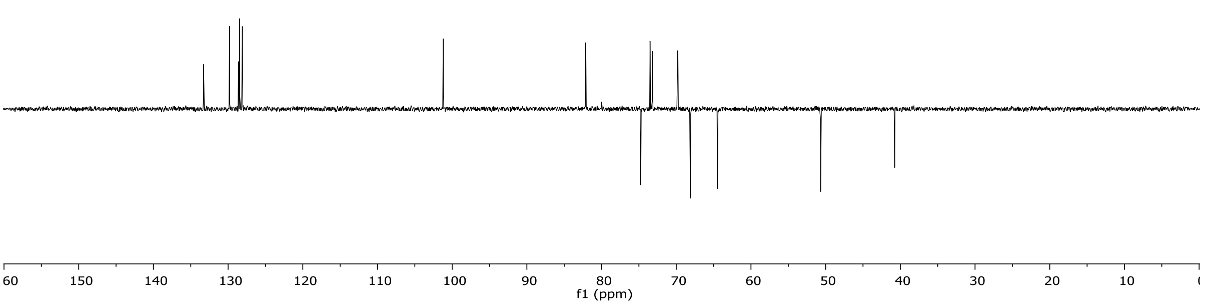
f1 (ppm)

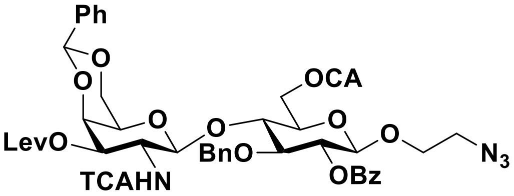
Ph
O
O
O
O
OCA
O
O
LevO
BnO
N3
TCAHN
OBz

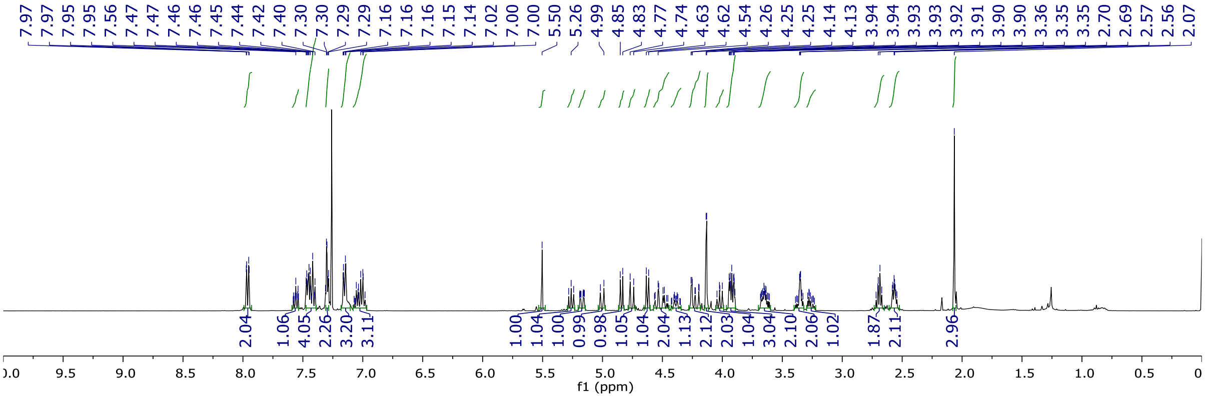
7.97 7.97 7.95 7.95 7.56 7.47 7.46 7.46 7.45 7.44 7.42 7.40 7.30 7.30 7.29 7.29 7.16 7.16 7.15 7.14 7.02 7.00 7.00 5.50 5.26 4.99 4.85 4.83 4.77 4.74 4.63 4.62 4.54 4.26 4.25 4.25 4.14 4.13 3.94 3.94 3.93 3.93 3.92 3.91 3.90 3.90 3.36 3.35 2.70 2.69 2.57 2.56 2.07
2.04 1.06 4.05 2.26 3.20 3.11 1.00 1.04 1.00 0.99 0.98 1.05 1.04 2.04 1.13 2.12 2.03 1.04 3.04 2.10 2.06 1.02 1.87 2.11 2.96
f1 (ppm)

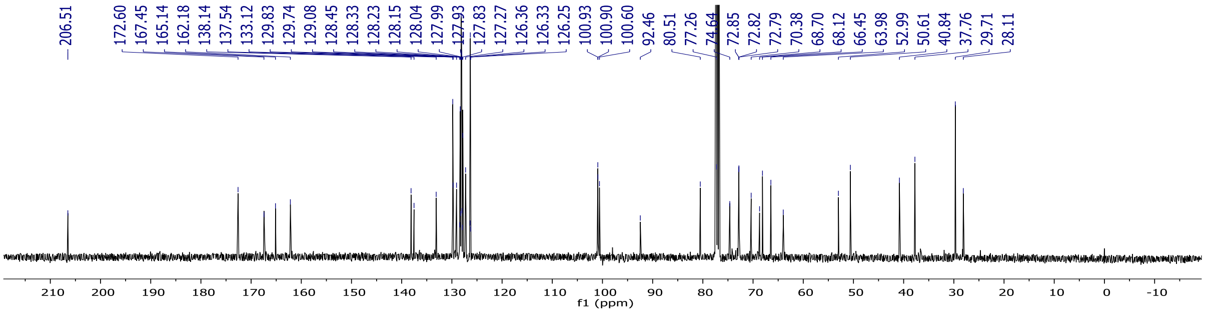
206.51 172.60 167.45 165.14 162.18 138.14 137.54 133.12 129.83 129.74 129.08 128.45 128.33 128.23 128.15 128.04 127.99 127.93 127.83 127.27 126.36 126.33 126.25 100.93 100.90 100.60 92.46 80.51 77.26 74.64 72.85 72.82 72.79 70.38 68.70 68.12 66.45 63.98 52.99 50.61 40.84 37.76 29.71 28.11
f1 (ppm)

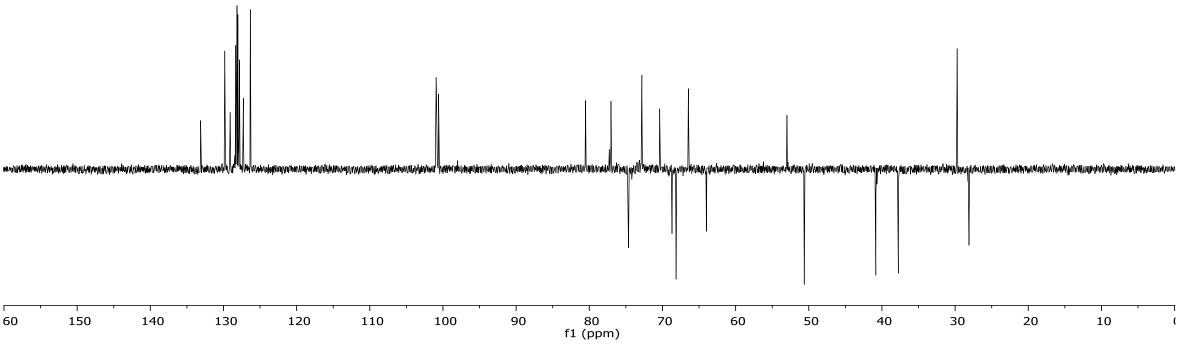
f1 (ppm)

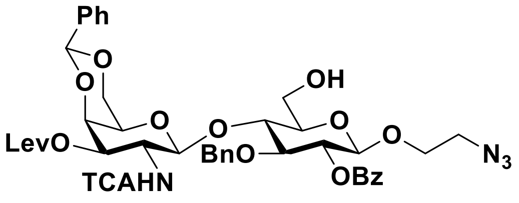
Ph
O
O
OH
LevO
O
O
O
O
BnO
N3
TCAHN
OBz

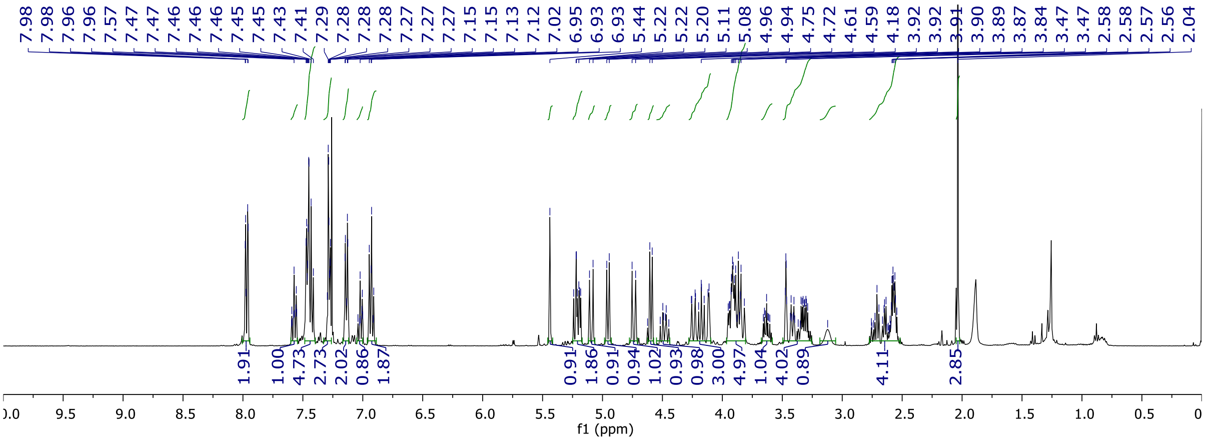
7.98 7.98 7.96 7.96 7.57 7.47 7.47 7.46 7.46 7.45 7.45 7.43 7.41 7.29 7.28 7.28 7.27 7.27 7.27 7.15 7.13 7.12 7.02 6.95 6.93 6.93 5.44 5.22 5.22 5.20 5.11 5.08 4.96 4.94 4.75 4.72 4.61 4.59 4.18 3.92 3.92 3.91 3.90 3.89 3.87 3.84 3.47 3.47 2.58 2.58 2.57 2.56 2.04
1.91 1.00 4.73 2.73 2.02 0.86 1.87 0.91 1.86 0.91 0.94 1.02 0.93 0.98 3.00 4.97 1.04 4.02 0.89 4.11 2.85
9.5 9.0 8.5 8.0 7.5 7.0 6.5 6.0 5.5 5.0 4.5 4.0 3.5 3.0 2.5 2.0 1.5 1.0 0.5 0
f1 (ppm)

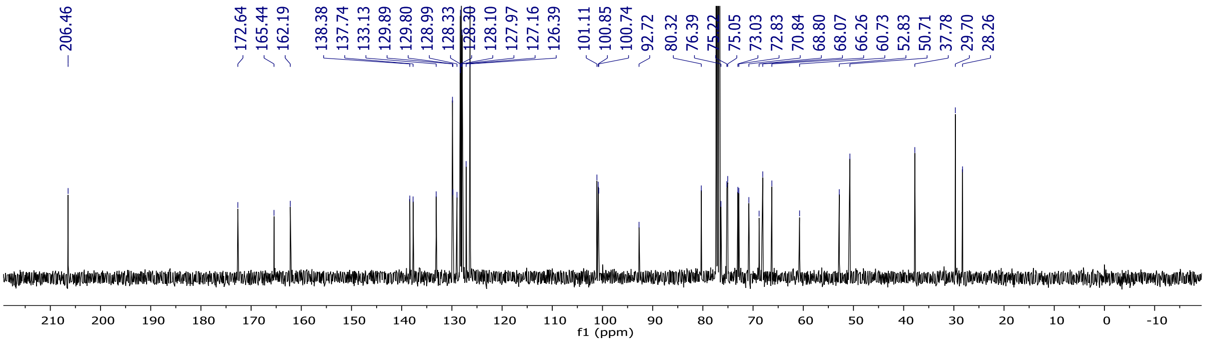
206.46
172.64 165.44 162.19
138.38 137.74 133.13 129.89 129.80 128.99 128.33 128.30 128.10 127.97 127.16 126.39
101.11 100.85 100.74 92.72 80.32 76.39 75.22 75.05 73.03 72.83 70.84 68.80 68.07 66.26 60.73 52.83 50.71 37.78 29.70 28.26
210 200 190 180 170 160 150 140 130 120 110 100 90 80 70 60 50 40 30 20 10 0 -10
f1 (ppm)

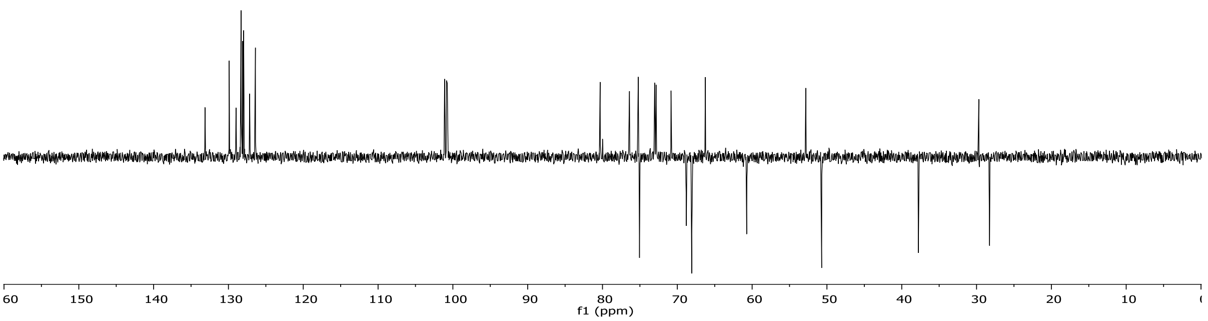
150 140 130 120 110 100 90 80 70 60 50 40 30 20 10
f1 (ppm)

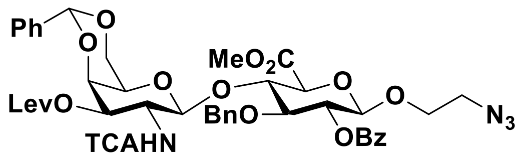
Ph
O
O
O
LevO
TCAHN
MeO2C
O
O
BnO
OBz
O
N3

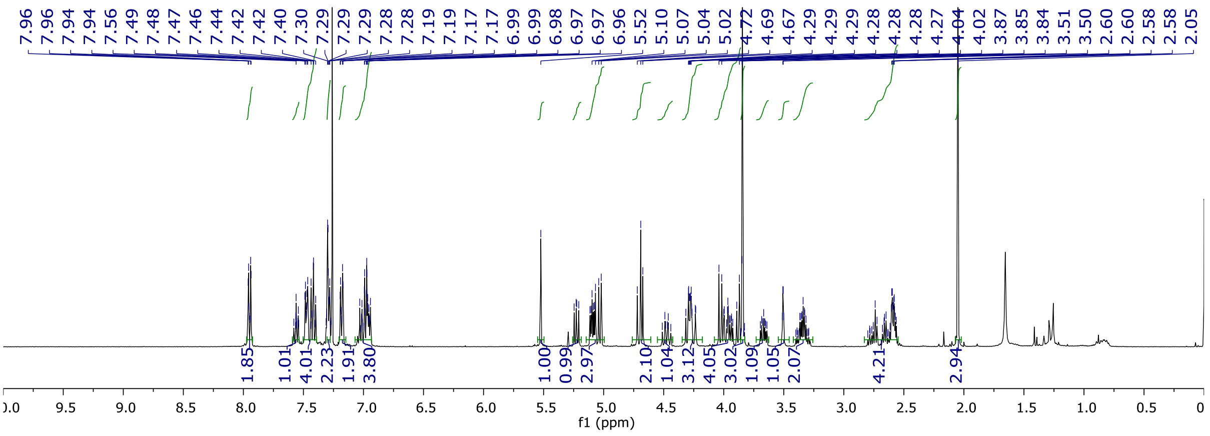
7.96 7.96 7.94 7.94 7.56 7.49 7.48 7.47 7.46 7.44 7.42 7.42 7.40 7.30 7.29 7.29 7.29 7.28 7.28 7.19 7.19 7.17 7.17 6.99 6.99 6.98 6.97 6.97 6.96 5.52 5.10 5.07 5.04 5.02 4.72 4.69 4.67 4.29 4.29 4.29 4.28 4.28 4.28 4.27 4.04 4.02 3.87 3.85 3.84 3.51 3.50 2.60 2.60 2.58 2.58 2.05
1.85 1.01 4.01 2.23 1.91 3.80 1.00 0.99 2.97 2.10 1.04 3.12 4.05 3.02 1.09 1.05 2.07 4.21 2.94
f1 (ppm)

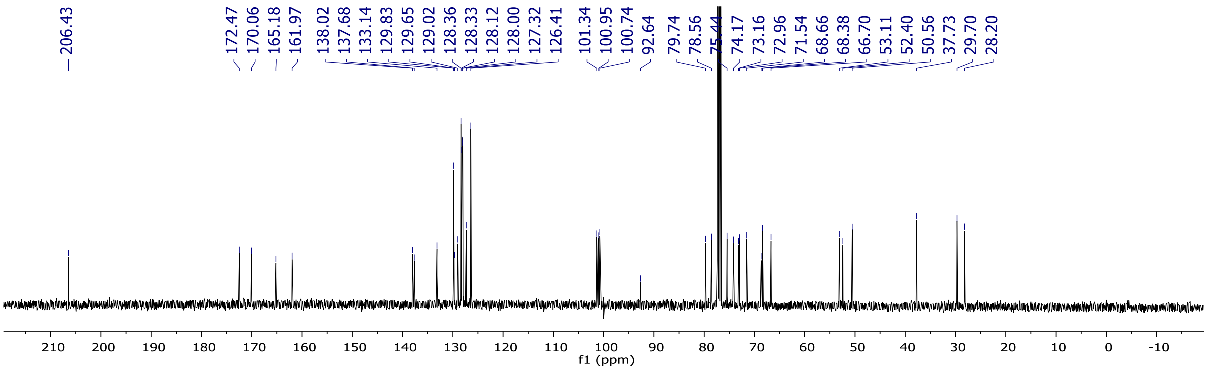
206.43 172.47 170.06 165.18 161.97 138.02 137.68 133.14 129.83 129.65 129.02 128.36 128.33 128.12 128.00 127.32 126.41 101.34 100.95 100.74 92.64 79.74 78.56 75.44 74.17 73.16 72.96 71.54 68.66 68.38 66.70 53.11 52.40 50.56 37.73 29.70 28.20
f1 (ppm)

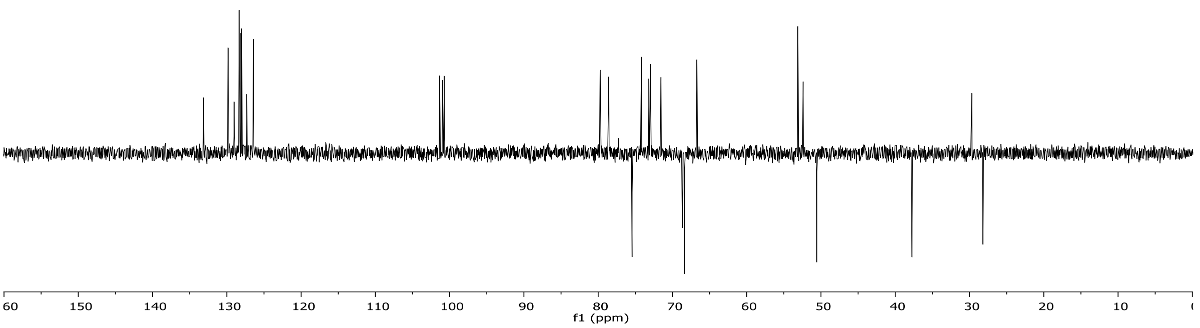
f1 (ppm)

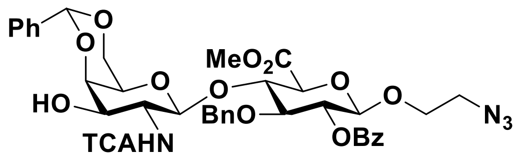
Ph
O
O
HO
O
TCAHN
MeO2C
O
O
BnO
O
O
OBz
N3

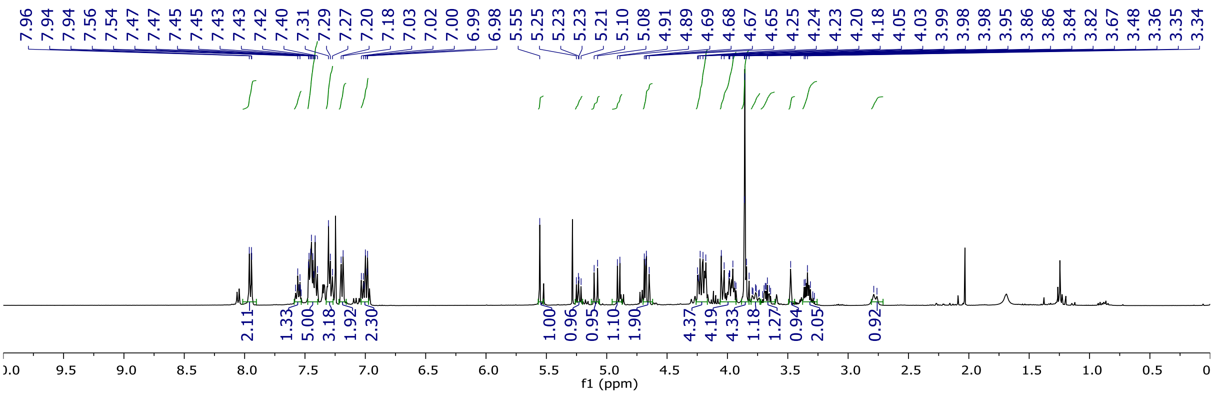
7.96
7.94
7.94
7.56
7.54
7.47
7.47
7.45
7.45
7.43
7.43
7.42
7.40
7.31
7.29
7.27
7.20
7.18
7.03
7.02
7.00
6.99
6.98
5.55
5.25
5.23
5.23
5.21
5.10
5.08
4.91
4.89
4.69
4.68
4.67
4.65
4.25
4.24
4.23
4.20
4.18
4.05
4.03
3.99
3.98
3.98
3.95
3.86
3.86
3.84
3.82
3.67
3.48
3.36
3.35
3.34
2.11
1.33
5.00
3.18
1.92
2.30
1.00
0.96
0.95
1.10
1.90
4.37
4.19
4.33
1.18
1.27
0.94
2.05
0.92
9.5
9.0
8.5
8.0
7.5
7.0
6.5
6.0
5.5
5.0
4.5
4.0
3.5
3.0
2.5
2.0
1.5
1.0
0.5
0
f1 (ppm)

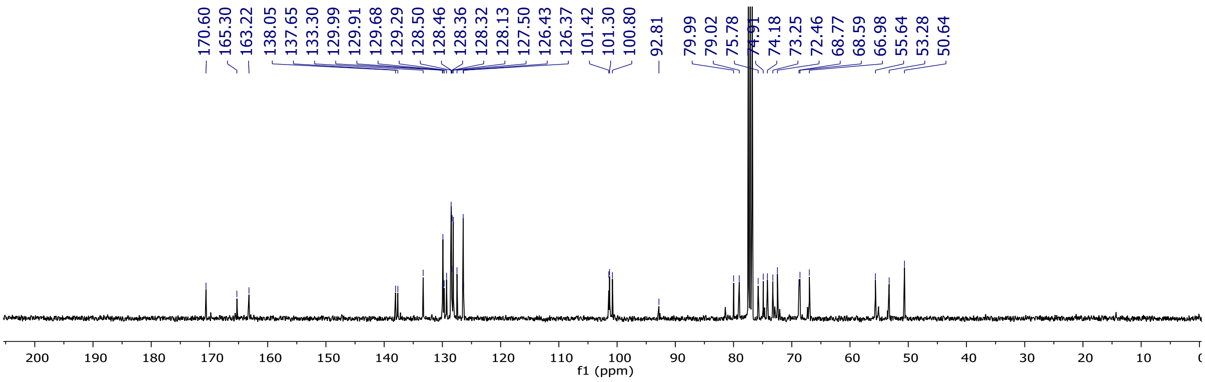
170.60
165.30
163.22
138.05
137.65
133.30
129.99
129.91
129.68
129.29
128.50
128.46
128.36
128.32
128.13
127.50
126.43
126.37
101.42
101.30
100.80
92.81
79.99
79.02
75.78
74.91
74.18
73.25
72.46
68.77
68.59
66.98
55.64
53.28
50.64
200
190
180
170
160
150
140
130
120
110
100
90
80
70
60
50
40
30
20
10
f1 (ppm)

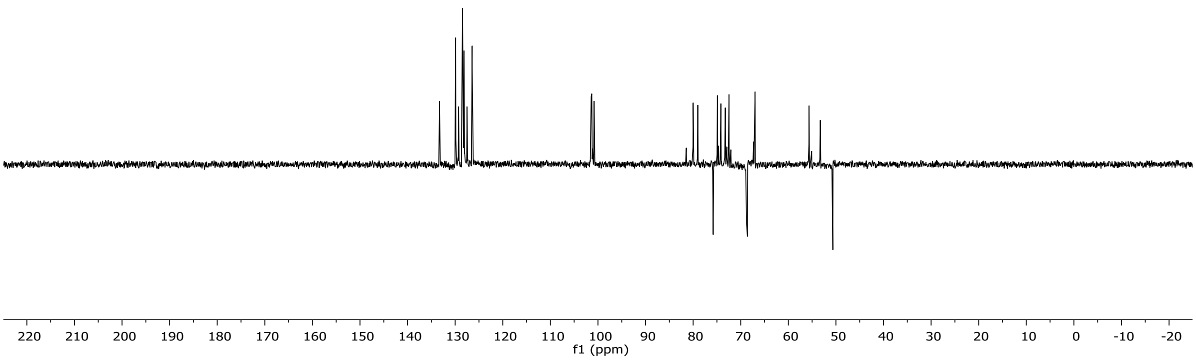
220
210
200
190
180
170
160
150
140
130
120
110
100
90
80
70
60
50
40
30
20
10
0
-10
-20
f1 (ppm)

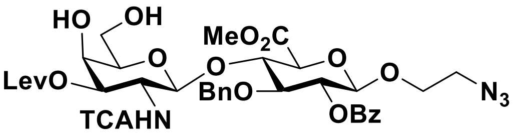
HO
OH
MeO2C
O
LevO
BnO
TCAHN
OBz
N3

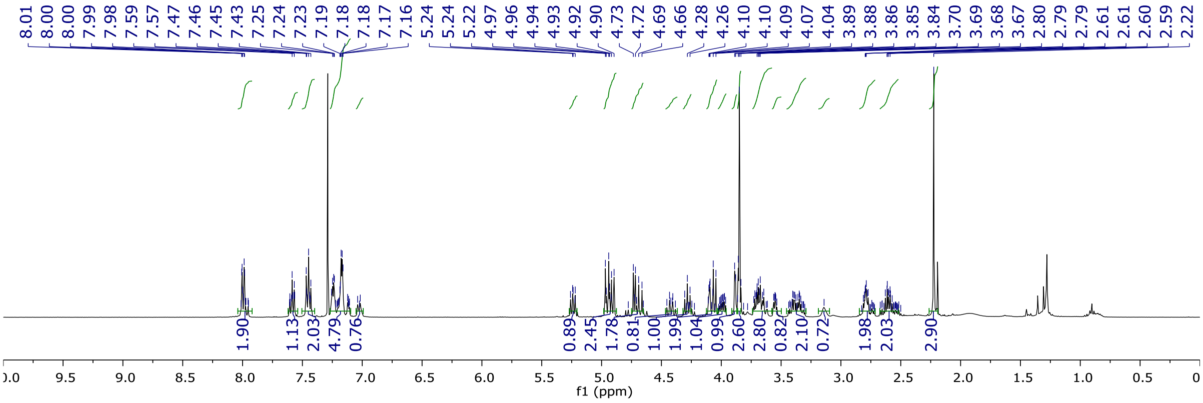
8.01 8.00 8.00 7.99 7.98 7.59 7.57 7.47 7.46 7.45 7.43 7.25 7.24 7.23 7.19 7.18 7.18 7.17 7.16 5.24 5.24 5.22 4.97 4.96 4.94 4.93 4.92 4.90 4.73 4.72 4.69 4.66 4.28 4.26 4.10 4.10 4.09 4.07 4.04 3.89 3.88 3.86 3.85 3.84 3.70 3.69 3.68 3.67 2.80 2.79 2.79 2.61 2.61 2.60 2.59 2.22
1.90 1.13 2.03 4.79 0.76 0.89 2.45 1.78 0.81 1.00 1.99 1.04 0.99 2.60 2.80 0.82 2.10 0.72 1.98 2.03 2.90
f1 (ppm)

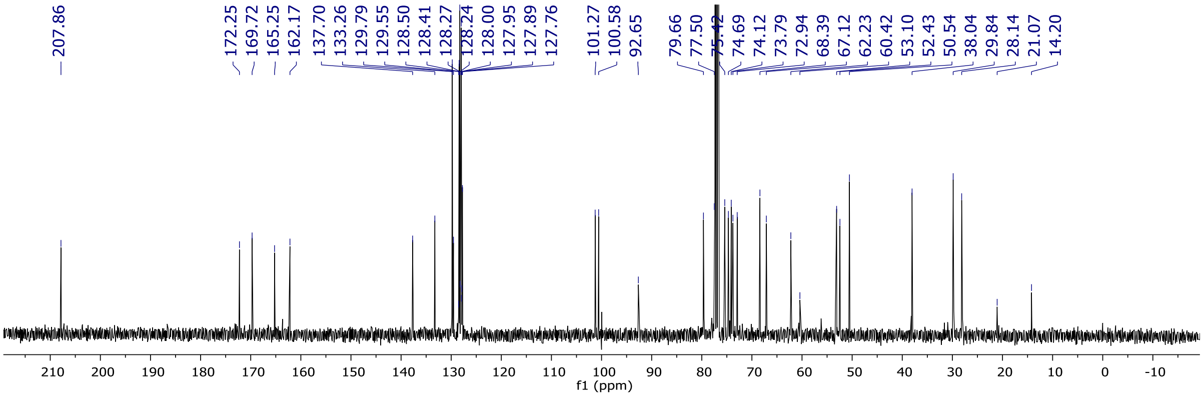
207.86 172.25 169.72 165.25 162.17 137.70 133.26 129.79 129.55 128.50 128.41 128.27 128.24 128.00 127.95 127.89 127.76 101.27 100.58 92.65 79.66 77.50 75.42 74.69 74.12 73.79 72.94 68.39 67.12 62.23 60.42 53.10 52.43 50.54 38.04 29.84 28.14 21.07 14.20
f1 (ppm)

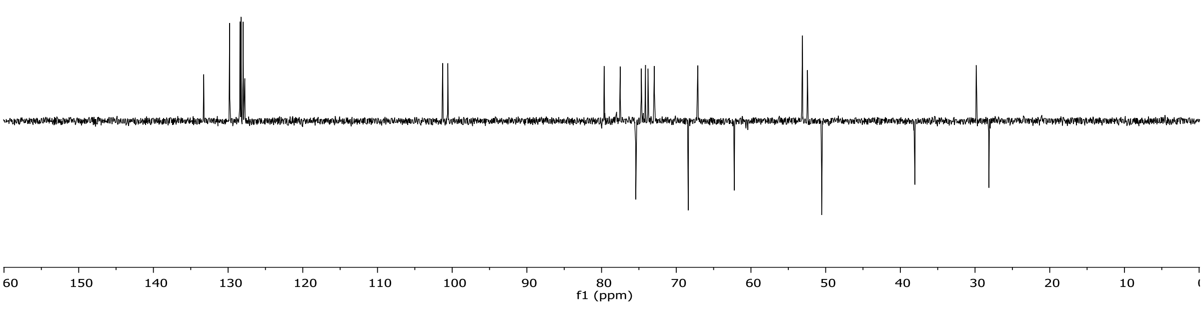
f1 (ppm)

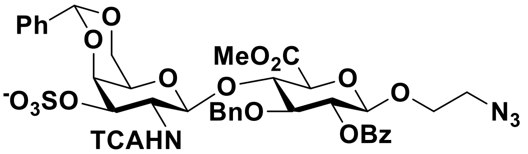
Ph
O
O
O
-O3SO
TCAHN
O
MeO2C
O
BnO
O
N3
OBz

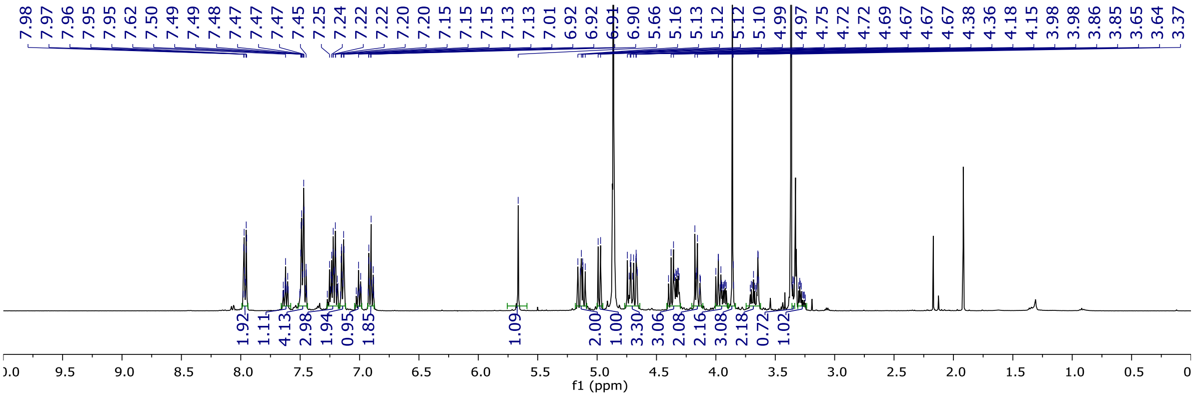
7.98 7.97 7.96 7.95 7.95 7.62 7.50 7.49 7.49 7.48 7.47 7.47 7.47 7.45 7.25 7.24 7.22 7.22 7.20 7.20 7.15 7.15 7.15 7.13 7.13 7.01 6.92 6.92 6.91 6.90 5.66 5.16 5.13 5.12 5.12 5.10 4.99 4.97 4.75 4.72 4.72 4.69 4.67 4.67 4.67 4.38 4.36 4.18 4.15 3.98 3.98 3.86 3.85 3.65 3.64 3.37
1.92 1.11 4.13 2.98 1.94 0.95 1.85 1.09 2.00 1.00 3.30 3.06 2.08 2.16 3.08 2.18 0.72 1.02
f1 (ppm)

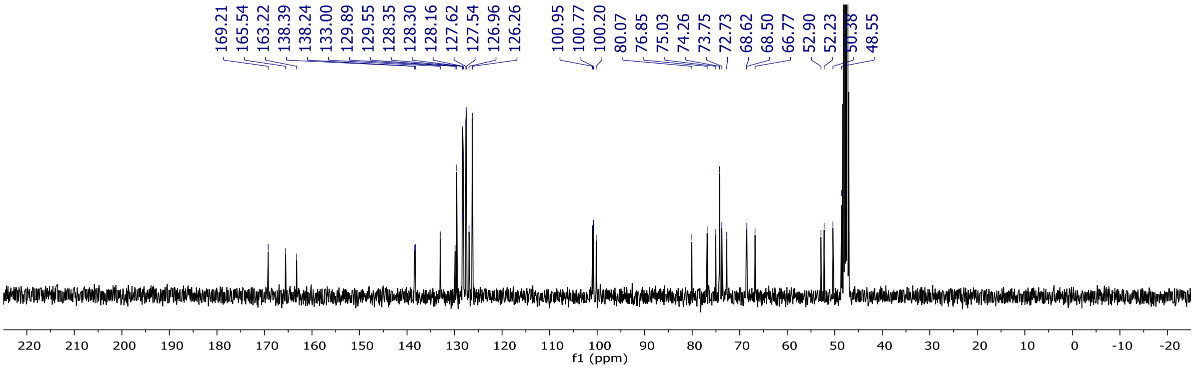
169.21 165.54 163.22 138.39 138.24 133.00 129.89 129.55 128.35 128.30 128.16 127.62 127.54 126.96 126.26 100.95 100.77 100.20 80.07 76.85 75.03 74.26 73.75 72.73 68.62 68.50 66.77 52.90 52.23 50.38 48.55
f1 (ppm)

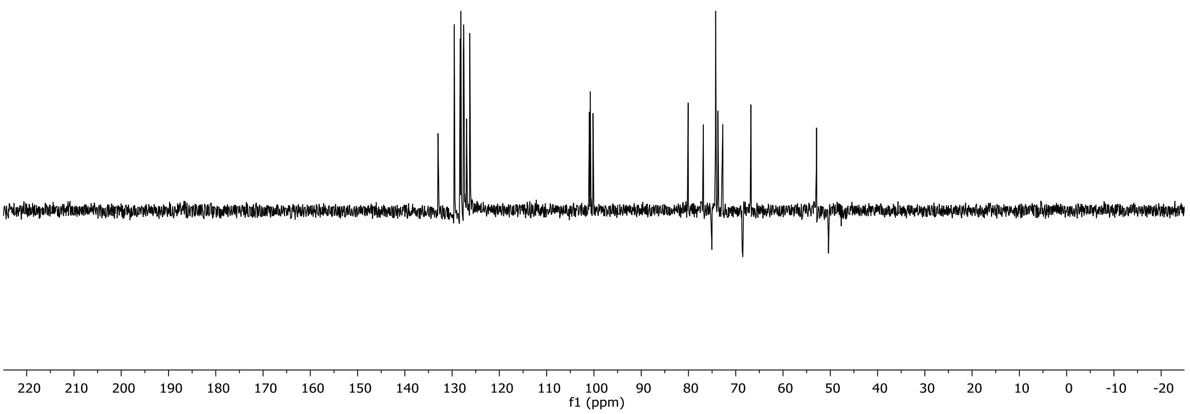
f1 (ppm)

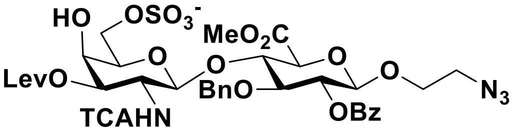
HO
OSO3-
MeO2C
LevO
O
BnO
TCAHN
OBz
N3

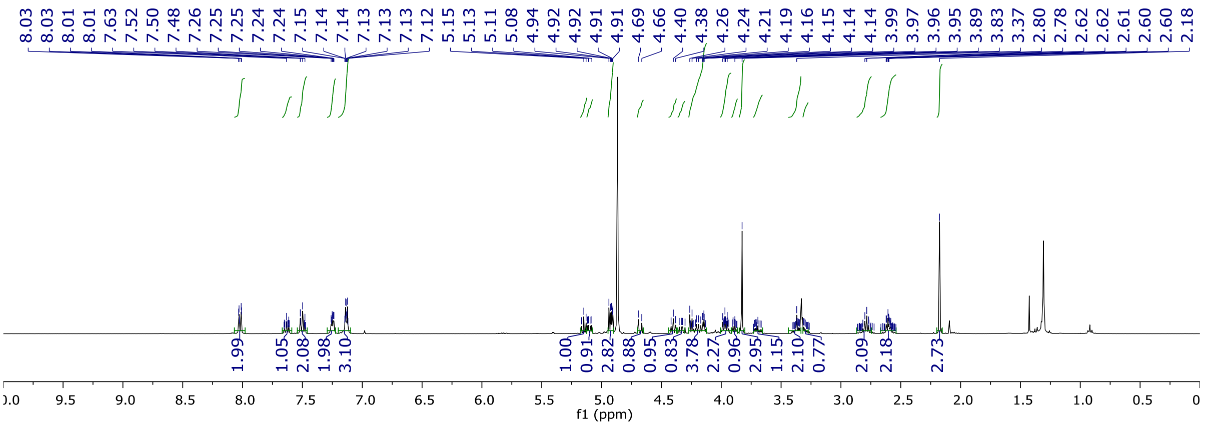
8.03 8.03 8.01 8.01 7.63 7.52 7.50 7.48 7.26 7.25 7.25 7.24 7.24 7.15 7.14 7.14 7.13 7.13 7.13 7.12 5.15 5.13 5.11 5.08 4.94 4.92 4.92 4.91 4.91 4.69 4.66 4.40 4.38 4.26 4.24 4.21 4.19 4.16 4.15 4.14 4.14 3.99 3.97 3.96 3.95 3.89 3.83 3.37 2.80 2.78 2.62 2.62 2.61 2.60 2.60 2.18
1.99 1.05 2.08 1.98 3.10 1.00 0.91 2.82 0.88 0.95 0.83 3.78 2.27 0.96 2.95 1.15 2.10 0.77 2.09 2.18 2.73
f1 (ppm)

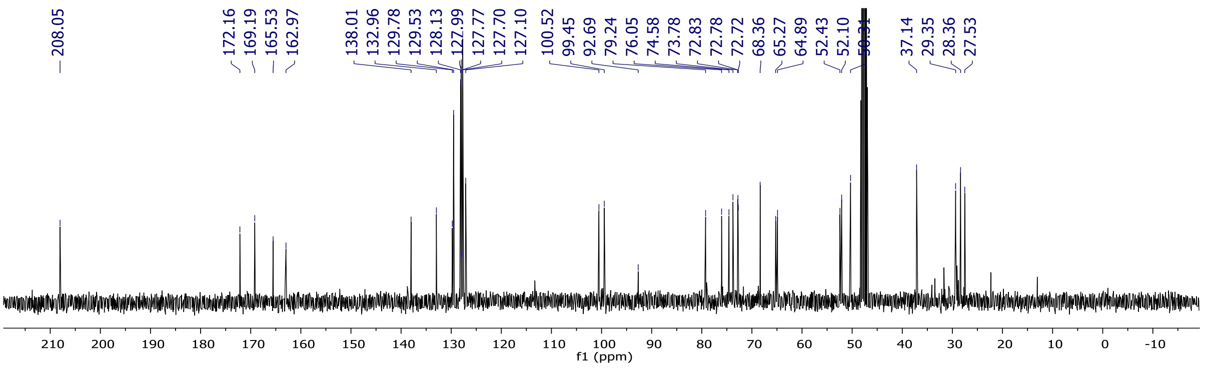
208.05 172.16 169.19 165.53 162.97 138.01 132.96 129.78 129.53 128.13 127.99 127.77 127.70 127.10 100.52 99.45 92.69 79.24 76.05 74.58 73.78 72.83 72.78 72.72 68.36 65.27 64.89 52.43 52.10 37.14 29.35 28.36 27.53
f1 (ppm)

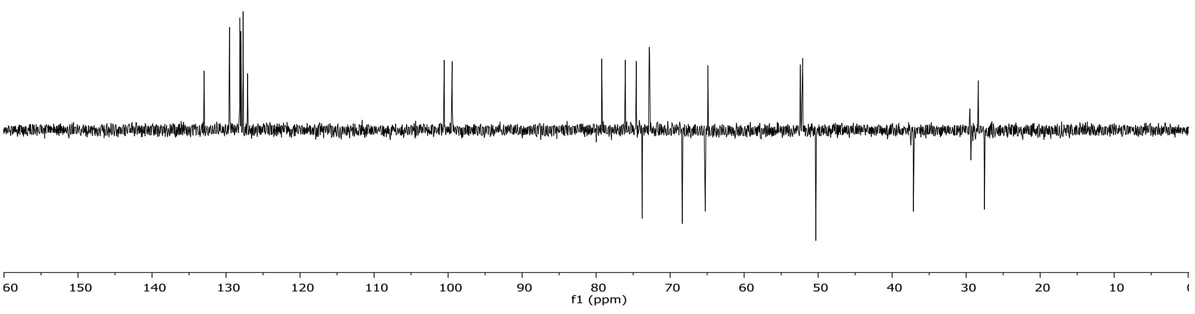
f1 (ppm)

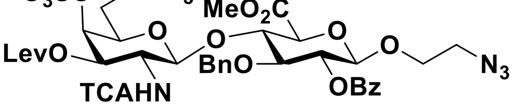
-O3SO
OSO3-
MeO2C
LevO
O
BnO
TCAHN
OBz
N3

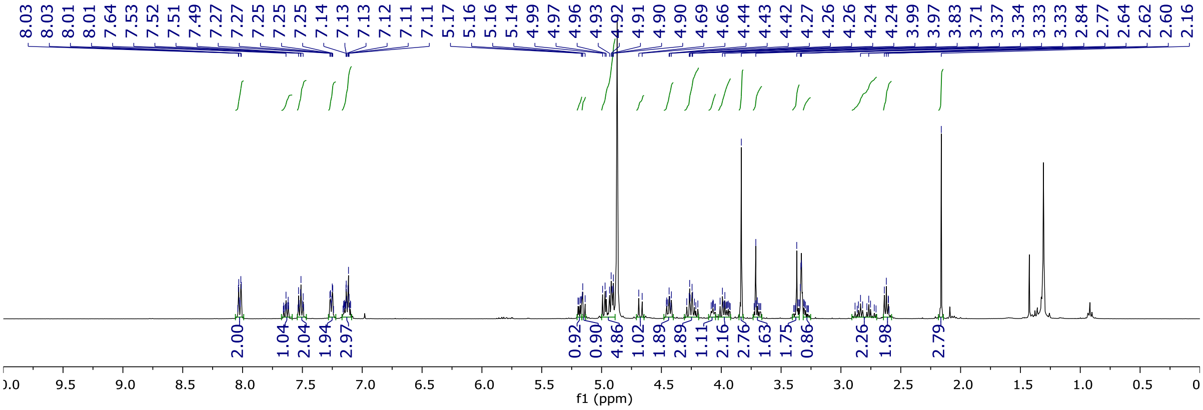
8.03 8.03 8.01 8.01 7.64 7.53 7.52 7.51 7.49 7.27 7.25 7.25 7.14 7.13 7.13 7.12 7.11 7.11 5.17 5.16 5.14 4.99 4.97 4.96 4.93 4.92 4.91 4.90 4.90 4.69 4.66 4.44 4.43 4.42 4.27 4.26 4.26 4.24 4.24 3.99 3.97 3.83 3.71 3.37 3.34 3.33 3.33 2.84 2.77 2.64 2.62 2.60 2.16
2.00 1.04 2.04 1.94 2.97 0.92 0.90 4.86 1.02 1.89 2.89 1.11 2.16 2.76 1.63 1.75 0.86 2.26 1.98 2.79
f1 (ppm)

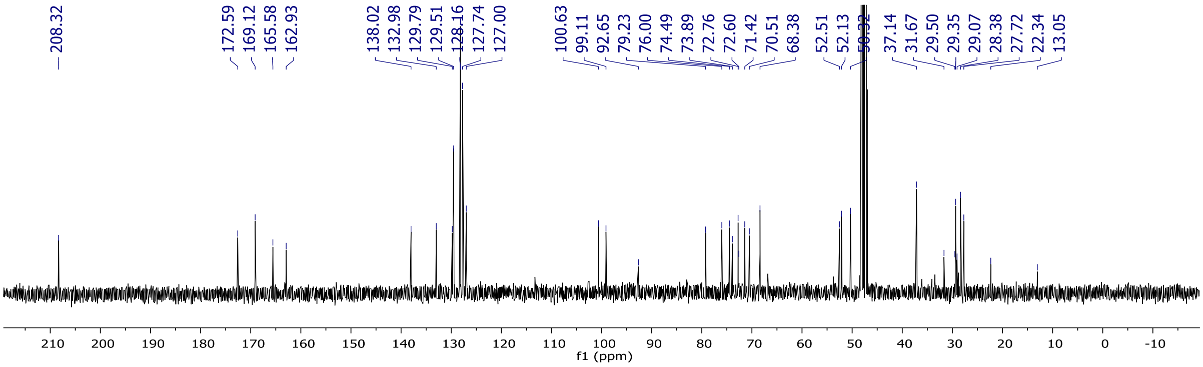
208.32
172.59 169.12 165.58 162.93
138.02 132.98 129.79 129.51 128.16 127.74 127.00
100.63 99.11 92.65 79.23 76.00 74.49 73.89 72.76 72.60 71.42 70.51 68.38
52.51 52.13 50.32 37.14 31.67 29.50 29.35 29.07 28.38 27.72 22.34 13.05
f1 (ppm)

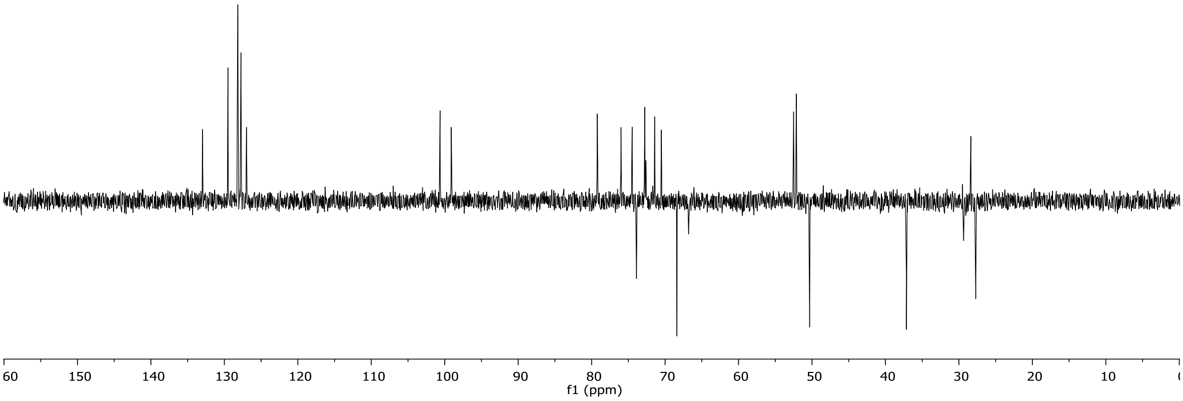
f1 (ppm)

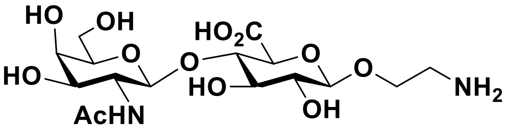
HO
OH
O
HO
AcHN
O
HO_2C
O
HO
OH
O
NH_2

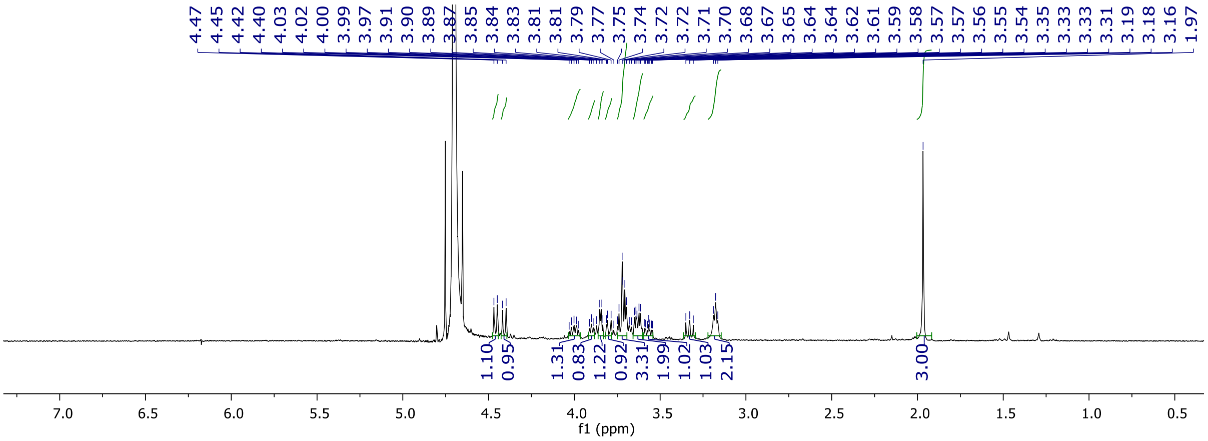
4.47 4.45 4.42 4.40 4.03 4.02 4.00 3.99 3.97 3.91 3.90 3.89 3.87 3.85 3.84 3.83 3.81 3.81 3.79 3.77 3.75 3.74 3.72 3.72 3.71 3.70 3.68 3.67 3.65 3.64 3.64 3.62 3.61 3.59 3.58 3.57 3.57 3.56 3.55 3.54 3.35 3.33 3.33 3.31 3.19 3.18 3.16 1.97
1.10 0.95 1.31 0.83 1.22 0.92 3.31 1.99 1.02 1.03 2.15 3.00
7.0 6.5 6.0 5.5 5.0 4.5 4.0 3.5 3.0 2.5 2.0 1.5 1.0 0.5
f1 (ppm)

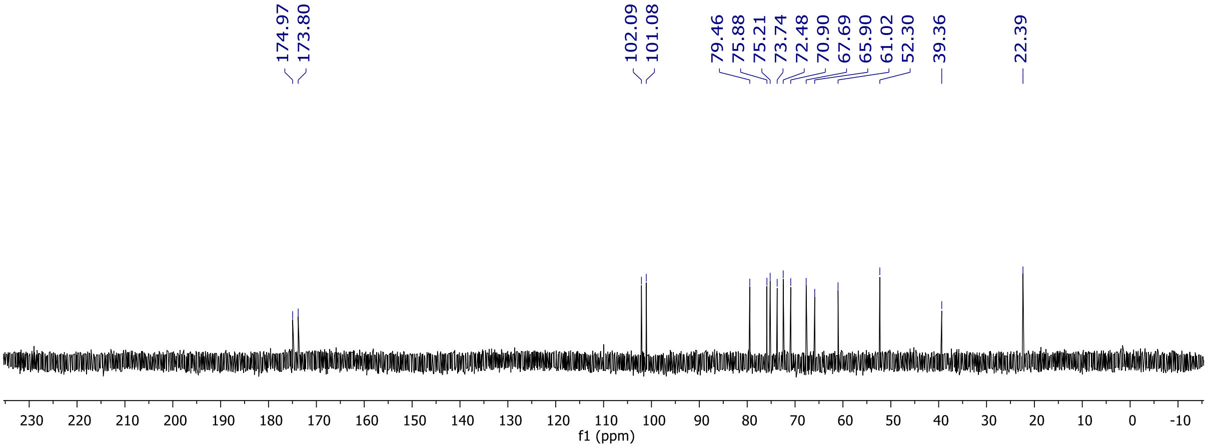
174.97 173.80 102.09 101.08 79.46 75.88 75.21 73.74 72.48 70.90 67.69 65.90 61.02 52.30 39.36 22.39
230 220 210 200 190 180 170 160 150 140 130 120 110 100 90 80 70 60 50 40 30 20 10 0 -10
f1 (ppm)

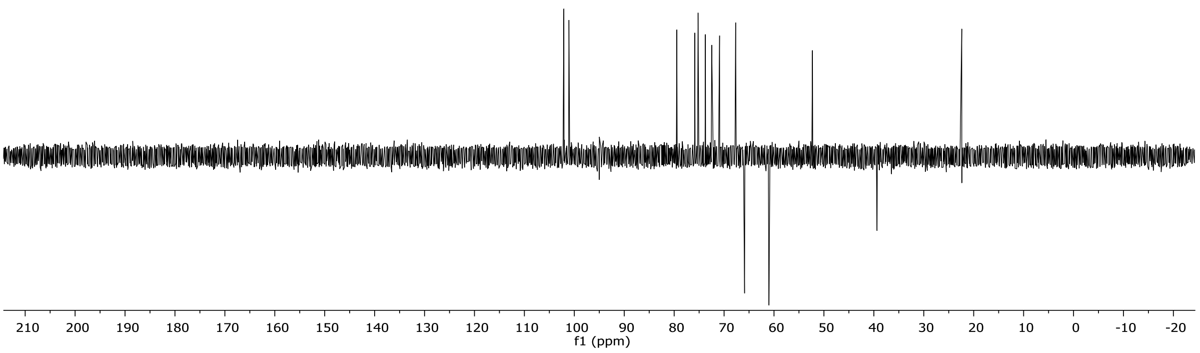
210 200 190 180 170 160 150 140 130 120 110 100 90 80 70 60 50 40 30 20 10 0 -10 -20
f1 (ppm)

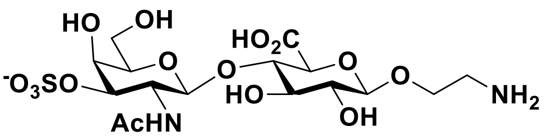
HO
OH
HO2C
O
O
O
O
-O3SO
HO
NH2
AcHN
OH

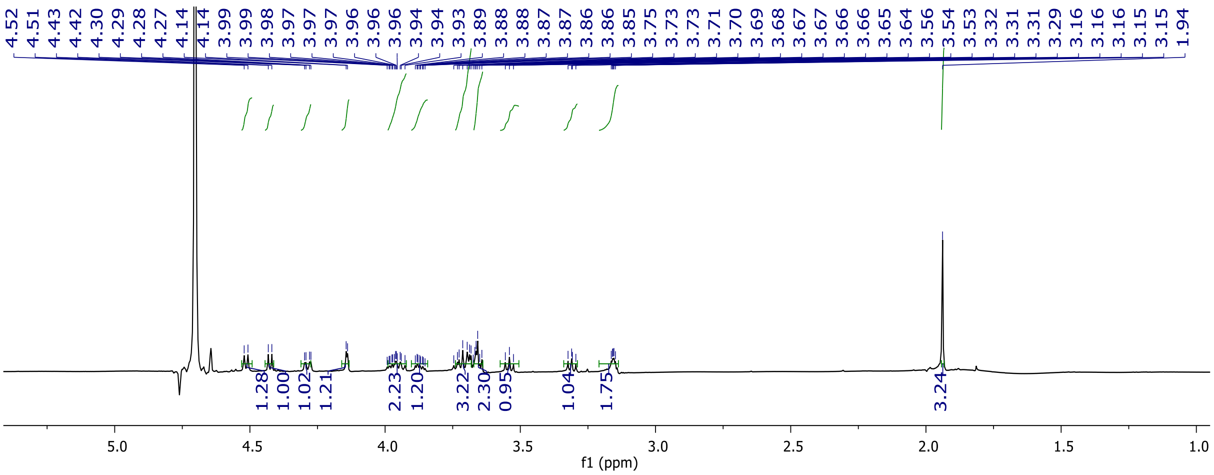
4.52 4.51 4.43 4.42 4.30 4.29 4.28 4.27 4.14 4.14 3.99 3.98 3.97 3.97 3.97 3.96 3.96 3.94 3.94 3.93 3.89 3.88 3.88 3.87 3.86 3.86 3.85 3.75 3.73 3.73 3.71 3.70 3.69 3.68 3.67 3.67 3.66 3.66 3.65 3.64 3.56 3.54 3.53 3.32 3.31 3.31 3.29 3.16 3.16 3.15 3.15 1.94
1.28 1.00 1.02 1.21 2.23 1.20 3.22 2.30 0.95 1.04 1.75 3.24
5.0 4.5 4.0 3.5 3.0 2.5 2.0 1.5 1.0
f1 (ppm)

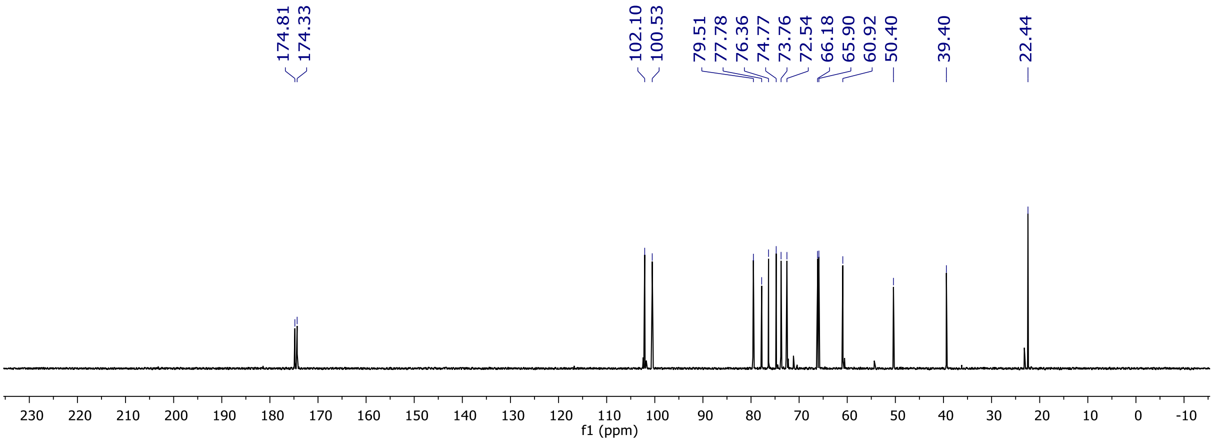
174.81 174.33 102.10 100.53 79.51 77.78 76.36 74.77 73.76 72.54 66.18 65.90 60.92 50.40 39.40 22.44
230 220 210 200 190 180 170 160 150 140 130 120 110 100 90 80 70 60 50 40 30 20 10 0 -10
f1 (ppm)

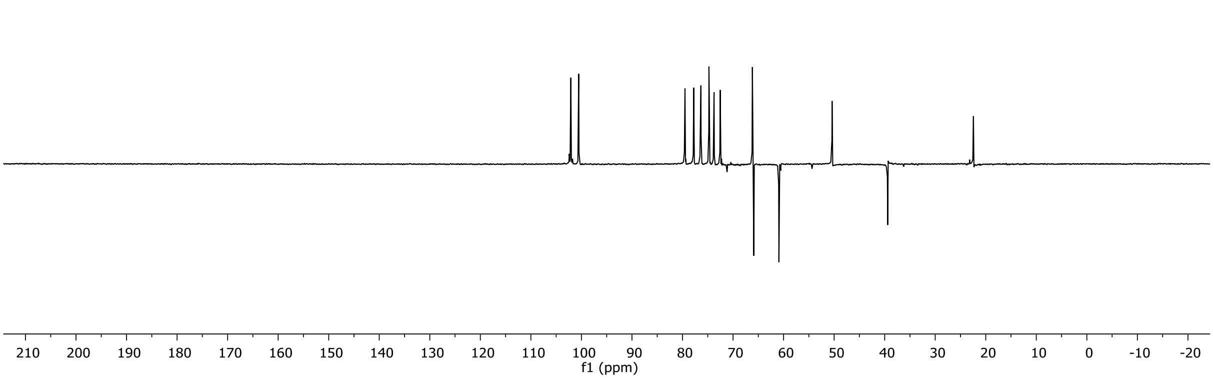
210 200 190 180 170 160 150 140 130 120 110 100 90 80 70 60 50 40 30 20 10 0 -10 -20
f1 (ppm)

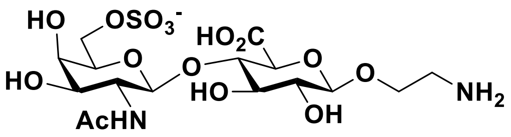
HO
OSO3-
HO2C
O
HO
HO
NH2
AcHN
OH

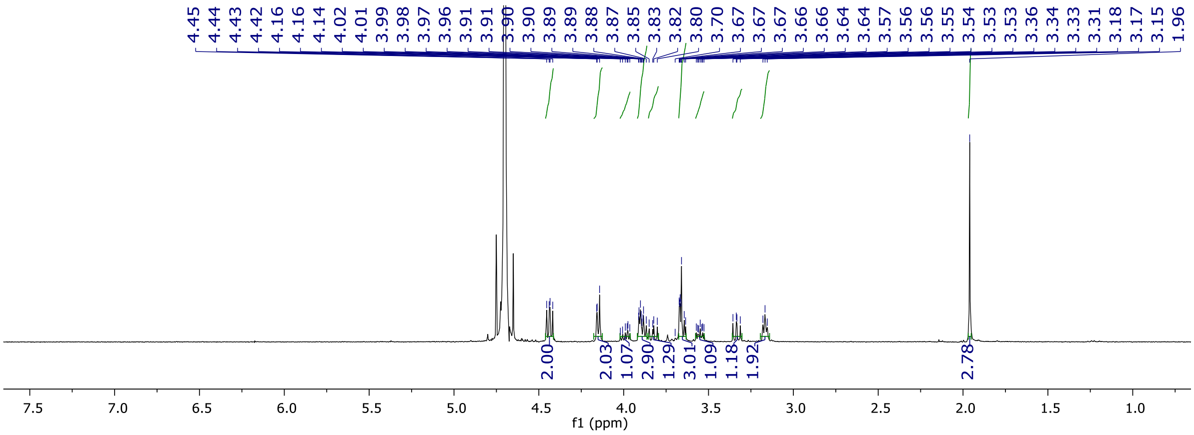
4.45 4.44 4.43 4.42 4.16 4.16 4.14 4.02 4.01 3.99 3.98 3.97 3.96 3.91 3.91 3.90 3.90 3.89 3.89 3.88 3.87 3.85 3.83 3.82 3.80 3.70 3.67 3.67 3.67 3.66 3.66 3.64 3.64 3.57 3.56 3.56 3.55 3.54 3.53 3.53 3.36 3.34 3.33 3.31 3.18 3.17 3.15 1.96
2.00 2.03 1.07 2.90 1.29 3.01 1.09 1.18 1.92 2.78
7.5 7.0 6.5 6.0 5.5 5.0 4.5 4.0 3.5 3.0 2.5 2.0 1.5 1.0
f1 (ppm)

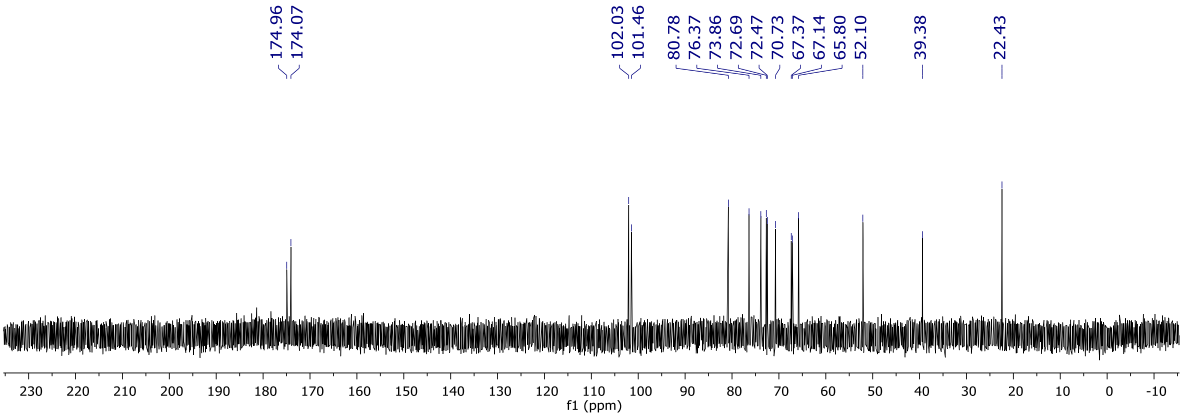
174.96 174.07 102.03 101.46 80.78 76.37 73.86 72.69 72.47 70.73 67.37 67.14 65.80 52.10 39.38 22.43
230 220 210 200 190 180 170 160 150 140 130 120 110 100 90 80 70 60 50 40 30 20 10 0 -10
f1 (ppm)

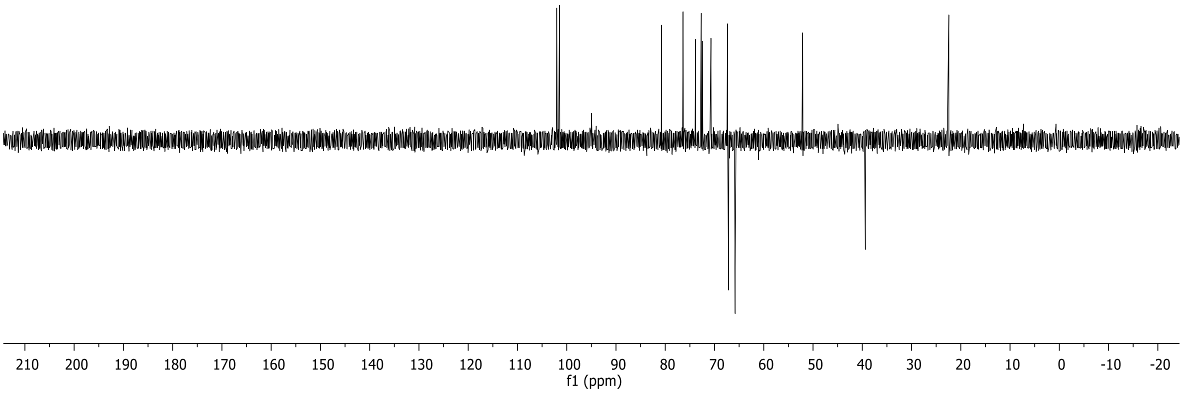
210 200 190 180 170 160 150 140 130 120 110 100 90 80 70 60 50 40 30 20 10 0 -10 -20
f1 (ppm)

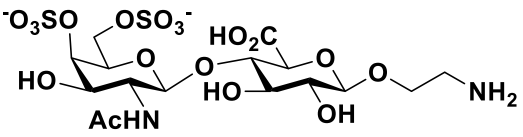

⁻O3SO
OSO3⁻
HO2C
O
HO
O
O
NH2
AcHN
HO
OH

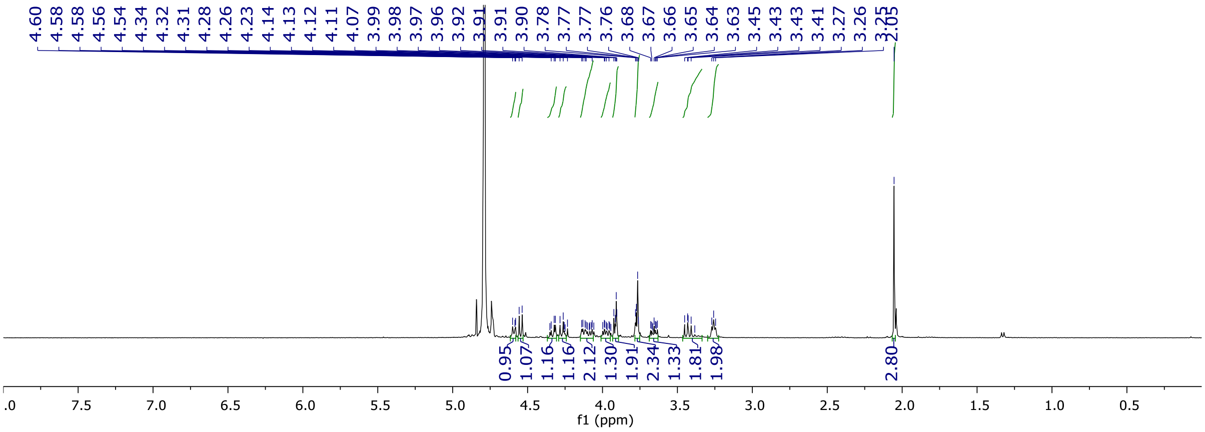

4.60 4.58 4.58 4.56 4.54 4.34 4.32 4.31 4.28 4.26 4.23 4.14 4.13 4.12 4.11 4.07 3.99 3.98 3.97 3.96 3.92 3.91 3.91 3.90 3.78 3.77 3.77 3.76 3.68 3.67 3.66 3.65 3.64 3.63 3.45 3.43 3.43 3.41 3.27 3.26 3.25 2.05
0.95 1.07 1.16 1.16 2.12 1.30 1.91 2.34 1.33 1.81 1.98 2.80
.0 7.5 7.0 6.5 6.0 5.5 5.0 4.5 4.0 3.5 3.0 2.5 2.0 1.5 1.0 0.5
f1 (ppm)

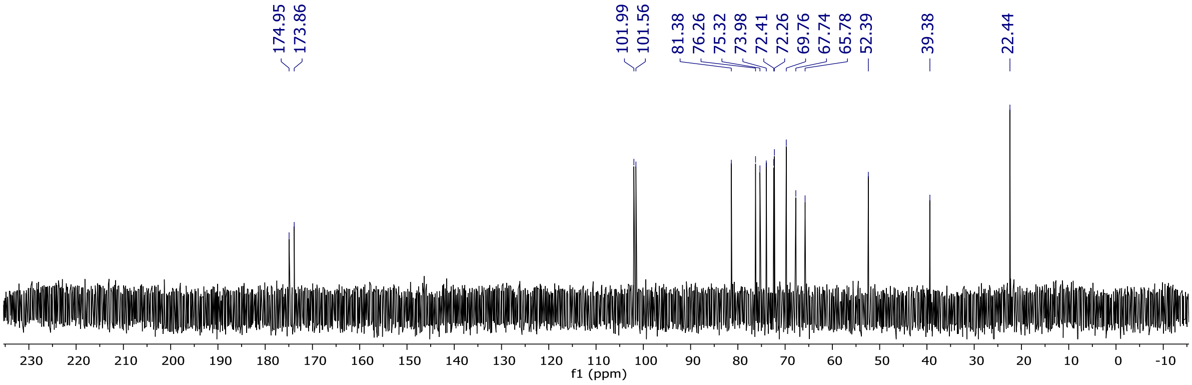

174.95 173.86 101.99 101.56 81.38 76.26 75.32 73.98 72.41 72.26 69.76 67.74 65.78 52.39 39.38 22.44
230 220 210 200 190 180 170 160 150 140 130 120 110 100 90 80 70 60 50 40 30 20 10 0 -10
f1 (ppm)

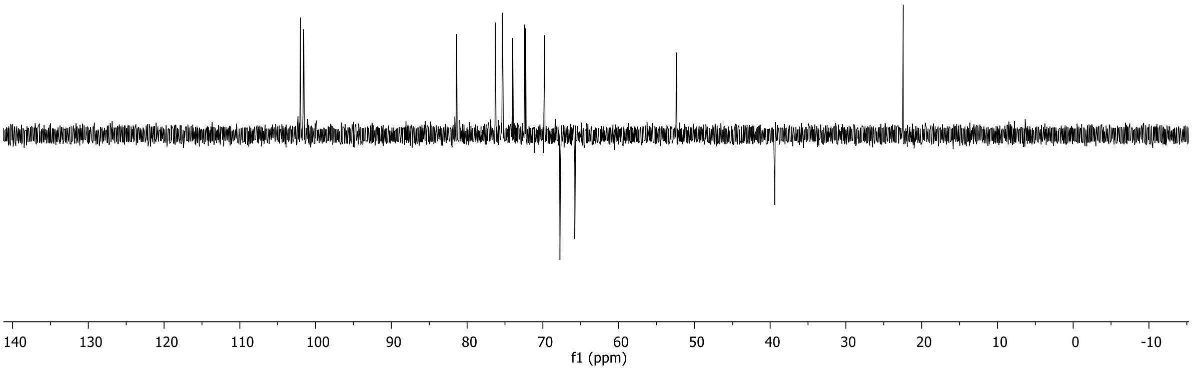

140 130 120 110 100 90 80 70 60 50 40 30 20 10 0 -10
f1 (ppm)

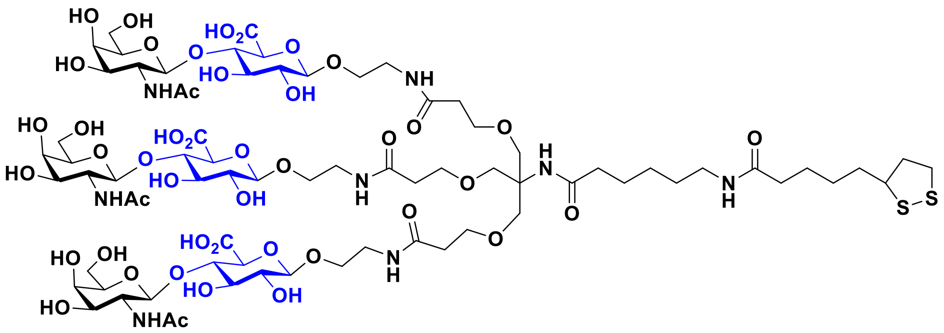

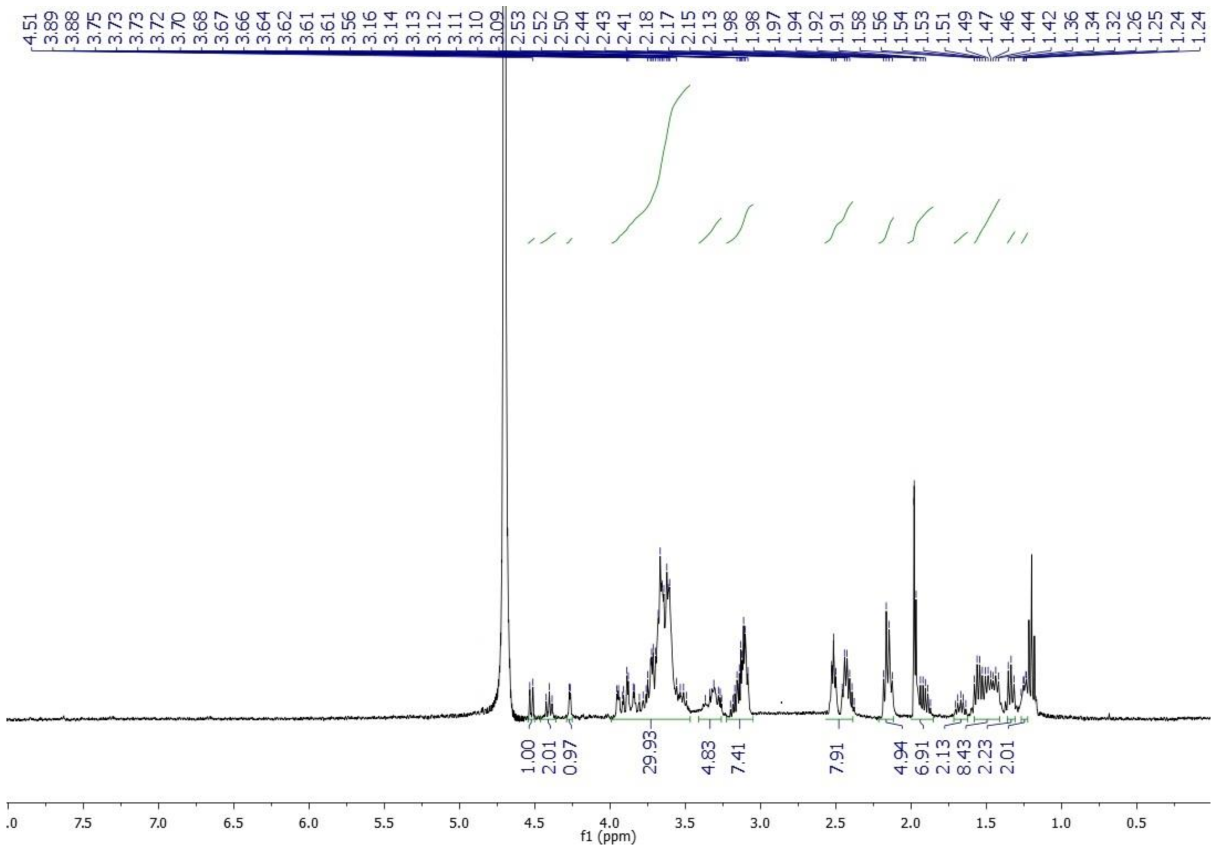

4.51 3.89 3.88 3.75 3.73 3.72 3.70 3.68 3.67 3.66 3.64 3.62 3.61 3.61 3.56 3.16 3.14 3.13 3.12 3.11 3.10 3.09 2.53 2.52 2.50 2.44 2.43 2.41 2.18 2.17 2.15 2.13 1.98 1.98 1.97 1.94 1.92 1.91 1.58 1.56 1.54 1.53 1.51 1.49 1.47 1.46 1.44 1.42 1.36 1.34 1.32 1.26 1.25 1.24 1.24
1.00 2.01 0.97 29.93 4.83 7.41 7.91 4.94 6.91 2.13 8.43 2.23 2.01
f1 (ppm)

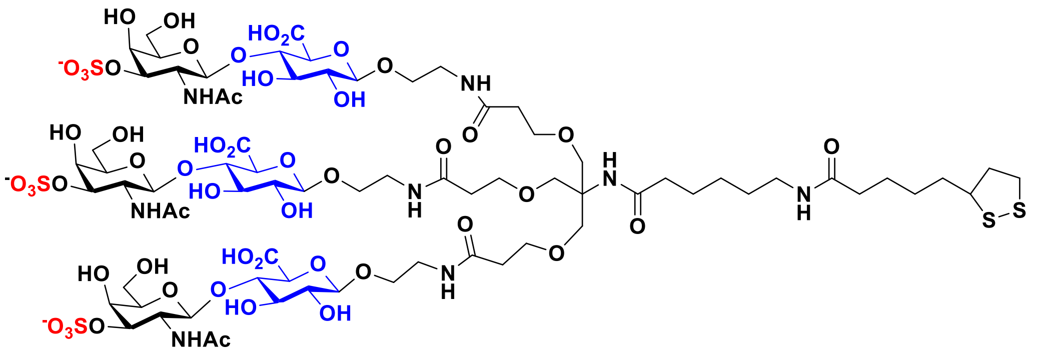

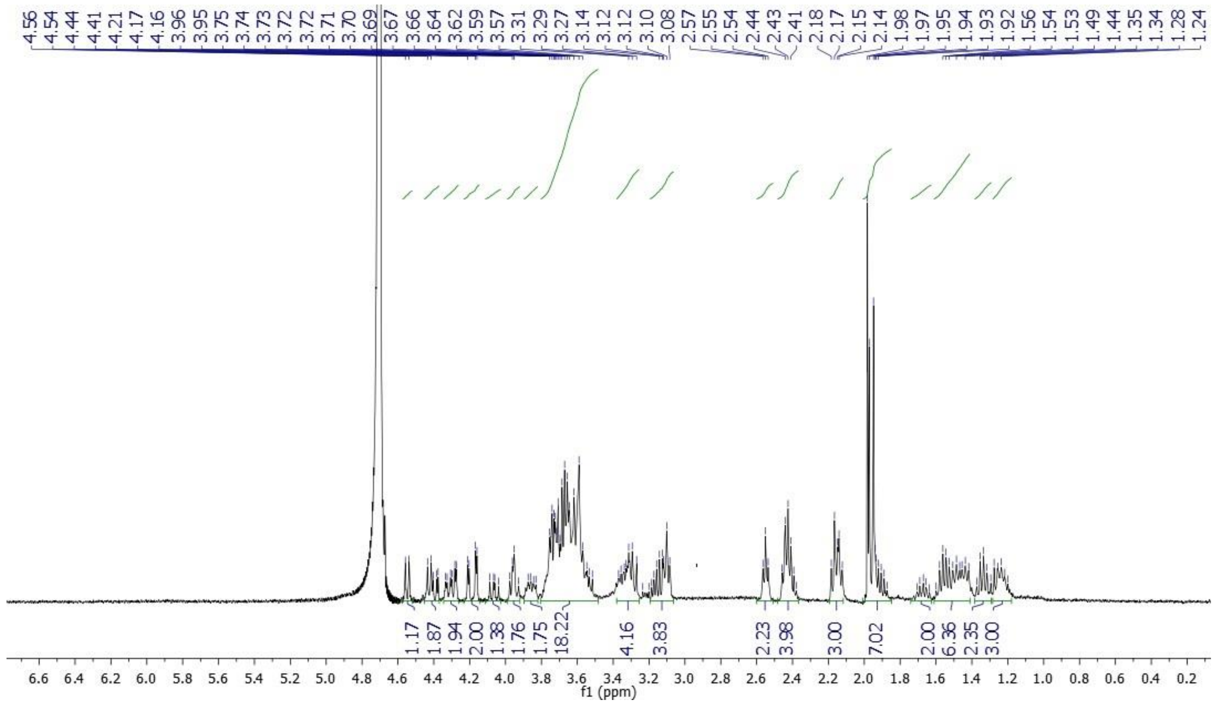

4.56 4.54 4.44 4.41 4.21 4.17 4.16 3.96 3.95 3.75 3.74 3.73 3.72 3.72 3.71 3.70 3.69 3.67 3.66 3.64 3.62 3.59 3.57 3.31 3.29 3.27 3.14 3.12 3.12 3.10 3.08 2.57 2.55 2.54 2.44 2.43 2.41 2.18 2.17 2.15 2.14 1.98 1.97 1.95 1.94 1.93 1.92 1.56 1.54 1.53 1.49 1.44 1.35 1.34 1.28 1.24
1.17 1.87 1.94 2.00 1.38 1.76 1.75 18.22 4.16 3.83 2.23 3.98 3.00 7.02 2.00 6.36 2.35 3.00
6.6 6.4 6.2 6.0 5.8 5.6 5.4 5.2 5.0 4.8 4.6 4.4 4.2 4.0 3.8 3.6 3.4 3.2 3.0 2.8 2.6 2.4 2.2 2.0 1.8 1.6 1.4 1.2 1.0 0.8 0.6 0.4 0.2
f1 (ppm)

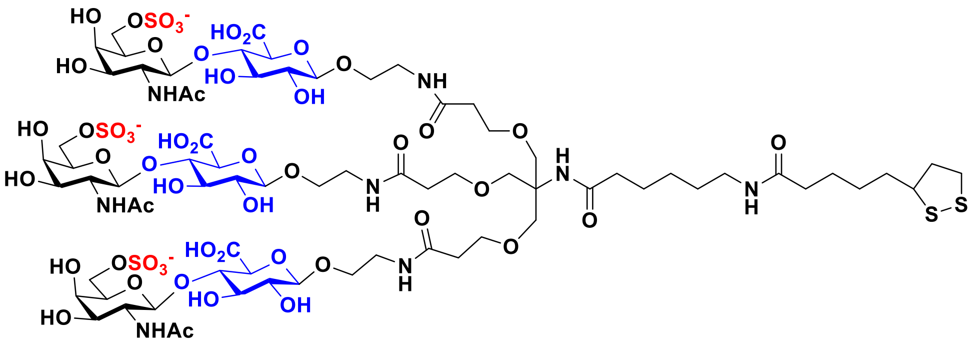
HO
OSO3-
HO2C
NH
HO
OH
NHAc
O
H
N
S
S

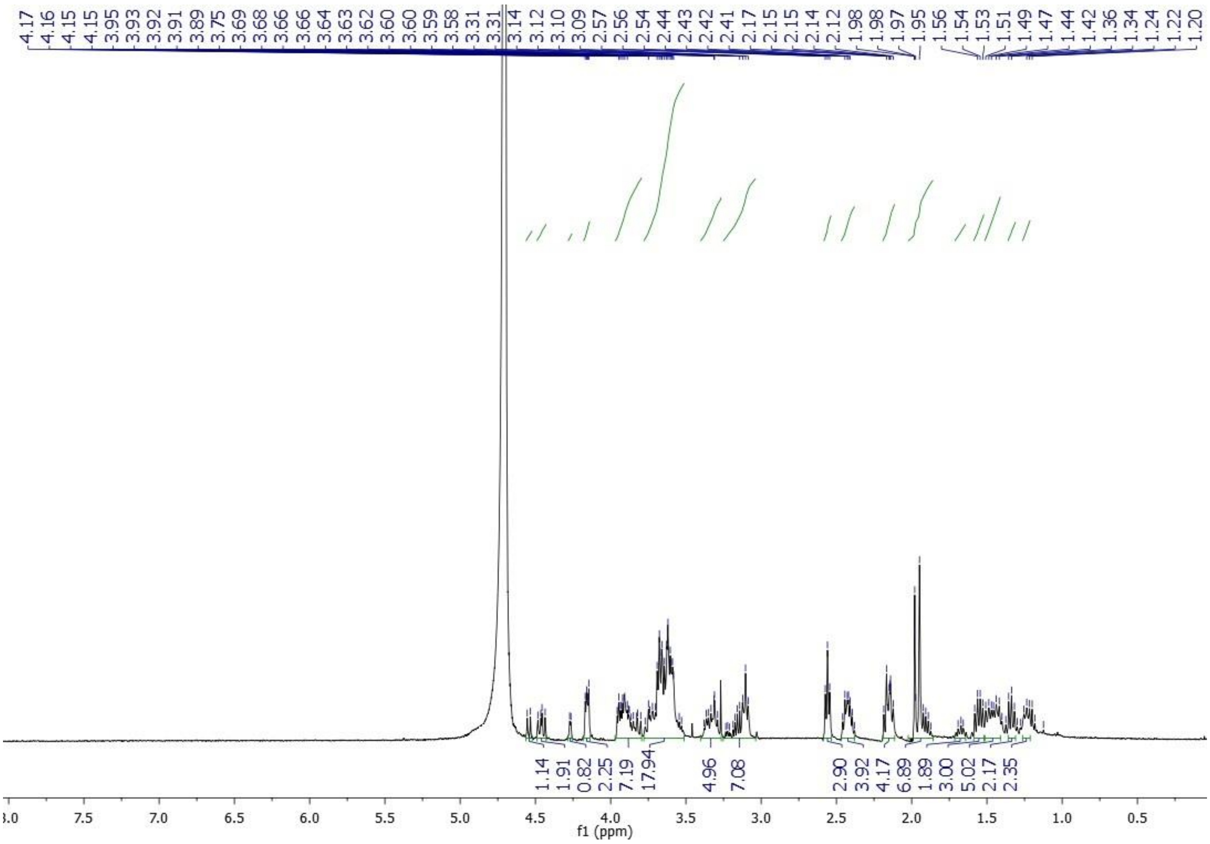
f1 (ppm)

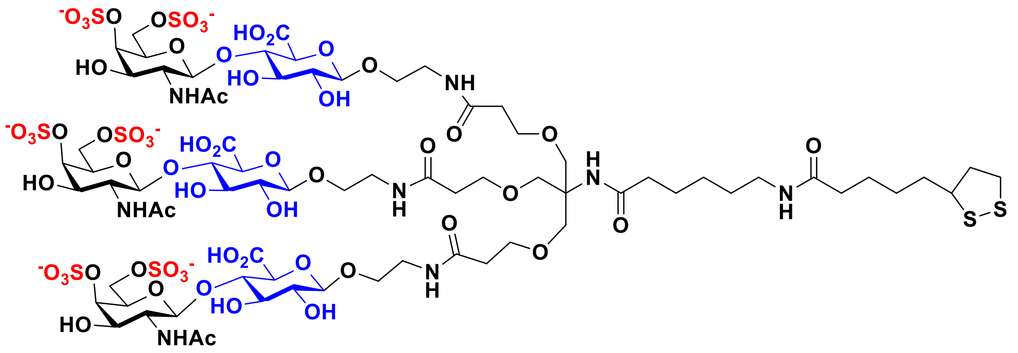

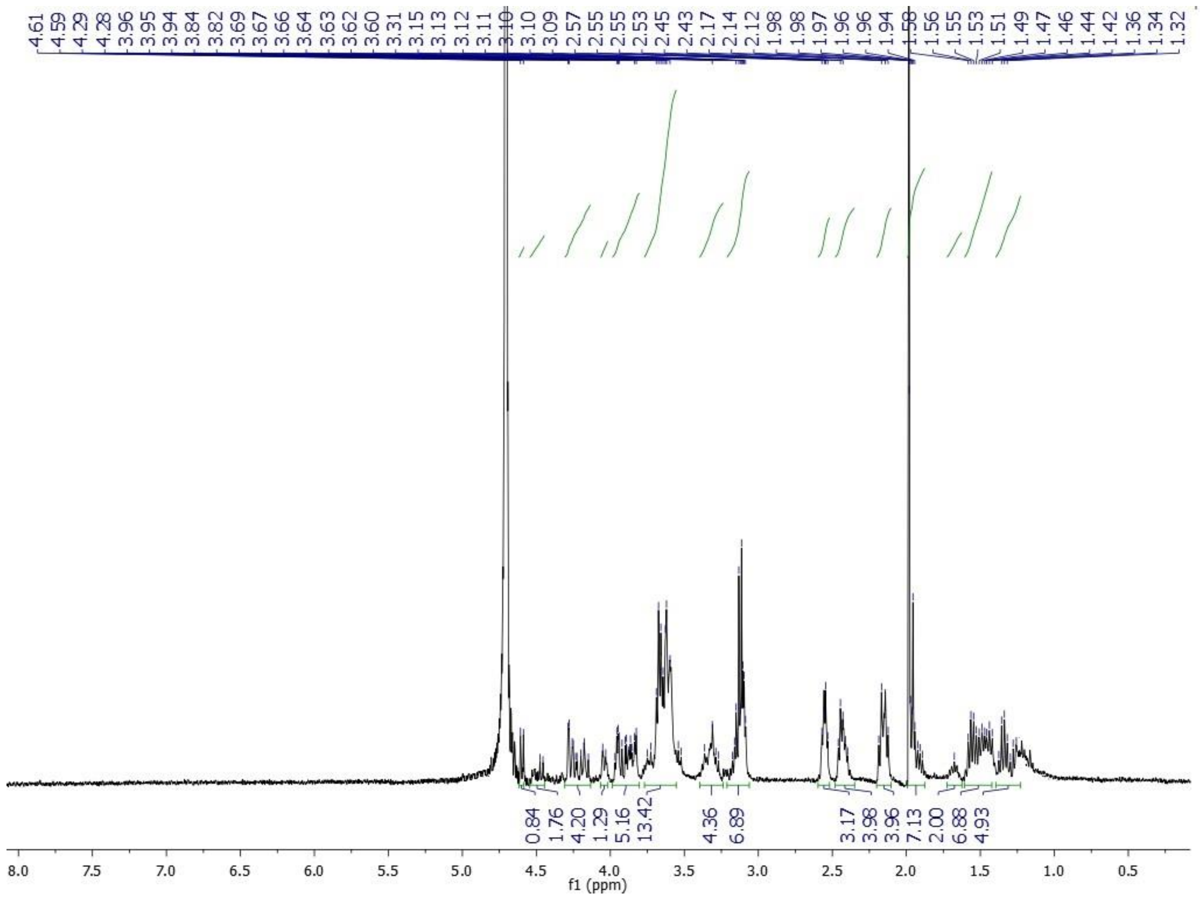
4.61 4.59 4.29 4.28 3.96 3.95 3.94 3.84 3.82 3.69 3.67 3.66 3.64 3.63 3.62 3.60 3.31 3.15 3.13 3.12 3.11 3.10 3.10 3.09 2.57 2.55 2.55 2.53 2.45 2.43 2.17 2.14 2.12 1.98 1.98 1.97 1.96 1.96 1.94 1.58 1.56 1.55 1.53 1.51 1.49 1.47 1.46 1.44 1.42 1.36 1.34 1.32
0.84 1.76 4.20 1.29 5.16 13.42 4.36 6.89 3.17 3.98 3.96 7.13 2.00 6.88 4.93
8.0 7.5 7.0 6.5 6.0 5.5 5.0 4.5 4.0 3.5 3.0 2.5 2.0 1.5 1.0 0.5
f1 (ppm)

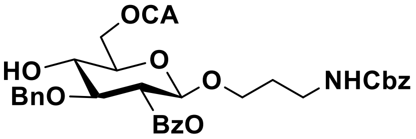
OCA
HO
O
O
NHCbz
BnO
BzO

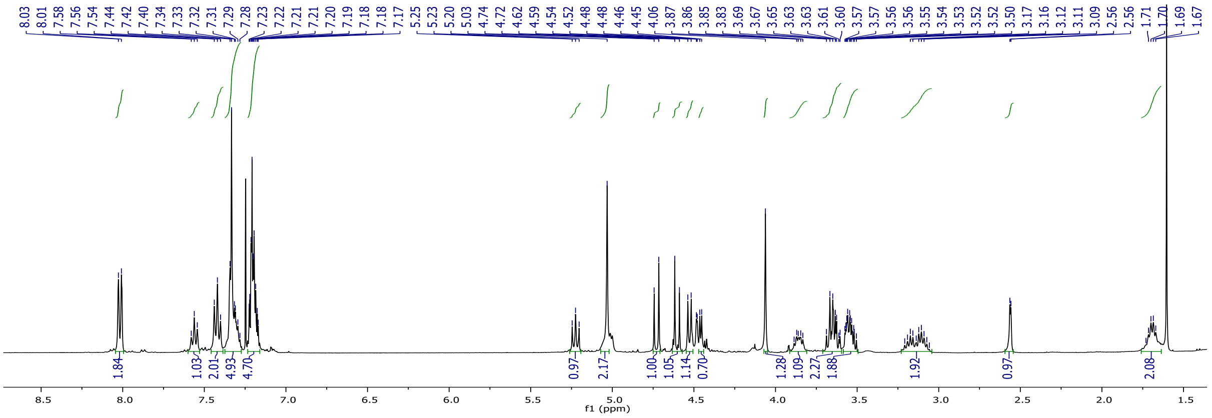
8.03
8.01
7.58
7.56
7.54
7.44
7.42
7.40
7.34
7.33
7.32
7.31
7.29
7.28
7.23
7.22
7.21
7.21
7.20
7.19
7.18
7.18
7.17
5.25
5.23
5.20
5.03
4.74
4.72
4.62
4.59
4.54
4.52
4.48
4.48
4.46
4.45
4.06
3.87
3.86
3.85
3.83
3.69
3.67
3.65
3.63
3.63
3.61
3.60
3.57
3.57
3.56
3.56
3.55
3.54
3.53
3.52
3.52
3.50
3.17
3.16
3.12
3.11
3.09
2.56
1.71
1.70
1.69
1.67
1.84
1.03
2.01
4.93
4.70
0.97
2.17
1.00
1.05
1.14
0.70
1.28
1.09
2.27
1.88
1.92
0.97
2.08
f1 (ppm)

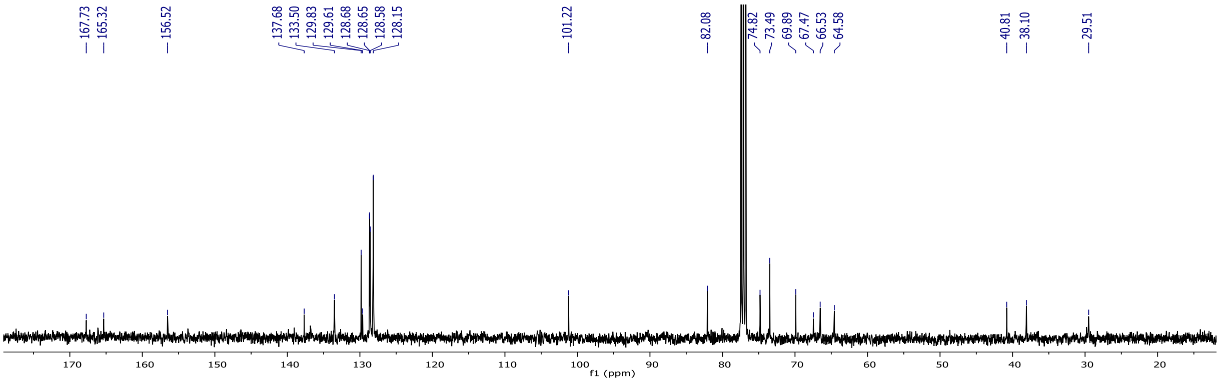
167.73
165.32
156.52
137.68
133.50
129.83
129.61
128.68
128.65
128.58
128.15
101.22
82.08
74.82
73.49
69.89
67.47
66.53
64.58
40.81
38.10
29.51
f1 (ppm)

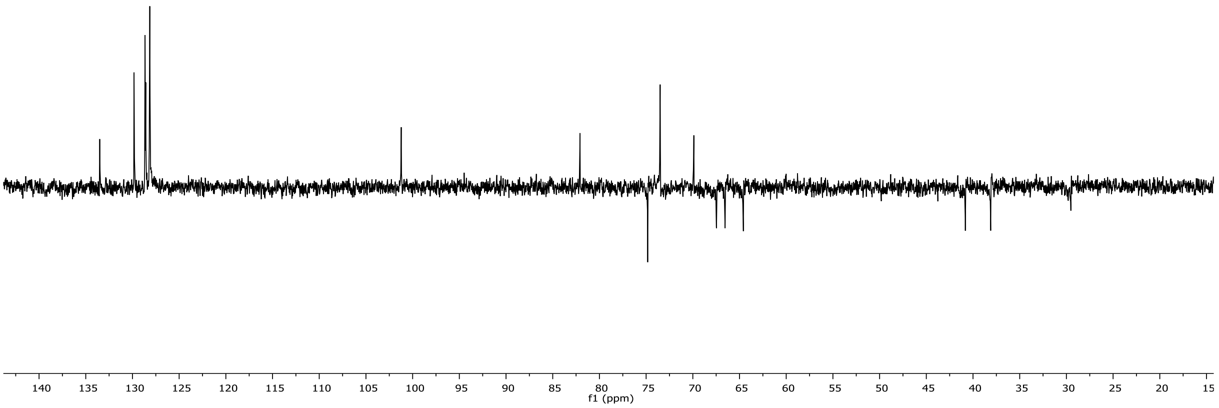
f1 (ppm)

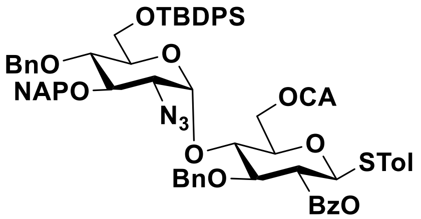
OTBDPS
BnO
O
NAPO
N3
OCA
O
O
STol
BnO
BzO

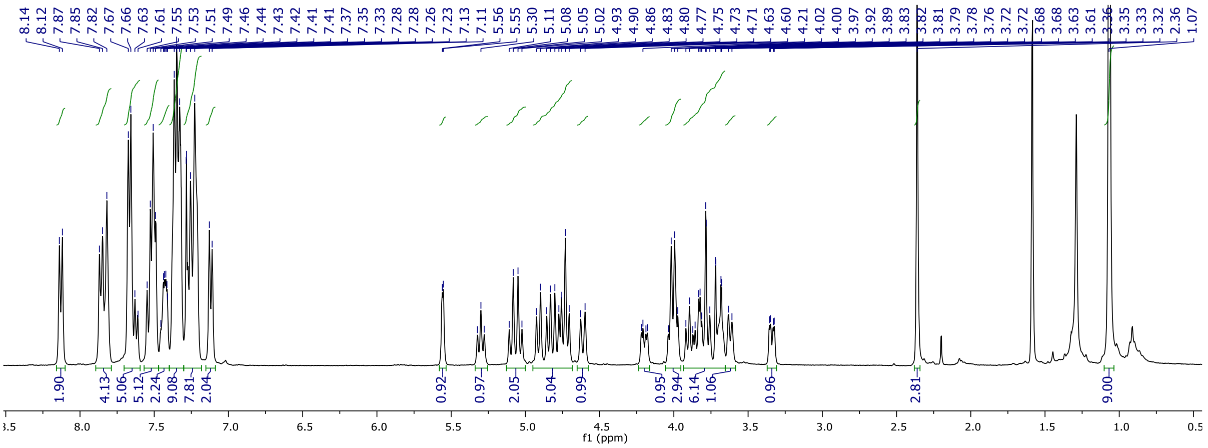
8.14 8.12 7.87 7.85 7.82 7.67 7.66 7.63 7.61 7.55 7.53 7.51 7.49 7.46 7.44 7.43 7.42 7.41 7.41 7.37 7.35 7.33 7.28 7.28 7.26 7.23 7.13 7.11 5.56 5.55 5.30 5.11 5.08 5.05 5.02 4.93 4.90 4.86 4.83 4.80 4.77 4.75 4.73 4.71 4.63 4.60 4.21 4.02 4.00 3.97 3.92 3.89 3.83 3.82 3.81 3.79 3.78 3.76 3.72 3.72 3.68 3.68 3.63 3.61 3.36 3.35 3.33 3.32 2.36 1.07
1.90 4.13 5.06 5.12 2.24 9.08 7.81 2.04 0.92 0.97 2.05 5.04 0.99 0.95 2.94 6.14 1.06 0.96 2.81 9.00
f1 (ppm)

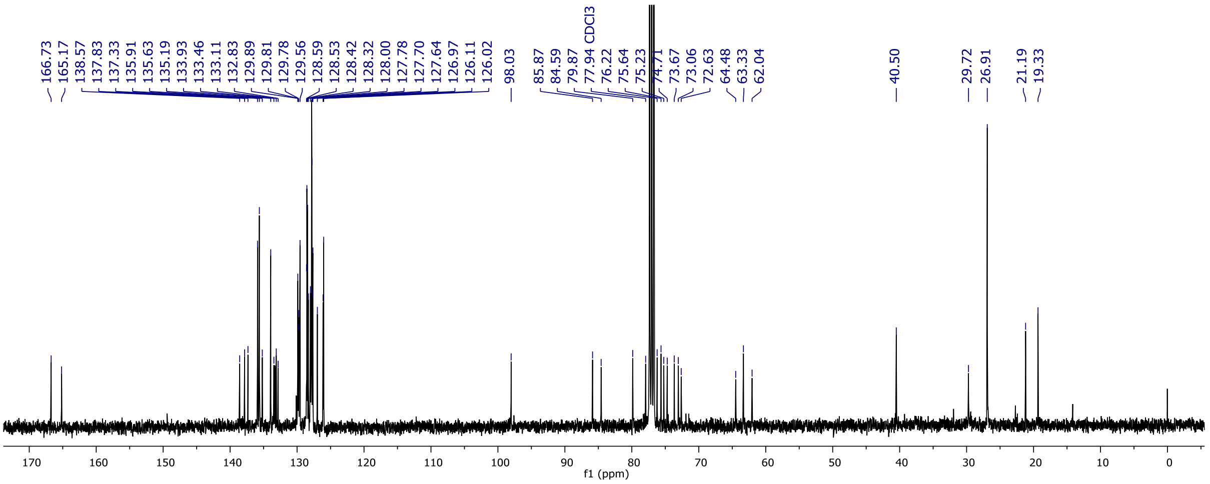
166.73 165.17 138.57 137.83 137.33 135.91 135.63 135.19 133.93 133.46 133.11 132.83 129.89 129.81 129.78 129.56 128.59 128.53 128.42 128.32 128.00 127.78 127.70 127.64 126.97 126.11 126.02 98.03 85.87 84.59 79.87 77.94 CDCl3 76.22 75.64 75.23 74.71 73.67 73.06 72.63 64.48 63.33 62.04 40.50 29.72 26.91 21.19 19.33
f1 (ppm)

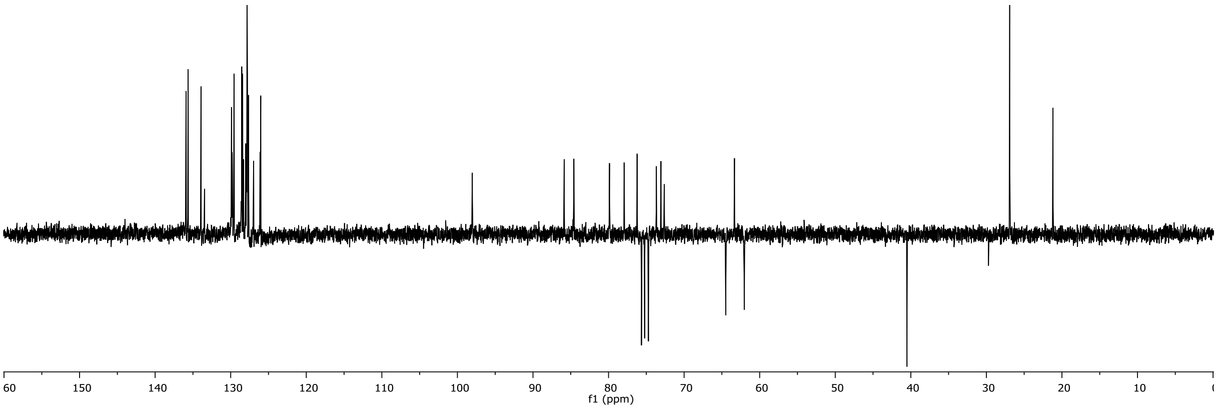
f1 (ppm)

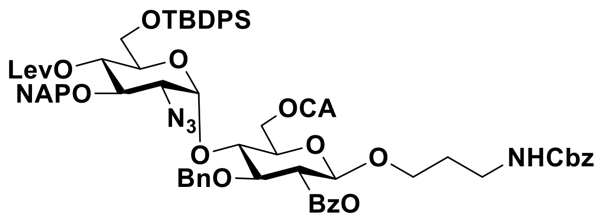
OTBDPS
LevO
NAPO
O
N3
O
OCA
O
BnO
BzO
O
NHCbz

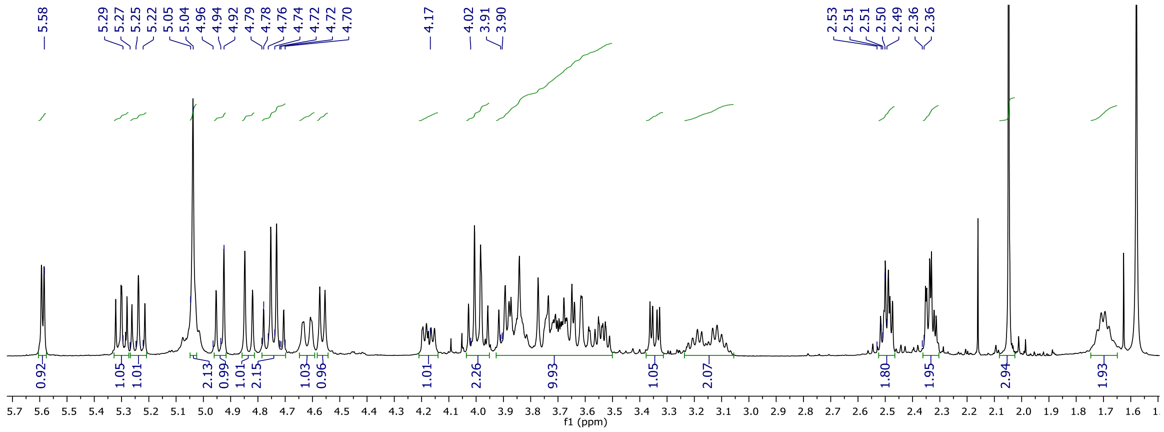
5.58
5.29 5.27 5.25 5.22 5.05 5.04 4.96 4.94 4.92 4.79 4.78 4.76 4.74 4.72 4.72 4.70
4.17
4.02 3.91 3.90
2.53 2.51 2.51 2.50 2.49 2.36 2.36
0.92 1.05 1.01 2.13 0.99 1.01 2.15 1.03 0.96 1.01 2.26 9.93 1.05 2.07 1.80 1.95 2.94 1.93
f1 (ppm)

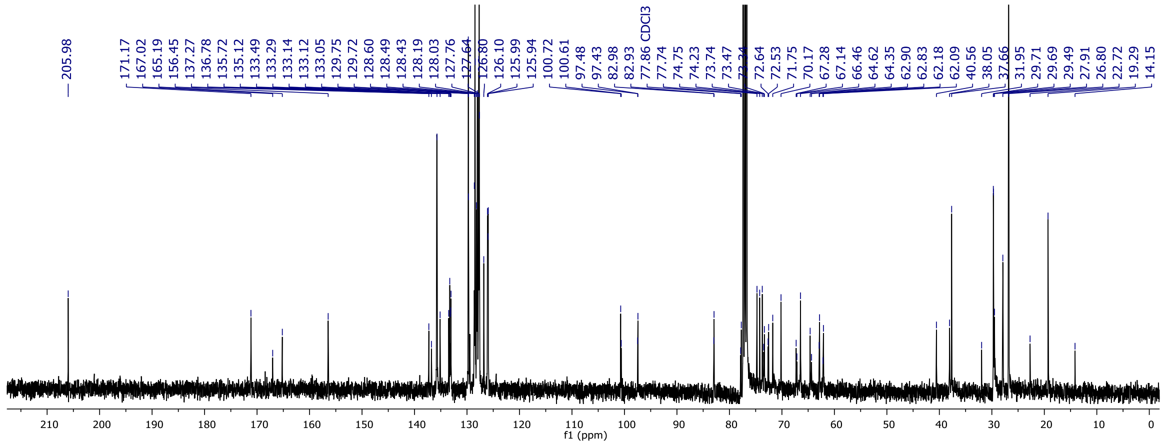
205.98
171.17 167.02 165.19 156.45 137.27 136.78 135.72 135.12 133.49 133.29 133.14 133.12 133.05 129.75 129.72 128.60 128.49 128.43 128.19 128.03 127.76 127.64 126.80 126.10 125.99 125.94
100.72 100.61 97.48 97.43 82.98 82.93 77.86 CDCl3 77.74 74.75 74.23 73.74 73.47
72.64 72.53 71.75 70.17 67.28 67.14 66.46 64.62 64.35 62.90 62.83 62.18 62.09 40.56 38.05 37.66 31.95 29.71 29.69 29.49 27.91 26.80 22.72 19.29 14.15
f1 (ppm)

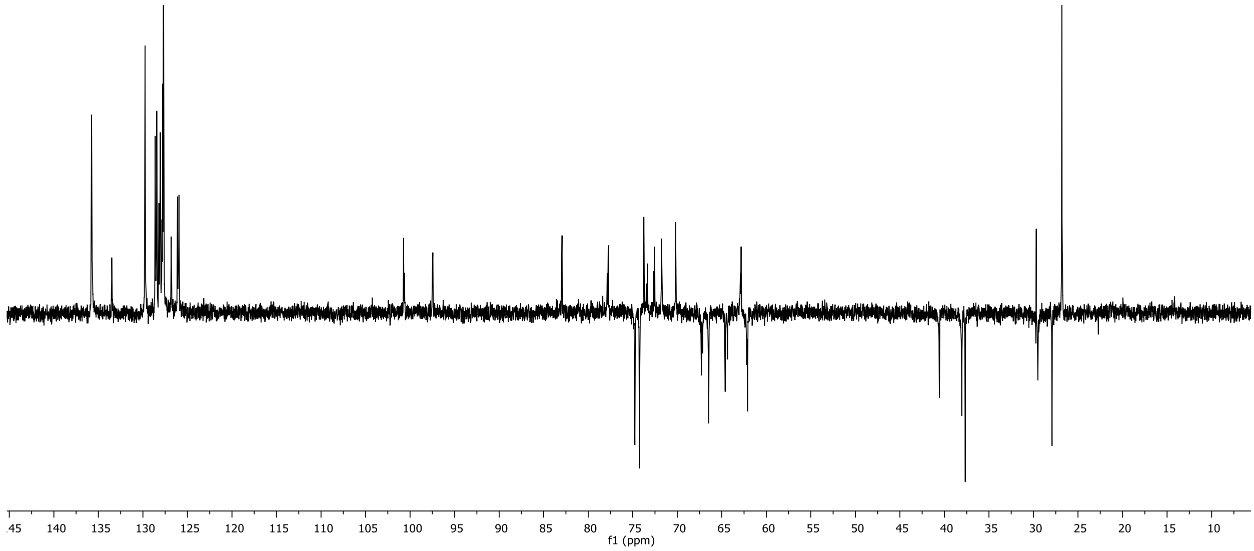
f1 (ppm)

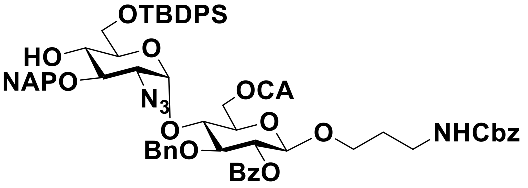
OTBDPS
HO
O
NAPO
N3
OCA
O
BnO
O
BzO
O
NHCbz

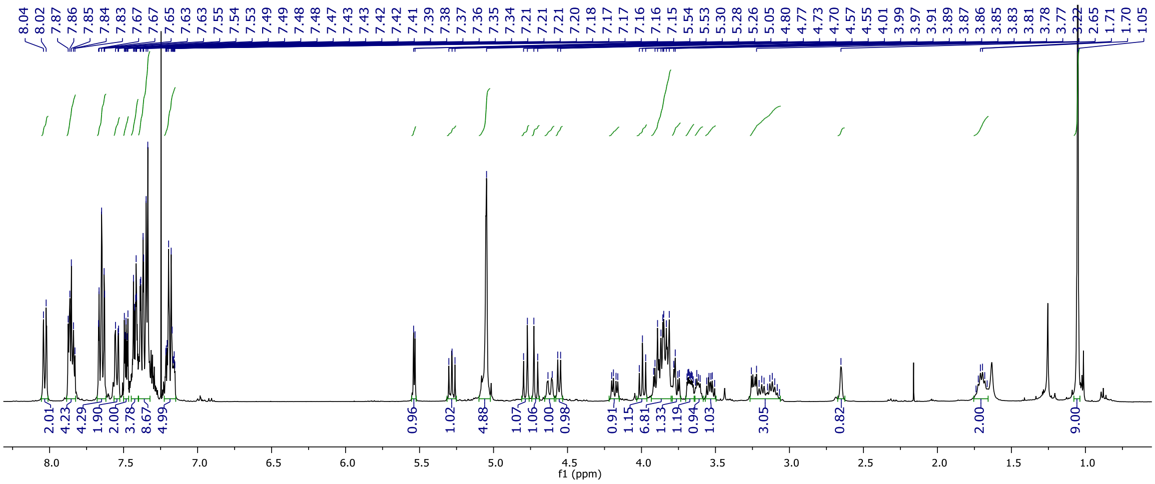
8.04 8.02 7.87 7.86 7.85 7.84 7.83 7.67 7.65 7.63 7.63 7.55 7.54 7.53 7.49 7.49 7.48 7.47 7.43 7.43 7.42 7.42 7.41 7.39 7.38 7.37 7.36 7.35 7.34 7.21 7.21 7.21 7.20 7.18 7.17 7.17 7.16 7.16 7.15 5.54 5.53 5.30 5.28 5.26 5.05 4.80 4.77 4.73 4.70 4.57 4.55 4.01 3.99 3.97 3.91 3.89 3.87 3.86 3.85 3.83 3.81 3.78 3.77 3.22 2.65 1.71 1.70 1.05
2.01 4.23 4.29 1.90 2.00 3.78 8.67 4.99 0.96 1.02 4.88 1.07 1.06 1.00 0.98 0.91 1.15 6.81 1.33 1.19 0.94 1.03 3.05 0.82 2.00 9.00
8.0 7.5 7.0 6.5 6.0 5.5 5.0 4.5 4.0 3.5 3.0 2.5 2.0 1.5 1.0
f1 (ppm)

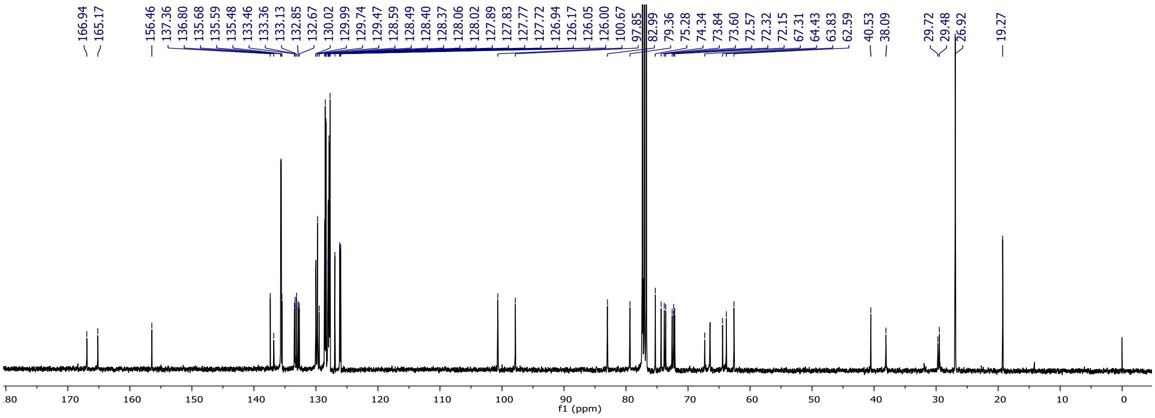
166.94 165.17 156.46 137.36 136.80 135.68 135.59 135.48 133.46 133.36 133.13 132.85 132.67 130.02 129.99 129.74 129.47 129.47 128.59 128.49 128.40 128.37 128.06 128.02 127.89 127.83 127.77 127.72 126.94 126.17 126.05 126.00 100.67 97.85 82.99 79.36 75.28 74.34 73.84 73.60 72.57 72.32 72.15 67.31 64.43 63.83 62.59 40.53 38.09 29.72 29.48 26.92 19.27
180 170 160 150 140 130 120 110 100 90 80 70 60 50 40 30 20 10 0
f1 (ppm)

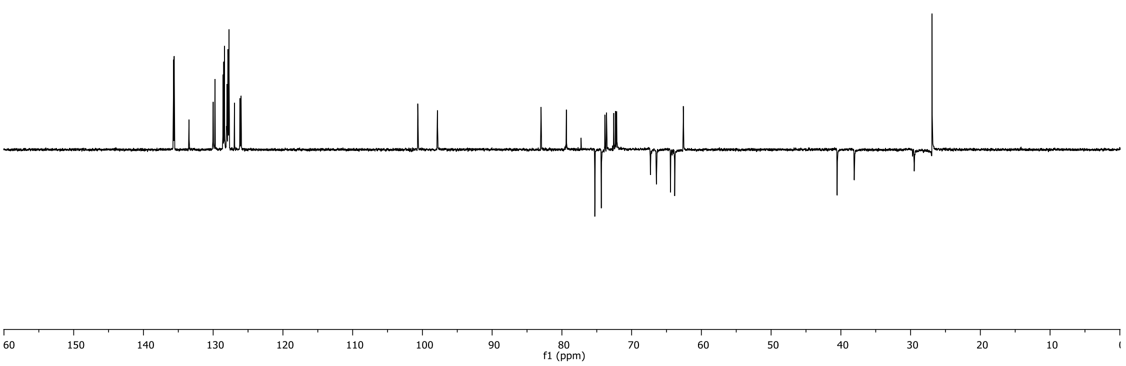
160 150 140 130 120 110 100 90 80 70 60 50 40 30 20 10
f1 (ppm)

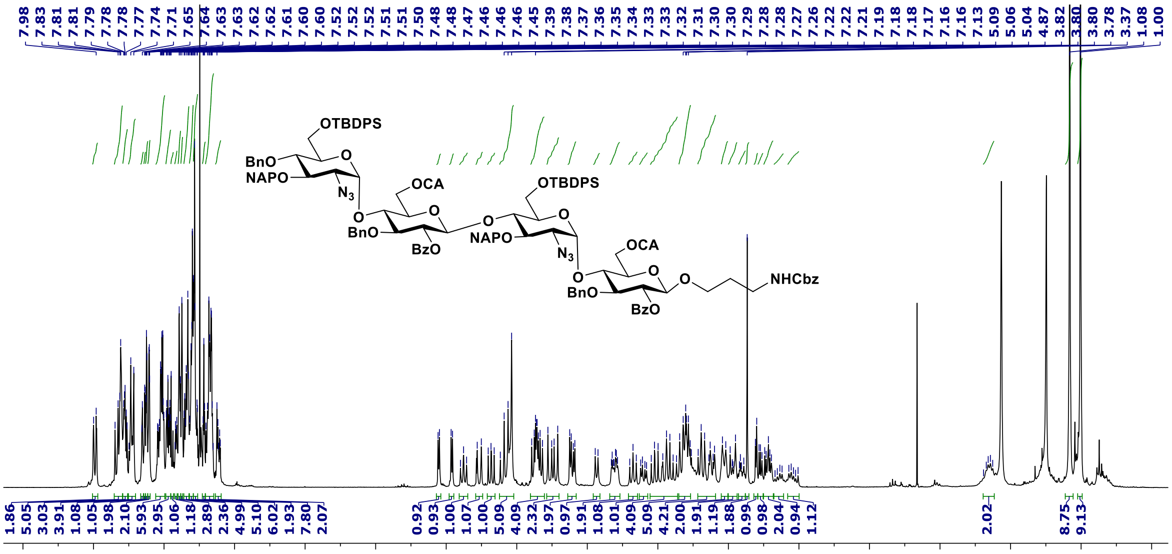
7.98 7.83 7.81 7.79 7.78 7.77 7.74 7.71 7.65 7.64 7.63 7.62 7.61 7.60 7.52 7.52 7.51 7.51 7.50 7.48 7.47 7.46 7.46 7.45 7.39 7.38 7.37 7.36 7.35 7.34 7.33 7.33 7.32 7.31 7.30 7.30 7.29 7.28 7.28 7.27 7.26 7.22 7.22 7.21 7.19 7.18 7.18 7.17 7.16 7.13 5.09 5.06 5.04 4.87 3.82 3.80 3.80 3.78 3.37 1.08 1.00
OTBDPS
BnO
NAPO
N3
O
OCA
BnO
BzO
OTBDPS
NAPO
N3
OCA
BnO
BzO
NHCbz
1.86 5.05 3.03 3.91 1.08 1.05 1.98 2.10 5.93 2.95 1.06 1.18 2.89 2.36 4.99 5.10 6.02 1.93 7.80 2.07
0.92 0.93 1.00 1.07 1.00 5.09 4.09 2.32 1.97 0.97 1.91 1.08 1.01 4.09 5.09 4.21 2.00 1.91 1.19 1.88 0.99 0.98 2.04 0.94 1.12
2.02 8.75 9.13
f1 (ppm)

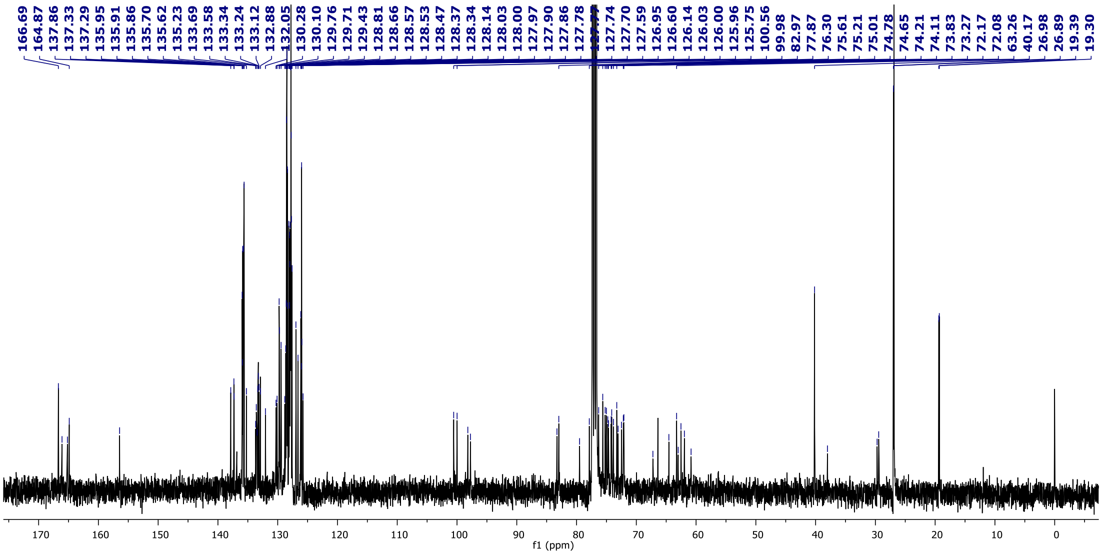
166.69 164.87 137.86 137.33 137.29 135.95 135.91 135.86 135.70 135.62 135.23 133.69 133.58 133.34 133.24 133.12 132.88 132.05 130.28 130.10 129.76 129.71 129.43 128.81 128.66 128.53 128.47 128.37 128.34 128.14 128.03 128.00 127.97 127.90 127.86 127.78 127.74 127.70 127.59 126.95 126.60 126.14 126.03 126.00 125.96 125.75 100.56 99.98 82.97 77.87 76.30 75.61 75.21 75.01 74.78 74.65 74.21 74.11 73.83 73.27 72.17 72.08 63.26 40.17 26.98 26.89 19.39 19.30
f1 (ppm)

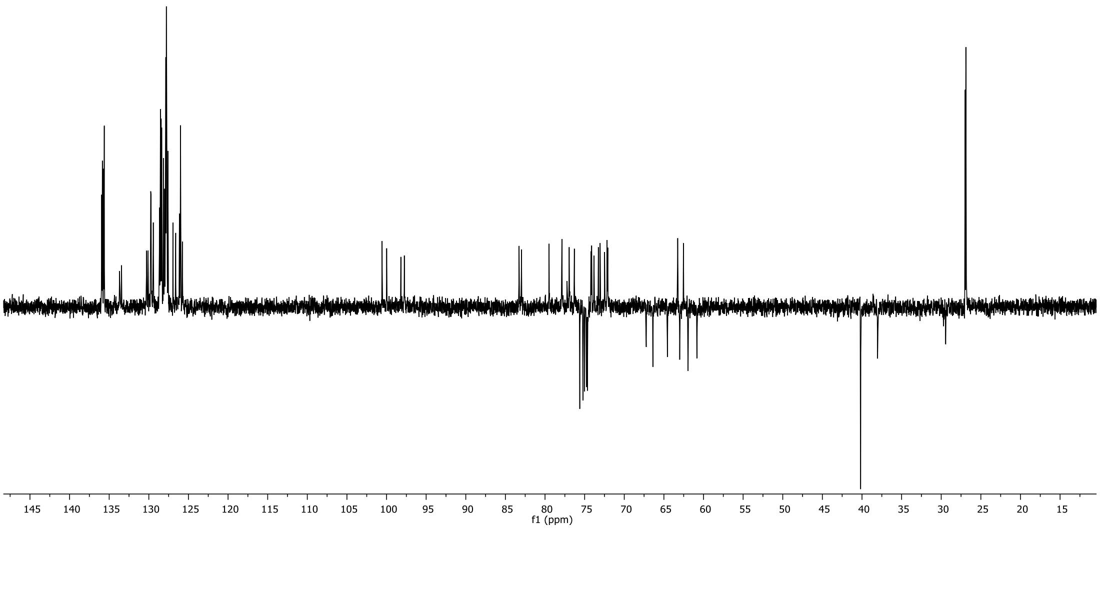
f1 (ppm)

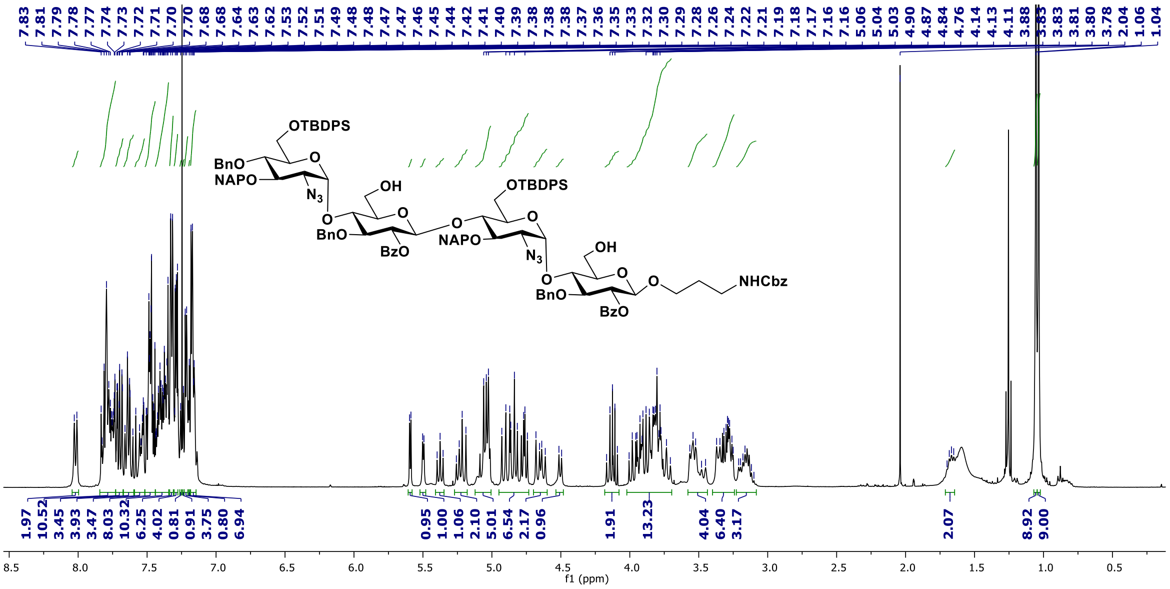
7.83 7.81 7.79 7.78 7.77 7.74 7.73 7.72 7.71 7.70 7.70 7.68 7.64 7.63 7.62 7.53 7.52 7.51 7.49 7.48 7.48 7.47 7.46 7.45 7.44 7.42 7.41 7.40 7.39 7.38 7.38 7.38 7.37 7.36 7.35 7.33 7.32 7.30 7.29 7.28 7.26 7.24 7.22 7.21 7.19 7.18 7.17 7.16 7.16 5.06 5.04 5.03 4.90 4.87 4.84 4.76 4.14 4.13 4.11 3.88 3.85 3.83 3.81 3.80 3.78 2.04 1.06 1.04
OTBDPS
BnO
NAPO
N_3
O
OH
BnO
BzO
OTBDPS
NAPO
N_3
OH
O
BnO
BzO
NHCbz
1.97 10.52 3.45 3.93 3.47 8.03 10.32 6.25 4.02 0.81 0.91 3.75 0.80 6.94
0.95 1.00 1.06 2.10 5.01 6.54 2.17 0.96
1.91 13.23 4.04 6.40 3.17 2.07 8.92 9.00
f1 (ppm)

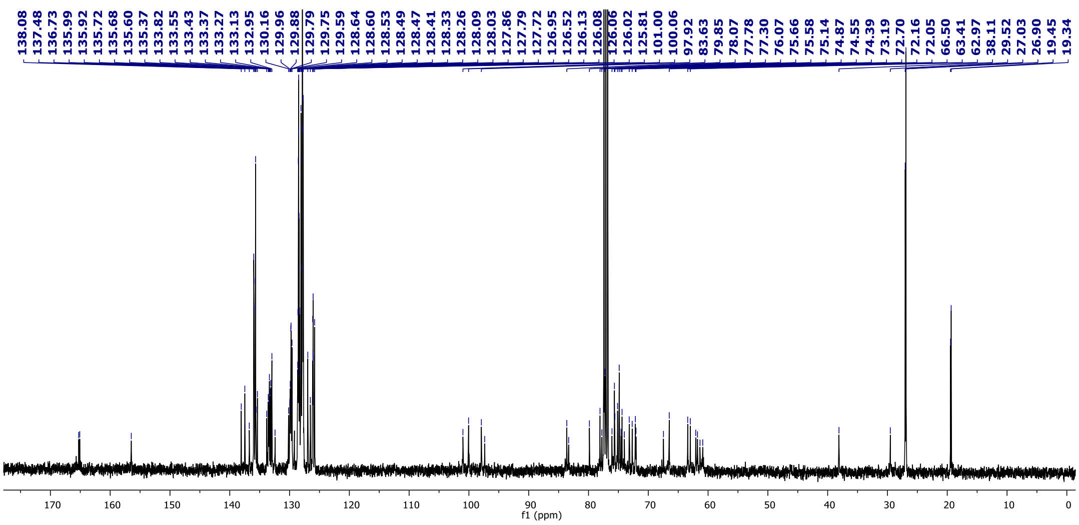
138.08 137.48 136.73 135.99 135.92 135.72 135.68 135.60 135.37 133.82 133.55 133.43 133.37 133.27 133.13 132.95 130.16 129.96 129.88 129.79 129.75 129.59 128.64 128.60 128.53 128.49 128.47 128.41 128.33 128.26 128.09 128.03 127.86 127.79 127.72 126.95 126.52 126.13 126.08 126.06 126.02 125.81 101.00 100.06 97.92 83.63 79.85 78.07 77.78 77.30 76.07 75.66 75.58 75.14 74.87 74.55 74.39 73.19 72.70 72.16 72.05 66.50 63.41 62.97 38.11 29.52 27.03 26.90 19.45 19.34
f1 (ppm)

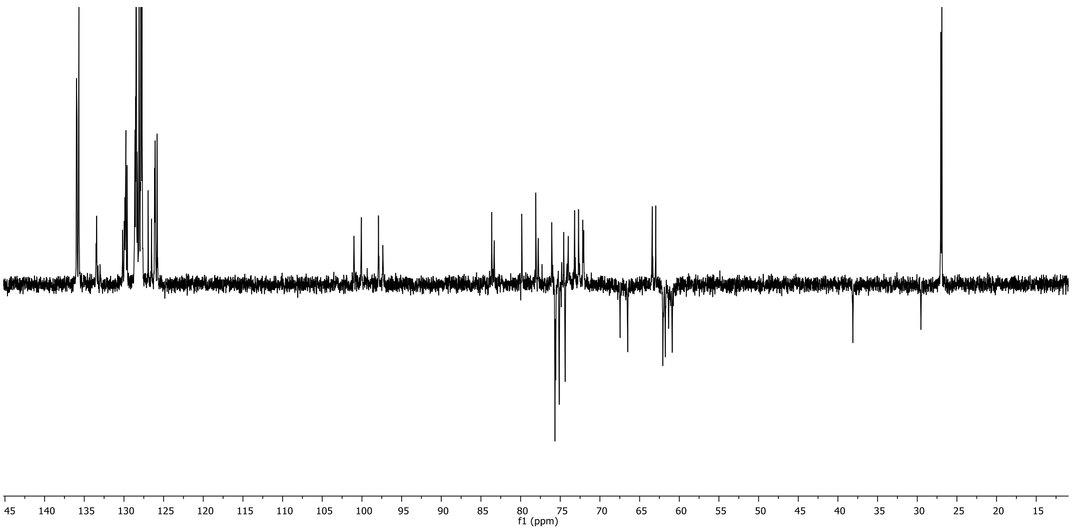
f1 (ppm)

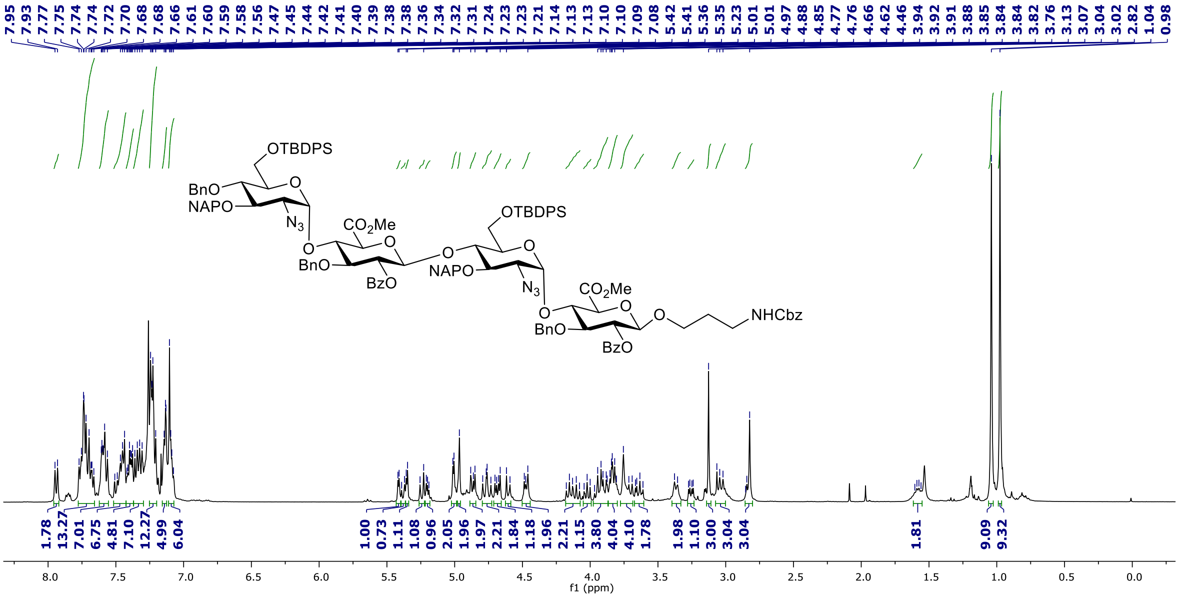
7.95 7.93 7.77 7.75 7.74 7.74 7.72 7.70 7.68 7.66 7.61 7.60 7.59 7.58 7.56 7.47 7.45 7.44 7.42 7.41 7.40 7.39 7.38 7.38 7.36 7.34 7.32 7.31 7.24 7.23 7.23 7.21 7.14 7.13 7.13 7.10 7.10 7.09 7.08 5.42 5.41 5.36 5.35 5.23 5.01 5.01 4.97 4.88 4.85 4.77 4.76 4.66 4.62 4.46 3.94 3.92 3.91 3.88 3.85 3.84 3.84 3.82 3.76 3.13 3.07 3.04 3.02 2.82 1.04 0.98
OTBDPS
BnO
NAPO
N3
O
CO2Me
BnO
BzO
NAPO
OTBDPS
N3
CO2Me
BnO
BzO
NHCbz
1.78 13.27 7.01 6.75 4.81 7.10 12.27 4.99 6.04 1.00 0.73 1.11 1.08 0.96 2.05 1.96 1.97 2.21 1.84 1.18 1.96 2.21 1.15 3.80 4.04 4.10 1.78 1.98 1.10 3.00 3.04 3.04 1.81 9.09 9.32
f1 (ppm)

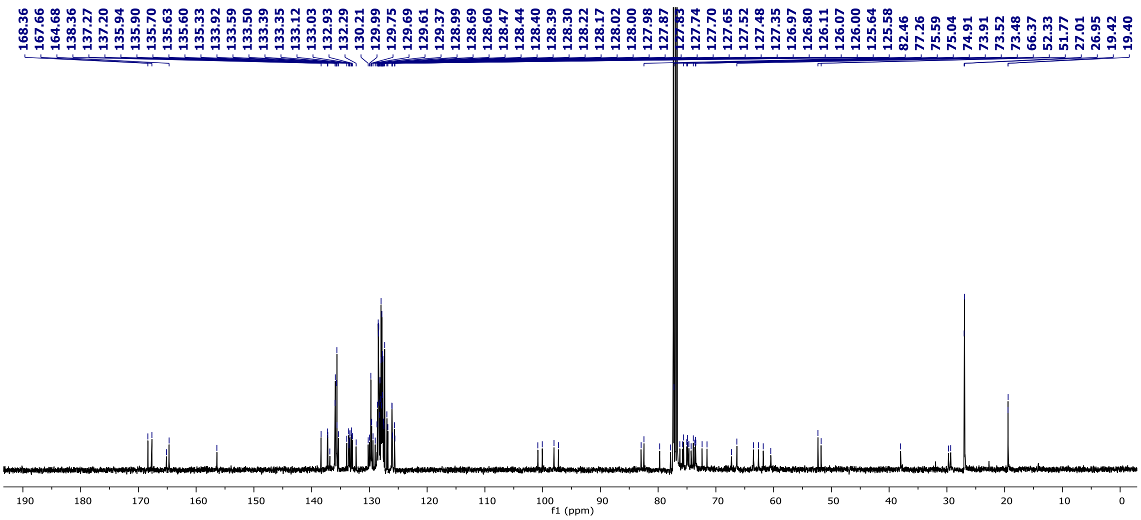
168.36 167.66 164.68 138.36 137.27 137.20 135.94 135.90 135.70 135.63 135.60 135.33 133.92 133.59 133.50 133.39 133.35 133.12 133.03 132.93 132.29 130.21 129.99 129.75 129.69 129.61 129.37 128.99 128.69 128.60 128.47 128.44 128.40 128.39 128.30 128.22 128.17 128.02 128.00 127.98 127.87 127.83 127.74 127.70 127.65 127.52 127.48 127.35 126.97 126.80 126.11 126.07 126.00 125.64 125.58 82.46 77.26 75.59 75.04 74.91 73.91 73.52 73.48 66.37 52.33 51.77 27.01 26.95 19.42 19.40
f1 (ppm)

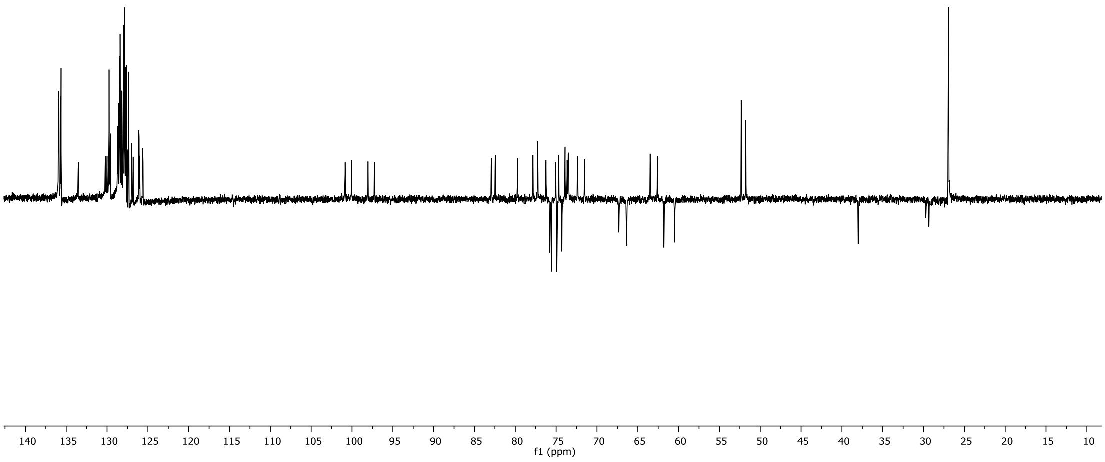
f1 (ppm)

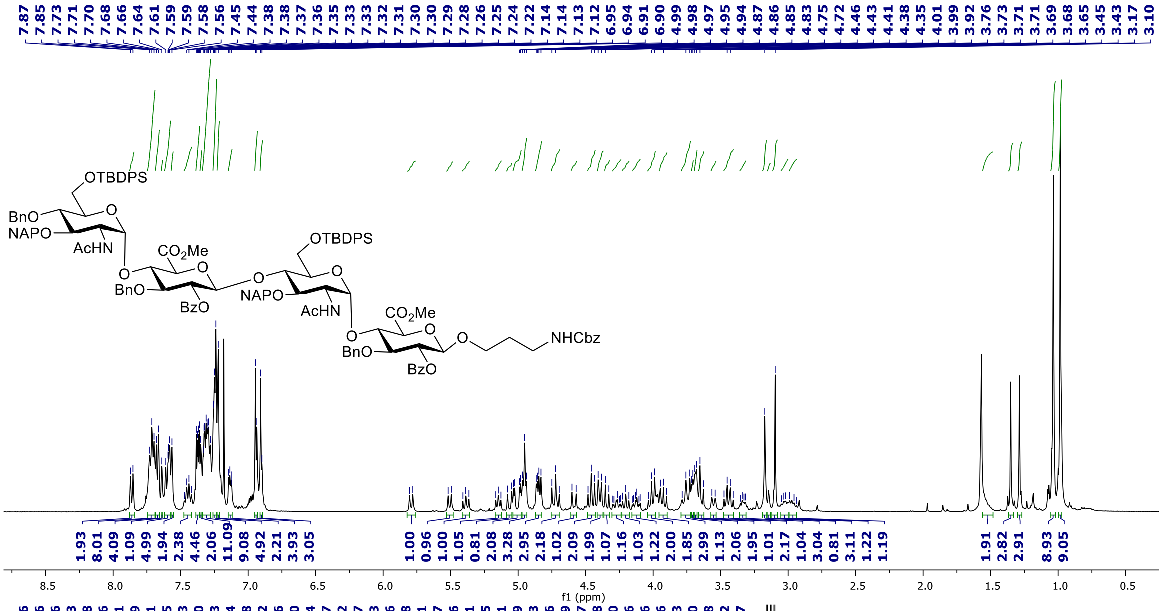
7.87 7.85 7.73 7.71 7.70 7.68 7.66 7.64 7.61 7.59 7.59 7.58 7.56 7.45 7.44 7.38 7.38 7.37 7.36 7.35 7.33 7.33 7.32 7.31 7.30 7.29 7.28 7.26 7.25 7.24 7.22 7.14 7.14 7.13 7.12 6.95 6.94 6.91 6.90 4.99 4.98 4.97 4.95 4.94 4.87 4.86 4.85 4.83 4.75 4.72 4.46 4.43 4.41 4.38 4.35 4.01 3.99 3.92 3.76 3.73 3.71 3.69 3.68 3.65 3.45 3.43 3.17 3.10
OTBDPS
BnO
NAPO
AcHN
O
CO_2Me
BnO
BzO
OTBDPS
NAPO
AcHN
CO_2Me
BnO
BzO
NHCbz
1.93 8.01 4.09 1.09 4.99 1.94 2.38 4.46 2.06 11.09 9.08 4.92 2.21 3.93 3.05 1.00 0.96 1.00 1.05 0.81 2.08 3.28 2.95 2.18 1.02 2.09 1.99 1.07 1.16 1.03 1.22 2.00 1.85 2.99 1.13 2.06 1.95 1.01 2.17 1.04 3.04 0.81 3.11 1.22 1.19 1.91 2.82 2.91 8.93 9.05
f1 (ppm)

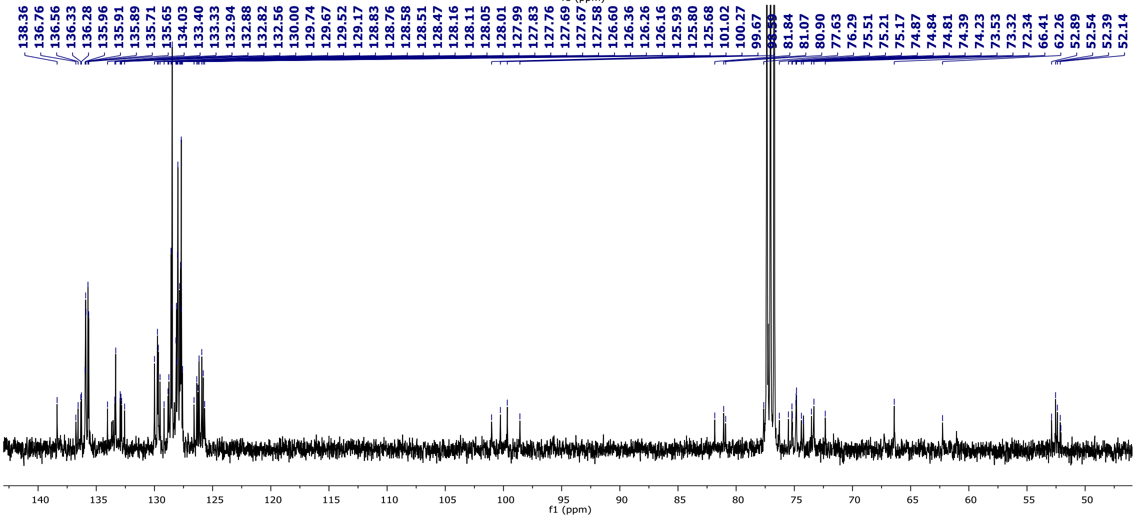
138.36 136.76 136.33 136.28 135.96 135.91 135.89 135.71 135.65 134.03 133.40 133.33 132.94 132.88 132.82 132.56 130.00 129.74 129.67 129.52 129.17 128.83 128.76 128.58 128.51 128.47 128.16 128.11 128.05 128.01 127.99 127.83 127.76 127.69 127.67 127.58 126.60 126.36 126.26 126.16 125.93 125.80 125.68 101.02 100.27 99.67 81.84 81.07 80.90 77.63 76.29 75.51 75.21 75.17 74.87 74.84 74.81 74.39 74.23 73.53 73.32 72.34 66.41 62.26 52.89 52.54 52.39 52.14
f1 (ppm)

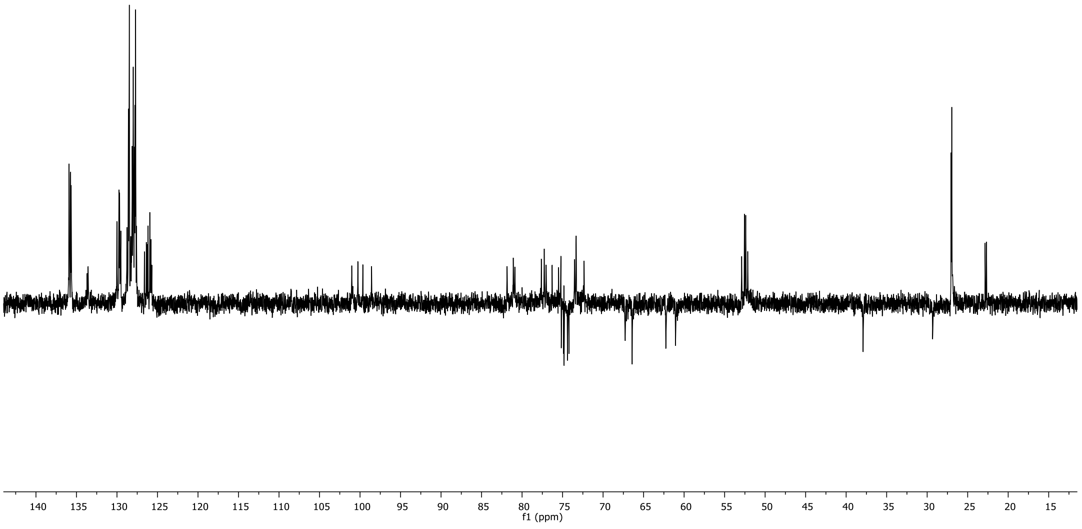
f1 (ppm)

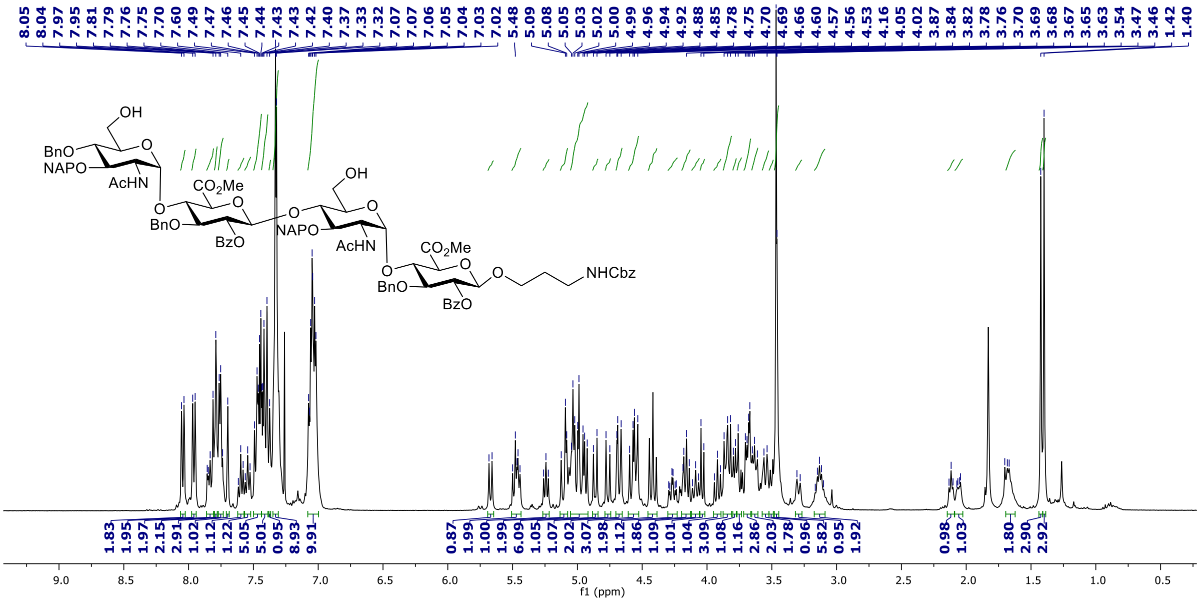
8.05 8.04 7.97 7.95 7.81 7.79 7.76 7.75 7.70 7.60 7.49 7.47 7.46 7.45 7.44 7.43 7.43 7.42 7.40 7.37 7.33 7.32 7.07 7.06 7.05 7.04 7.03 7.02 5.48 5.09 5.08 5.05 5.03 5.02 5.00 4.99 4.96 4.94 4.92 4.88 4.85 4.78 4.75 4.70 4.69 4.66 4.60 4.57 4.56 4.53 4.16 4.05 4.02 3.87 3.84 3.82 3.78 3.76 3.70 3.69 3.68 3.67 3.65 3.63 3.54 3.47 3.46 1.42 1.40
OH
BnO
NAPO
AcHN
CO_2Me
BnO
BzO
OH
NAPO
AcHN
CO_2Me
BnO
BzO
NHCbz
1.83 1.95 1.97 2.15 2.91 1.02 1.12 1.22 5.05 5.01 0.95 8.93 9.91 0.87 1.99 1.00 6.09 1.05 1.07 2.02 3.07 1.98 1.12 1.86 1.09 1.01 1.04 3.09 1.08 1.16 2.86 2.03 1.78 0.96 5.82 0.95 1.92 0.98 1.03 1.80 2.90 2.92
9.0 8.5 8.0 7.5 7.0 6.5 6.0 5.5 5.0 4.5 4.0 3.5 3.0 2.5 2.0 1.5 1.0 0.5
f1 (ppm)

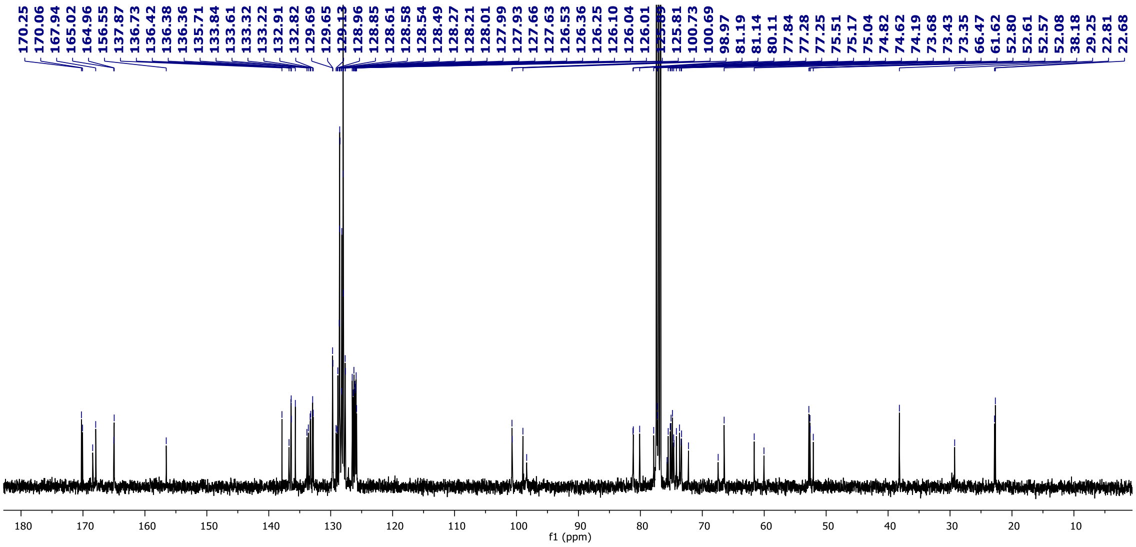
170.25 170.06 167.94 165.02 164.96 156.55 137.87 136.73 136.42 136.38 135.71 133.84 133.61 133.32 133.22 132.91 132.82 129.69 129.65 129.13 128.96 128.85 128.61 128.58 128.54 128.49 128.27 128.21 128.01 127.99 127.93 127.66 127.63 126.53 126.36 126.25 126.10 126.04 126.01 125.89 125.81 100.73 100.69 98.97 81.19 81.14 80.11 77.84 77.28 77.25 75.51 75.17 75.04 74.82 74.62 74.19 73.68 73.43 73.35 66.47 61.62 52.80 52.61 52.57 52.08 38.18 29.25 22.81 22.68
180 170 160 150 140 130 120 110 100 90 80 70 60 50 40 30 20 10
f1 (ppm)

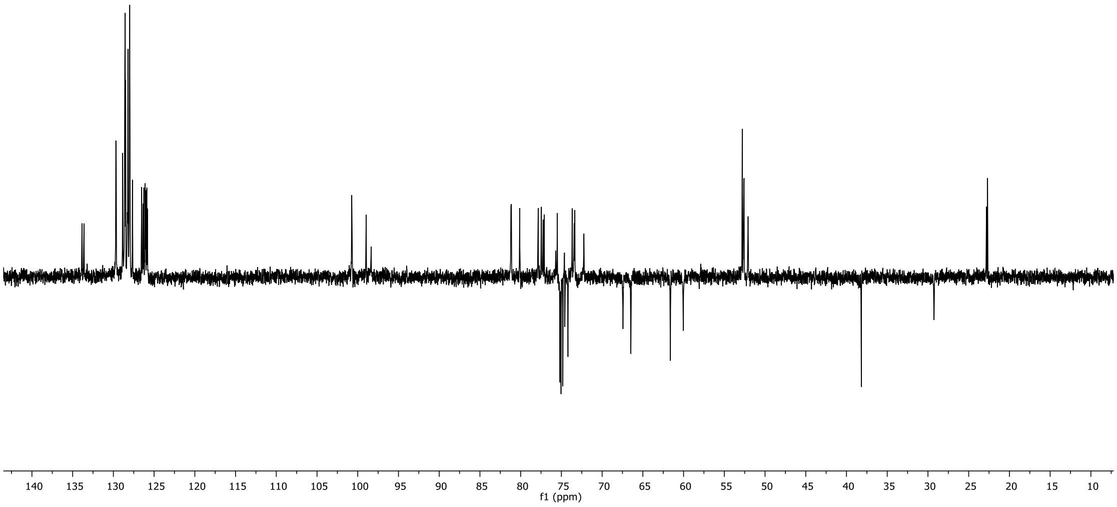
140 135 130 125 120 115 110 105 100 95 90 85 80 75 70 65 60 55 50 45 40 35 30 25 20 15 10
f1 (ppm)

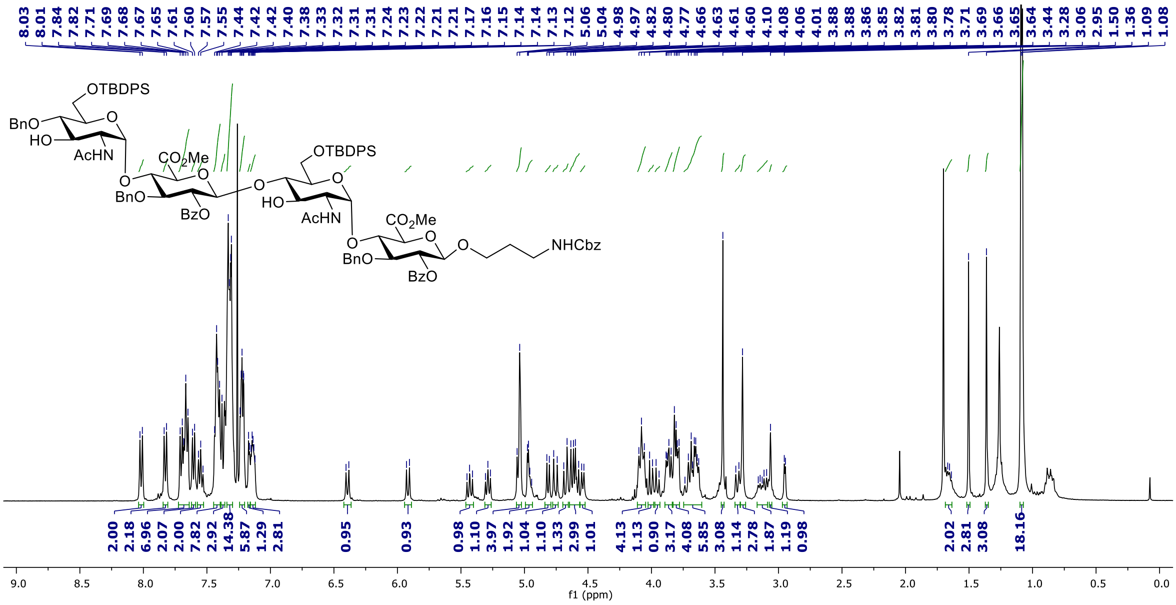
8.03 8.01 7.84 7.82 7.71 7.69 7.68 7.67 7.65 7.61 7.60 7.57 7.55 7.44 7.42 7.40 7.38 7.33 7.32 7.31 7.24 7.23 7.22 7.21 7.17 7.16 7.15 7.14 7.13 7.12 5.06 5.04 4.98 4.97 4.82 4.80 4.77 4.66 4.63 4.61 4.60 4.10 4.08 4.06 4.01 3.88 3.86 3.85 3.82 3.81 3.80 3.78 3.71 3.69 3.66 3.65 3.64 3.44 3.28 3.06 2.95 1.50 1.36 1.09 1.08
OTBDPS
BnO
HO
AcHN
CO2Me
O
BnO
BzO
OTBDPS
HO
AcHN
CO2Me
BnO
BzO
NHCbz
2.00 2.18 6.96 2.07 2.00 7.82 2.92 14.38 5.87 1.29 2.81 0.95 0.93 0.98 1.10 3.97 1.92 1.04 1.10 1.33 2.99 1.01 4.13 1.13 0.90 3.17 4.08 5.85 3.08 1.14 2.78 1.87 1.19 0.98 2.02 2.81 3.08 18.16
9.0 8.5 8.0 7.5 7.0 6.5 6.0 5.5 5.0 4.5 4.0 3.5 3.0 2.5 2.0 1.5 1.0 0.5 0.0
f1 (ppm)

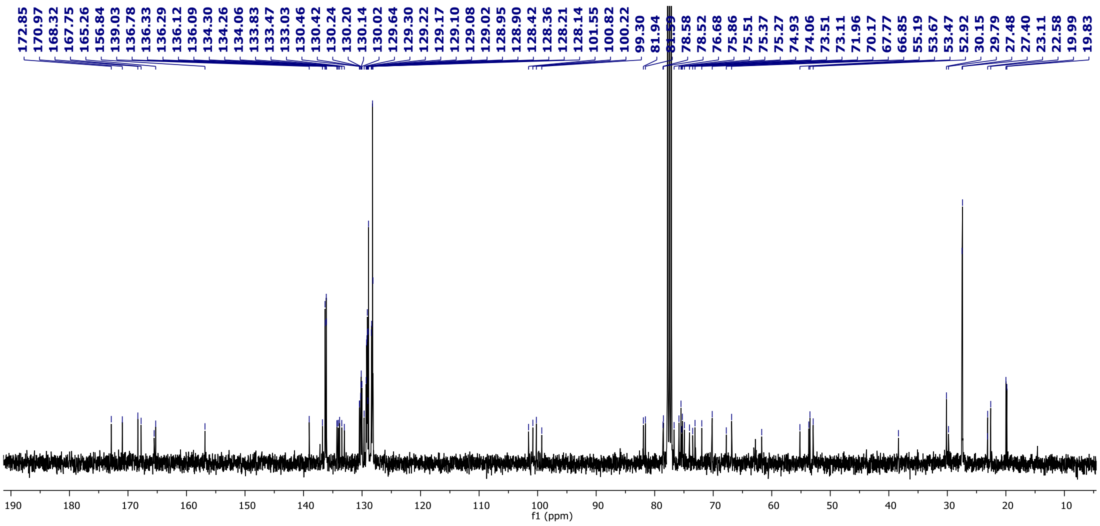
172.85 170.97 168.32 167.75 165.26 156.84 139.03 136.78 136.33 136.29 136.12 136.09 134.30 134.26 134.06 133.83 133.47 133.03 130.46 130.42 130.24 130.20 130.14 130.02 129.64 129.30 129.22 129.17 129.10 129.08 129.02 128.95 128.90 128.42 128.36 128.21 128.14 101.55 100.82 100.22 99.30 81.94 [illegible] 78.58 78.52 76.68 75.86 75.51 75.37 75.27 74.93 74.06 73.51 73.11 71.96 70.17 67.77 66.85 55.19 53.67 53.47 52.92 30.15 29.79 27.48 27.40 23.11 22.58 19.99 19.83
190 180 170 160 150 140 130 120 110 100 90 80 70 60 50 40 30 20 10
f1 (ppm)

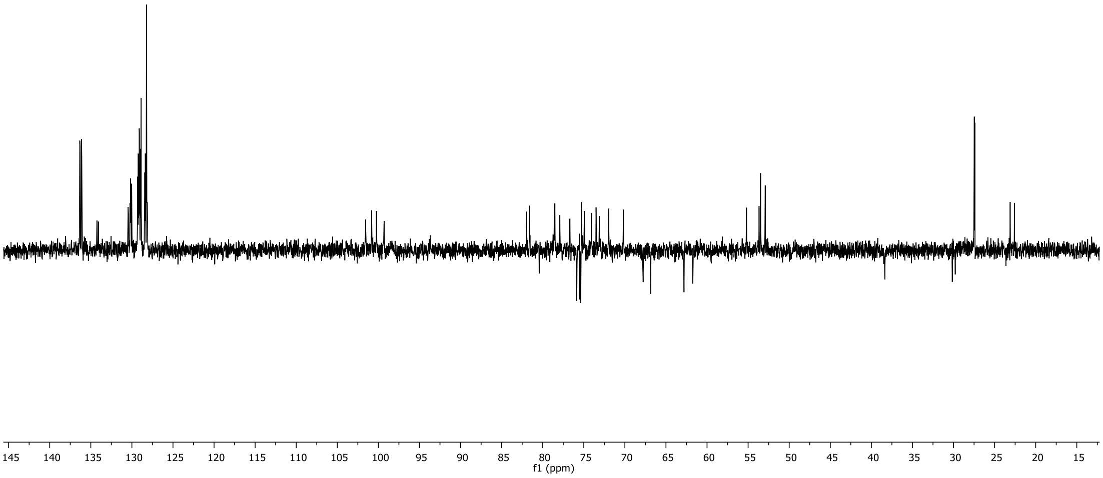
145 140 135 130 125 120 115 110 105 100 95 90 85 80 75 70 65 60 55 50 45 40 35 30 25 20 15
f1 (ppm)

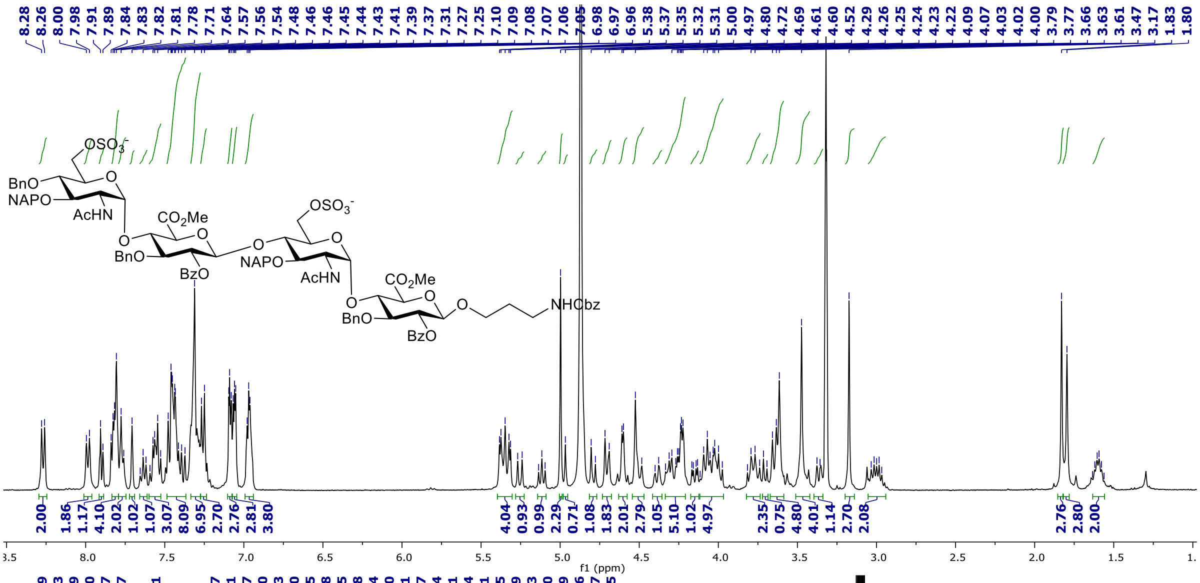
8.28 8.26 8.00 7.98 7.91 7.89 7.84 7.83 7.82 7.81 7.78 7.71 7.64 7.57 7.56 7.54 7.48 7.46 7.45 7.44 7.43 7.41 7.39 7.37 7.31 7.27 7.25 7.10 7.09 7.08 7.07 7.06 7.05 6.98 6.97 6.96 5.38 5.37 5.35 5.32 5.31 5.00 4.97 4.80 4.72 4.69 4.61 4.60 4.52 4.29 4.26 4.25 4.24 4.23 4.22 4.09 4.07 4.03 4.02 4.00 3.79 3.77 3.66 3.63 3.61 3.47 3.17 1.83 1.80
OSO3-
BnO
NAPO
AcHN
CO2Me
O
BnO
BzO
NAPO
OSO3-
AcHN
CO2Me
BnO
BzO
NHCbz
2.00 1.86 1.17 4.10 2.02 1.02 1.07 3.07 8.09 6.95 2.70 2.76 2.81 3.80 4.04 0.93 0.99 2.29 0.71 1.08 1.83 2.01 2.79 1.05 5.10 1.02 4.97 2.35 0.75 4.80 4.01 1.14 2.70 2.08 2.76 2.80 2.00
f1 (ppm)

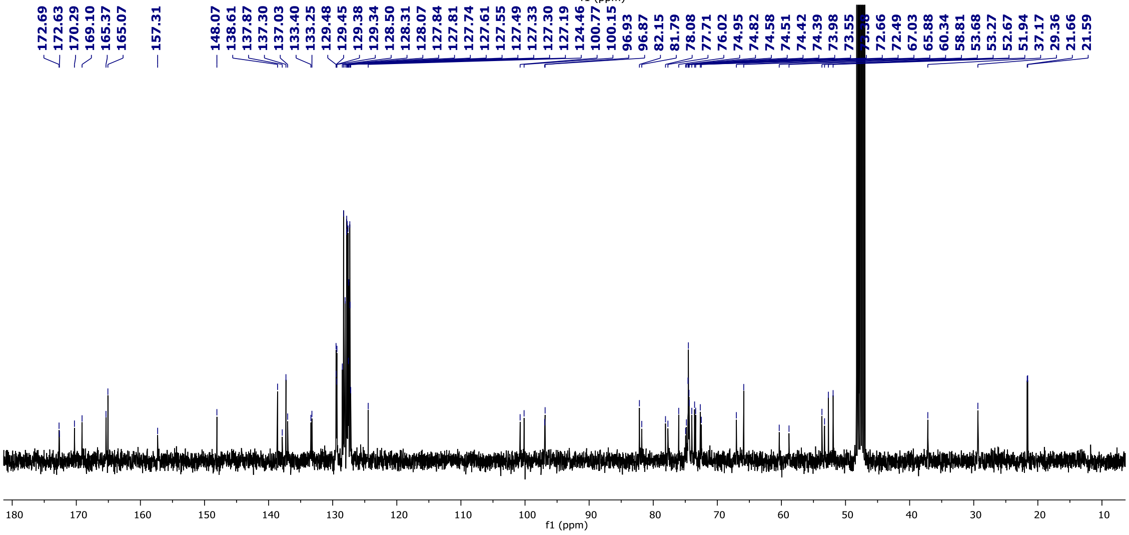
172.69 172.63 170.29 169.10 165.37 165.07 157.31 148.07 138.61 137.87 137.30 137.03 133.40 133.25 129.48 129.45 129.38 129.34 128.50 128.31 128.07 127.84 127.81 127.74 127.61 127.55 127.49 127.33 127.30 127.19 124.46 100.77 100.15 96.93 96.87 82.15 81.79 78.08 77.71 76.02 74.95 74.82 74.58 74.51 74.42 74.39 73.98 73.55 72.66 72.49 67.03 65.88 60.34 58.81 53.68 53.27 52.67 51.94 37.17 29.36 21.66 21.59
f1 (ppm)

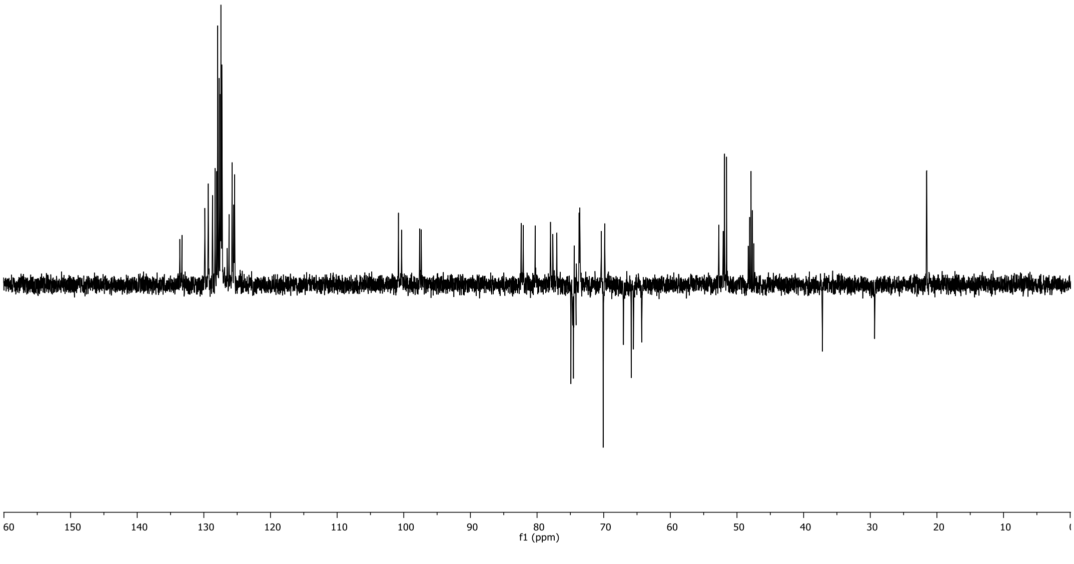
f1 (ppm)

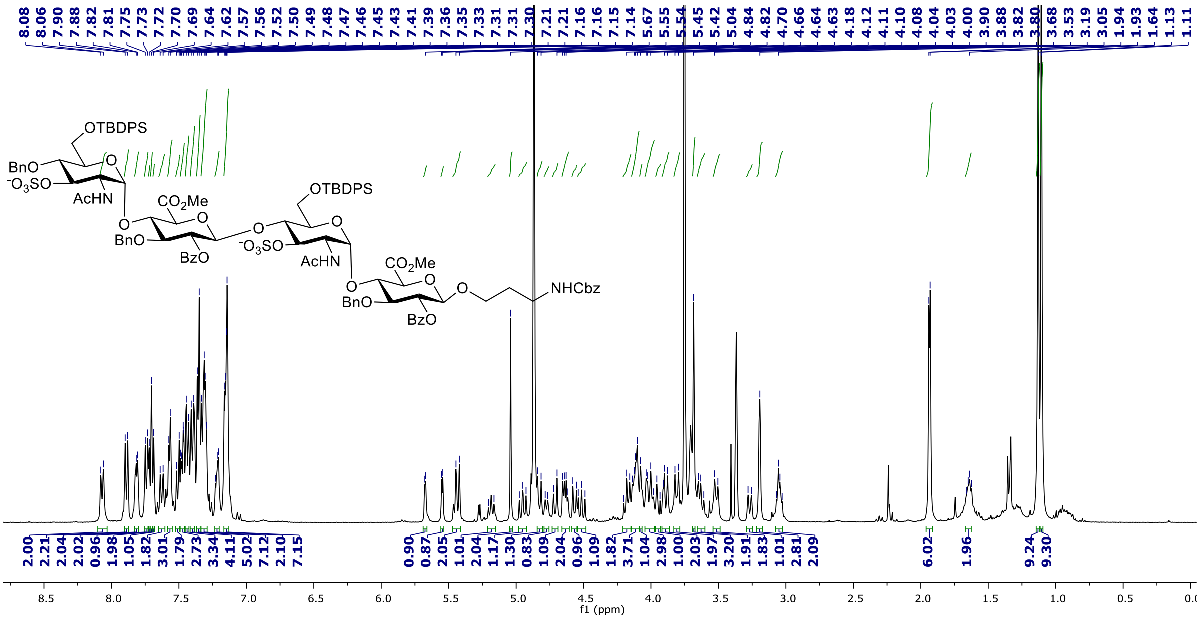
8.08 8.06 7.90 7.88 7.82 7.81 7.75 7.73 7.72 7.70 7.69 7.64 7.62 7.57 7.56 7.52 7.50 7.49 7.48 7.47 7.46 7.45 7.43 7.41 7.39 7.36 7.35 7.33 7.31 7.31 7.30 7.21 7.21 7.16 7.15 7.14 5.67 5.55 5.54 5.45 5.42 5.04 4.84 4.82 4.70 4.66 4.64 4.63 4.18 4.12 4.11 4.10 4.08 4.04 4.03 4.00 3.90 3.88 3.82 3.80 3.68 3.53 3.19 3.05 1.94 1.93 1.64 1.13 1.11
OTBDPS
BnO
$^{-}O_3SO$
AcHN
CO_2Me
BnO
BzO
OTBDPS
$^{-}O_3SO$
AcHN
CO_2Me
BnO
BzO
NHCbz
2.00 2.21 2.04 2.02 0.96 1.98 1.05 1.82 3.01 1.79 2.73 3.34 4.11 5.02 7.12 2.10 7.15 0.90 0.87 2.05 1.01 2.04 1.17 1.30 0.83 1.09 2.04 0.96 1.09 1.82 3.71 1.04 2.98 1.00 2.03 1.97 3.20 1.91 1.83 1.01 2.81 2.09 6.02 1.96 9.24 9.30
8.5 8.0 7.5 7.0 6.5 6.0 5.5 5.0 4.5 4.0 3.5 3.0 2.5 2.0 1.5 1.0 0.5
f1 (ppm)

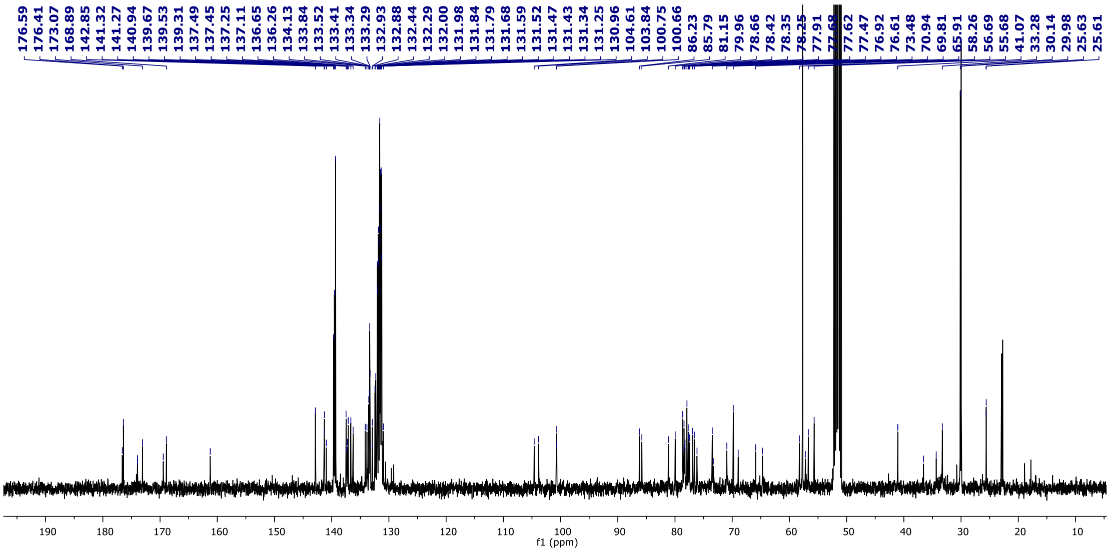
176.59 176.41 173.07 168.89 142.85 141.32 141.27 140.94 139.67 139.53 139.31 137.49 137.45 137.25 137.11 136.65 136.26 134.13 133.84 133.52 133.41 133.34 133.29 132.93 132.88 132.44 132.29 132.00 131.98 131.84 131.79 131.68 131.59 131.52 131.47 131.43 131.34 131.25 130.96 104.61 103.84 100.75 100.66 86.23 85.79 81.15 79.96 78.66 78.42 78.35 78.25 77.91 77.68 77.62 77.47 76.92 76.61 73.48 70.94 69.81 65.91 58.26 56.69 55.68 41.07 33.28 30.14 29.98 25.63 25.61
190 180 170 160 150 140 130 120 110 100 90 80 70 60 50 40 30 20 10
f1 (ppm)

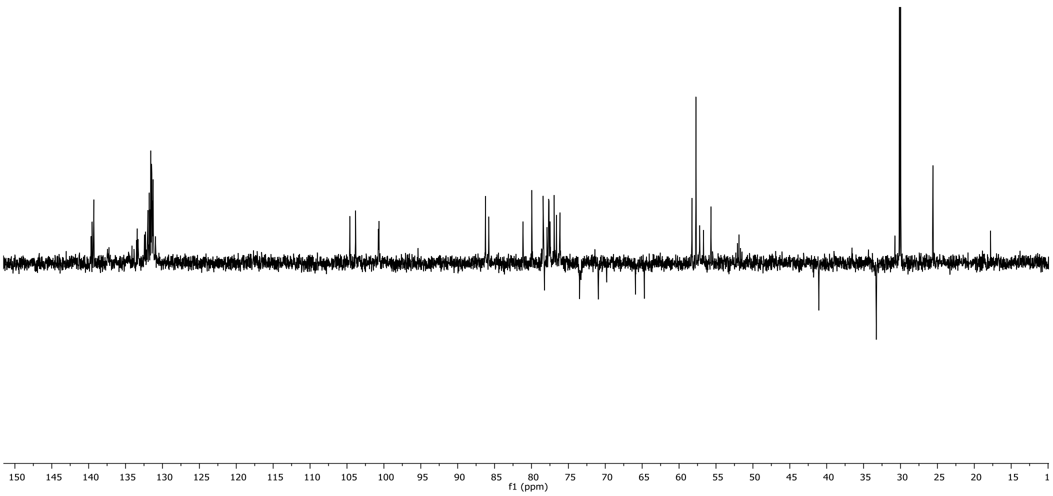
150 145 140 135 130 125 120 115 110 105 100 95 90 85 80 75 70 65 60 55 50 45 40 35 30 25 20 15
f1 (ppm)

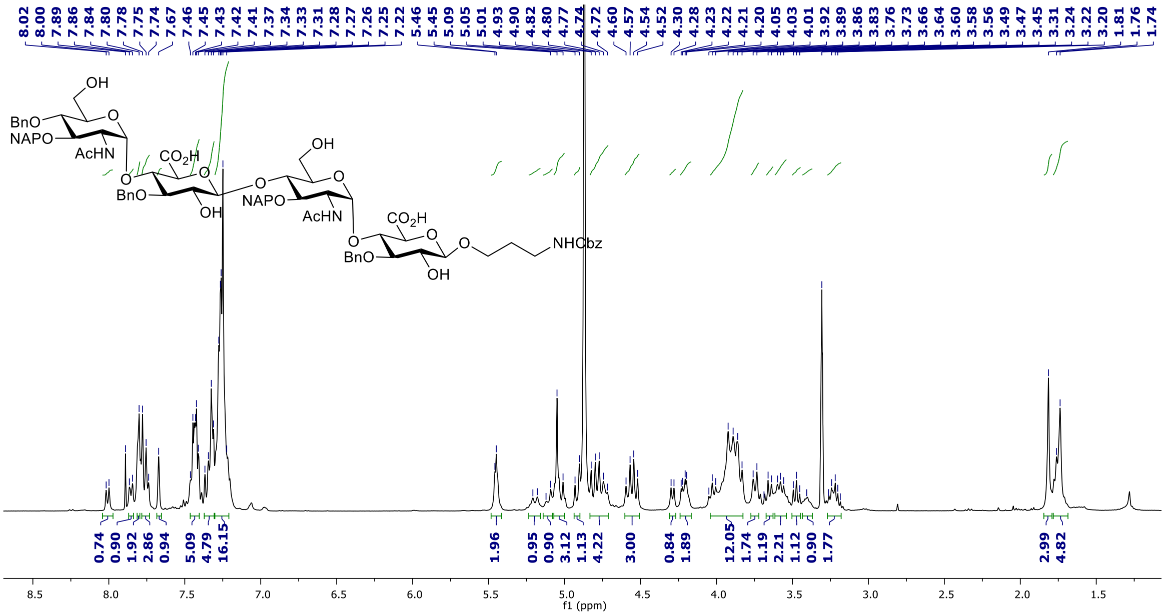
8.02 8.00 7.89 7.86 7.84 7.80 7.78 7.75 7.74 7.67 7.46 7.45 7.43 7.42 7.41 7.37 7.34 7.33 7.31 7.28 7.27 7.26 7.25 7.22 5.46 5.45 5.09 5.05 5.01 4.93 4.90 4.82 4.80 4.77 4.74 4.72 4.60 4.57 4.54 4.52 4.30 4.28 4.23 4.22 4.21 4.20 4.05 4.03 4.01 3.92 3.89 3.86 3.83 3.76 3.73 3.66 3.64 3.60 3.58 3.56 3.49 3.47 3.45 3.31 3.24 3.22 3.20 1.81 1.76 1.74
OH
BnO
NAPO
AcHN
CO_2H
BnO
OH
NAPO
OH
AcHN
CO_2H
BnO
OH
NHCbz
0.74 0.90 1.92 2.86 0.94 5.09 4.79 16.15 1.96 0.95 0.90 3.12 1.13 4.22 3.00 0.84 1.89 12.05 1.74 1.19 2.21 1.12 0.90 1.77 2.99 4.82
8.5 8.0 7.5 7.0 6.5 6.0 5.5 5.0 4.5 4.0 3.5 3.0 2.5 2.0 1.5
f1 (ppm)

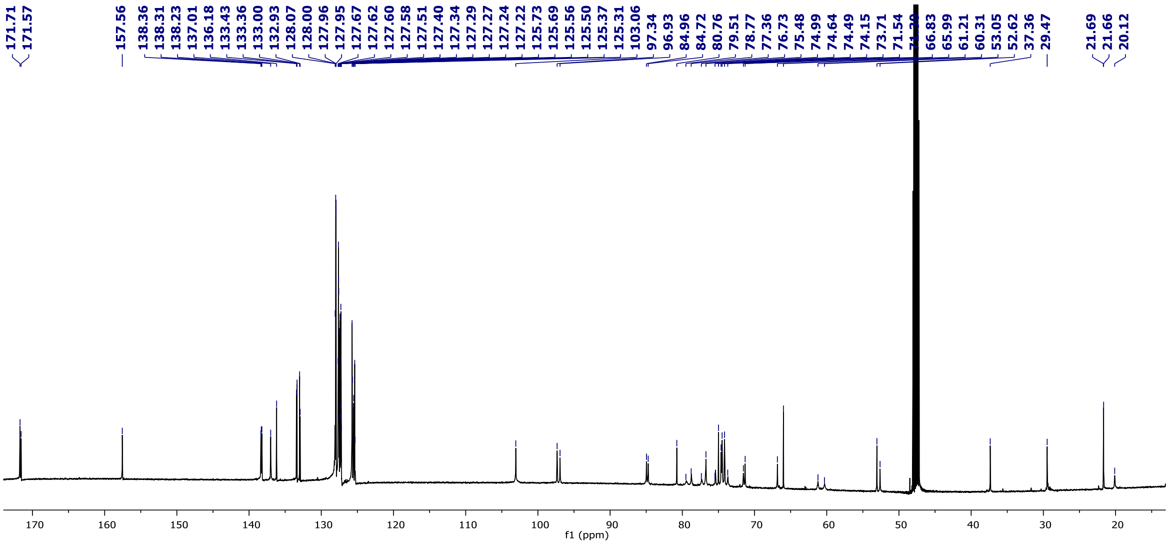
171.71 171.57 157.56 138.36 138.31 138.23 137.01 136.18 133.43 133.36 133.00 132.93 128.07 128.00 127.96 127.95 127.67 127.62 127.60 127.58 127.51 127.40 127.34 127.29 127.27 127.24 127.22 125.73 125.69 125.56 125.50 125.37 125.31 103.06 97.34 96.93 84.96 84.72 80.76 79.51 78.77 77.36 76.73 75.48 74.99 74.64 74.49 74.15 73.71 71.54 66.83 65.99 61.21 60.31 53.05 52.62 37.36 29.47 21.69 21.66 20.12
170 160 150 140 130 120 110 100 90 80 70 60 50 40 30 20
f1 (ppm)

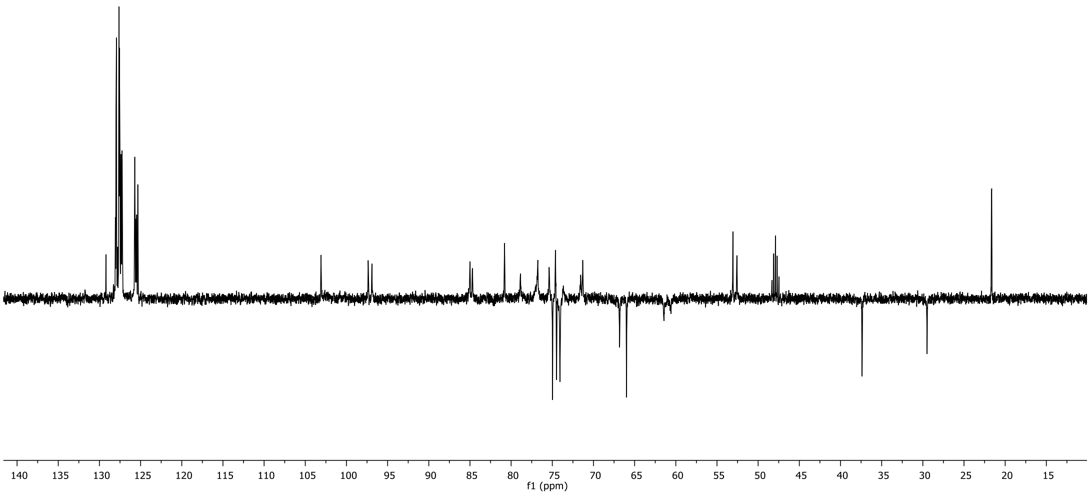
140 135 130 125 120 115 110 105 100 95 90 85 80 75 70 65 60 55 50 45 40 35 30 25 20 15
f1 (ppm)

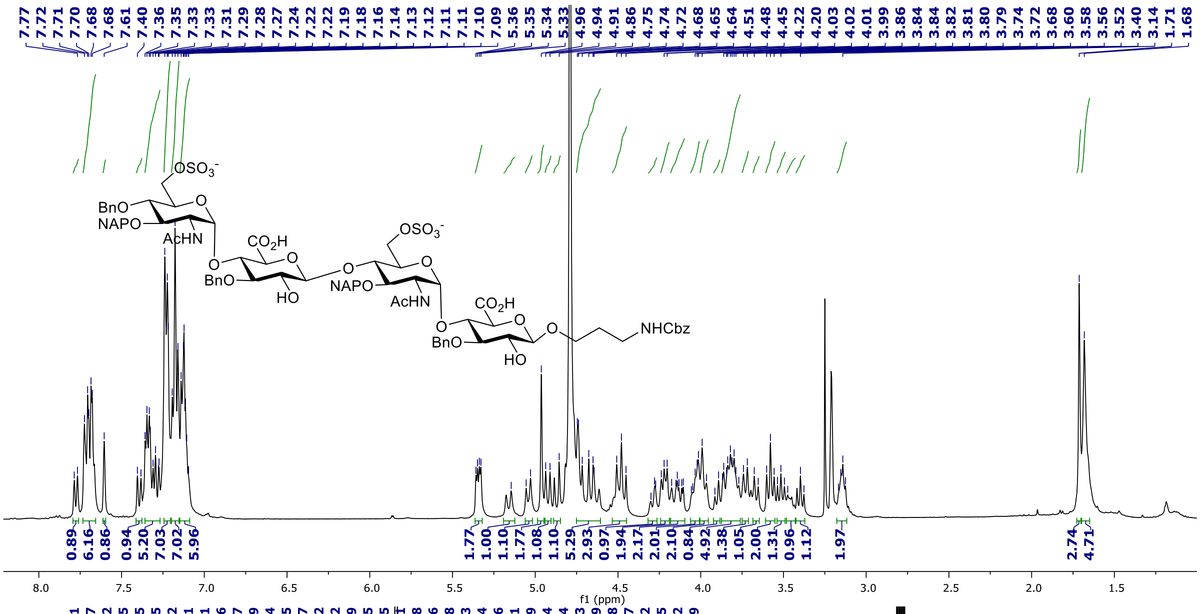
7.77 7.72 7.71 7.70 7.68 7.61 7.40 7.36 7.35 7.33 7.31 7.29 7.28 7.27 7.24 7.22 7.19 7.18 7.16 7.14 7.13 7.12 7.11 7.10 7.09 5.36 5.35 5.34 5.33 4.96 4.94 4.91 4.86 4.75 4.74 4.72 4.68 4.65 4.64 4.51 4.48 4.45 4.22 4.20 4.03 4.02 4.01 3.99 3.86 3.84 3.84 3.82 3.81 3.80 3.79 3.74 3.72 3.68 3.60 3.58 3.56 3.52 3.40 3.14 1.71 1.68
OSO3-
BnO
NAPO
AcHN
O
CO2H
BnO
HO
O
OSO3-
NAPO
AcHN
O
CO2H
BnO
HO
O
NHCbz
0.89 6.16 0.86 0.94 5.20 7.03 7.02 5.96 1.77 1.00 1.10 1.77 1.08 1.10 5.29 2.93 0.97 1.94 2.17 2.01 2.10 0.84 4.92 1.38 1.05 2.00 1.31 0.96 1.12 1.97 2.74 4.71
8.0 7.5 7.0 6.5 6.0 5.5 5.0 4.5 4.0 3.5 3.0 2.5 2.0 1.5
f1 (ppm)

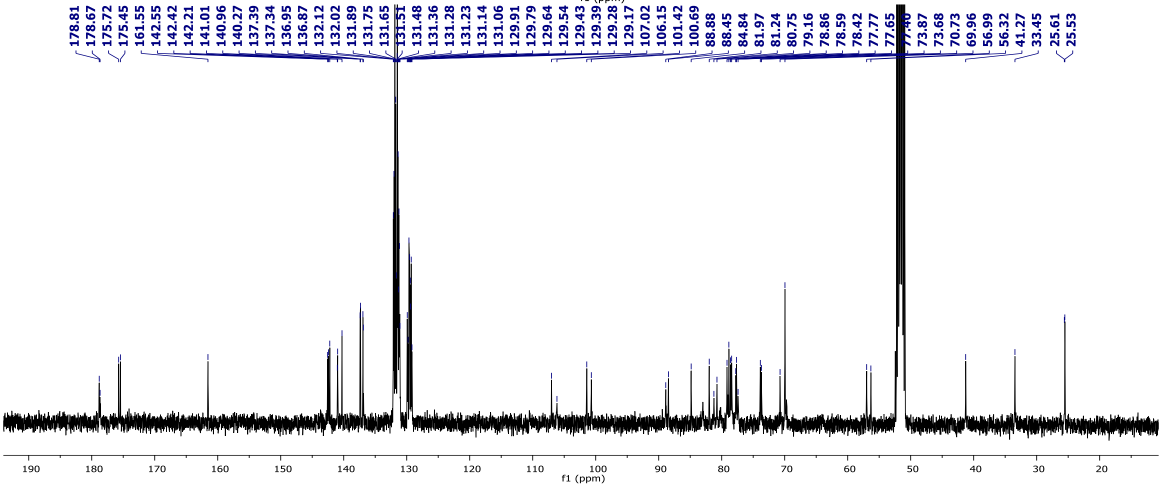
178.81 178.67 175.72 175.45 161.55 142.55 142.42 142.21 141.01 140.96 140.27 137.39 137.34 136.95 136.87 132.12 132.02 131.89 131.75 131.65 131.51 131.48 131.36 131.28 131.23 131.14 131.06 129.91 129.79 129.64 129.54 129.43 129.39 129.28 129.17 107.02 106.15 101.42 100.69 88.88 88.45 84.84 81.97 81.24 80.75 79.16 78.86 78.59 78.42 77.65 77.46 73.87 73.68 70.73 69.96 56.99 56.32 41.27 33.45 25.61 25.53
190 180 170 160 150 140 130 120 110 100 90 80 70 60 50 40 30 20
f1 (ppm)

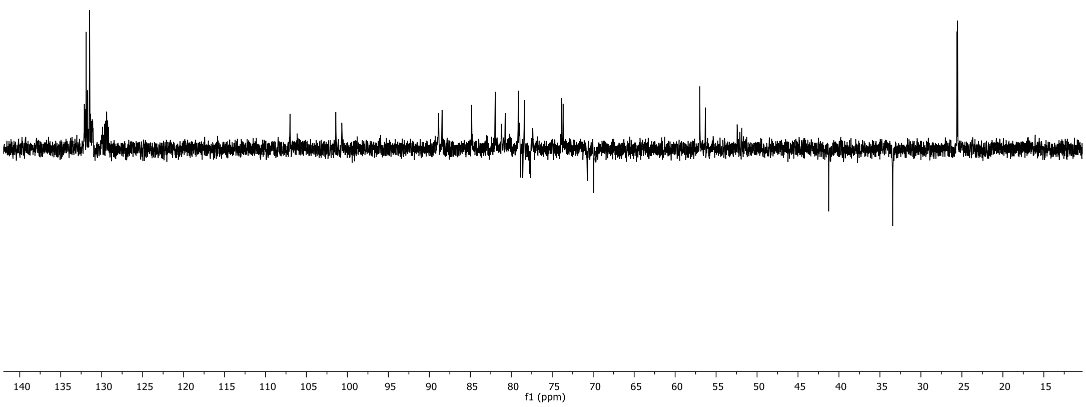
140 135 130 125 120 115 110 105 100 95 90 85 80 75 70 65 60 55 50 45 40 35 30 25 20 15
f1 (ppm)

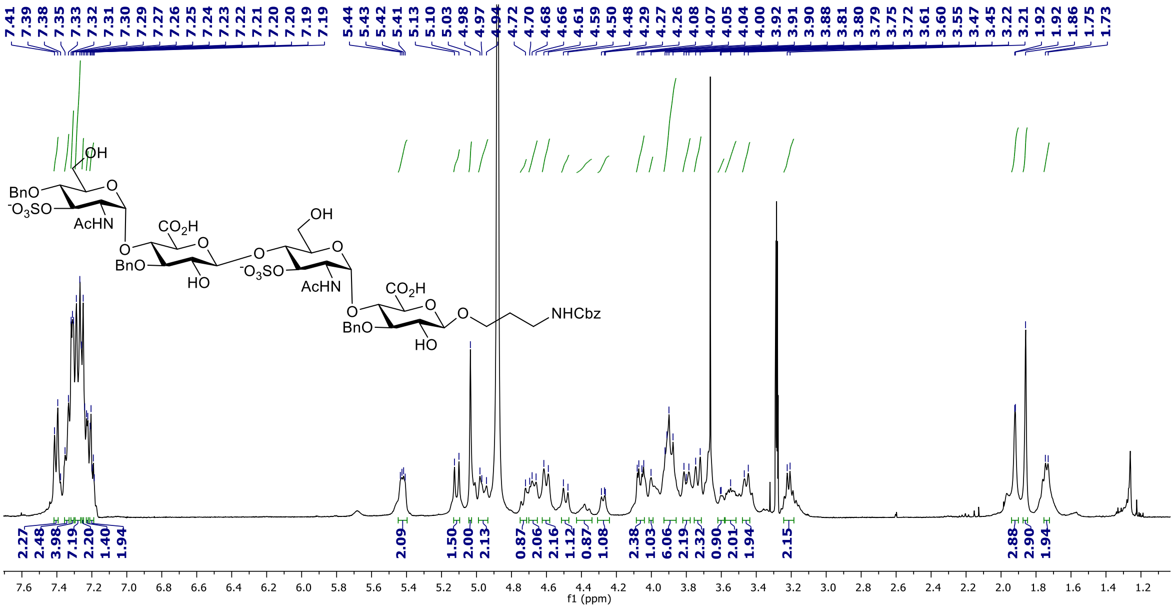
7.41 7.39 7.38 7.35 7.33 7.32 7.31 7.30 7.29 7.27 7.26 7.25 7.24 7.23 7.22 7.21 7.20 7.20 7.19 7.19 5.44 5.43 5.42 5.41 5.13 5.10 5.03 4.98 4.97 4.94 4.72 4.70 4.68 4.66 4.61 4.59 4.50 4.48 4.29 4.27 4.26 4.08 4.07 4.05 4.04 4.00 3.92 3.91 3.90 3.88 3.81 3.80 3.79 3.75 3.72 3.61 3.60 3.55 3.47 3.45 3.22 3.21 1.92 1.92 1.86 1.75 1.73
OH
BnO
$^{-}O_3SO$
AcHN
CO_2H
BnO
HO
OH
$^{-}O_3SO$
AcHN
CO_2H
BnO
HO
NHCbz
2.27 2.48 3.98 7.19 2.20 1.40 1.94 2.09 1.50 2.00 2.13 0.87 2.06 2.16 1.12 0.87 1.08 2.38 1.03 6.06 2.19 2.32 0.90 2.01 1.94 2.15 2.88 2.90 1.94
f1 (ppm)

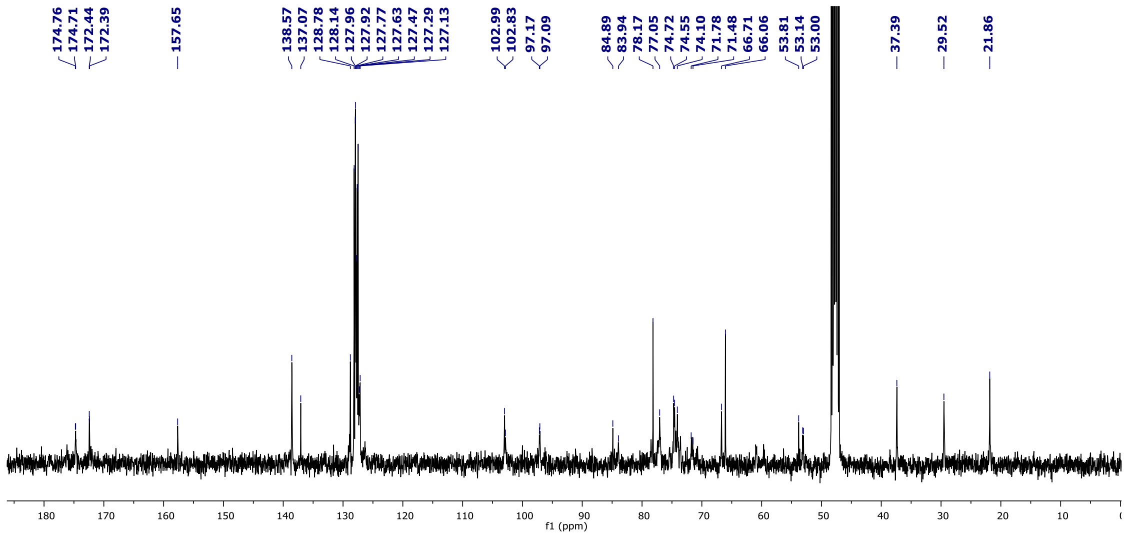
174.76 174.71 172.44 172.39 157.65 138.57 137.07 128.78 128.14 127.96 127.92 127.77 127.63 127.47 127.29 127.13 102.99 102.83 97.17 97.09 84.89 83.94 78.17 77.05 74.72 74.55 74.10 71.78 71.48 66.71 66.06 53.81 53.14 53.00 37.39 29.52 21.86
f1 (ppm)

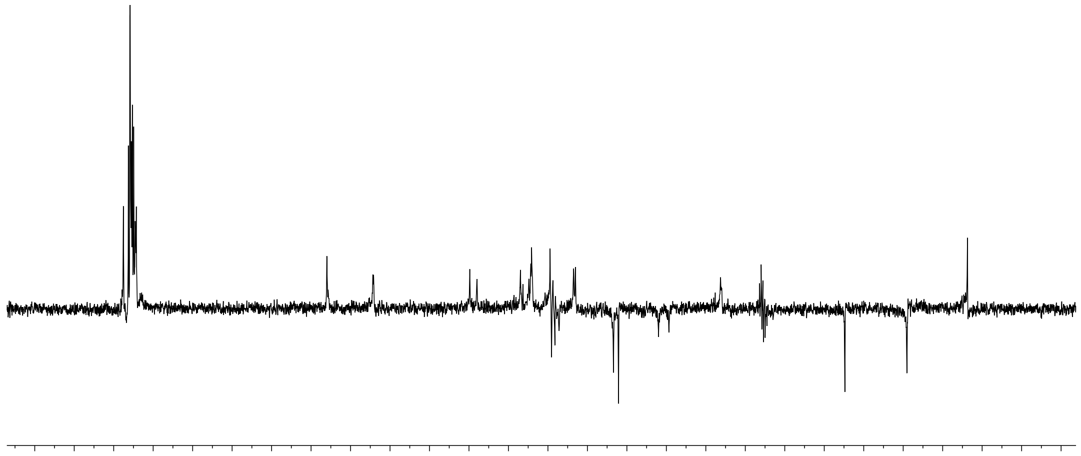
f1 (ppm)

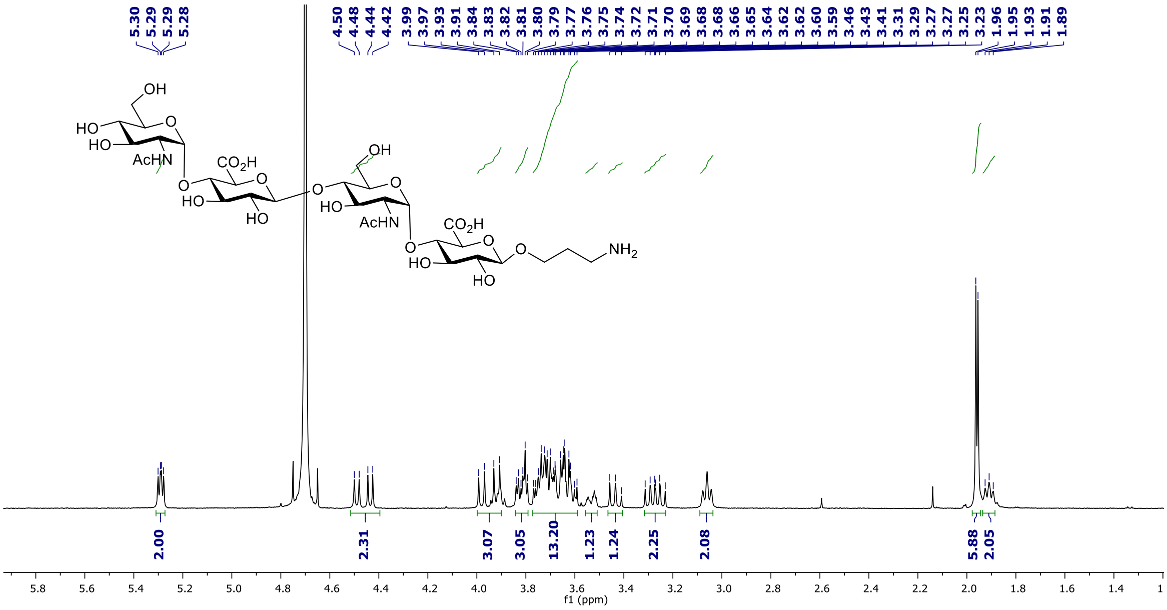
5.30 5.29 5.29 5.28
4.50 4.48 4.44 4.42
3.99 3.97 3.93 3.91 3.84 3.83 3.82 3.81 3.80 3.79 3.77 3.76 3.75 3.74 3.72 3.71 3.70 3.69 3.68 3.68 3.66 3.65 3.64 3.62 3.62 3.60 3.59 3.46 3.43 3.41 3.31 3.29 3.27 3.25 3.23 1.96 1.95 1.93 1.91 1.89
OH
HO
HO
AcHN
O
CO2H
HO
HO
AcHN
CO2H
NH2
2.00
2.31
3.07
3.05
13.20
1.23
1.24
2.25
2.08
5.88
2.05
f1 (ppm)

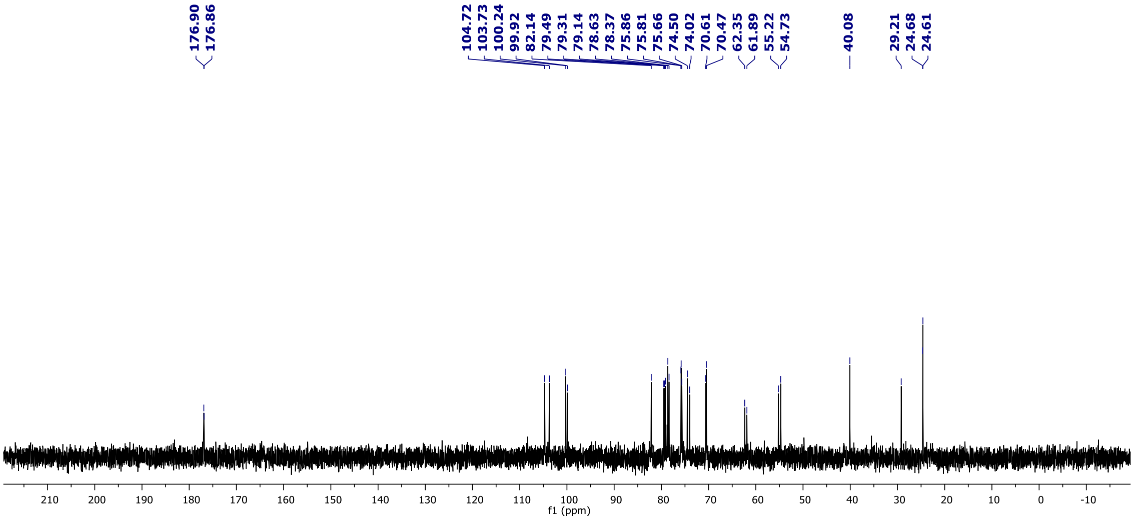
176.90 176.86
104.72 103.73 100.24 99.92 82.14 79.49 79.31 79.14 78.63 78.37 75.86 75.81 75.66 74.50 74.02 70.61 70.47 62.35 61.89 55.22 54.73
40.08
29.21 24.68 24.61
f1 (ppm)

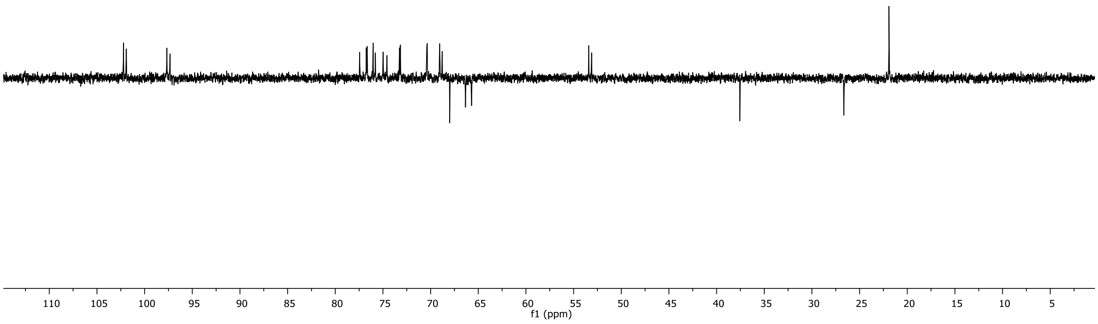
f1 (ppm)

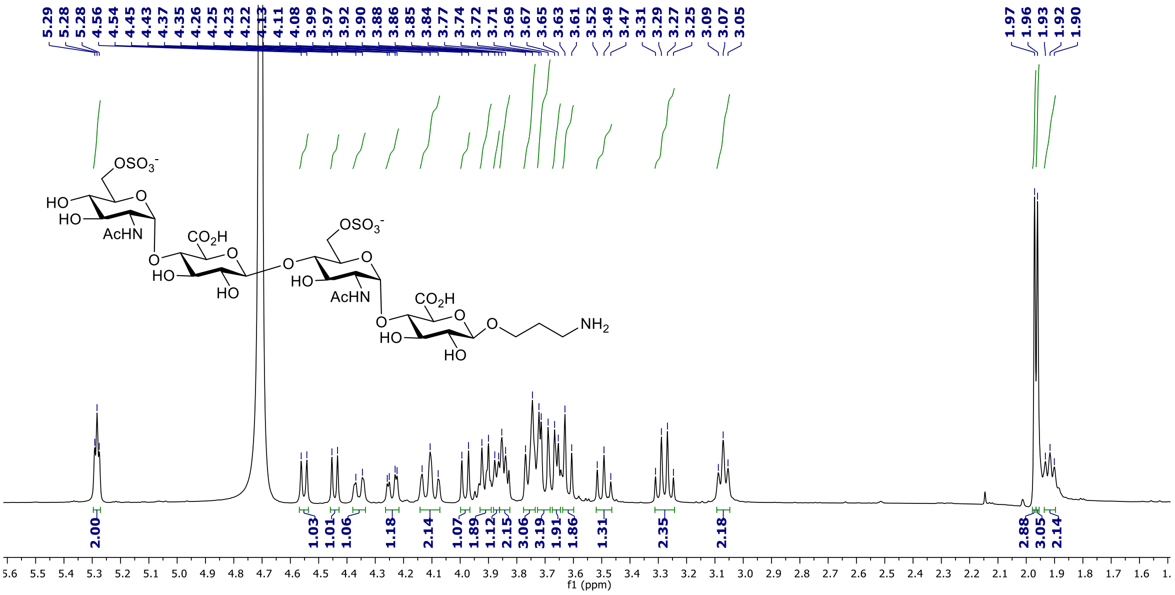
5.29 5.28 5.28 4.56 4.54 4.45 4.43 4.37 4.35 4.26 4.25 4.23 4.22 4.13 4.11 4.08 3.99 3.97 3.92 3.90 3.88 3.86 3.85 3.84 3.77 3.74 3.72 3.71 3.69 3.67 3.65 3.63 3.61 3.52 3.49 3.47 3.31 3.29 3.27 3.25 3.09 3.07 3.05
1.97 1.96 1.93 1.92 1.90
OSO_3^-
HO
HO
AcHN
O
CO_2H
HO
HO
OSO_3^-
HO
AcHN
CO_2H
HO
HO
NH_2
2.00 1.03 1.01 1.06 1.18 2.14 1.07 1.89 1.12 2.15 3.06 3.19 1.91 1.86 1.31 2.35 2.18 2.88 3.05 2.14
f1 (ppm)

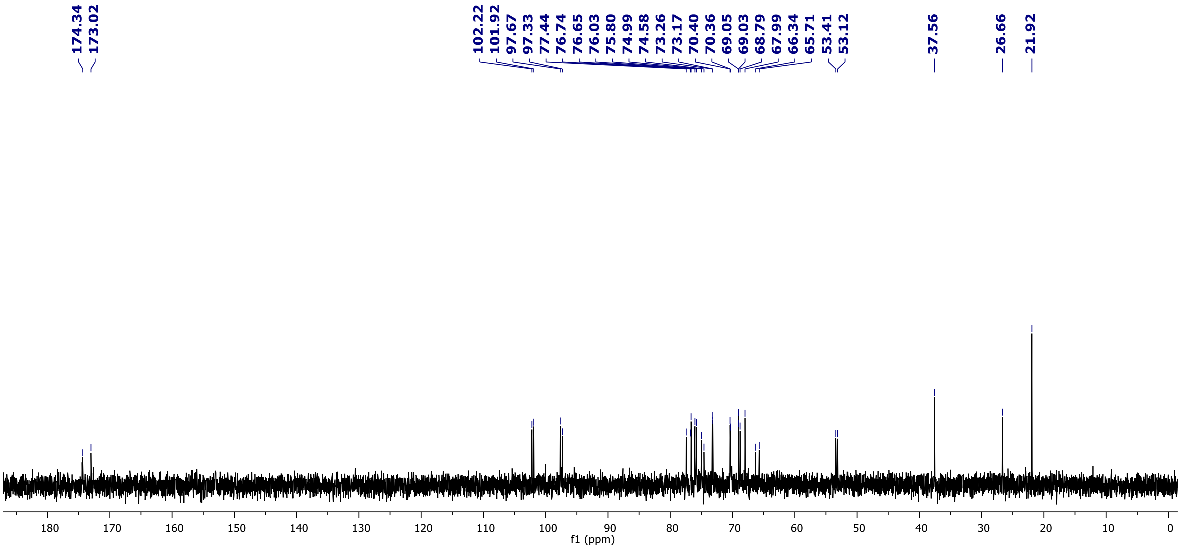
174.34 173.02
102.22 101.92 97.67 97.33 77.44 76.74 76.65 76.03 75.80 74.99 74.58 73.26 73.17 70.40 70.36 69.05 69.03 68.79 67.99 66.34 65.71 53.41 53.12
37.56
26.66
21.92
f1 (ppm)

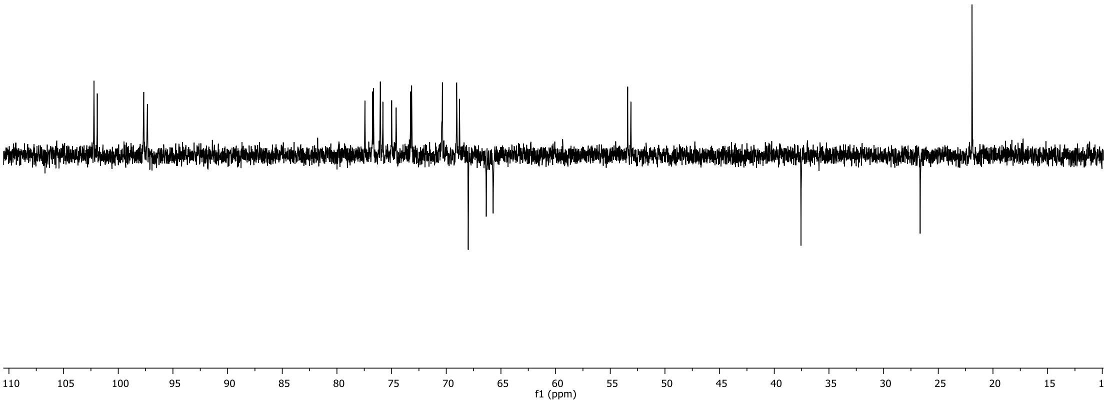
f1 (ppm)

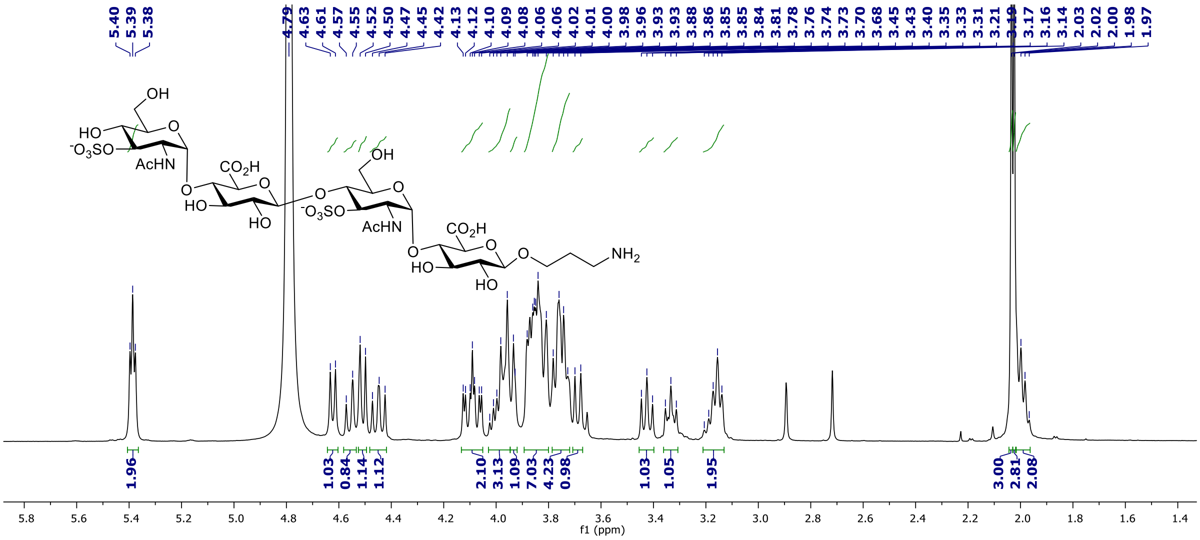
f1 (ppm)

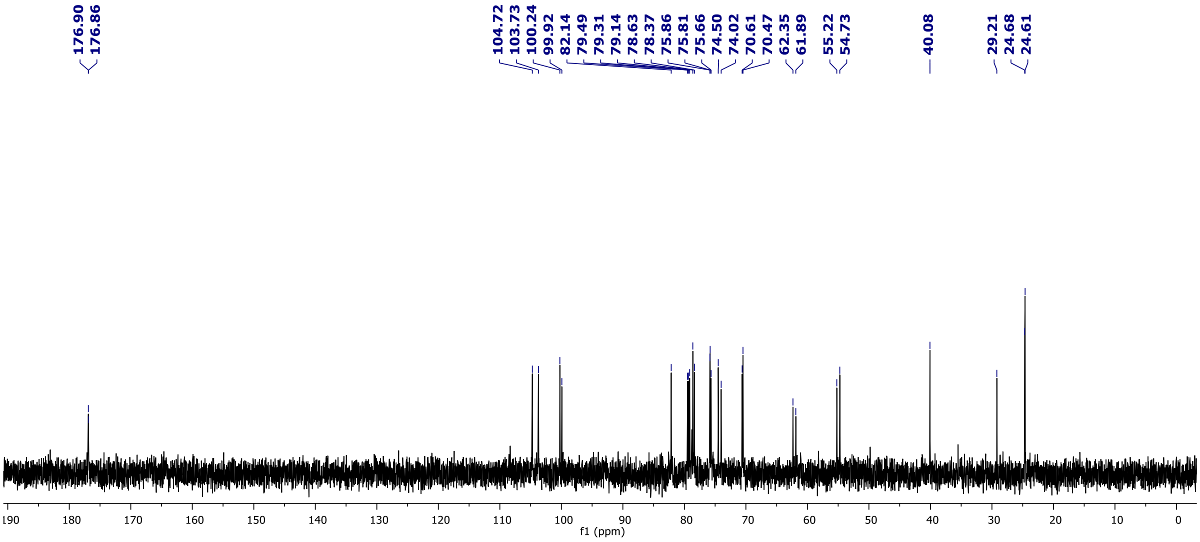
f1 (ppm)

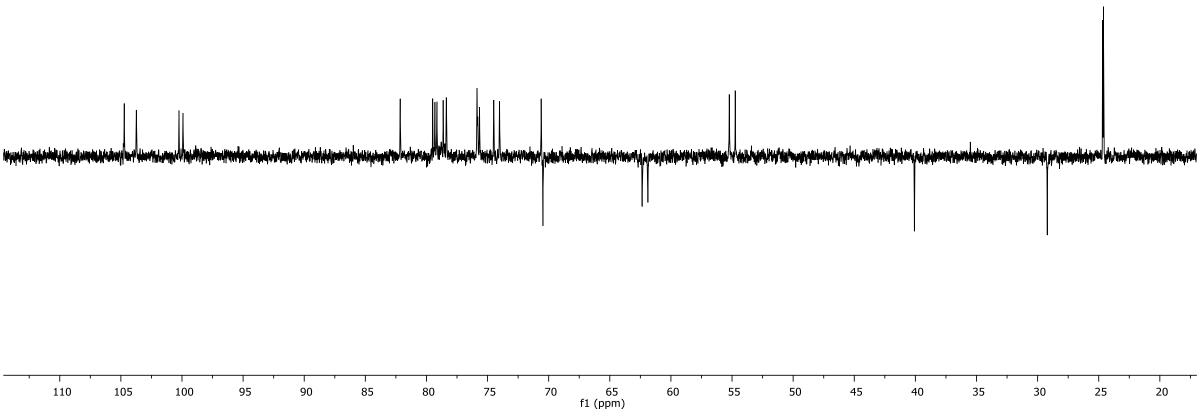
f1 (ppm)

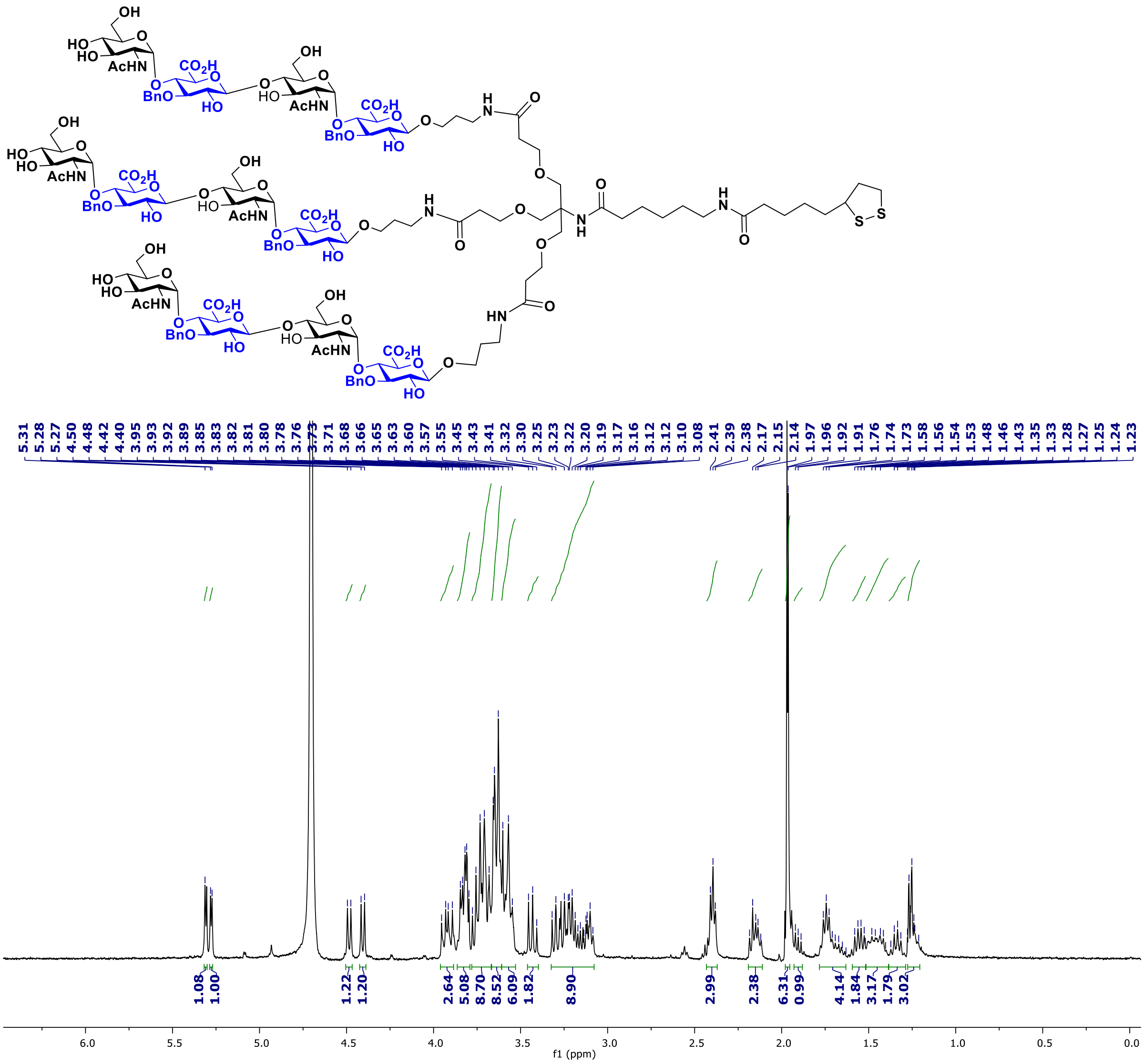
f1 (ppm)
6.0
5.5
5.0
4.5
4.0
3.5
3.0
2.5
2.0
1.5
1.0
0.5
0.0

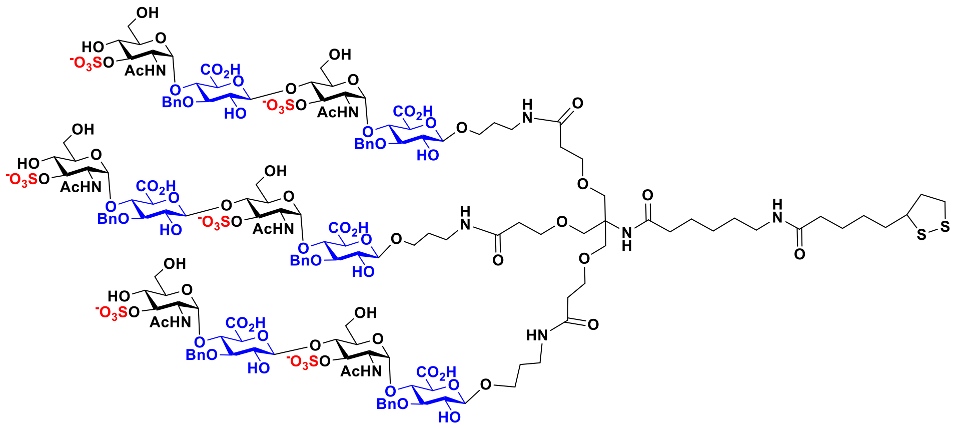

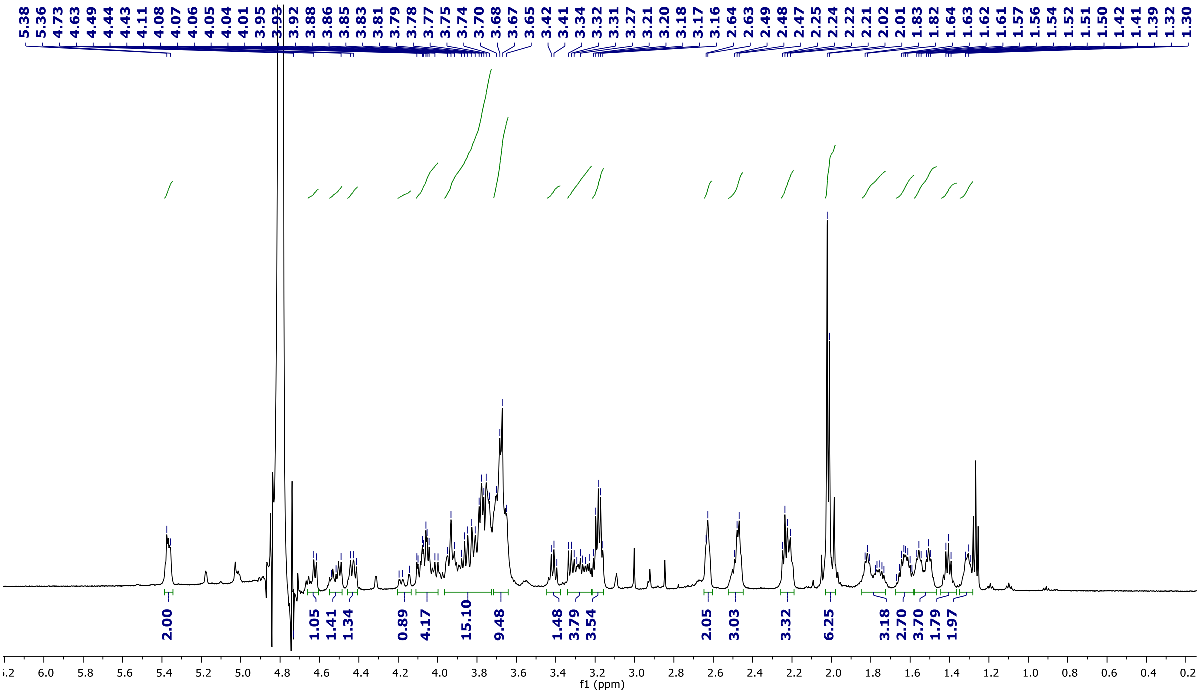
2.00
1.05
1.41
1.34
0.89
4.17
15.10
9.48
1.48
3.79
3.54
2.05
3.03
3.32
6.25
3.18
2.70
3.70
1.79
1.97
f1 (ppm)

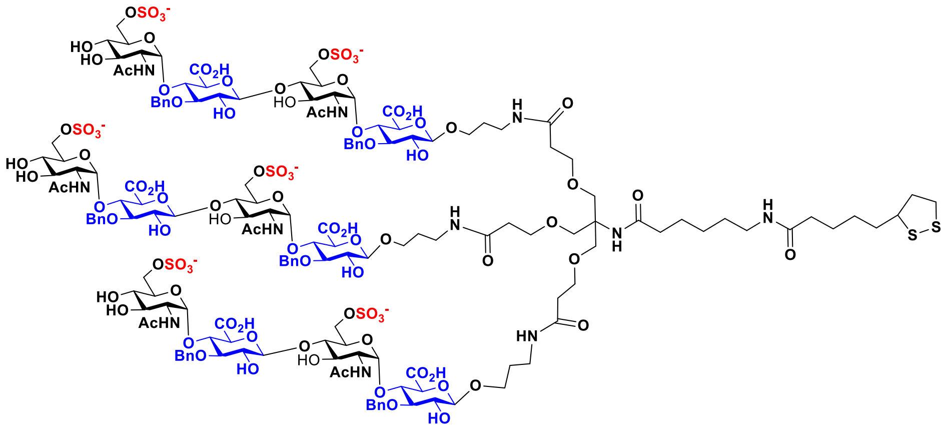
OSO3-
HO
HO
AcHN
CO2H
BnO
HO
NH
O
S-S

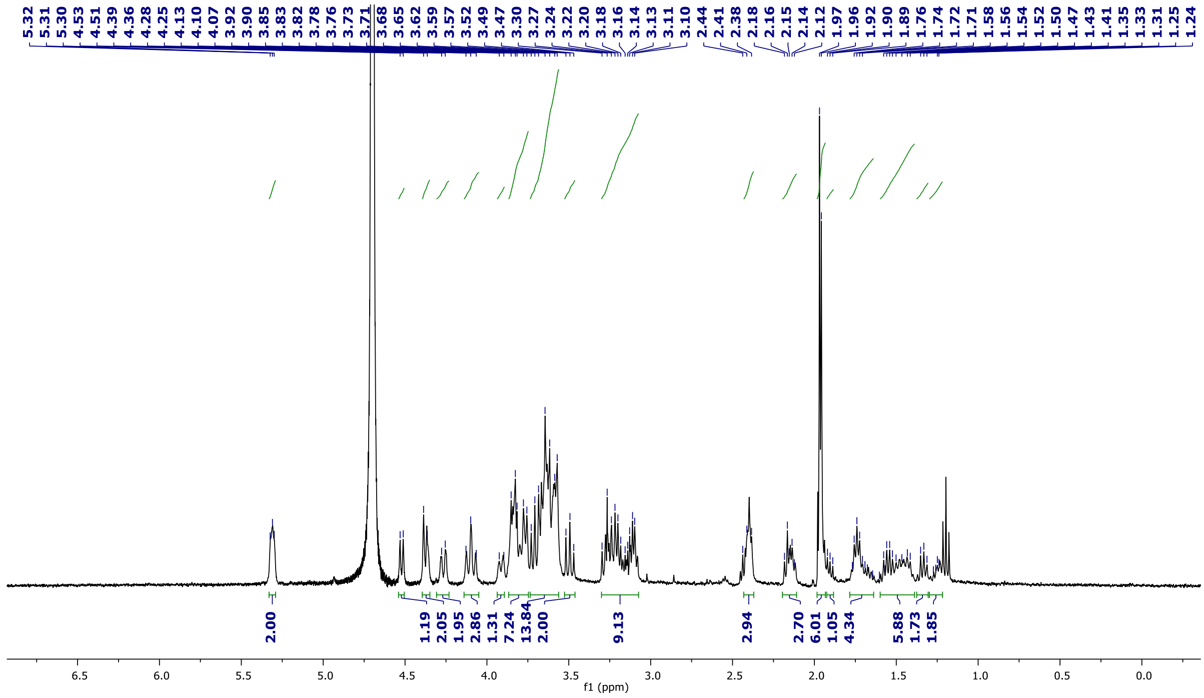
5.32 5.31 5.30 4.53 4.51 4.39 4.36 4.28 4.25 4.13 4.10 4.07 3.92 3.90 3.85 3.83 3.82 3.78 3.76 3.73 3.71 3.68 3.65 3.62 3.59 3.57 3.52 3.49 3.47 3.30 3.27 3.24 3.22 3.20 3.18 3.16 3.14 3.13 3.11 3.10 2.44 2.41 2.38 2.18 2.16 2.15 2.14 2.12 1.97 1.96 1.92 1.90 1.89 1.76 1.74 1.72 1.71 1.58 1.56 1.54 1.52 1.50 1.47 1.43 1.41 1.35 1.33 1.31 1.25 1.24
2.00
1.19
2.05
1.95
2.86
1.31
7.24
13.84
2.00
9.13
2.94
2.70
6.01
1.05
4.34
5.88
1.73
1.85
6.5 6.0 5.5 5.0 4.5 4.0 3.5 3.0 2.5 2.0 1.5 1.0 0.5 0.0
f1 (ppm)
